# Compressional Behavior of Naphthalene (C_10_H_8_) and Anthracene (C_14_H_10_) up to 50 GPa

**DOI:** 10.1021/acsomega.5c06935

**Published:** 2025-10-19

**Authors:** Wenju Zhou, Xiang Li, Fariia Iasmin Akbar, Anna Pakhomova, Michael Hanfland, Leonid Dubrovinsky, Natalia Dubrovinskaia

**Affiliations:** † Material Physics and Technology at Extreme Conditions, Laboratory of Crystallography, 26523 University of Bayreuth, 95440 Bayreuth, Germany; ‡ Bayerisches Geoinstitut, University of Bayreuth, 95440 Bayreuth, Germany; § European Synchrotron Radiation Facility, CS 40220, 38043 Grenoble Cedex 9, France; ∥ Department of Physics, Chemistry and Biology (IFM), Linköping University, SE-581 83 Linköping, Sweden; ⊥ Institut für Mineralogie, University of Münster, Corrensstr. 24, 48149 Münster, Germany

## Abstract

In this study, we explored the behavior of naphthalene and anthracene under compression to ∼50 and ∼42 GPa, respectively, using synchrotron single-crystal X-ray diffraction (SCXRD) in diamond anvil cells. Both compounds demonstrate remarkable structural stability, with no phase transitions being observed. The equations of states were obtained with the following parameters: *V*_0_ = 182.2(2) Å^3^, *K*_0_ = 8.4(10) GPa, and *K*′ = 5.5(6) for naphthalene, and *V*_0_ = 238.0(2) Å^3^, *K*_0_ = 8.4(5) GPa, and *K*′ = 8.0(4) for anthracene. Theoretical calculations correctly reproduce experimental data and enable accurate localization of hydrogen atoms. The analysis of Hirshfeld surfaces for both naphthalene and anthracene suggests that the herringbone packing motif of the molecules, the limited contribution of stronger C···C interactions to intermolecular bonding, and the high flexibility of relatively weak H···H interactions allow a gradual compaction of their structures without phase transitions. Our research contributes to the understanding of the compressional mechanisms, bonding evolution, and structural stability of polyaromatic hydrocarbons under compression.

## Graphical abstract

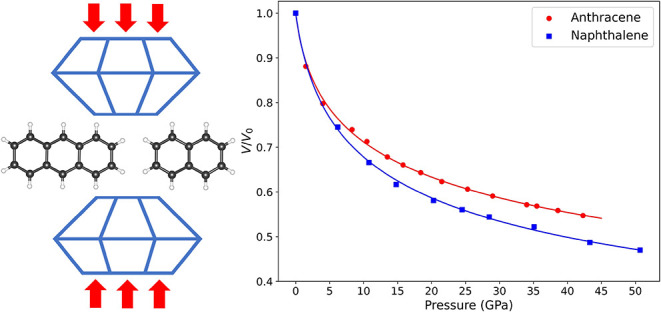

## Introduction

Naphthalene (C_10_H_8_) and anthracene (C_14_H_10_), the first and second members in a series of polycyclic aromatic hydrocarbons (PAHs), are among the representative compounds of this large class of organic materials and have long served as model solids. The crystal structure, molecular packing, and relative orientation of molecules within a molecular crystal can be significantly influenced by changes in temperature, pressure, and electric or magnetic fields. Due to the relatively weak intermolecular interactions in PAHs, the properties of these solids are highly responsive to applied pressure, but have not been sufficiently studied so far.

The crystal structure of naphthalene and anthracene have been investigated extensively at ambient conditions. The structure of naphthalene was first determined by Abrahams et al. using powder X-ray diffraction (PXRD),^1^ who identified a monoclinic cell with space group *P*2_1_/*a*. Later Natkaniec et al. applied single-crystal neutron diffraction at 12 K,^2^ while Oddershede and Larsen refined the structure at various temperatures by single-crystal X-ray diffraction (SC-XRD),^3^ both providing precise atomic coordinates. Similarly, anthracene was first refined by Cruickshank in 1956 using PXRD,^4^ and subsequently by Lehmann and Pawley via neutron diffraction at 12 K on perdeuteroanthracene (C_14_D_10_),^5^ yielding accurate positions of both C and D atoms. In both compounds, the unit cell contains two molecules arranged in the characteristic herringbone motif common to many PAHs.

High-pressure investigations of naphthalene date back to Bridgman, who reported a small but distinct volume discontinuity around 3 GPa in compression experiments to 5 GPa, suggesting a possible phase transition.^6^ Fluorescence spectroscopy likewise indicated an irreversible molecular change near 3 GPa,^7^ while other volumetric and Raman studies reported no clear evidence of transitions in this range.^8,9^ Infrared data by O’Bannon and Williams extended to 54.5 GPa revealed anomalies at 2–3 GPa, which have been interpreted as indicative of a phase transition, and additional features near ∼30 GPa, as well as likely amorphization between ∼30 and 45 GPa.^10^ PXRD studies by Likhacheva et al. indicated structural changes near 2 GPa,^11^ but their later work found the monoclinic phase stable from 3 to 15 GPa,^12^ in agreement with Shinozaki et al., who reported no transitions up to 20 GPa.^13^ The only SC-XRD study to date, by Fabbiani et al., showed that neither recrystallization at 0.2–0.6 GPa nor direct compression to 2.6 GPa produced a new phase.^14^ For anthracene, spectroscopic data similarly pointed to possible low-pressure transitions but without consensus. Fluorescence (0–2.5 GPa),^15^ infrared (up to 4.5 GPa),^16^ and Raman (up to 3.1 GPa)^17^ studies suggested changes in this regime, whereas O’Bannon and Williams observed transitions at ∼2–3 GPa and possibly near 7 GPa in infrared spectra to 19.9 GPa.^10^ Leger and Aloualiti reported PXRD up to 5 GPa, noting a possible second-order or weakly first-order transformation near 2.4 GPa,^18^ while Oehzelt et al. found no transition up to 27.8 GPa.^19^ As seen, structural crystallographic data are rare. To our knowledge, no high-pressure SC-XRD data are available for anthracene.

As already mentioned, linear PAHs, acenes (naphthalene, anthracene, tetracene (C_18_H_12_), pentacene (C_22_H_14_)), and small- to medium-sized angular PAHs, phenacenes (for example, phenanthrene (C_14_H_10_), pyrene (C_16_H_10_), benzo­[*a*]­pyrene (C_20_H_12_), and benzo­[*a*]­anthracene (C_18_H_12_)), adopt herringbone packing. This structural motif is also observed in the simplest aromatic hydrocarbon, benzene (C_6_H_6_), which, although not formally a PAH, is clearly related to the systems discussed here. Comparative studies of the high-pressure behavior of these compounds can therefore provide valuable insight into the compressional mechanisms and the evolution of chemical bonding in PAHs. However, high-pressure structural investigations of these systems using SC-XRD remain scarce and are limited to very low pressures. For example, benzene has been studied by SC-XRD only up to 5 GPa,^20^ and phenanthrene only up to 0.7 GPa.^14^ Although the pressure range of studies on benzene,^21^ phenanthrene,^22,23^ tetracene,^24,25^ benzo­[*a*]­anthracene,^26^ and pentacene^25,27,28^ (to mention some) has been extended beyond these very low values, these investigations rely exclusively on PXRD, spectroscopy, or combinations of PXRD and/or spectroscopy with theoretical calculations. Consequently, they do not provide unambiguous structural information.

For the first time, SC-XRD studies of PAHs were conducted by our group at pressures as high as 35 GPa for pyrene^29^ and 28 GPa for benzo­[*a*]­pyrene.^30^ These studies provided accurate structural data, revealed polymorphism, and offered insight into the evolution of bonding and the mechanisms of compression in these two representatives of phenacenes. The present work continues our systematic investigations of PAHs under pressure. It contributes to the growing body of knowledge on the compressional behavior of the two simplest acenes, naphthalene and anthracene, and addresses inconsistencies in earlier literature reports obtained by other methods. Accordingly, we conducted synchrotron SC-XRD experiments in diamond anvil cells (DACs), examining the behavior of naphthalene in the pressure range from ambient to 50 GPa and anthracene from ambient to 43 GPa, with the aim of obtaining accurate crystallographic data. Notably, no phase transitions were observed in either compound within these pressure ranges. The results of these investigations are presented below.

## Experimental Section

### Samples and Diamond Anvil Cells Preparation

Crystalline powders of anthracene and naphthalene of >98% purity were purchased from Merck. Single crystals of anthracene and naphthalene were selected under an optical microscope and preselected for high-pressure XRD studies in DAC#1 (with naphthalene) and DAC#3 (with anthracene) at ambient pressure (see Table S1 for the summary of all experiments). Two high-quality crystals of anthracene and two of naphthalene, along with a piece of ruby, were then loaded into membrane-type DAC#2 (with naphthalene) and DAC#4 (with anthracene), each equipped with Boehler-Almax type diamonds,^31^ with culet sizes of 250 μm, and a rhenium gasket with a hole of ∼120 μm in diameter and a thickness of ∼30 μm. Helium (He) was used as the pressure-transmitting medium (PTM). The DAC#2 was gradually pressurized from ∼6 to ∼53 GPa, and DAC#4 from ∼1.5 to ∼45 GPa. Pressure was determined using quasi-hydrostatic ruby pressure scale^32^ and uncertainty in pressure measurements was better than 0.3 GPa at pressures up to ∼50 GPa.

### Single-Crystal XRD Experiments

SC-XRD studies at room temperature were conducted in DAC #1 and DAC #2 on the ID27 beamline (λ = 0.3738 Å, ESRF) with a beam size of approximately 2 × 2 μm^2^, and in DAC #3 and DAC #4 on the ID15B beamline (λ = 0.4100 Å, ESRF) with a beam size of approximately 1.5 × 1.5 μm^2^. In both experiments, a micrograin of tungsten was placed in the center of the pressure chamber along with the sample. The strong X-ray absorption signal of tungsten was used to align DACs at rotation center. At each pressure step, the data were collected in step-scans of 0.5° upon rotating the DAC from −34 to +34° about the vertical axis (ω-scan). For single-crystal data analysis (peak search, unit cell finding, and intensity integration), the CrysAlisPro Software was employed,^33^ whereas the crystal structures were solved using SHELX^34^ and refined utilizing the OLEX2 software.^35^ Hydrogen atoms were located using two different methods, the riding model constraint (HFIX instructions)^36^ and Hirshfeld Atom Refinement (HAR),^37^ to automatically constrain their positions in OLEX2. OLEX2 software was used to calculate the interplanar angles (δ) in molecular structures. Crystal structure visualization was made with the VESTA software.^38^ EoSFIT7 software was used to fit the pressure–volume data.^39^

### Theoretical Calculations

Our density functional theory (DFT) calculations were performed using the Vienna ab initio simulation package (VASP)^40^ with the Projector-Augmented-Wave (PAW) method.^41^ The Generalized Gradient Approximation (GGA) functional was used for calculating the exchange-correlation energy, as proposed by Perdew–Burke–Ernzerhof (PBE).^42^ Additionally, we employed the DFT-D3 method for dispersion correction.^43^ The Brillouin zone was sampled with a 5 × 6 × 5 Monkhorst–Pack special *k*-point grid for naphthalene, and 4 × 6 × 5 for anthracene.^44^ Furthermore, the valence states 2s^2^2p^2^ for C and 1s^1^ for H were used with the energy cutoff of 520 eV for the plane wave basis set. The geometries were optimized until the remaining atomic forces were less than 5 × 10^–3^ eV/Å and the energy convergence criterion was set at 10^–5^ eV.

## Results and Discussion

### Structure of Naphthalene

The structure of naphthalene determined at ambient conditions in this work (Figure 1a) is similar to previously reported monoclinic structures (space group *P*2_1_/*c*, #14) and has the following unit cell parameters: *a* = 8.147(6) Å, *b* = 6.0035(8) Å, *c* = 8.293(3) Å, β = 116.08(7)°, and *V* = 364.3(4) Å^3^. The unit cell contains two naphthalene molecules (*Z* = 2), which are crystallographically equivalent and reside on the inversion center. In the structure they are arranged in a herringbone packing motif stabilized by C–H···π interactions between adjacent molecules (Figure 1a). A comparison with the crystallographic data, refined using SC-XRD at 205 K^3^ and single-crystal neutron diffraction at 295 K,^45^ as well as with the structure data for perdeuteronaphthalene (C_10_D_8_), refined using single-crystal neutron diffraction at 12 K^2^, can be found in Table S2. This comparison confirms that our results are consistent with previous reports.

**Figure 1:**
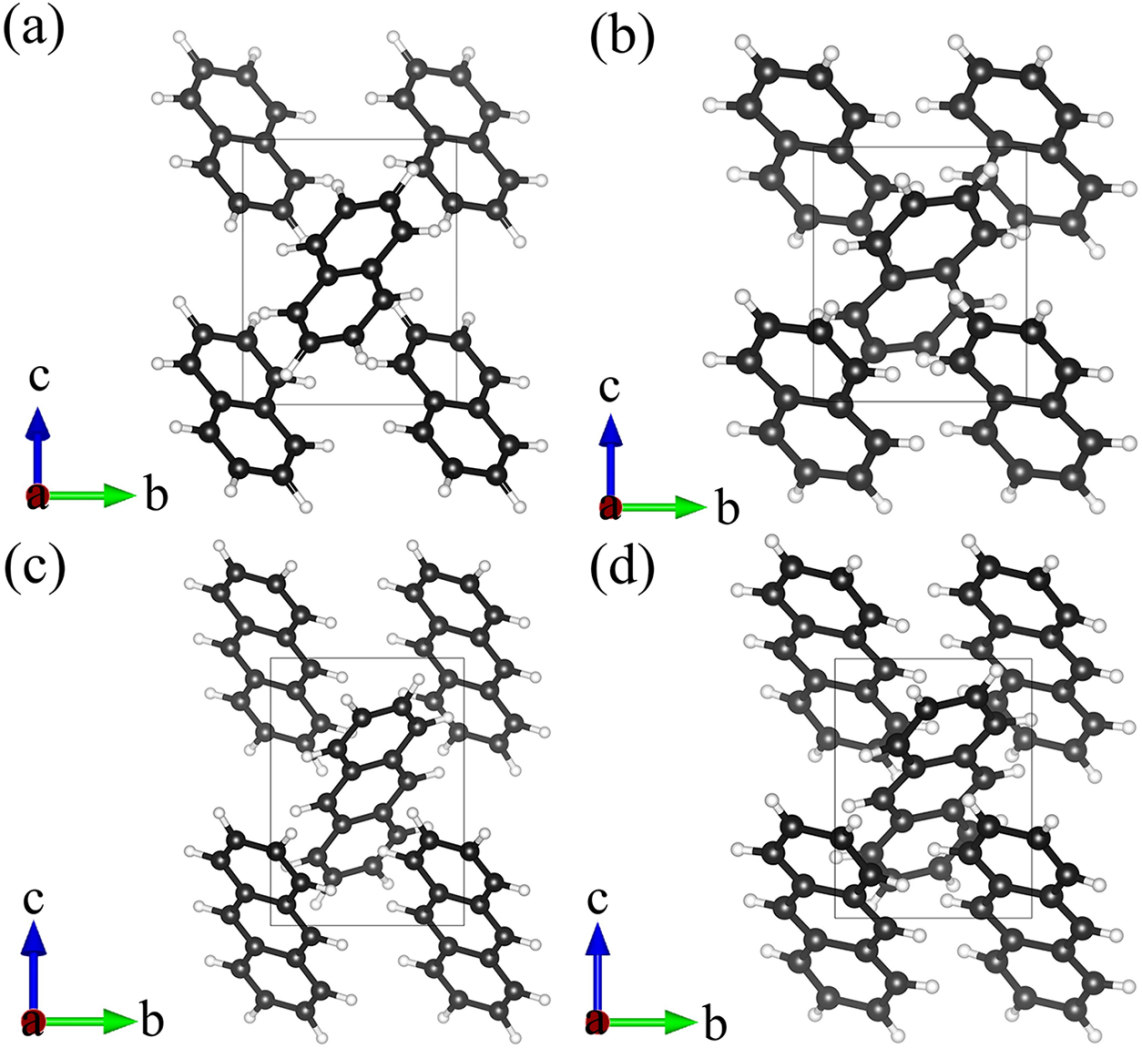
Crystal structures of naphthalene and anthracene viewed along the [100] direction. Naphthalene (a) at ambient conditions, (b) at 50.7 GPa; anthracene (c) at ambient conditions, (d) at 42.3 GPa. C atoms are black. H atoms are white.

### Compressional Behavior of Naphthalene

Upon compression up to 50.7 GPa in a He pressure medium, we did not observe any phase transition. At the next pressure step of 53 GPa, the XRD pattern disappeared, probably because the crystal was bridged between anvils. Full crystallographic and experimental data for naphthalene at different pressures are provided in Table S3.

The dependences of the lattice parameters of naphthalene on pressure are shown in Figure 2a,b (see Table S4 for numerical values). Upon compression, the *a*, *b*, and *c* parameters, as well as the β angle, gradually decrease: the *b*/*b*_0_ ratio-down to 0.82, while *a*/*a*_0_ and *c*/*c*_0_-down to 0.73 at 50.7 GPa. The β angle of naphthalene decreases from 116.08(7)° at ambient pressure to 103.3(3)° at 50.7 GPa. Pronounced anisotropic compressional behavior of naphthalene is due to its low symmetry: As seen in Figure 2, the *b* axis in naphthalene is much stiffer than the *a* and *c* axes. It is likely due to a shorter intermolecular distance in *b* direction and the smaller unit cell parameter *b* (ca. 6.00 Å) compared to parameters *a* (ca. 8.14 Å) and *c* (ca. 8.29 Å), which are quite similar.

**Figure 2:**
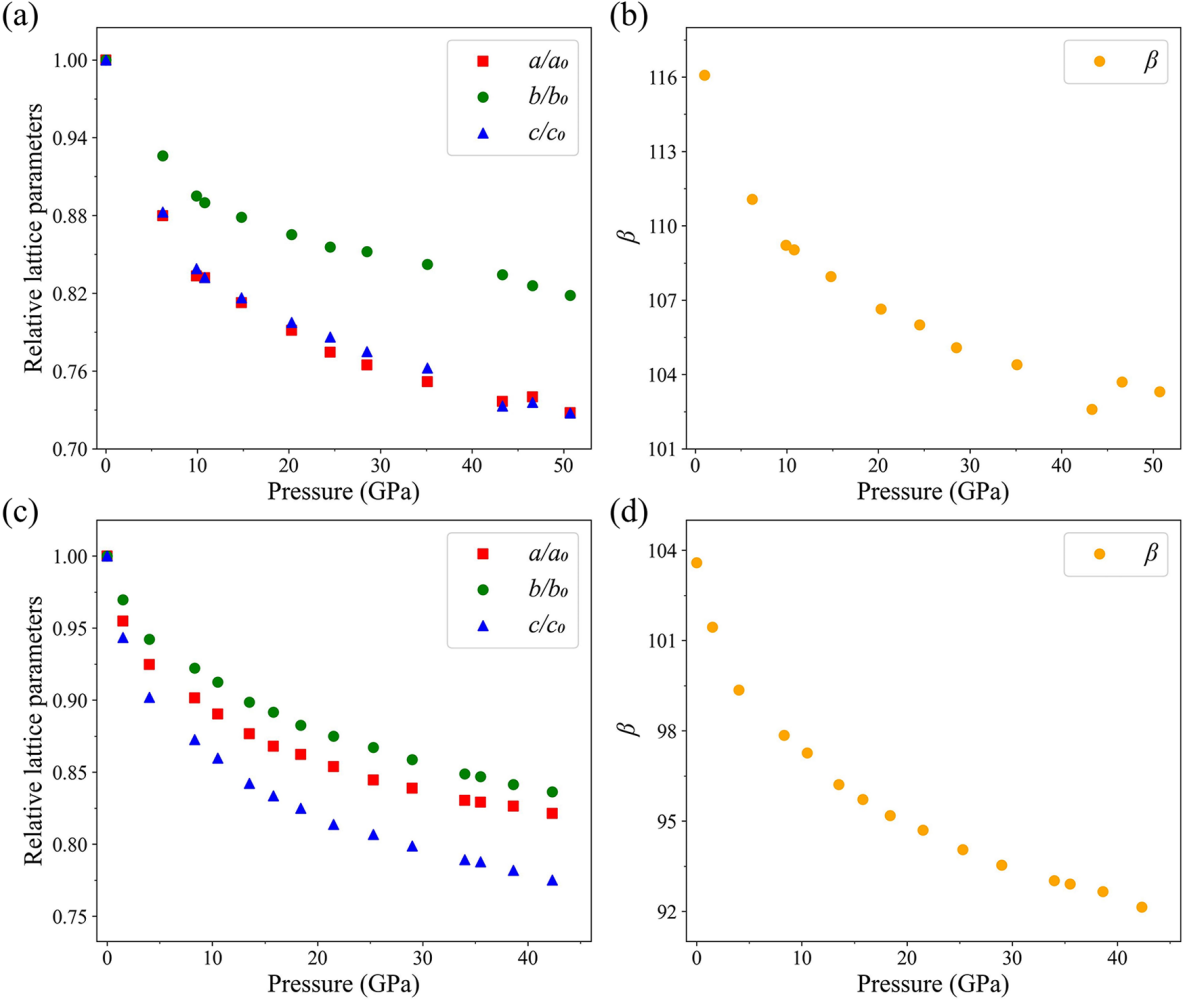
Pressure dependence of the relative lattice parameters (*a*/*a*_0_, *b*/*b*_0,_ and *c*/*c*_0_) and parameter β of naphthalene and anthracene. (a), (b) naphthalene up to 50.7 GPa; (c), (d) anthracene up to 42.3 GPa. *a*_0,_ *b*_0_, and *c*_0_ are the lattice parameters at ambient conditions.

The values of the unit cell volume per formula unit (V/Z) for naphthalene as a function of pressure, obtained from our experiments (Table S5), are shown in Figure 3a. These pressure–volume data were fitted using the third-order Birch–Murnaghan equation of state with the fixed unit cell volume per formula unit *V*_0_ = 182.2(2) Å^3^, which is the unit cell volume of naphthalene at ambient conditions determined in our studies. The bulk modulus, *K*_0_, and its first derivative, *K*′, were determined to be 8.4(10) GPa and 5.5(6), respectively.

**Figure 3:**
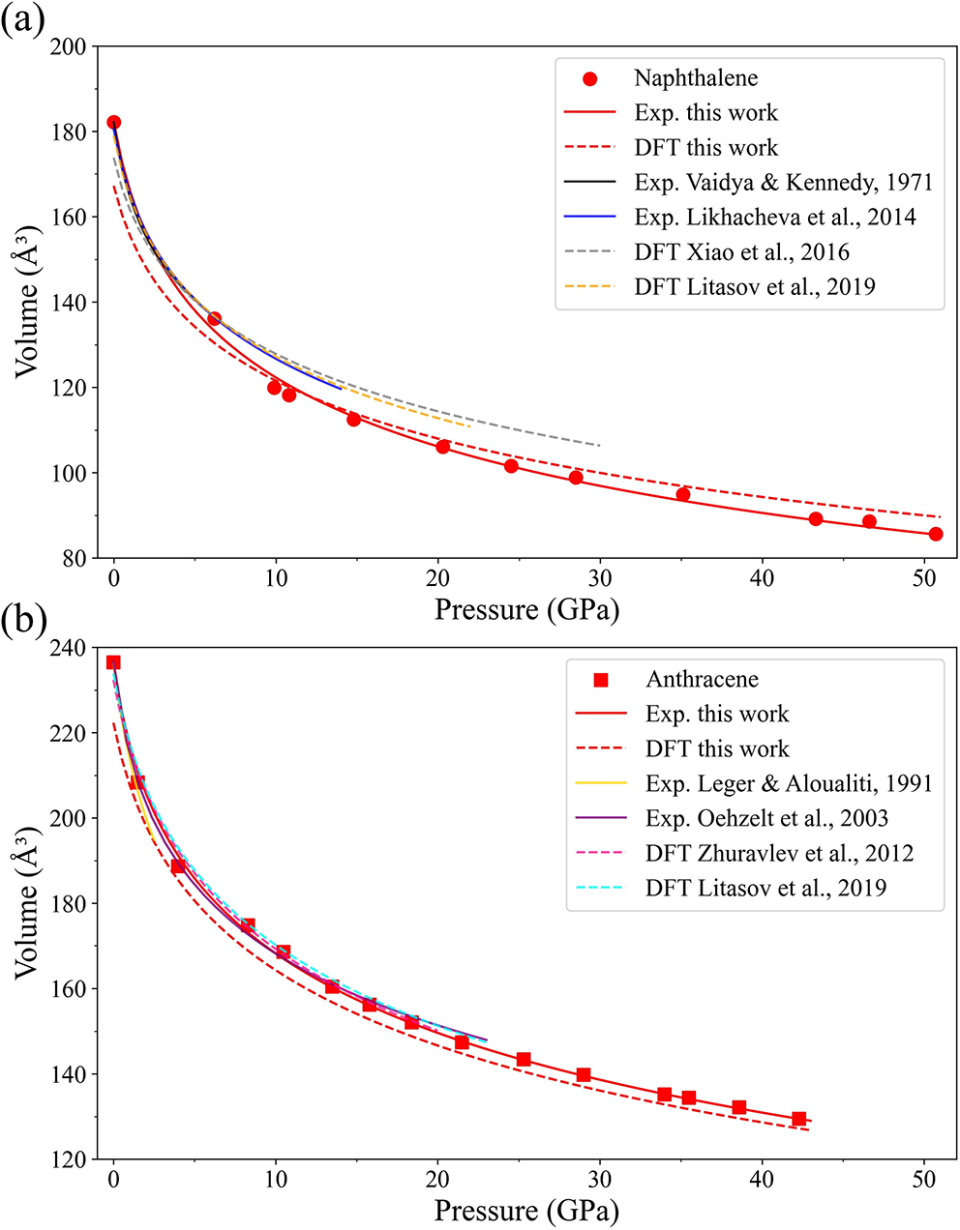
Compressional behavior of naphthalene and anthracene. (a) Naphthalene and (b) anthracene. Experimental points and their fit using the third order Birch–Murnaghan equation of state from this work are given by red symbols and red solid lines, correspondingly. Solid lines and dashed lines of other colors (see color code in the inset) represent experimental and theoretical literature data, correspondingly. The following literature data are referred to in this figure: Vaidya and Kennedy (1971),^8^ Likhacheva et al.,^12^ Xiao et al.,^46^ Litasov et al.,^47^ Leger and Aloualiti et al.,^18^ Oehzelt et al.,^19^ and Zhuravlev et al.^48^

The pressure–volume data calculated using DFT for naphthalene are presented in Table S6. The crystal structure at ambient pressure was obtained by performing relaxation of structural parameters in *P*1 space group without any restrictions. The fixed lattice volumes obtained from experiments were used to calculate corresponding pressure values. These calculated pressure–volume data were fitted using the third-order Birch–Murnaghan equation of state. The equation of state (EOS) parameters appeared to be as follows: *V*_0_ = 167.2 Å^3^, *K*_0_ = 10.8(2) GPa, and *K*′ = 6.99(13). Calculated *V*_0_ turns to be lower than the experimental value, but similar difference was found for benzo­[*a*]­pyrene (B*a*P) in our previous work.^30^

A comparison of our experimental and theoretical compressibility data for naphthalene with those from the literature is provided in Figure 3a and in (Tables S7 and S8). As seen, all experimental data, including ours and those of Likhacheva et al.,^12^ Vaidya and Kennedy,^8^ and Fabbiani et al.,^14^ agree well up to about 6 GPa (see also scatter plots in Figure S1). However, above 6 GPa, the experimental *P*–*V* points from Likhacheva et al.,^12^ available up to about 13 GPa, deviate from ours, and the difference in the volume at 13 GPa is of about 4%. This can be explained by different experimental conditions, such as the absence of pressure-transmitting media in experiments of Likhacheva et al.^12^ resulting in nonhydrostatic stresses. Additionally, the refinement in Likhacheva et al. was based on PXRD data.^12^ There is also a discrepancy between our calculations and those reported in the literature^46,47^ that can arise from the difference in computational approaches (we used a different dispersion correction method compared to previous studies; see Tables S8 and S9 for more details). Specifically, we adopted the DFT-D3 method, which has been demonstrated to be suitable for structural predictions of molecular crystals under high-pressure conditions.^49^ Our theoretical data reasonably agree with our experimental results.

### Geometrical Analysis of the Structure of Naphthalene under Compression

Figure 4a shows the structure of naphthalene as viewed along the [504] direction, chosen for an optimal projection. The molecules were approximated by mean molecular planes (red lines in Figure 4a) considering 10 carbon atoms in a molecule. The interplanar angles (δ) are indicated. These angles are listed in Table S10 and presented graphically in Figure 4b as a function of pressure. The intermolecular angle of naphthalene shows an overall decrease with increasing pressure from 53.5° at ambient pressure to 40.4° at 35.1 GPa. Some scattering above 35.1 GPa is likely due to deterioration of the quality of the single-crystal and decrease in reliability of the determined atomic positions.

**Figure 4:**
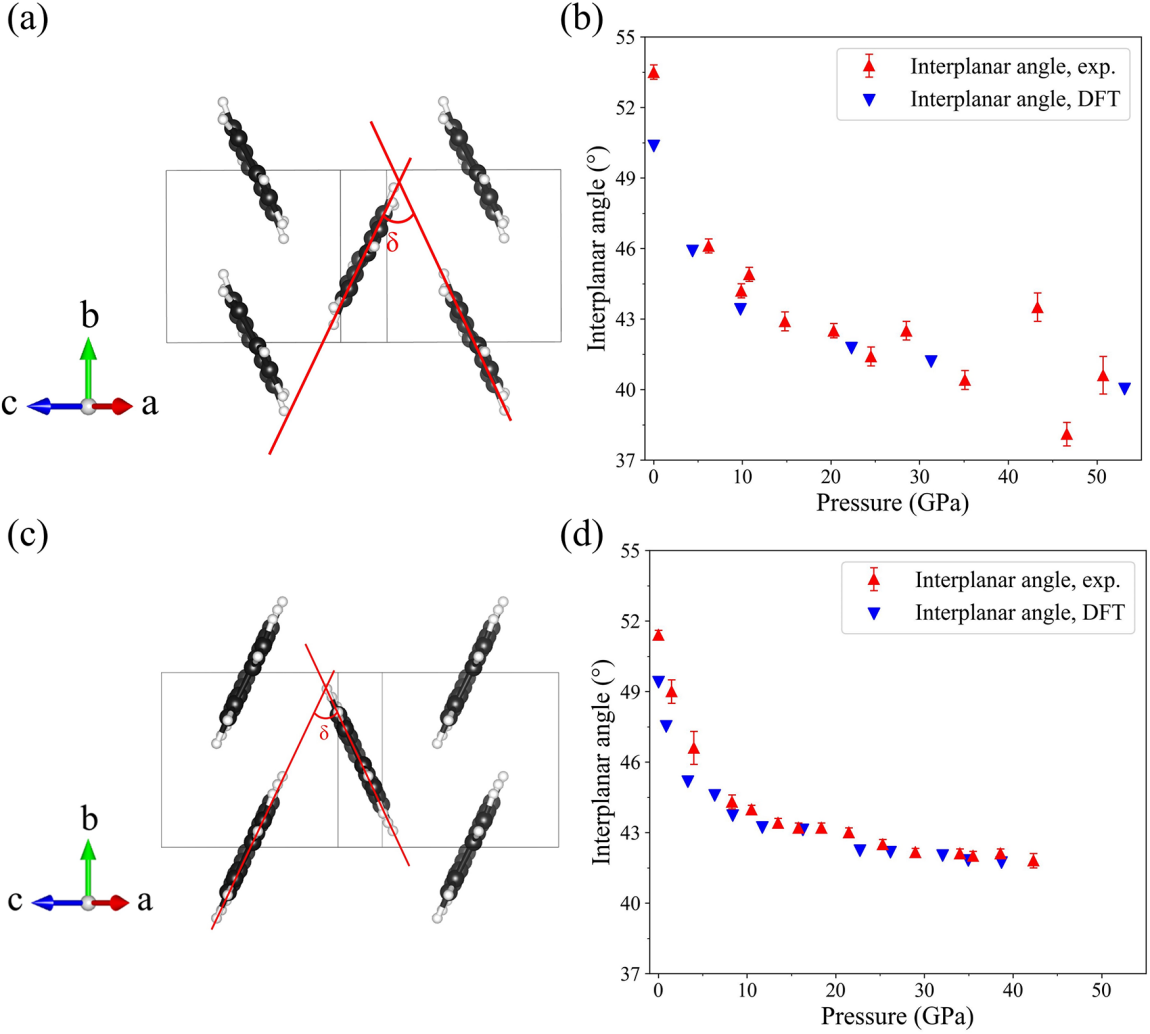
Interplanar angles for molecules in the structures of naphthalene and anthracene as viewed along the [504] direction. (a), (b) Naphthalene; (c), (d) anthracene. C atoms are black, H atoms are white; δ is the interplanar angle.

### Structure of Anthracene

The structure of anthracene (Figure 1c) is monoclinic (space group *P*2_1_/*c*, #14) with the following unit cell parameters at ambient conditions: *a* = 9.488(5) Å, *b* = 6.0253(3) Å, *c* = 8.5642(14) Å, β = 103.52(3)°, and *V* = 476.0(3) Å^3^ (*Z* = 2). These parameters are similar to those previously reported for perdeuteroanthracene (C_14_D_10_), as determined by single crystal neutron diffraction at 293 K^5^, see Table S11. Like in naphthalene, crystallographically equivalent anthracene molecules exhibit a herringbone packing motif in projection along the [100] direction.

### Compressional Behavior of Anthracene

No phase transitions were observed in anthracene up to 42.3 GPa, and diffraction was lost at the next pressure point of 45 GPa. Full crystallographic and experimental data are provided in Table S12. Variation of lattice parameters of anthracene is like that of naphthalene (Figure 2c and Table S4 for numerical values) showing a decrease with pressure, but the degree of anisotropy in similar directions is a bit different. For example, the *a* axis in anthracene is stiffer than in naphthalene that is explained by the size of the molecules (three fused benzene rings in anthracene vs two in naphthalene).

Figure 3b illustrates the pressure dependence of the unit cell volume per formula unit up to 42.3 GPa. These data were fitted with the fixed *V*_0_ = 238.0(2)­Å^3^, which is the unit cell volume per formula unit of anthracene at ambient conditions. The bulk modulus, *K*_0_, and its first derivative, *K*′, were determined to be 8.4(5) GPa and 8.0(4). The pressure–volume data calculated for anthracene using DFT (Table S6) and fitted using the Birch–Murnaghan EOS, gave the following parameters: *V*_0_ = 222.3 Å^3^, *K*_0_ = 11.57(13) GPa, and *K*′ = 7.34(8), in agreement with those obtained from the experimental data. However, the volume values from the calculated data fitting are consistently lower than those from the experimental data fitting. At least partially, this can be attributed to the fact that DFT calculations simulate the structures at 0 K while experiments are performed at 300 K.

Both our theoretical and experimental results agree well with the literature data (Figure 3b). Detailed information for the comparison with the experimental data from Oehzelt et al.^19^ and the theoretical data from Zhuravlev et al.^48^ and Litasov et al.^47^ can be found in Tables S13 and S14. The systematic discrepancy in theoretical data arises from the use of a different dispersion correction method compared to previous studies.

### Geometrical Analysis of the Structure of Anthracene under Compression

Figure 4c illustrates the structures of anthracene viewed along the [504] direction. The interplanar angles (δ) in anthracene were calculated using the same method as applied to naphthalene, approximating the molecules by mean molecular planes considering 14 carbon atoms. They are listed in Table S10 and presented graphically in Figure 4d as a function of pressure. The intermolecular angle of anthracene shows an overall decrease with increasing pressure from 51.4° at ambient pressure to 41.8° at 42.3 GPa.

### Hirshfeld Atom Refinement (HAR) for Naphthalene and Anthracene

Traditionally, crystal structure refinements have relied on the independent atom model (IAM). In the IAM, the lack of asphericity significantly affects the description of the electron density around hydrogen atoms, which have only one valence electron. This electron density is often strongly shifted toward the atoms to which the hydrogens are bonded. The most significant consequence of this approach is the underestimation of the bond lengths formed by hydrogen atoms. In this work, all C–H bond lengths were constrained to 0.93 Å using the HFIX 43 instruction in OLEX2.^50^

Recent studies have shown that HAR can achieve C–H bond lengths within one standard uncertainty of those obtained from neutron diffraction measurements, demonstrating comparable precision.^51^ Additionally, using high-resolution, high-quality XRD data can further enhance the accuracy and precision of bond length determination, improving the overall quality of the refinement. With high-quality data, it is also possible to apply an anisotropic treatment of hydrogen atom thermal motions, although this approach may result in slightly lower accuracy compared to anisotropic displacement parameters (ADPs) derived from neutron diffraction or other structural methods.^51,52^ In 2021, Guńka et al. attempted HAR for the α-C_6_H_12_N_4_ (urotropine polymorph) using diffraction data collected under pressure (although quite low, below 0.5 GPa).^53^ The authors obtained the C–H bond lengths within one standard uncertainty of those determined from neutron diffraction experiment on urotropine single crystal carried out at similar pressure.^54^ This example demonstrates that despite the lower completeness of diffraction data obtained for crystals in a DAC compared to those from free crystals, HAR can still be performed, provided the data quality is sufficiently high. Subsequent studies on other molecular crystals under high pressure have also demonstrated the reliability of HAR in high-pressure crystallography.^55,56^

We attempted HAR for naphthalene and anthracene using diffraction data collected with He as PTM. The completeness of the data for the maximum attained 2θ value ranged from 20 to 35%. The low completeness was due to the low symmetry of naphthalene and anthracene, as well as the limitations of the opening angle (70°) of the DACs. Additionally, we aimed to check if the refined C–H bond lengths would agree with our DFT-calculated data.

Figure 5a shows the average C–H bond lengths plotted as a function of pressure for naphthalene, with the experimental data up to about 35 GPa (see Table S15 for numerical values) and DFT-calculated values up to about 53 GPa (see Table S16 for numerical values). At ambient pressure, the average C–H bond length determined using HAR (1.08(5) Å) is very close to the average C–D bond length 1.093(3) Å determined from neutron diffraction experiments on perdeuteronaphthalene single crystals at 12 K.^2^ At nonambient pressure, there is also a reasonable agreement between the experimental average C–H bond lengths and those obtained by DFT calculations. However, the uncertainties for the HAR values are up to ±0.06 Å that questions applicability of these data for analysis of trends in bond length at high pressures.

**Figure 5:**
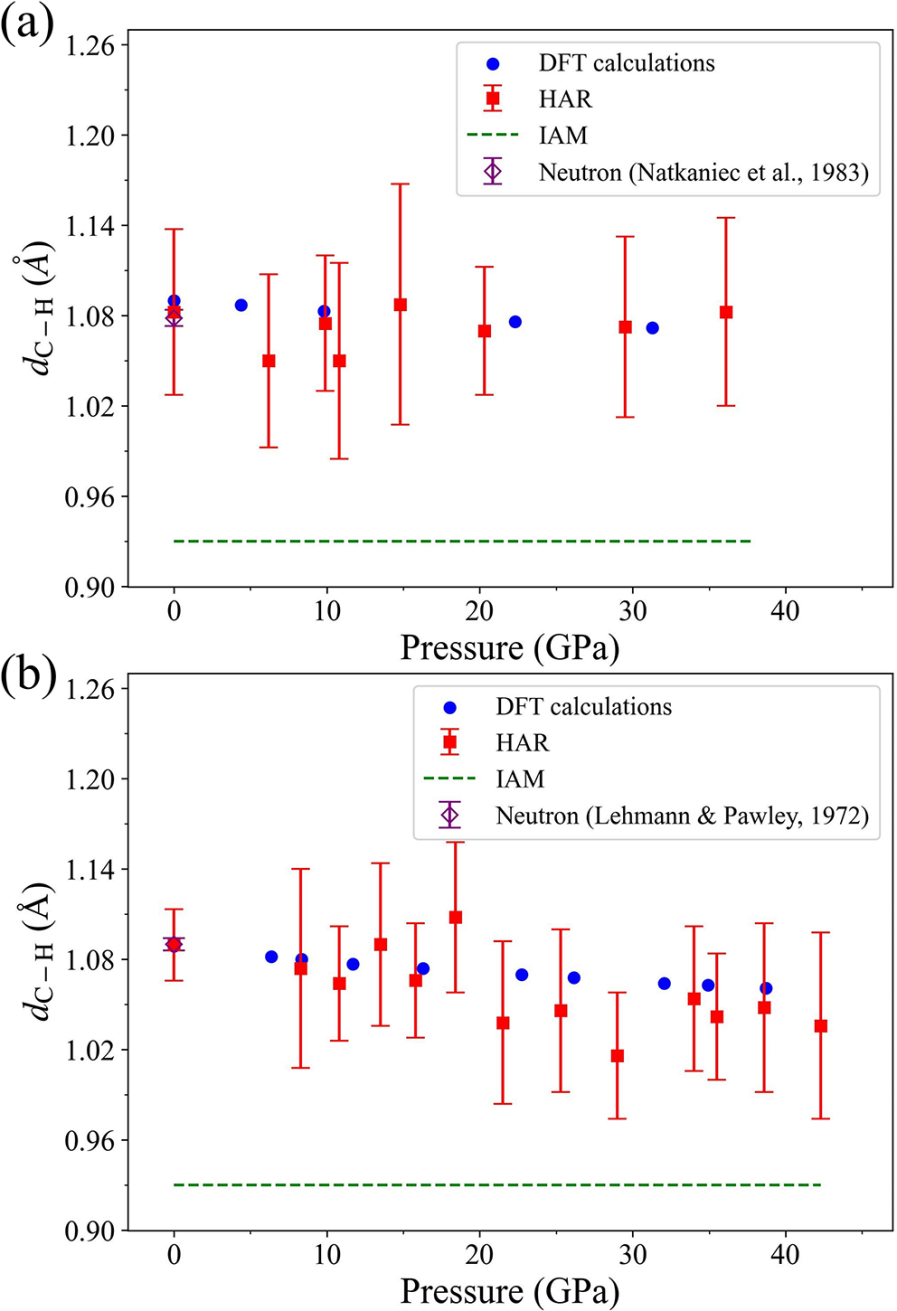
Average C–H bond lengths plotted as a function of pressure for naphthalene and anthracene molecules. (a) Naphthalene up to 35.1 GPa; (b) anthracene up to 42.3 GPa. Solid symbols of different colors represent the following: blue circles—DFT calculated values; red squares—the values obtained using Hirshfeld atom refinement (HAR); green dash—the values derived from the independent atom model (IAM), and a purple open diamond—the value acquired from neutron diffraction.^2,5^

The same problem appears with anthracene. Figure 5b shows the average C–H bond lengths plotted as a function of pressure for anthracene, with the experimental data up to about 42 GPa (see Table S15 for numerical values) and calculations up to about 39 GPa (see Table S16 for numerical values). The HAR value of the average C–H bond length of 1.09(2) Å at ambient pressure is within one standard uncertainty from the average C–D bond length of 1.104(2) Å, which was determined from neutron diffraction experiments on perdeuteroanthracene single crystals.^5^ The average error for the HAR value of the C–H bond length across all pressure points in the experimental data is ±0.047 Å that is quite high, similarly to that for naphthalene. Overall, the HAR data on the C–H bond lengths are in good agreement with the DFT results for both naphthalene and anthracene, but large uncertainties still preclude reliable application of HAR for detail analysis of high pressure data.

### Hirshfeld Surface Analysis for Naphthalene and Anthracene

To visualize intermolecular interactions and explore their evolution upon compression, we constructed Hirshfeld surfaces and generated fingerprint plots for naphthalene and anthracene at different pressures using CrystalExplorer.^57^ The molecular Hirshfeld surface defines the volume of space where the promolecule electron density exceeds that of all neighboring molecules, therefore it carries information about intermolecular interactions.^58^ The fingerprint plot provides a 2D representation of two key distance metrics: the distances from internal and external atoms (*d*_i_ and *d*_e_) to the Hirshfeld surface. As a result, these plots are highly responsive to the molecule’s immediate surroundings. Each fingerprint plot is distinct for a specific molecule in each polymorphic form. For details on the functions of distance and curvature (shape index) mapped on Hirshfeld surfaces, see Spackman and Jayatilaka.^58^

Figures 6 and 7 present the Hirshfeld surfaces mapped with the shape index and the *d*_e_ for naphthalene (Figure 6) and anthracene (Figure 7) at ambient conditions and high pressures (35.1 GPa for naphthalene and 42.3 GPa for anthracene), as well as the corresponding fingerprint plots. The front and back views of Hirshfeld surfaces are identical because the molecules reside on an inversion center.

**Figure 6:**
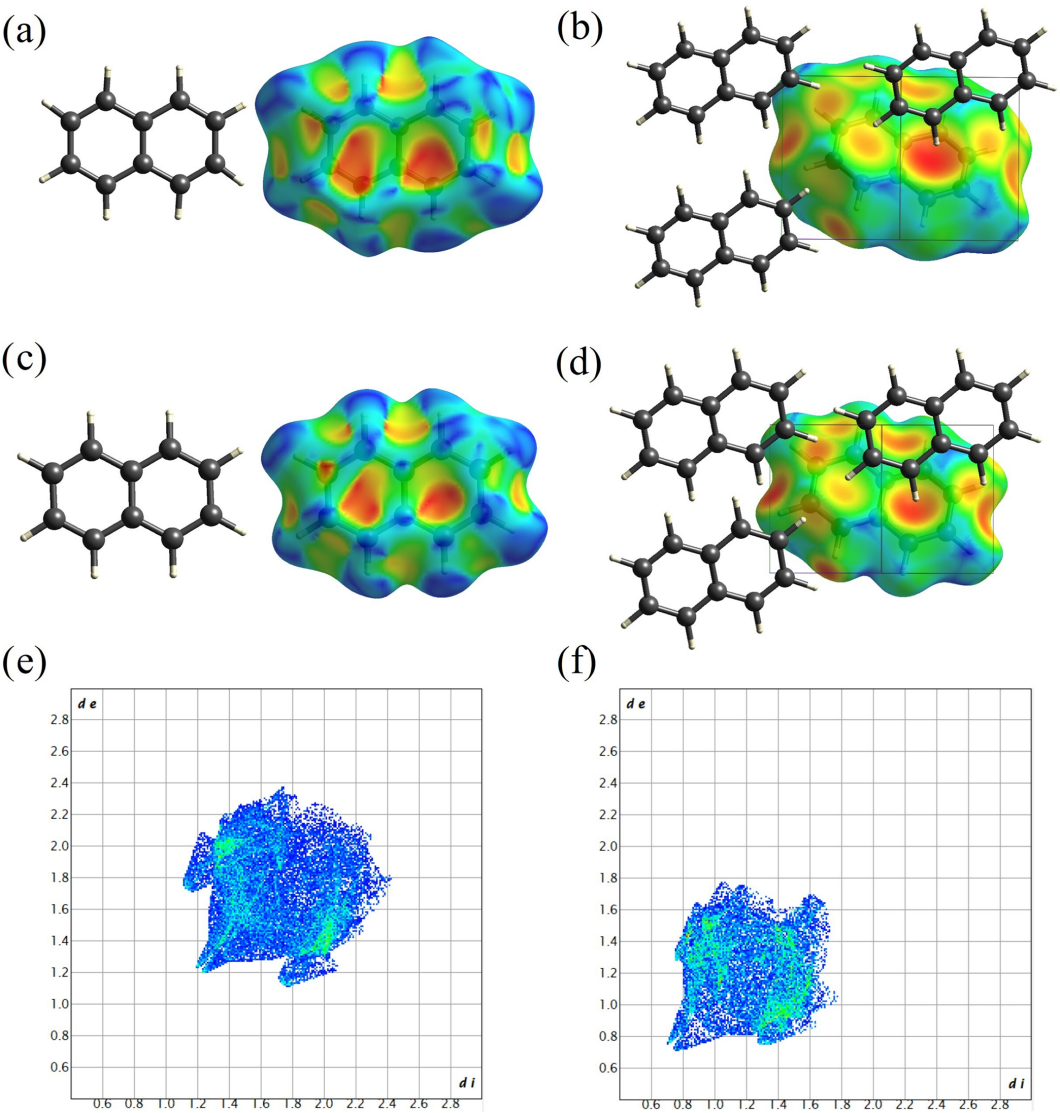
Hirshfeld surfaces and fingerprint plots of naphthalene at different pressures. Hirshfeld surfaces at ambient conditions mapped (a) with shape index and (b) with *d*_e_, as viewed along the [−101] direction. Shape index is mapped from −1.0 (red) to 0.0 (green) to 1.0 (blue); Hirshfeld surfaces at 35.1 GPa mapped with (c) shape index and (d) *d*_e_, as viewed along the [−101] direction. Shape index is mapped from −1.0 (red) to 0.0 (green) to 1.0 (blue); fingerprint plots: (e) at ambient conditions and (f) at 35.1 GPa. Single crystal XRD data used here were refined using IAM. The front and back views of Hirshfeld surfaces for naphthalene are identical.

**Figure 7:**
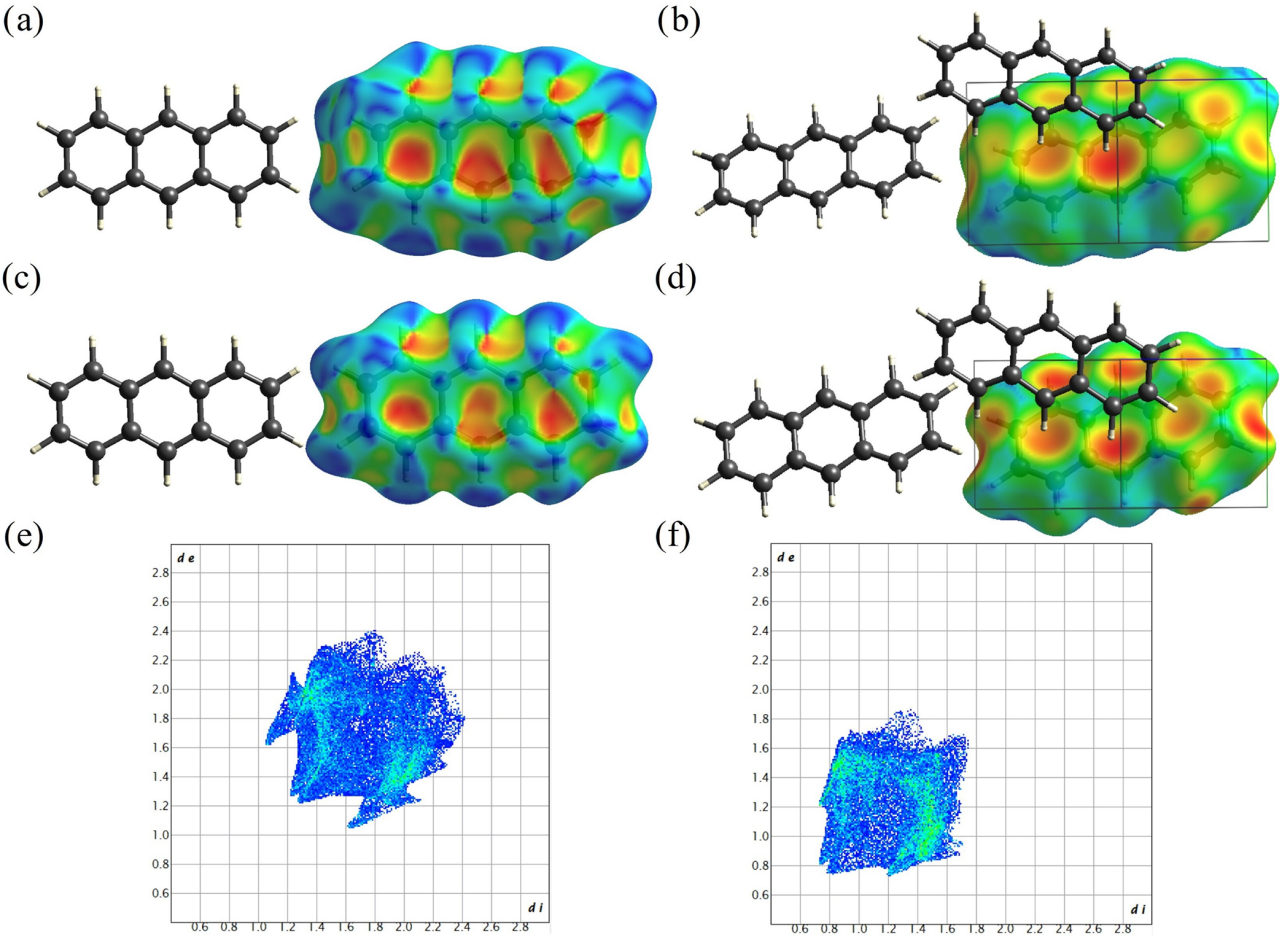
Hirshfeld surface and fingerprints plots of anthracene at different pressures. Hirshfeld surfaces at ambient condition mapped (a) with shape index and (b) with *d*_e_, as viewed along the [−101] direction. Shape index is mapped from −1.0 (red) to 0.0 (green) to 1.0 (blue); Hirshfeld surfaces at 42.3 GPa mapped with (c) shape index and (d) *d*_e_, as viewed along the [−101] direction. Shape index is mapped from −1.0 (red) to 0.0 (green) to 1.0 (blue); fingerprint plots: (e) at ambient conditions and (f) at 42.3 GPa. Single crystal XRD data used here were refined using IAM. The front and back views of Hirshfeld surfaces for anthracene are identical.

Both Hirshfeld surfaces and fingerprint plots for naphthalene and anthracene at ambient conditions have been previously described and discussed in detail by McKinnon et al., and our observations are fully consistent with the literature data.^59^ The intermolecular interactions between naphthalene molecules are dominated by C–H···π and H···H interactions. The C–H···π interactions are reflected by the two red regions of concave shape in the center part of the surface mapped with the shape index (Figure 6a). Corresponding C–H donor regions appear as blue bumps at the edge of the surface (see bottom of the surface, to the right; Figure 6a). In Figure 6b the surface mapped with *d*_e_ is displayed in the crystal-packing diagram showing three more surrounding molecules. The darkest red region indicates the main C–H···π interactions that is reflected in the fingerprint plot as a pair of wings (Figure 6e), with the shortest C–H···π contact (*d*_C···H_ ≈ 2.81 Å) at (*d*_i_, *d*_e_) ∼1.7 Å, 1.1 Å (and *vice versa*). Weaker C–H···π interactions (Figure 6b) (reddish-yellow area to the left of the red one in Figure 6b) reflected as the second pair of wings in Figure 6e. The head-to-head H···H interactions are indicated by the two red regions (at “7 and 9 o’clock”) on the left side of the surface mapped with *d*_e_ in Figure 6b and are reflected in the fingerprint plot (Figure 6e) as a double tip in the lower left corner of the fingerprint plot at (*d*_i_, *d*_e_) ∼1.2 Å.

The character of intermolecular interactions does not change much with pressure, as seen from the Hirshfeld surfaces for naphthalene molecules at 35.1 GPa (Figure 6c,d). However, the size of the area in the fingerprint plot (Figure 6f) reduces considerably, indicating high compaction and considerable decrease of interatomic distances. The wings on both sides are not much spread anymore. The shortest C–H···π contact at 35.1 GPa is (*d*_C···H_ ≈ 2.05 Å) vs *d*_C···H_ ≈ 2.81 Å at ambient conditions. The head-to-head H···H interactions shorten to approximately (*d*_i_, *d*_e_) ∼0.75 Å vs (*d*_i_, *d*_e_) ∼1.2 Å at ambient, suggesting a high density of molecular packing.

The observations for anthracene are quite similar (Figure 7). The intermolecular interactions C–H···π and H···H are also dominant. As an anthracene molecule contains one more fused benzene ring compared to naphthalene, there are two major C–H···π interactions (seen red at the center of the Hirschfeld surface mapped with *d*_e_, (Figure 7b). The color intensity, changing from dark to light red and yellow, indicates the weakening of interactions as the interatomic distances increase. Among these, the darker red region represents the strongest C–H···π interaction with the shortest C–H···π contact *d*_C···H_ ≈ 2.70 Å) at ambient conditions, reflected in the fingerprint plot (Figure 7e) at approximately (*d*_i_, *d*_e_) ∼ (1.6, 1.1 Å). Weaker interactions are observed at (*d*_i_, *d*_e_) ∼ (2.1, 1.2 Å) and ∼ (2.3, 1.5 Å), respectively. The head-to-head H···H interactions are indicated by the two red regions on the left side of the Hirschfeld surface mapped with *d*_e_ (at “8 and 9 o’clock” at the very left edge of the surface in Figure 7b) and are reflected in the fingerprint plot (Figure 7e) as a tip in the lower left corner at (*d*_i_, *d*_e_) ∼1.3, 1.2 Å. The head-to-head H···H interactions occur between the anthracene molecule inside the Hirshfeld surface and one adjacent anthracene molecule (shown to the left in Figure 7b). In contrast, in naphthalene, the head-to-head H···H interactions involve the molecule inside the Hirshfeld surface and two neighboring molecules (Figure 6b).

At 42.3 GPa, the character of the interactions does not change much, and three distinct red and light red regions representing different C–H···π interactions are well seen in Figure 7d. Corresponding wings in fingerprint plot (Figure 7f) merge, and the shortest C–H···π contact decreases to the value (*d*_C···H_ ≈ 1.9 Å vs *d*_C···H_ ≈ 2.70 Å) at ambient conditions. Additionally, the shortest H···H interactions are observed at approximately (*d*_i_, *d*_e_) ∼0.8 Å indicating the decrease of H···H contact from ∼2.4 Å at ambient conditions down to ∼1.6 Å in the much denser molecular packing under high pressure.

One can break down the Hirshfeld surface into patches associated with specific atom-type/atom-type pairs, to highlight just those regions on the surface, and sum the areas of surface patches associated with various contacts.^58^ We have made the calculations using the CrystalExplorer program^57^ for naphthalene and anthracene as a function of pressure (Table S17). Figure 8a,b presents percentage contributions to the Hirschfeld surface area for the various close intermolecular contacts (H···H, C···H and C···C) as a function of pressure for molecules in naphthalene and anthracene. For naphthalene at ambient conditions, H···H interactions are associated with nearly 55% of the surface area, and C···H interactions- with the rest of ca. 45% of the surface area, so that the contribution of C···C interactions is negligible. Upon compression, the percentage contribution of H···H interactions monotonically decreases down to about 41%, while that of C···H interactions monotonically increases up to ca. 53%, giving a contribution of the C···C interactions to increase to about 6% at 43.3 GPa (Figure 8a). Similar trend is observed for anthracene: at ambient conditions, H···H interactions are associated with 49% of the surface area, the C···H - of about 50%, whereas the contribution of C···C interactions is of about 1% (Figure 8b). The percentage of C···H interactions is slightly higher for anthracene compared to naphthalene (50% vs 45%, respectively), due to the molecule of anthracene contains one more benzene ring compared with the molecule of naphthalene that makes additional contribution to the overall C–H···π interactions in anthracene, and provides some space for C···C interactions, although very small. As pressure increases, the percentage of H···H interactions decreases down to ca. 36%, while that of C···H interactions grows smoothly up to ca. 59% at 42.3 GPa, so that the percentage of C···C interactions does not increase beyond approximately 5%.

**Figure 8:**
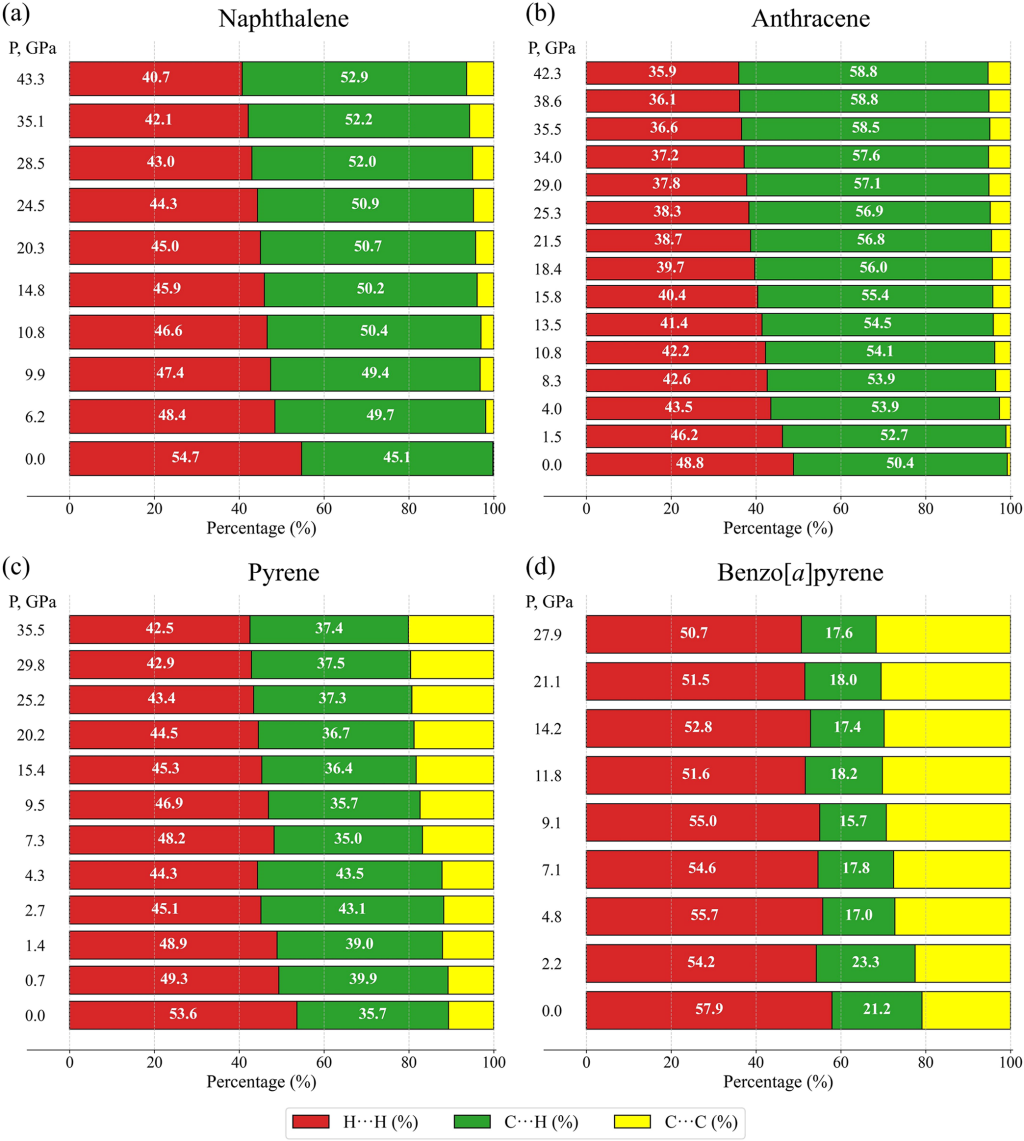
Percentage contribution to the Hirschfeld surface area for the various close intermolecular contacts (H···H, C···H and C···C) as a function of pressure for molecules in (a) naphthalene, (b) anthracene, (c) pyrene and (d) benzo­[*a*]­pyrene.

Our results, based on experimental structural data, are in a very good agreement with those of DFT calculations of Hirshfeld surface intermolecular close-contact fractions as a function of pressure up to 20 GPa for naphthalene and anthracene.^60^ According to ref 50, “the C···C and C···H interactions increase with increasing pressure, whereas the H···H interactions decrease”. For example, “for naphthalene, there is a ∼4% increase in % C···C from 0 to 20 GPa, a C···H···∼4% increase in % C···H, and an ∼8% decrease in % H···H”,^50^ fully in line with our data.

It appears that the herringbone packing motif of the molecules, the limited contribution of stronger C···C interactions to intermolecular bonding, and the high flexibility of relatively weak H···H interactions—which significantly contribute to the Hirshfeld surface area in both naphthalene and anthracene—facilitate a gradual compaction of their structures without phase transitions. This is achieved through a smooth decrease in the intermolecular angle.

Like naphthalene and anthracene, both pyrene and B*a*P, next members of the PAH series, exhibit a herringbone motif in molecular packing, but additionally, in pyrene two molecules are related by the center of inversion forming “sandwiches”, so that their packing is called “sandwich-herringbone”. Contrary to naphthalene and anthracene, both pyrene and B*a*P, undergo a few phase transitions below about 7 GPa: pyrene-I → pyrene-II (0.7 GPa), pyrene-II → pyrene-IV (2.7 GPa), pyrene-IV→ pyrene-V (7.3 GPa);^51^ B*a*P–I → B*a*P–II (4.8 GPa), B*a*P–II→ B*a*P–III (7.2 GPa).^30^

For comparison, based on the structural data from our previous work,^29,30^ we calculated the percentage contributions of H···H, C···H, and C···C contacts to the Hirshfeld surface area as a function of pressure for pyrene and B*a*P studied up to ca. 36 and 28 GPa, respectively (numerical data are provided in Table S17). The corresponding diagrams are presented in Figure 8c,d. As seen, the C···C contributions in both pyrene (∼10%) and BaP (∼20%) at ambient pressures are considerably higher than those in naphthalene and anthracene, likely providing less flexibility for a smooth compaction, especialy at low pressures. To accumulate applied stress, intermolecular angles change abruptly causing the phase transitions that reflects in the irregular change of interactions contributions below ca. 7 GPa. Above 7 GPa, there is a noticeable increase of contributions of C···C interactions up to 20% in pyrene-V and ca. 30% in B*a*P–III, which remain structurally stable to the highest pressures achieved in the experiments.^29,30^ Such a stability can be explained by strength of C···C interactions acting on both sides of the molecules in pyrene-V, not featuring sandwich arrangements anymore, and on both sides of the molecules in B*a*P–III in their planar stacking.

## Conclusions

In this work, we experimentally studied compressional behavior of naphthalene and anthracene up to about 50 and 43 GPa, respectively, using SC-XRD. At each pressure point their crystal structures were refined. Our experimental and complementing computational research contributes to understanding of compressional mechanisms, bonding evolution, and structural stability of PAHs under nonambient conditions.

To summarize, our crystallographic study of naphthalene and anthracene under pressure reveals that these PAHs exhibit structural similarities, as well as comparable compressional behavior. With increasing pressure, the intermolecular angles decrease, resulting in significantly denser molecular packing. The unit cell volumes of both compounds decrease at similar rates, reaching approximately half their ambient values within the studied pressure ranges. Specifically, the unit cell volume of naphthalene decreases from 364.3(4) Å^3^ at ambient conditions to 171.2(8) Å^3^ at 50.7 GPa, while that of anthracene decreases from 476.0(3) Å^3^ at ambient pressure to 258.8(6) Å^3^ at 42.3 GPa. The analysis of Hirshfeld surfaces and fingerprint plots for naphthalene and anthracene at ambient and high pressures has shown that the character of intermolecular interactions in both compounds does not change much with pressure. Our experimental and computational investigations demonstrate that both compounds exhibit remarkable structural stability, as no phase transitions were observed within the studied pressure ranges.

## Supplementary Material

































































## Notes

Comparison of naphthalene compressibility data obtained experimentally in our study with those reported in the literature; summary of the experiments conducted in this work; experimental crystallographic data for naphthalene at ambient conditions obtained by single crystal X-ray diffraction in this work; experimental crystallographic data for anthracene obtained by single-crystal X-ray diffraction at room temperature; lattice parameters for naphthalene up to 50.7 GPa and anthracene up to 42.3GP; summary of available HP XRD experimental data for naphthalene (PDF)
Naphthalene_00.0GPa_IAM (CIF)
Naphthalene_06.2GPa_IAM (CIF)
Naphthalene_09.9GPa_IAM (CIF)
Naphthalene_10.8GPa_IAM (CIF)
Naphthalene_14.8GPa_IAM (CIF)
Naphthalene_20.3GPa_IAM (CIF)
Naphthalene_24.5GPa_IAM (CIF)
Naphthalene_28.5GPa_IAM (CIF)
Naphthalene_35.1GPa_IAM (CIF)
Naphthalene_43.3GPa_IAM (CIF)
Naphthalene_46.6GPa_IAM (CIF)
Naphthalene_50.7GPa_IAM (CIF)
Naphthalene_IAM_Merged (CIF)
Naphthalene_IAM_Merged_CheckCIF report (PDF)
Anthracene_00.0GPa_IAM (CIF)
Anthracene_01.5GPa_IAM (CIF)
Anthracene_04.0GPa_IAM (CIF)
Anthracene_08.3GPa_IAM (CIF)
Anthracene_10.8GPa_IAM (CIF)
Anthracene_13.5GPa_IAM (CIF)
Anthracene_15.8GPa_IAM (CIF)
Anthracene_18.4GPa_IAM (CIF)
Anthracene_21.5GPa_IAM (CIF)
Anthracene_25.3GPa_IAM (CIF)
Anthracene_29.0GPa_IAM (CIF)
Anthracene_34.0GPa_IAM (CIF)
Anthracene_35.5GPa_IAM (CIF)
Anthracene_38.6GPa_IAM (CIF)
Anthracene_42.3GPa_IAM (CIF)
Anthracene_IAM_Merged (CIF)
Anthracene_IAM_Merged_CheckCIF report (PDF)

## #

Center for High Pressure Science and Technology Advanced Research, Beijing 100193, China

## Abbreviations

SC-XRD: single-crystal X-ray diffraction
PAHs: polycyclic aromatic hydrocarbons
PXRD: powder X-ray diffraction
DACs: diamond anvil cells
PTM: pressure-transmitting medium
HAR: Hirshfeld atom refinement
DFT: density functional theory
VASP: Vienna ab initio simulation package
PAW: projector-augmented-wave
GGA: generalized gradient approximation
PBE: Perdew–Burke–Ernzerhof
EOS: equation of state
B*a*P: benzo­[*a*]­pyrene
IAM: independent atom model
ADPs: anisotropic displacement parameters
