# Supplementary material for: Compressional Behavior of Naphthalene (C10H8) and Anthracene (C14H10) up to 50 GPa
Source: ACS Omega. 2025 Oct 19;10(42):50230–42. doi: 10.1021/acsomega.5c06935 (PMC12573047; doi:10.1021/acsomega.5c06935)
Supplement: Supplementary file 1 [file ao5c06935_si_001.pdf]

Supporting Information

Compressional Behavior of Naphthalene ($C_{10}H_8$) and Anthracene ($C_{14}H_{10}$) up to 50 GPa

Wenju Zhou[a,f]*, Xiang Li[c,e], Fariia Iasmin Akbar[a,b], Anna Pakhomova[c], Michael Hanfland[c], Leonid Dubrovinsky[b], Natalia Dubrovinskaia[a,d]*

[a]Material Physics and Technology at Extreme Conditions, Laboratory of Crystallography, University of Bayreuth, 95440 Bayreuth, Germany

[b]Bayerisches Geoinstitut, University of Bayreuth, 95440 Bayreuth, Germany

[c]European Synchrotron Radiation Facility, CS 40220, 38043 Grenoble Cedex 9, France

[d]Department of Physics, Chemistry and Biology (IFM), Linköping University, SE-581 83, Linköping, Sweden

[e]Institut für Mineralogie, University of Münster, Corrensstr. 24, 48149 Münster, Germany.

[f]Current address: Center for High Pressure Science and Technology Advanced Research, Beijing 100193, China

*Correspondence E-mails: Wenju.Zhou@uni-bayreuth.de, Natalia.Dubrovinskaia@uni-bayreuth.de

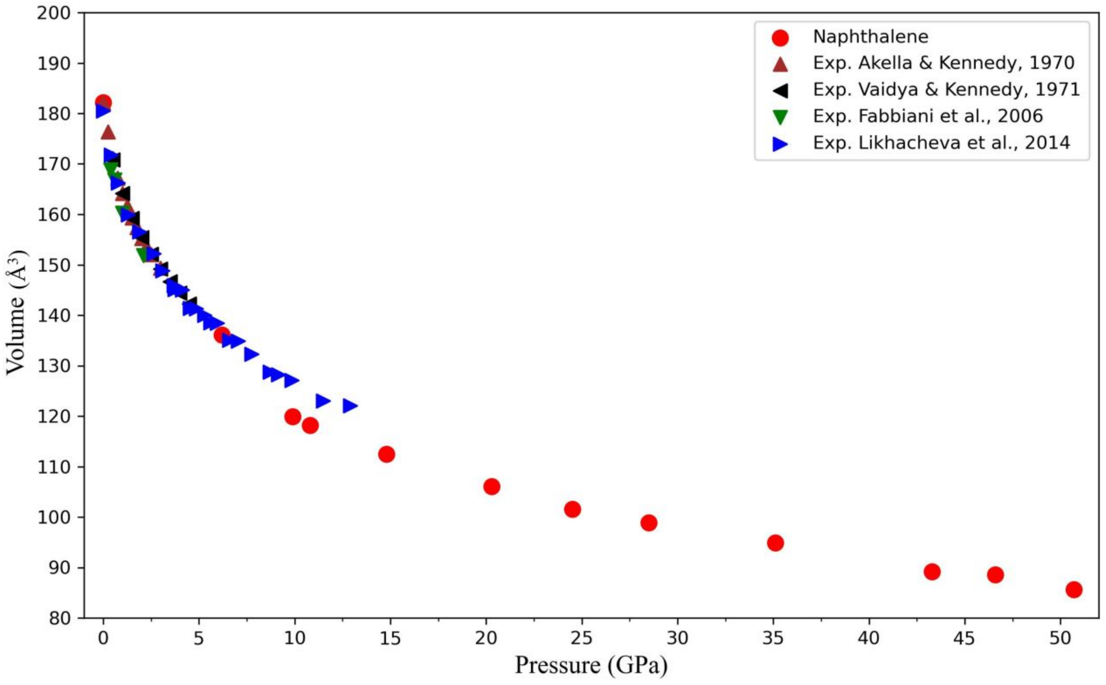

Figure S1. Comparison of naphthalene compressibility data obtained experimentally in our study with those reported in the literature. Red solid circles represent the experimental data from our study. Brown triangles represent experimental data from Akella and Kennedy[1]. Black left triangles represent experimental data from Vaidya and Kennedy[2]. Green invert triangles represent experimental data from Fabbiani *et al.*[3]. Blue right triangles represent experimental data from Likhacheva *et al.*[4].

Table S1. Summary of the experiments conducted in this work.

DAC number	DAC type	Anvils type/ culet size, µm	Starting material/ pressure transmitting medium	Beamline/XRD wavelength, Å	Result	Pressure, GPa
1	Membrane-type	Boehler-Almax 250	Naphthalene/ No medium	ID27 ESRF, 0.3738	Naphthalene	ambient
2	Membrane-type	Boehler-Almax 250	Naphthalene/ Helium	ID27 ESRF, 0.3738	Naphthalene	6.2, 9.9, 10.8, 14.8 20.3, 24.5, 28.5, 35.1, 43.3, 46.6, 50.7
3	Membrane-type	Boehler-Almax 250	Anthracene/ No medium	ID15B ESRF, 0.4100	Anthracene	ambient
4	Membrane-type	Boehler-Almax 250	Anthracene/ Helium	ID15B ESRF, 0.4100	Anthracene	1.5, 4.0, 8.3, 10.8, 13.5, 15.8, 18.4, 21.5, 25.3, 29.0, 34.0, 35.5, 38.6, 42.3

Table S2. Experimental crystallographic data for naphthalene at ambient conditions obtained by single crystal X-ray diffraction in this work and in ref. (5), obtained by neutron diffraction in ref. (6), and perdeuteronaphthalene obtained by neutron diffraction in ref. (7).

	Naphthalene at ambient condition	Naphthalene at 205 K[5]
CCDC deposition number	2407670	233931
Crystal data		
Chemical formula	$C_{10}H_8$	$C_{10}H_8$
M_r	128.175	128.175
Crystal system, space group	Monoclinic, $P2_1/c$	Monoclinic, $P2_1/c$
a, b, c (Å)	8.147(6), 6.0035(8), 8.293(3)	7.9435(1), 5.9534(1), 8.1645(1)
α, β, γ (°)	90, 116.08(7), 90	90, 114.998(1), 90
V (Å^3)	364.3(4)	349.93(1)
Z	2	2
Density (Mg/m^3)	1.168	1.217
Wavelength (Å)	0.3738	0.7107 (Mo Kα)
μ (mm^{-1})	0.029	0.070
Data collection		
Absorption correction	Multi-scan	Multi-scan
T_{min}, T_{max}	1.00, 0.37	0.970, 0.979
No. of measured, independent and observed reflections	888, 589, 190	63300, 4250
R_{int}	0.093	0.0321
θ_{max} (°)	20.84	27.5
Refinement		
Refinement on	F^2	F^2
$R[F^2>2\sigma(F^2)]$,$wR(F^2)$, S	0.081, 0.184, 0.858	0.037, 0.036, 0.952
Data / restraints / parameters	589/ 0/ 46	4250/ 0
H-atom treatment	Refined by ride model	
Weighting scheme	$w=1/[\sigma^2(F_o^2)+(0.0403P)^2]$ where $P=(F_o^2+2Fc^2)/3$	$w = 1/[\sigma^2 (F_o^2)]$
$\Delta\rho_{max}$, $\Delta\rho_{min}$ (e Å^{-3})	0.09, -0.09	

Table S2. (continuation)

	Naphthalene at 295 K[6]	Perdeuteronaphthalene at 12K[7]
CCDC deposition number	600187	1216820
Crystal data		
Chemical formula	$C_{10}H_8$	$C_{10}D_8$
M_r	128.175	136.212
Crystal system, space group	Monoclinic, $P2_1/a$	Monoclinic, $P2_1/c$
a, b, c (Å)	8.256(2), 5.983(2), 8.677(3)	7.795(11), 5.941(5), 8.096(7)
α, β, γ (°)	90, 122.729(7), 90	90,114.09(13),
V (Å^3)	360.6(3)	342.3(11)
Z	2	2
Density (Mg/m^3)	1.179	1.244
Wavelength (Å)	0.840 (Neutron)	1.26 (Neutron)
μ (mm^{-1})		0.948
Data collection		
Absorption correction		
T_{min}, T_{max}		
No. of measured, independent and observed reflections	600, 600, 324	1036, 907, 1036
R_{int}		0.01
θ_{max} (°)		
Refinement		
Refinement on	F^2	
R[F^2>2σ(F^2)],wR(F^2), S	0.028, 0.028	0.031, 0.031
Data / restraints / parameters	600/ 25	907/ 0
H-atom treatment		
Weighting scheme		
$\Delta\rho_{max}$, $\Delta\rho_{min}$ (e Å^{-3})		

Table S3. Experimental crystallographic data for naphthalene obtained by single-crystal X-ray diffraction at room temperature in this work.

	Naphthalene at 6.2 GPa	Naphthalene at 9.9 GPa
CCDC deposition number	2407678	2407642
Crystal data		
Chemical formula	$C_{10}H_8$	$C_{10}H_8$
M_r	128.175	128.175
Crystal system, space group	Monoclinic, $P2_1/c$	Monoclinic, $P2_1/c$
a, b, c (Å)	7.168(7), 5.5599(6), 7.320(3)	6.790(8), 5.3738(8), 6.959(3)
α, β, γ (°)	90, 111.07(8), 90	90, 109.22(9), 90
V (Å^3)	272.2(3)	239.8(3)
Z	2	2
Density (Mg/m^3)	1.564	1.775
Wavelength (Å)	0.3738	0.3738
μ (mm^{-1})	0.038	0.043
Data collection		
Absorption correction	Multi-scan	Multi-scan
T_{min}, T_{max}	1.00, 0.37	1.00, 0.23
No. of measured, independent and observed reflections	557, 308, 177	459, 290, 210
R_{int}	0.031	0.019
θ_{max} (°)	17.23	17.24
Refinement		
Refinement on	F^2	F^2
R[F^2>2σ(F^2)],wR(F^2), S	0.067, 0.166, 0.938	0.077, 0.193, 0.983
Data / restraints / parameters	308/ 0/ 46	290/ 6/ 46
H-atom treatment	Refined by ride model	Refined by ride model
Weighting scheme	$w=1/[\sigma^2(F_o^2)+(0.1347P)^2]$ where $P=(F_o^2+2F_c^2)/3$	$w=1/[\sigma^2(F_o^2)+(0.1717P)^2]$ where $P=(F_o^2+2F_c^2)/3$
$\Delta\rho_{max}$, $\Delta\rho_{min}$ (e Å^{-3})	0.17, -0.18	0.20, -0.34

Table S3. (continuation)

	Naphthalene at 10.8 GPa	Naphthalene at 14.8 GPa	Naphthalene at 20.3 GPa
CCDC deposition number	2364321	2364322	2364323
Crystal data			
Chemical formula	$C_{10}H_8$	$C_{10}H_8$	$C_{10}H_8$
M_r	128.175	128.175	128.175
Crystal system, space group	Monoclinic, $P2_1/c$	Monoclinic, $P2_1/c$	Monoclinic, $P2_1/c$
a, b, c (Å)	6.779(14), 5.3433(8), 6.901(3)	6.622(11), 5.2755(7), 6.771(2)	6.447(11), 5.1951(7), 6.6135(18)
α, β, γ (°)	90, 109.04(13), 90	90, 107.96(9), 90	90, 106.64(8), 90
V (Å^3)	236.3(5)	225.0(4)	212.2(4)
Z	2	2	2
Density (Mg/m^3)	1.802	1.892	2.006
Wavelength (Å)	0.3738	0.3738	0.3738
μ (mm^{-1})	0.044	0.046	0.049
Data collection			
Absorption correction	Multi-scan	Multi-scan	Multi-scan
T_{min}, T_{max}	1.00, 0.39	1.00, 0.14	1.00, 0.03
No. of measured, independent and observed reflections	492, 324, 209	560, 281, 167	560, 321, 221
R_{int}	0.035	0.030	0.025
θ_{max} (°)	21.24	17.25	20.84
Refinement			
Refinement on	F^2	F^2	F^2
$R[F^2>2\sigma(F^2)]$,$wR(F^2)$, S	0.089, 0.203, 0.927	0.098, 0.226, 0.946	0.075, 0.174, 0.901
Data / restraints / parameters	324/ 0/ 46	281/ 0/ 46	321/ 0/ 46
H-atom treatment	Refined by ride model	Refined by ride model	Refined by ride model
Weighting scheme	$w=1/[\sigma^2(F_o^2)+(0.1801P)^2]$ where $P=(F_o^2+2F_c^2)/3$	$w=1/[\sigma^2(F_o^2)+(0.1987P)^2]$ where $P=(F_o^2+2F_c^2)/3$	$w=1/[\sigma^2(F_o^2)+(0.1479P)^2]$ where $P=(F_o^2+2Fc^2)/3$
$\Delta\rho_{max}$, $\Delta\rho_{min}$ (e Å^{-3})	0.28, -0.28	0.26, -0.27	0.24, -0.29

Table S3. (continuation)

	Naphthalene at 24.5 GPa	Naphthalene at 28.5 GPa	Naphthalene at 35.1 GPa
CCDC deposition number	2364324	2364325	2364326
Crystal data			
Chemical formula	$C_{10}H_8$	$C_{10}H_8$	$C_{10}H_8$
M_r	128.175	128.175	128.175
Crystal system, space group	Monoclinic, $P2_1/c$	Monoclinic, $P2_1/c$	Monoclinic, $P2_1/c$
a, b, c (Å)	6.311(14), 5.1374(7), 6.5216(18)	6.231(10), 5.1163(7), 6.427(2)	6.125(11), 5.0575(7), 6.322(3)
α, β, γ (°)	90, 106.01(10), 90	90, 105.08(10), 90	90, 104.40(11), 90
V (Å^3)	203.2(5)	197.8(4)	189.7(4)
Z	2	2	2
Density (Mg/m^3)	2.095	2.152	2.244
Wavelength (Å)	0.3738	0.3738	0.3738
μ (mm^{-1})	0.051	0.053	0.055
Data collection			
Absorption correction	Multi-scan	Multi-scan	Multi-scan
T_{min}, T_{max}	1.00, 0.21	1.00, 0.07	1.00, 0.31
No. of measured, independent and observed reflections	423, 277, 169	480, 240, 150	464, 227, 154
R_{int}	0.023	0.044	0.023
θ_{max} (°)	21.08	17.23	17.54
Refinement			
Refinement on	F^2	F^2	F^2
$R[F^2>2\sigma(F^2)]$, $wR(F^2)$, S	0.096, 0.233, 0.978	0.078, 0.183, 0.986	0.093, 0.223, 1.012
Data / restraints / parameters	277/ 0/ 46	240/ 0/ 46	227/ 12/ 46
H-atom treatment	Refined by ride model	Refined by ride model	Refined by ride model
Weighting scheme	$w=1/[\sigma^2(F_o^2)+(0.1889P)^2]$ where $P=(F_o^2+2F_c^2)/3$	$w=1/[\sigma^2(F_o^2)+(0.1442P)^2]$ where $P=(F_o^2+2F_c^2)/3$	$w=1/[\sigma^2(F_o^2)+(0.1944P)^2]$ where $P=(F_o^2+2F_c^2)/3$
$\Delta\rho_{max}$, $\Delta\rho_{min}$ (e Å^{-3})	0.28, -0.24	0.24, -0.28	0.28, -0.27

Table S3. (continuation)

	Naphthalene at 43.3 GPa	Naphthalene at 46.6 GPa	Naphthalene at 50.7 GPa
CCDC deposition number	2364327	2364328	2364329
Crystal data			
Chemical formula	$C_{10}H_8$	$C_{10}H_8$	$C_{10}H_8$
M_r	128.175	128.175	128.175
Crystal system, space group	Monoclinic, $P2_1/c$	Monoclinic, $P2_1/c$	Monoclinic, $P2_1/c$
a, b, c (Å)	6.00(3), 5.009(2), 6.080(8)	6.03(2), 4.9580(17), 6.103(8)	5.93(2), 4.913(2), 6.035(11)
α, β, γ (°)	90, 102.6(3), 90	90, 103.7(3), 90	90, 103.3(3), 90
V (Å^3)	178.3(10)	177.2(7)	171.2(8)
Z	2	2	2
Density (Mg/m^3)	2.388	2.403	2.486
Wavelength (Å)	0.3738	0.3738	0.3738
μ (mm^{-1})	0.058	0.059	0.061
Data collection			
Absorption correction	Multi-scan	Multi-scan	Multi-scan
T_{min}, T_{max}	1.00, 0.20	1.00, 0.13	1.00, 0.34
No. of measured, independent and observed reflections	318, 169, 99	288, 188, 107	164, 122, 67
R_{int}	0.045	0.035	0.012
θ_{max} (°)	16.13	16.65	14.834
Refinement			
Refinement on	F^2	F^2	F^2
R[F^2>2σ(F^2)],wR(F^2), S	0.098, 0.238, 1.166	0.097, 0.233, 1.082	0.089, 0.214, 1.113
Data / restraints / parameters	169/ 0/ 46	188/ 0/ 46	122/ 12/ 46
H-atom treatment	Refined by ride model	Refined by ride model	Refined by ride model
Weighting scheme	w=1/[$\sigma^2(F_o^2)$+(0.1908P)^2] where P=(F_o^2+2F_c^2)/3	w=1/[$\sigma^2(F_o^2)$+(0.1956P)^2] where P=(F_o^2+2F_c^2)/3	w=1/[$\sigma^2(F_o^2)$+(0.2000P)^2] where P=(F_o^2+2Fc^2)/3
$\Delta\rho_{max}$, $\Delta\rho_{min}$ (e Å^{-3})	0.20, -0.18	0.24, -0.24	0.22, -0.18

Table S4. Lattice parameters for naphthalene up to 50.7 GPa and anthracene up to 42.3GPa in this work.

Polymorph	Pressure, GPa	*a*, Å	*b*, Å	*c*, Å	*β*, °
Naphthalene	0	8.147(6)	6.0035(8)	8.293(3)	116.08(7)
Naphthalene	6.2	7.168(7)	5.5599(6)	7.320(3)	111.07(8)
Naphthalene	9.9	6.790(8)	5.3738(8)	6.959(3)	109.22(9)
Naphthalene	10.8	6.779(14)	5.3433(8)	6.901(3)	109.04(13)
Naphthalene	14.8	6.622(11)	5.2755(7)	6.771(2)	107.96(9)
Naphthalene	20.3	6.447(11)	5.1951(7)	6.6135(18)	106.64(8)
Naphthalene	24.5	6.311(14)	5.1374(7)	6.5216(18)	106.01(10)
Naphthalene	28.5	6.231(10)	5.1163(7)	6.427(2)	105.08(10)
Naphthalene	35.1	6.125(11)	5.0575(7)	6.322(3)	104.40(11)
Naphthalene	43.3	6.00(3)	5.009(2)	6.080(8)	102.6(3)
Naphthalene	46.6	6.03(2)	4.9580(17)	6.103(8)	103.7(3)
Naphthalene	50.7	5.93(2)	4.913(2)	6.035(11)	103.3(3)
Anthracene	0	9.488(5)	6.0253(3)	8.5642(14)	103.52(3)
Anthracene	1.5	8.0741(10)	5.8283(7)	9.032(11)	101.44(4)
Anthracene	4	8.747(11)	5.6633(7)	7.718(2)	99.35(7)
Anthracene	8.3	8.522(6)	5.5444(4)	7.4715(11)	97.85(3)
Anthracene	10.8	8.422(5)	5.4845(4)	7.3579(8)	97.26(2)
Anthracene	13.5	8.291(3)	5.4007(3)	7.2067(6)	96.21(2)
Anthracene	15.8	8.209(5)	5.3592(4)	7.1344(9)	95.72(3)
Anthracene	18.4	8.156(6)	5.3045(3)	7.0586(3)	95.183(13)
Anthracene	21.5	8.075(5)	5.2590(4)	6.9637(9)	94.70(3)
Anthracene	25.3	7.989(10)	5.2110(4)	6.9048(5)	94.05(2)
Anthracene	29	7.935(11)	5.1607(5)	6.8359(6)	93.54(2)
Anthracene	34	7.855(16)	5.1011(9)	6.7534(8)	93.02(3)
Anthracene	35.5	7.843(16)	5.0901(9)	6.7422(8)	92.91(3)
Anthracene	38.6	7.816(16)	5.0564(7)	6.6915(7)	92.66(3)
Anthracene	42.3	7.769(19)	5.0266(9)	6.6325(8)	92.14(4)

Table S5. Unit cell volume per formula unit for naphthalene up to 50.7 GPa and anthracene up to 42.3GPa in this work.

Polymorph/ pressure transmitting medium	Pressure, GPa	Volume per formula unit, Å^3
Naphthalene/ He	0	182.2(2)
Naphthalene/ He	6.2	136.10(15)
Naphthalene/ He	9.9	119.90(15)
Naphthalene/ He	10.8	118.2(2)
Naphthalene/ He	14.8	112.5(2)
Naphthalene/ He	20.3	106.1(2)
Naphthalene/ He	24.5	101.6(3)
Naphthalene/ He	28.5	98.9(2)
Naphthalene/ He	35.1	94.9(2)
Naphthalene/ He	43.3	89.2(5)
Naphthalene/ He	46.6	88.6(4)
Naphthalene/ He	50.7	85.6(4)
Anthracene/ He	0	238.00(15)
Anthracene/ He	1.5	208.3(3)
Anthracene/ He	4	188.6(3)
Anthracene/ He	8.3	174.85(15)
Anthracene/ He	10.8	168.57(10)
Anthracene/ He	13.5	160.40(7)
Anthracene/ He	15.8	156.16(9)
Anthracene/ He	18.4	152.05(10)
Anthracene/ He	21.5	147.35(10)
Anthracene/ He	25.3	143.4(2)
Anthracene/ He	29	139.7(2)
Anthracene/ He	34	135.1(3)
Anthracene/ He	35.5	134.4(3)
Anthracene/ He	38.6	132.1(3)
Anthracene/ He	42.3	129.4(3)

Table S6. DFT-calculated unit cell volume per formula unit for naphthalene up to 50.7 GPa and anthracene up to 42.3GPa.

Polymorph	Pressure (GPa)	Volume per formula unit (Å^3)
Naphthalene	0	167.17
Naphthalene	4.4	135.70
Naphthalene	9.8	121.62
Naphthalene	22.3	105.80
Naphthalene	31.3	99.10
Naphthalene	53.1	88.70
Anthracene	0	222.29
Anthracene	0.9	208.29
Anthracene	3.3	188.62
Anthracene	6.4	174.84
Anthracene	8.4	168.57
Anthracene	11.7	160.40
Anthracene	16.3	152.07
Anthracene	22.7	143.37
Anthracene	26.2	139.70
Anthracene	32.1	134.41
Anthracene	34.9	132.09
Anthracene	38.7	129.42

Table S7. Summary of available HP XRD experimental data for naphthalene.

**Ref.**	**Pressure interval (GPa)**	**Pressure medium**	**DAC type**	**Instrument, radiation type**	**Unit cell parameters at ambient conditions**	V_0 **(Å³)**	K_0 **(GPa)**	K'	**EOS used**	**Comments**
This study	0 – 50.7	Helium	Membrane DAC	Synchrotron, λ = 0.3685 Å	a = 8.147(6) Å, b = 6.0035(8) Å, c = 8.293(3) Å, β = 116.08(7) °, V = 364.3(4) Å^3	364.3	8.4(10)	5.5(6)	3rd Birch-Murnaghan (fixed V_0)	Not observed phase transition
(3)	0.4 – 2.1	Dichloro-methane	Merrill–Bassett DAC	In house, λ = 0.71073 Å (Mo $K\alpha$)	Not mentioned	361	3.6(6)	19(5)	3rd Birch-Murnaghan (fixed V_0)*	Not observed phase transition
(4)	0 – 12.9	No medium	Membrane DAC Helios (EasyLab)	Synchrotron, λ = 0.3685 Å	a = 8.259(1) Å, b = 5.983(1) Å, c = 8.674(1) Å, β = 122.65(1) °, V = 360.8(1) Å^3	361	8.4(3)	7.2(3)	Vinet (fixed V_0)	Not observed phase transition
(8)	0 – 5.6	No medium	Membrane DAC Helios (EasyLab)	Synchrotron, λ = 0.3685 Å	a = 8.259(1) Å, b = 5.984(1) Å, c = 8.676(1) Å, β = 122.65(1) °, V = 360.98(5) Å^3	361	7.9(3)	7.5(3)	Vinet (fixed V_0)	Not observed phase transition
(9)	0 – 21.3	Neon	Not mentioned	Synchrotron, λ = 0.6128 Å	Not mentioned				Not mentioned**	Not observed phase transition

* The EOS was fitted using the lattice parameters provided for each pressure point in the paper.

** The paper does not provide lattice parameters for each pressure point.

Table S8. Summary of available theoretical data for naphthalene.

**Ref.**	**Pressure interval (GPa)**	**Calculation program**	**Potentials**	**Dispersion correction**	**Temperature**	**Unit cell parameters at ambient conditions**	$\boldsymbol{V_0}$ **(Å^3) =V/Z**	$\boldsymbol{K_0}$ **(GPa)**	$\boldsymbol{K'}$	**EOS used**
This study	0 – 53.1	VASP	GGA functional of PBE	DFT-D3	0K	a = 7.764 Å, b = 5.876 Å, c = 8.025 Å, β = 114.05 °, V = 334.4 Å^3	167.2	10.8(2)	6.99(13)	3rd Birch-Murnaghan (fixed V_0)
(10)	0 – 30	VASP	GGA functional of PBE	vdW-DF2 correlation functional	0K	a = 8.221 Å, b = 5.904 Å, c = 8.550 Å, β = 123.10 °, V = 347.6 Å^3	173.8	10.26	8.18	3rd Birch-Murnaghan (fixed V_0)
(11)	0 – 22	VASP	GGA functional of PBE	optPBE-vdW exchange–correlation functional	0K	a = 8.213 Å, b = 6.002 Å, c = 8.731 Å, β = 123.58 °, V = 358.58 Å^3	179	8.9(3)	7.3(1)	Vinet (unfixed V_0)

Table S9. Comparison of lattice parameters from different computational methods and experimental data for various compounds at ambient pressure.

**Ref.**	**Compound**	**Unit cell parameter**	**Temperature**	**Dispersion correction**	**Note**
CSD reference code NAPHTA31 (6)	Naphthalene	a = 8.080(5) Å, b = 5.933(2) Å, c = 8.632(5) Å, β = 124.65(4) °, V = 340.4(3) $Å^3$	5K		Exp.
This study	Naphthalene	a = 7.7639 Å, b = 5.8761 Å, c = 8.0247 Å, β = 114.05 °, V = 334.33 $Å^3$	0K	DFT-D3	DFT
(10)	Naphthalene	a = 8.221 Å, b = 5.904 Å, c = 8.550 Å, β = 123.10 °, V = 347.6 $Å^3$	0K	vdW-DF2 correlation functional	DFT
(11)	Naphthalene	a = 8.213 Å, b = 6.002 Å, c = 8.731 Å, β = 123.58 °, V = 358.58 $Å^3$	0K	optPBE-vdW exchange–correlation functional	DFT
CSD reference code ANTCEN16 (12)	Perdeutero-anthracene ($C_{14}D_{10}$)	a = 8.37(3) Å, b = 6.00(2) Å, c = 11.12(4) Å, β = 125.4 °, V = 455(2) $Å^3$	16K		Exp.
This study	Anthracene	a = 8.3648 Å, b = 5.9071 Å, c = 11.0927 Å, β = 125.80 °, V = 444.58 $Å^3$	0K	DFT-D3	DFT
(13)	Anthracene	a = 8.5494 Å, b = 6.01151 Å, c = 11.0647 Å, β = 125.12 °, V = 465.42 $Å^3$	0K	Grimme′06 scheme	DFT
(11)	Anthracene	a = 8.603 Å, b = 5.954 Å, c = 11.166 Å, β = 124.51 °, V = 471.30 $Å^3$	0K	optPBE-vdW exchange–correlation functional	DFT

Table S10. Interplanar angles of naphthalene and anthracene from experiments.

Polymorph	Pressure, GPa	Interplanar angle, °
Naphthalene	0	53.5(3)
Naphthalene	6.2	46.1(3)
Naphthalene	9.9	44.2(3)
Naphthalene	10.8	44.9(3)
Naphthalene	14.8	42.9(4)
Naphthalene	20.3	42.5(3)
Naphthalene	24.5	41.4(4)
Naphthalene	28.5	42.5(4)
Naphthalene	35.1	40.4(4)
Naphthalene	43.3	43.5(6)
Naphthalene	46.6	38.1(5)
Naphthalene	50.7	40.6(8)
Anthracene	0	51.4(2)
Anthracene	1.5	49.0(5)
Anthracene	4.0	46.6(7)
Anthracene	8.3	44.3(3)
Anthracene	10.8	43.98(18)
Anthracene	13.5	43.4(2)
Anthracene	15.8	43.2(2)
Anthracene	18.4	43.2(2)
Anthracene	21.5	43.0(2)
Anthracene	25.3	42.5(2)
Anthracene	29	42.15(17)
Anthracene	34	42.1(2)
Anthracene	35.5	42.0(2)
Anthracene	38.6	42.1(2)
Anthracene	42.3	41.8(3)

Table S11. Experimental crystallographic data for anthracene at ambient conditions obtained by single-crystal X-ray diffraction in this work and Perdeuteroanthracene by neutron diffraction in ref. (14).

	Anthracene at ambient condition	Perdeuteroanthracene at 293 K[14]
CCDC deposition number	2364306	1103075
Crystal data		
Chemical formula	$C_{14}H_{10}$	$C_{14}D_{10}$
M_r	178.22	188.280
Crystal system, space group	Monoclinic, $P2_1/c$	Monoclinic, $P2_1/c$
a, b, c (Å)	9.488(5), 6.0253(3), 8.5642(14)	9.451(10), 6.016(6), 8.542(5)
α, β, γ (°)	90, 103.52(3), 90	90, 103.49(9), 90
V (Å^3)	476.0(3)	472.3(7)
Z	2	2
Density (Mg/m^3)	1.243	1.253
Wavelength (Å)	0.3738	1.025
μ (mm^{-1})	0.030	0.9
Data collection		
Absorption correction	Multi-scan	
T_{min}, T_{max}	1.00, 0.46	
No. of measured, independent and observed reflections	926, 506, 347	1145, 1093, 1053
R_{int}	0.005	0.020
θ_{max} (°)	15.46	
Refinement		
Refinement on	F^2	
$R[F^2>2\sigma(F^2)]$,$wR(F^2)$, S	0.038, 0.119, 1.021	0.034, 0.034
Data / restraints / parameters	506/ 0/ 64	
H-atom treatment	Refined by ride model	
Weighting scheme	$w=1/[\sigma^2(F_o^2)+(0.0857P)^2+0.0049P]$ where $P=(F_o^2+2F_c^2)/3$	
$\Delta\rho_{max}$, $\Delta\rho_{min}$ (e Å^{-3})	0.07, -0.08	

Table S12. Experimental crystallographic data for anthracene obtained by single-crystal X-ray diffraction at room temperature in this work.

	Anthracene at 1.5 GPa	Anthracene at 4.0 GPa
CCDC deposition number	2364307	2364308
Crystal data		
Chemical formula	$C_{14}H_{10}$	$C_{14}H_{10}$
M_r	178.22	178.22
Crystal system, space group	Monoclinic, $P2_1/c$	Monoclinic, $P2_1/c$
a, b, c (Å)	8.0741(10), 5.8283(7), 9.032(11)	8.747(11), 5.6633(7), 7.718(2)
α, β, γ (°)	90, 101.44(4), 90	90, 99.35(7), 90
V (Å^3)	416.6(5)	377.3(5)
Z	2	2
Density (Mg/m^3)	1.421	1.569
Wavelength (Å)	0.410	0.410
μ (mm^{-1})	0.037	0.041
Data collection		
Absorption correction	Multi-scan	Multi-scan
T_{min}, T_{max}	1.00, 0.66	1.00, 0.09
No. of measured, independent and observed reflections	731, 336, 163	515, 285, 130
R_{int}	0.073	0.053
θ_{max} (°)	15.711	15.63
Refinement		
Refinement on	F^2	F^2
$R[F^2>2\sigma(F^2)]$,$wR(F^2)$, S	0.059, 0.156, 0.631	0.054, 0.110, 0.923
Data / restraints / parameters	336/ 81/ 64	285/ 36/ 64
H-atom treatment	Refined by ride model	Refined by ride model
Weighting scheme	$w=1/[\sigma^2(F_o^2)]$ where $P=(F_o^2+2Fc^2)/3$	$w=1/[\sigma^2(F_o^2)+(0.0377P)^2]$ where $P=(F_o^2+2F_c^2)/3$
$\Delta\rho_{max}$, $\Delta\rho_{min}$ (e Å^{-3})	0.09, -0.10	0.13, -0.12

Table S12. (continuation)

	Anthracene at 8.3 GPa	Anthracene at 10.8 GPa	Anthracene at 13.5 GPa
CCDC deposition number	2364309	2364310	2364311
Crystal data			
Chemical formula	$C_{14}H_{10}$	$C_{14}H_{10}$	$C_{14}H_{10}$
M_r	178.235	178.235	178.235
Crystal system, space group	Monoclinic, $P2_1/c$	Monoclinic, $P2_1/c$	Monoclinic, $P2_1/c$
a, b, c (Å)	8.522(6), 5.5444(4), 7.4715(11)	8.422(5), 5.4845(4), 7.3579(8)	8.291(3), 5.4007(3), 7.2067(6)
α, β, γ (°)	90, 97.85(3), 90	90, 97.26(2), 90	90, 96.21(2), 90
V (Å^3)	349.7(3)	337.12(19)	320.81(14)
Z	2	2	2
Density (Mg/m^3)	1.692	1.756	1.845
Wavelength (Å)	0.410	0.410	0.410
μ (mm^{-1})	0.044	0.046	0.048
Data collection			
Absorption correction	Multi-scan	Multi-scan	Multi-scan
T_{min}, T_{max}	1.00, 0.01	1.00, 0.33	1.00, 0.36
No. of measured, independent and observed reflections	540, 354, 237	559, 402, 246	711, 459, 248
R_{int}	0.040	0.019	0.038
θ_{max} (°)	16.31	18.94	21.019
Refinement			
Refinement on	F^2	F^2	F^2
R[F^2>2σ(F^2)],wR(F^2), S	0.071, 0.191, 1.065	0.045, 0.102, 0.930	0.059, 0.158, 1.094
Data / restraints / parameters	354/ 6/ 64	402/ 0/ 64	459/ 0/ 64
H-atom treatment	Refined by ride model	Refined by ride model	Refined by ride model
Weighting scheme	$w=1/[\sigma^2(F_o^2)+(0.1466P)^2]$ where $P=(F_o^2+2F_c^2)/3$	$w=1/[\sigma^2(F_o^2)+(0.0683P)^2]$ where $P=(F_o^2+2F_c^2)/3$	$w=1/[\sigma^2(F_o^2)+(0.1037P)^2+0.0551P]$ where $P=(F_o^2+2Fc^2)/3$
$\Delta\rho_{max}$, $\Delta\rho_{min}$ (e Å^{-3})	0.20, -0.18	0.12, -0.12	0.20, -0.19

Table S12. (continuation)

	Anthracene at 15.8 GPa	Anthracene at 18.4 GPa	Anthracene at 21.5 GPa
CCDC deposition number	2364312	2364313	2364314
Crystal data			
Chemical formula	$C_{14}H_{10}$	$C_{14}H_{10}$	$C_{14}H_{10}$
M_r	178.235	178.235	178.235
Crystal system, space group	Monoclinic, $P2_1/c$	Monoclinic, $P2_1/c$	Monoclinic, $P2_1/c$
a, b, c (Å)	8.209(5), 5.3592(4), 7.1344(9)	8.156(6), 5.3045(3), 7.0586(3)	8.075(5), 5.2590(4), 6.9637(9)
α, β, γ (°)	90, 95.72(3), 90	90, 95.183(13), 90	90, 94.70(3), 90
V (Å^3)	312.31(18)	304.1(2)	294.7(2)
Z	2	2	2
Density (Mg/m^3)	1.895	1.946	2.008
Wavelength (Å)	0.410	0.410	0.410
μ (mm^{-1})	0.049	0.051	0.052
Data collection			
Absorption correction	Multi-scan	Multi-scan	Multi-scan
T_{min}, T_{max}	1.00, 0.60	1.00, 0.37	1.00, 0.04
No. of measured, independent and observed reflections	524, 348, 225	491, 291, 234	608, 412, 251
R_{int}	0.018	0.021	0.018
θ_{max} (°)	17.012	16.985	21.022
Refinement			
Refinement on	F^2	F^2	F^2
$R[F^2>2\sigma(F^2)]$,$wR(F^2)$, S	0.044, 0.110, 1.066	0.062, 0.177, 1.096	0.071, 0.196, 1.029
Data / restraints / parameters	348/ 0/ 64	291/ 0/ 64	412/ 0/ 64
H-atom treatment	Refined by ride model	Refined by ride model	Refined by ride model
Weighting scheme	$w=1/[\sigma^2(F_o^2)+(0.0712P)^2]$ where $P=(F_o^2+2Fc^2)/3$	$w=1/[\sigma^2(F_o^2)+(0.1512P)^2]$ where $P=(F_o^2+2Fc^2)/3$	$w=1/[\sigma^2(F_o^2)+(0.1451P)^2]$ where $P=(F_o^2+2Fc^2)/3$
$\Delta\rho_{max}$, $\Delta\rho_{min}$ (e Å^{-3})	0.13, -0.12	0.15, -0.21	0.23, -0.23

Table S12. (continuation)

	Anthracene at 25.3 GPa	Anthracene at 29.0 GPa	Anthracene at 34.0 GPa
CCDC deposition number	2364315	2364316	2364317
Crystal data			
Chemical formula	$C_{14}H_{10}$	$C_{14}H_{10}$	$C_{14}H_{10}$
M_r	178.235	178.235	178.235
Crystal system, space group	Monoclinic, $P2_1/c$	Monoclinic, $P2_1/c$	Monoclinic, $P2_1/c$
a, b, c (Å)	7.989(10), 5.2110(4), 6.9048(5)	7.935(11), 5.1607(5), 6.8359(6)	7.855(16), 5.1011(9), 6.7534(8)
α, β, γ (°)	90, 94.05(2), 90	90, 93.54(2), 90	90, 93.02(3), 90
V (Å^3)	286.7(4)	279.4(4)	270.2(6)
Z	2	2	2
Density (Mg/m^3)	2.064	2.118	2.190
Wavelength (Å)	0.410	0.410	0.410
μ (mm^{-1})	0.054	0.055	0.057
Data collection			
Absorption correction	Multi-scan	Multi-scan	Multi-scan
T_{min}, T_{max}	1.00, 0.27	1.00, 0.21	1.00, 0.47
No. of measured, independent and observed reflections	577, 343, 239	528, 334, 238	432, 285, 196
R_{int}	0.019	0.011	0.013
θ_{max} (°)	20.804	20.983	21.212
Refinement			
Refinement on	F^2	F^2	F^2
R[F^2>2σ(F^2)],wR(F^2), S	0.082, 0.243, 1.086	0.064, 0.186, 1.101	0.056, 0.154, 0.984
Data / restraints / parameters	343/ 6/ 64	334/ 0/ 64	285/ 6/ 64
H-atom treatment	Refined by ride model	Refined by ride model	Refined by ride model
Weighting scheme	w=1/[$\sigma^2(F_o^2)+(0.1954P)^2$] where $P=(F_o^2+2Fc^2)/3$	w=1/[$\sigma^2(F_o^2)+(0.1523P)^2$] where $P=(F_o^2+2Fc^2)/3$	w=1/[$\sigma^2(F_o^2)+(0.1417P)^2$] where $P=(F_o^2+2Fc^2)/3$
$\Delta\rho_{max}$, $\Delta\rho_{min}$ (e Å^{-3})	0.18, -0.18	0.18, -0.21	0.17, -0.18

Table S12. (continuation)

	Anthracene at 35.5 GPa	Anthracene at 38.6 GPa	Anthracene at 42.3 GPa
CCDC deposition number	2364318	2364319	2364320
Crystal data			
Chemical formula	$C_{14}H_{10}$	$C_{14}H_{10}$	$C_{14}H_{10}$
M_r	178.235	178.235	178.235
Crystal system, space group	Monoclinic, $P2_1/c$	Monoclinic, $P2_1/c$	Monoclinic, $P2_1/c$
a, b, c (Å)	7.843(16), 5.0901(9), 6.7422(8)	7.816(16), 5.0564(7), 6.6915(7)	7.769(19), 5.0266(9), 6.6325(8)
α, β, γ (°)	90, 92.91(3), 90	90, 92.66(3), 90	90, 92.14(4), 90
V (Å^3)	268.8(6)	264.2(5)	258.8(6)
Z	2	2	2
Density (Mg/m^3)	2.202	2.241	2.287
Wavelength (Å)	0.410	0.410	0.410
μ (mm^{-1})	0.057	0.058	0.060
Data collection			
Absorption correction	Multi-scan	Multi-scan	Multi-scan
T_{min}, T_{max}	1.00, 0.12	1.00, 0.34	1.00, 0.53
No. of measured, independent and observed reflections	494, 309, 213	467, 284, 196	448, 284, 175
R_{int}	0.015	0.013	0.032
θ_{max} (°)	21.242	21.051	20.708
Refinement			
Refinement on	F^2	F^2	F^2
$R[F^2>2\sigma(F^2)]$,$wR(F^2)$, S	0.056, 0.155, 1.021	0.056, 0.157, 1.074	0.059, 0.167, 1.102
Data / restraints / parameters	309/ 0/ 64	284/ 0/ 64	284/ 0/ 64
H-atom treatment	Refined by ride model	Refined by ride model	Refined by ride model
Weighting scheme	$w=1/[\sigma^2(F_o^2)+(0.1307P)^2]$ where $P=(F_o^2+2Fc^2)/3$	$w=1/[\sigma^2(F_o^2)+(0.1288P)^2]$ where $P=(F_o^2+2Fc^2)/3$	$w=1/[\sigma^2(F_o^2)+(0.1010P)^2+0.1928P]$ where $P=(F_o^2+2Fc^2)/3$
$\Delta\rho_{max}$, $\Delta\rho_{min}$ (e Å^{-3})	0.17, -0.16	0.15, -0.20	0.14, -0.17

Table S13. Summary of available HP XRD experimental data for anthracene.

**Ref.**	**Pressure interval (GPa)**	**Pressure medium**	**Instrument, radiation type**	**Unit cell parameters at ambient conditions**	**V_0 (Å^3)**	**K_0 (GPa)**	**K'**	**EOS used**	**Comments**
This study	0 – 42.3	Helium	Membrane DAC	a = 9.488(5) Å, b = 6.0253(3) Å, c = 8.5642(14) Å, β = 103.52(3) °, V = 476.0(3) Å^3	476	10.8(2)	6.99(13)	3rd Birch-Murnaghan (fixed V_0)	Not observed phase transition
(15)	0 – 5.6	No medium	In house, λ = 0.71073 Å (Mo $K\alpha$)	a = 8.542 Å, b = 6.015 Å, c = 11.192 Å, β = 124.7 °, V = 472.72 Å^3	472.72	7.7(2)	5	Murnaghan (fixed V_0)	At pressures above 2.4 GPa, the c parameter increased significantly, and the volume showed a slight increase, indicating a phase transformation and that the low-pressure indexing is no longer applicable.
(16)	0.16 – 22.7	Methanol–ethanol mixture (4:1 by volume) from 0.16 GPa to 10 GPa, Helium from 6.77 to 22.7 GPa	Synchrotron, λ = 0.9204 Å	Not mentioned	473.22	6.80(15)	9.00(11)	Vinet (fixed V_0)	Not observed phase transition

Table S14. Summary of available theoretical data for anthracene.

**Ref.**	**Pressure interval (GPa)**	**Calculation program**	**Potentials**	**Dispersion correction**	**Temperature**	**Unit cell parameters at ambient conditions**	V_0 **(Å^3)**	K_0 **(GPa)**	K'	**EOS used**
This study	0 – 38.7	VASP	GGA functional of PBE	DFT-D3	0K	a = 9.191 Å, b = 5.907 Å, c = 8.365 Å, β = 101.78 °, V = 444.58 Å^3	444.6	11.57(13)	7.34(8)	3rd Birch-Murnaghan
(11)	0 – 21	VASP	GGA functional of PBE	optPBE-vdW exchange–correlation functional	0K	a = 8.603 Å, b = 5.954 Å, c = 11.166 Å, β = 124.51 °, V = 471.30 Å^3	467.7(6)	10.7(1)	7.1(1)	Vinet (unfixed V_0)
(13)	0 – 20	Quantum ESPRESSO	GGA functional of PBE	Grimme′06 scheme	0K	a = 8.5494 Å, b = 6.01151 Å, c = 11.0647 Å, β = 125.12 °, V = 465.42 Å^3	464.712	11.448	6.613	Vinet (fixed V_0)

Table S15. The experimental average the C-H bond length in anthracene and naphthalene molecules.

**Polymorph**	**Pressure, GPa**	**C-H Avg, Å**
Naphthalene	0	1.08(5)
Naphthalene	6.2	1.05(6)
Naphthalene	9.9	1.08(5)
Naphthalene	10.8	1.05(7)
Naphthalene	14.8	1.09(8)
Naphthalene	20.3	1.07(4)
Naphthalene	28.5	1.07(6)
Naphthalene	35.1	1.08(6)
Anthracene	0	1.09(2)
Anthracene	8.3	1.07(7)
Anthracene	10.8	1.06(4)
Anthracene	13.5	1.09(5)
Anthracene	15.8	1.07(4)
Anthracene	18.4	1.11(5)
Anthracene	21.5	1.04(5)
Anthracene	25.3	1.05(5)
Anthracene	29	1.02(4)
Anthracene	34	1.05(5)
Anthracene	35.5	1.04(4)
Anthracene	38.6	1.05(6)
Anthracene	42.3	1.04(6)

Table S16. The calculation average C-H bond lengths in anthracene and naphthalene molecules.

**Polymorph**	**Pressure, GPa**	**C-H Avg, Å**
Naphthalene	0	1.09
Naphthalene	4.4	1.087
Naphthalene	9.8	1.083
Naphthalene	22.3	1.076
Naphthalene	31.3	1.072
Naphthalene	53.1	1.064
Anthracene	0	1.089
Anthracene	6.36	1.082
Anthracene	8.36	1.08
Anthracene	11.71	1.077
Anthracene	16.29	1.074
Anthracene	22.73	1.07
Anthracene	26.15	1.068
Anthracene	32.05	1.064
Anthracene	34.94	1.063
Anthracene	38.7	1.061

Table S17. Percentage contribution to the Hirschfeld surface area for the various close intermolecular contacts (H···H, C···H and C···C) as a function of pressure for molecules in naphthalene, anthracene, and in pyrene and benzo[*a*]pyrene polymorphs.

**Polymorph**	**Pressure, GPa**	**H···H, %**	**C···H, %**	**C···C, %**
Naphthalene	0	54.7	45.1	0.2
Naphthalene	6.2	48.4	49.7	1.9
Naphthalene	9.9	47.4	49.4	3.2
Naphthalene	10.8	46.6	50.4	3
Naphthalene	14.8	45.9	50.2	3.9
Naphthalene	20.3	45	50.7	4.3
Naphthalene	24.5	44.3	50.9	4.8
Naphthalene	28.5	43	52	5
Naphthalene	35.1	42.1	52.2	5.7
Naphthalene	43.3	40.7	52.9	6.4
Anthracene	0	48.8	50.4	0.8
Anthracene	1.5	46.2	52.7	1.1
Anthracene	4	43.5	53.9	2.6
Anthracene	8.3	42.6	53.9	3.5
Anthracene	10.8	42.2	54.1	3.7
Anthracene	13.5	41.4	54.5	4.1
Anthracene	15.8	40.4	55.4	4.2
Anthracene	18.4	39.7	56	4.3
Anthracene	21.5	38.7	56.8	4.5
Anthracene	25.3	38.3	56.9	4.8
Anthracene	29	37.8	57.1	5.1
Anthracene	34	37.2	57.6	5.2
Anthracene	35.5	36.6	58.5	4.9
Anthracene	38.6	36.1	58.8	5.1
Anthracene	42.3	35.9	58.8	5.3

Table S17. (continue)

**Polymorph**	**Pressure, GPa**	**H···H, %**	**C···H, %**	**C···C, %**
Pyrene-I	0	53.6	35.7	10.7
Pyrene-II	0.7	49.3	39.9	10.8
Pyrene-II	1.4	48.9	39	12.1
Pyrene-IV	2.7	45.1	43.1	11.8
Pyrene-IV	4.3	44.3	43.5	12.2
Pyrene-V	7.3	48.2	35	16.8
Pyrene-V	9.5	46.9	35.7	17.4
Pyrene-V	15.4	45.3	36.4	18.3
Pyrene-V	20.2	44.5	36.7	18.8
Pyrene-V	25.2	43.4	37.3	19.3
Pyrene-V	29.8	42.9	37.5	19.6
Pyrene-V	35.5	42.5	37.4	20.1
Benzo[*a*]pyrene-I	0	57.9	21.2	20.9
Benzo[*a*]pyrene-I	2.2	54.2	23.3	22.5
Benzo[*a*]pyrene-II	4.8	55.7	17	27.3
Benzo[*a*]pyrene-III	7.1	54.6	17.8	27.6
Benzo[*a*]pyrene-III	9.1	55	15.7	29.3
Benzo[*a*]pyrene-III	11.8	51.6	18.2	30.2
Benzo[*a*]pyrene-III	14.2	52.8	17.4	29.8
Benzo[*a*]pyrene-III	21.1	51.5	18	30.5
Benzo[*a*]pyrene-III	27.9	50.7	17.6	31.7

References:

(1) Akella, J.; Kennedy, G. C. Melting of Three Organic Compounds at High Pressures. *J. Chem. Phys.* **1970**, *52* (2), 970–974. https://doi.org/10.1063/1.1673085.
(2) Vaidya, S. N.; Kennedy, G. C. Compressibility of 18 Molecular Organic Solids to 45 Kbar. *J. Chem. Phys.* **1971**, *55* (3), 987–992. https://doi.org/10.1063/1.1676268.
(3) Fabbiani, F. P. A.; Allan, D. R.; Parsons, S.; Pulham, C. R. Exploration of the High-Pressure Behaviour of Polycyclic Aromatic Hydrocarbons: Naphthalene, Phenanthrene and Pyrene. *Acta Crystallogr. B* **2006**, *62* (5), 826–842. https://doi.org/10.1107/S0108768106026814.
(4) Likhacheva, A. Y.; Rashchenko, S. V.; Chanyshev, A. D.; Inerbaev, T. M.; Litasov, K. D.; Kilin, D. S. Thermal Equation of State of Solid Naphthalene to 13 GPa and 773 K: *In Situ* X-Ray Diffraction Study and First Principles Calculations. *J. Chem. Phys.* **2014**, *140* (16), 164508. https://doi.org/10.1063/1.4871741.
(5) Oddershede, J.; Larsen, S. Charge Density Study of Naphthalene Based on X-Ray Diffraction Data at Four Different Temperatures and Theoretical Calculations. *J. Phys. Chem. A* **2004**, *108* (6), 1057–1063. https://doi.org/10.1021/jp036186g.
(6) Capelli, S. C.; Albinati, A.; Mason, S. A.; Willis, B. T. M. Molecular Motion in Crystalline Naphthalene: Analysis of Multi-Temperature X-Ray and Neutron Diffraction Data. *J. Phys. Chem. A* **2006**, *110* (41), 11695–11703. https://doi.org/10.1021/jp062953a.
(7) Natkaniec, I.; Belushkin, A. V.; Dyck, W.; Fuess, H.; Zeyen, C. M. E. The Structure of Perdeuteronaphthalene C_{10} D_8 at 12 K by Neutron Diffraction. *Z. Für Krist. - Cryst. Mater.* **1983**, *163* (1–4), 285–294. https://doi.org/10.1524/zkri.1983.163.14.285.
(8) Likhacheva, A. Y.; Rashchenko, S. V.; Litasov, K. D. High-Pressure Structural Properties of Naphthalene up to 6 GPa. *J. Appl. Crystallogr.* **2014**, *47* (3), 984–991. https://doi.org/10.1107/S1600576714005937.
(9) Shinozaki, A.; Mimura, K.; Nishida, T.; Inoue, T.; Nakano, S.; Kagi, H. Stability and Partial Oligomerization of Naphthalene under High Pressure at Room Temperature. *Chem. Phys. Lett.* **2016**, *662*, 263–267. https://doi.org/10.1016/j.cplett.2016.09.042.
(10) Xiao, L.-P.; Zhong, G.-H.; Zeng, Z.; Chen, X.-J. Theoretical Study on Structural and Electronic Properties of Solid Anthracene under High Pressure by Density Functional Theory. *Mol. Phys.* **2016**, *114* (2), 283–289. https://doi.org/10.1080/00268976.2015.1099753.
(11) Litasov, K. D.; Inerbaev, T. M.; Abuova, F. U.; Chanyshev, A. D.; Dauletbekova, A. K.; Akilbekov, A. T. High-Pressure Elastic Properties of Polycyclic Aromatic Hydrocarbons Obtained by First-Principles Calculations. *Geochem. Int.* **2019**, *57* (5), 499–508. https://doi.org/10.1134/S0016702919050069.
(12) Chaplot, S. L.; Lehner, N. t; Pawley, G. S. The Structure of Anthracene-D10 at 16 K Using Neutron Diffraction. *Acta Crystallogr. B* **1982**, *38* (2), 483–487.
(13) Zhuravlev, Yu. N.; Fedorov, I. A.; Kiyamov, M. Yu. First-Principles Study of the Crystal Structure and Equation of State of Naphthaline and Anthracene. *J. Struct. Chem.* **2012**, *53* (3), 417–423. https://doi.org/10.1134/S0022476612030018.
(14) Lehmann, M. S.; Pawley, G. S.; Haaland, A.; Øye, H. A.; Svensson, S. The Structure of Perdeuterioanthracene by Neutron Diffraction. *Acta Chem. Scand.* **1972**, *26*, 1996–2004. https://doi.org/10.3891/acta.chem.scand.26-1996.

(15) Léger, J. M.; Aloualiti, H. X-Ray Study of Anthracene under High Pressure. *Solid State Commun.* **1991**, *79* (11), 901–904. https://doi.org/10.1016/0038-1098(91)90440-7.
(16) Oehzelt, M.; Heimel, G.; Resel, R.; Puschnig, P.; Hummer, K.; Ambrosch-Draxl, C.; Takemura, K.; Nakayama, A. High Pressure X-Ray Study on Anthracene. *J. Chem. Phys.* **2003**, *119* (2), 1078–1084. https://doi.org/10.1063/1.1578994.
