# Supplementary material for: Compressional Behavior of Naphthalene (C10H8) and Anthracene (C14H10) up to 50 GPa
Source: ACS Omega. 2025 Oct 19;10(42):50230–42. doi: 10.1021/acsomega.5c06935 (PMC12573047; doi:10.1021/acsomega.5c06935)

checkCIF/PLATON report

Structure factors have been supplied for datablock(s) Naphthalene_00.0GPa_IAM, Naphthalene_06.2GPa_IAM, Naphthalene_09.9GPa_IAM, Naphthalene_10.8GPa_IAM, Naphthalene_14.8GPa_IAM, Naphthalene_20.3GPa_IAM, Naphthalene_24.5GPa_IAM, Naphthalene_28.5GPa_IAM, Naphthalene_35.1GPa_IAM, Naphthalene_43.3GPa_IAM, Naphthalene_46.6GPa_IAM, Naphthalene_50.7GPa_IAM

THIS REPORT IS FOR GUIDANCE ONLY. IF USED AS PART OF A REVIEW PROCEDURE FOR PUBLICATION, IT SHOULD NOT REPLACE THE EXPERTISE OF AN EXPERIENCED CRYSTALLOGRAPHIC REFEREE.

No syntax errors found. CIF dictionary Interpreting this report

Datablock: Naphthalene_00.0GPa_IAM

```
Bond precision:   C-C = 0.0055 A          Wavelength=0.37380

Cell:               a=8.147(6)      b=6.0035(8)         c=8.293(3)
                    alpha=90        beta=116.08(7)      gamma=90
Temperature:        293 K

                 Calculated                 Reported
Volume           364.3(4)                   364.3(4)
Space group      P 21/c                     P 1 21/c 1
Hall group       -P 2ybc                    -P 2ybc
Moiety formula   C10 H8                     C10 H8
Sum formula      C10 H8                     C10 H8
Mr               128.16                     128.16
Dx,g cm-3        1.168                      1.168
Z                2                          2
Mu (mm-1)        0.029                      0.029
F000             136.0                      136.0
F000'            135.97
h,k,lmax         15,11,15                   8,11,11
Nref             2633                       589
Tmin,Tmax        1.000,1.000                0.370,1.000
Tmin'            1.000
```

Correction method= # Reported T Limits: Tmin=0.370 Tmax=1.000
AbsCorr = MULTI-SCAN

Data completeness= 0.224 Theta(max)= 20.844

R(reflections)= 0.0813(190) wR2(reflections)= 0.2489(589)

S = 0.858 Npar= 46

The following ALERTS were generated. Each ALERT has the format
test-name_ALERT_alert-type_alert-level.
Click on the hyperlinks for more details of the test.

Alert level A

PLAT029_ALERT_3_A _diffrn_measured_fraction_theta_full value Low . 0.376 Why?

Author Response: This measurement was performed at high pressure which, due to the high

---

Alert level B

PLAT026_ALERT_3_B Ratio Observed / Unique Reflections (too) Low .. 32% Check

Author Response: The max resolution of measurement is 0.63 A. The diffraction signal of

PLAT149_ALERT_3_B s.u. on the beta Angle is Too Large .... 0.07 Degree

Author Response: Large errors due to low completeness.

Alert level C

PLAT340_ALERT_3_C Low Bond Precision on C-C Bonds .... 0.0055 Ang.

Alert level G

```
ABSMU01_ALERT_1_G  Calculation of _exptl_absorpt_correction_mu
                   not performed for this radiation type.
PLAT012_ALERT_1_G N.O.K.   _shelx_res_checksum Found in CIF ......     Please Check
PLAT199_ALERT_1_G Reported _cell_measurement_temperature ..... (K)        293 Check
PLAT200_ALERT_1_G Reported   _diffrn_ambient_temperature ..... (K)        293 Check
PLAT802_ALERT_4_G CIF Input Record(s) with more than 80 Characters          3 Info
PLAT883_ALERT_1_G Absent Datum for _atom_sites_solution_primary ..     Please Do !
PLAT933_ALERT_2_G Number of HKL-OMIT Records in Embedded .res File          2 Note
                -3   6   2,   -1   3   4,
PLAT941_ALERT_3_G Average HKL Measurement Multiplicity ..........         1.5 Low
PLAT950_ALERT_5_G Calculated (ThMax) and CIF-Reported Hmax Differ           7 Units
PLAT952_ALERT_5_G Calculated (ThMax) and CIF-Reported Lmax Differ.          4 Units
```

1 **ALERT level A** = Most likely a serious problem - resolve or explain
2 **ALERT level B** = A potentially serious problem, consider carefully
1 **ALERT level C** = Check. Ensure it is not caused by an omission or oversight
10 **ALERT level G** = General information/check it is not something unexpected

5 ALERT type 1 CIF construction/syntax error, inconsistent or missing data
1 ALERT type 2 Indicator that the structure model may be wrong or deficient
5 ALERT type 3 Indicator that the structure quality may be low
1 ALERT type 4 Improvement, methodology, query or suggestion
2 ALERT type 5 Informative message, check

Datablock: Naphthalene_06.2GPa_IAM

Bond precision: C-C = 0.0070 A Wavelength=0.37380

Cell:	a=7.168(7)	b=5.5599(6)	c=7.320(3)
	alpha=90	beta=111.07(8)	gamma=90
Temperature:	293 K		

	Calculated	Reported
Volume	272.2(3)	272.2(3)
Space group	P 21/c	P 1 21/c 1
Hall group	-P 2ybc	-P 2ybc
Moiety formula	C10 H8	C10 H8
Sum formula	C10 H8	C10 H8
Mr	128.16	128.18
Dx,g cm-3	1.564	1.564
Z	2	2
Mu (mm-1)	0.039	0.038
F000	136.0	136.0
F000’	135.97	
h,k,lmax	11,8,11	5,8,10
Nref	1133	308
Tmin,Tmax	1.000,1.000	0.372,1.000
Tmin’	1.000	

Correction method= # Reported T Limits: Tmin=0.372 Tmax=1.000
AbsCorr = MULTI-SCAN

Data completeness= 0.272 Theta(max)= 17.230

R(reflections)= 0.0666(177) wR2(reflections)= 0.1910(308)

S = 0.938 Npar= 46

The following ALERTS were generated. Each ALERT has the format
test-name_ALERT_alert-type_alert-level.
Click on the hyperlinks for more details of the test.

Alert level A

PLAT029_ALERT_3_A _diffrn_measured_fraction_theta_full value Low . 0.298 Why?

Author Response: This measurement was performed at high pressure which, due to the high

Alert level B

PLAT088_ALERT_3_B Poor Data / Parameter Ratio .... 6.70 Note

Author Response: This measurement was performed at high pressure which, due to the high

PLAT149_ALERT_3_B s.u. on the beta Angle is Too Large .... 0.08 Degree

Author Response: Large errors due to low completeness.

```
PLAT910_ALERT_3_B Missing FCF Reflection(s) Below Theta(Min)[Deg]=       4.16 Note
                1  0  0,   2  0  0,   1  1  0,   2  1  0,   0  2  0,  -2  1  1,
               -1  1  1,   0  1  1,   1  1  1,  -2  0  2,  -1  0  2,   0  0  2,
                1  0  2,  -2  1  2,  -1  1  2,   0  1  2,
```

Author Response: This measurement was performed at high pressure which, due to the high

```
PLAT911_ALERT_3_B Missing FCF Refl Between Thmin & STh/L=    0.600        248 Report
                3  0  0,   4  0  0,   5  0  0,   3  1  0,   4  1  0,   5  1  0,
                1  2  0,   2  2  0,   3  2  0,   4  2  0,   5  2  0,   2  3  0,
                3  3  0,   4  3  0,   5  3  0,   3  4  0,   4  4  0,   5  4  0,
                5  5  0,  -5  1  1,  -4  1  1,  -3  1  1,   2  1  1,   3  1  1,
                4  1  1,   5  1  1,  -5  2  1,  -4  2  1,  -3  2  1,  -2  2  1,
                   (  218 More Missing: see the .ckf listing file)
```

Author Response: This measurement was performed at high pressure which, due to the high

Alert level C

```
PLAT340_ALERT_3_C Low Bond Precision on  C-C Bonds ...............      0.007 Ang.
PLAT350_ALERT_3_C Short  C-H (X0.96,N1.08A)  C1        - H1       .      0.93 Ang.
PLAT350_ALERT_3_C Short  C-H (X0.96,N1.08A)  C2        - H2       .      0.93 Ang.
PLAT350_ALERT_3_C Short  C-H (X0.96,N1.08A)  C3        - H3       .      0.93 Ang.
PLAT350_ALERT_3_C Short  C-H (X0.96,N1.08A)  C4        - H4       .      0.93 Ang.
PLAT411_ALERT_2_C Short Inter H...H Contact  H3        ..H4       .      2.12 Ang.
                                             -x,1/2+y,1/2-z  =      2_555 Check
PLAT913_ALERT_3_C Missing # of Very Strong Reflections in FCF ....         10 Note
```

```
                1  0  0,   3  0  0,   0  2  0,  -1  1  1,  -2  0  2,  -1  0  2,
                0  0  2,  -2  1  2,  -1  1  2,  -2  1  3,
```

Alert level G

```
ABSMU01_ALERT_1_G  Calculation of _exptl_absorpt_correction_mu
                not performed for this radiation type.
PLAT072_ALERT_2_G SHELXL First  Parameter in WGHT  Unusually Large       0.13 Report
PLAT073_ALERT_1_G H-atoms ref., but hydrogen treatment Reported as     constr Check
PLAT199_ALERT_1_G Reported _cell_measurement_temperature ..... (K)        293 Check
PLAT200_ALERT_1_G Reported   _diffrn_ambient_temperature ..... (K)        293 Check
PLAT432_ALERT_2_G Short Inter X...Y Contact  C1       ..C5       .       3.03 Ang.
                                            1-x,1/2+y,1/2-z  =      2_655 Check
PLAT432_ALERT_2_G Short Inter X...Y Contact  C1       ..C5       .       3.18 Ang.
                                             x,3/2-y,-1/2+z  =      4_575 Check
PLAT802_ALERT_4_G CIF Input Record(s) with more than 80 Characters          5 Info
PLAT912_ALERT_4_G Missing # of FCF Reflections Above STh/L=  0.600        176 Note
PLAT933_ALERT_2_G Number of HKL-OMIT Records in Embedded .res File         16 Note
               -1  4  9,   0  0  2,   0  1  1,   0  1  2,   0  3  6,   0  3  7,
                0  3  8,   0  8  4,   1  7  4,   2  1  1,   2  1  2,   2  2  1,
                3  3  4,   3  4  7,   3  7  2,   4  1  3,
PLAT956_ALERT_1_G Calculated (ThMax) and Actual (FCF) Hmax Differ           6 Units
PLAT969_ALERT_5_G The 'Henn et al.' R-Factor-gap value ..........      10.302 Note
              Predicted wR2: Based on SigI**2  1.85 or SHELX Weight 20.32
PLAT978_ALERT_2_G Number C-C Bonds with Positive Residual Density.          2 Info
PLAT979_ALERT_1_G NoSpherA2 Scattering Factors Used .............      Please Note
```

```
   1 ALERT level A = Most likely a serious problem - resolve or explain
   4 ALERT level B = A potentially serious problem, consider carefully
   7 ALERT level C = Check. Ensure it is not caused by an omission or oversight
  14 ALERT level G = General information/check it is not something unexpected

   6 ALERT type 1 CIF construction/syntax error, inconsistent or missing data
   6 ALERT type 2 Indicator that the structure model may be wrong or deficient
  11 ALERT type 3 Indicator that the structure quality may be low
   2 ALERT type 4 Improvement, methodology, query or suggestion
   1 ALERT type 5 Informative message, check
```

Datablock: Naphthalene_09.9GPa_IAM

```
Bond precision:   C-C = 0.0063 A            Wavelength=0.37380

Cell:               a=6.790(8)      b=5.3738(8)          c=6.959(3)
                    alpha=90        beta=109.22(9)       gamma=90
Temperature:        293 K
```

	Calculated	Reported
Volume	239.8(3)	239.8(3)
Space group	P 21/c	P 1 21/c 1
Hall group	-P 2ybc	-P 2ybc
Moiety formula	C10 H8	C10 H8
Sum formula	C10 H8	C10 H8
Mr	128.16	128.18
Dx,g cm-3	1.775	1.775
Z	2	2
Mu (mm-1)	0.044	0.043
F000	136.0	136.0
F000'	135.97	
h,k,lmax	10,8,11	5,8,10
Nref	1004	290
Tmin,Tmax	0.999,0.999	0.229,1.000
Tmin'	0.999	

Correction method= # Reported T Limits: Tmin=0.229 Tmax=1.000
AbsCorr = MULTI-SCAN

Data completeness= 0.289 Theta(max)= 17.240

R(reflections)= 0.0768(210) wR2(reflections)= 0.2102(290)

S = 0.995 Npar= 46

The following ALERTS were generated. Each ALERT has the format
test-name_ALERT_alert-type_alert-level.
Click on the hyperlinks for more details of the test.

Alert level A

PLAT029_ALERT_3_A _diffrn_measured_fraction_theta_full value Low . 0.341 Why?

Author Response: This measurement was performed at high pressure which, due to the high

---

Alert level B

PLAT088_ALERT_3_B Poor Data / Parameter Ratio .... 6.30 Note

Author Response: This measurement was performed at high pressure which, due to the high

```
PLAT149_ALERT_3_B s.u. on the   beta    Angle is Too Large .......       0.09 Degree
```

Author Response: Large errors due to low completeness.

```
PLAT411_ALERT_2_B Short Inter H...H Contact  H3       ..H4       .       1.95 Ang.
                                             -x,1/2+y,1/2-z  =      2_555 Check
```

Author Response: H atoms are close to each other at high pressure.

```
PLAT911_ALERT_3_B Missing FCF Refl Between Thmin & STh/L=    0.600        225 Report
                2  0  0,   3  0  0,   4  0  0,   5  0  0,   2  1  0,   3  1  0,
                4  1  0,   5  1  0,   0  2  0,   3  2  0,   4  2  0,   5  2  0,
                2  3  0,   3  3  0,   4  3  0,   5  3  0,   4  4  0,   5  4  0,
                4  5  0,  -5  1  1,  -4  1  1,  -3  1  1,  -2  1  1,  -1  1  1,
                3  1  1,   4  1  1,   5  1  1,  -5  2  1,  -4  2  1,  -3  2  1,
                   (  195 More Missing: see the .ckf listing file)
```

Author Response: This measurement was performed at high pressure which, due to the high

---

Alert level C

```
PLAT148_ALERT_3_C s.u. on the      a    - Axis is (Too) Large ....      0.008 Ang.
PLAT340_ALERT_3_C Low Bond Precision on  C-C Bonds ...............    0.00633 Ang.
PLAT350_ALERT_3_C Short  C-H (X0.96,N1.08A)  C1       - H1       .       0.93 Ang.
PLAT350_ALERT_3_C Short  C-H (X0.96,N1.08A)  C2       - H2       .       0.93 Ang.
PLAT350_ALERT_3_C Short  C-H (X0.96,N1.08A)  C3       - H3       .       0.93 Ang.
PLAT350_ALERT_3_C Short  C-H (X0.96,N1.08A)  C4       - H4       .       0.93 Ang.
PLAT913_ALERT_3_C Missing # of Very Strong Reflections in FCF ....          8 Note
                1  0  0,   3  0  0,  -1  1  1,  -2  0  2,  -1  0  2,  -2  1  2,
               -1  1  2,  -3  1  4,
PLAT939_ALERT_3_C Large Value of Not (SHELXL) Weight Optimized S .      12.58 Check
```

Alert level G

```
ABSMU01_ALERT_1_G  Calculation of _exptl_absorpt_correction_mu
                not performed for this radiation type.
PLAT003_ALERT_2_G Number of Uiso or U(i,j)  Restrained non-H-Atoms          1 Report
PLAT072_ALERT_2_G SHELXL First  Parameter in WGHT  Unusually Large       0.17 Report
PLAT073_ALERT_1_G H-atoms ref., but hydrogen treatment Reported as     constr Check
PLAT186_ALERT_4_G The CIF-Embedded .res File Contains ISOR Records          1 Report
PLAT199_ALERT_1_G Reported _cell_measurement_temperature ..... (K)        293 Check
PLAT200_ALERT_1_G Reported   _diffrn_ambient_temperature ..... (K)        293 Check
PLAT432_ALERT_2_G Short Inter X...Y Contact  C1       ..C5       .       2.90 Ang.
                                            1-x,1/2+y,1/2-z  =      2_655 Check
PLAT432_ALERT_2_G Short Inter X...Y Contact  C1       ..C5       .       3.02 Ang.
                                             x,3/2-y,-1/2+z  =      4_575 Check
PLAT432_ALERT_2_G Short Inter X...Y Contact  C1       ..C4       .       3.07 Ang.
                                            1-x,1/2+y,1/2-z  =      2_655 Check
PLAT432_ALERT_2_G Short Inter X...Y Contact  C1       ..C4       .       3.12 Ang.
                                                   x,1+y,z  =      1_565 Check
PLAT432_ALERT_2_G Short Inter X...Y Contact  C1       ..C1       .       3.18 Ang.
```

```
                                             1-x,2-y,1-z  =      3_676 Check
PLAT432_ALERT_2_G Short Inter X...Y Contact  C2       ..C3       .       3.06 Ang.
                                          -x,1/2+y,1/2-z  =      2_555 Check
PLAT432_ALERT_2_G Short Inter X...Y Contact  C2       ..C5       .       3.17 Ang.
                                         1-x,1/2+y,1/2-z  =      2_655 Check
PLAT802_ALERT_4_G CIF Input Record(s) with more than 80 Characters          4 Info
PLAT860_ALERT_3_G Number of Least-Squares Restraints ............           6 Note
PLAT910_ALERT_3_G Missing FCF Reflection(s) Below Theta(Min)[Deg]=       2.60 Note
                1  0  0,   0  1  1,
PLAT912_ALERT_4_G Missing # of FCF Reflections Above STh/L=  0.600        184 Note
PLAT933_ALERT_2_G Number of HKL-OMIT Records in Embedded .res File          4 Note
               -3  7  1,   0  1 10,   1  8  1,   3  7  2,
PLAT956_ALERT_1_G Calculated (ThMax) and Actual (FCF) Hmax Differ           5 Units
PLAT969_ALERT_5_G The 'Henn et al.' R-Factor-gap value ..........      14.368 Note
              Predicted wR2: Based on SigI**2  1.46 or SHELX Weight 21.08
PLAT978_ALERT_2_G Number C-C Bonds with Positive Residual Density.          4 Info
PLAT979_ALERT_1_G NoSpherA2 Scattering Factors Used .............     Please Note
```

```
   1 ALERT level A = Most likely a serious problem - resolve or explain
   4 ALERT level B = A potentially serious problem, consider carefully
   8 ALERT level C = Check. Ensure it is not caused by an omission or oversight
  23 ALERT level G = General information/check it is not something unexpected

   6 ALERT type 1 CIF construction/syntax error, inconsistent or missing data
  12 ALERT type 2 Indicator that the structure model may be wrong or deficient
  14 ALERT type 3 Indicator that the structure quality may be low
   3 ALERT type 4 Improvement, methodology, query or suggestion
   1 ALERT type 5 Informative message, check
```

Datablock: Naphthalene_10.8GPa_IAM

```
Bond precision:   C-C = 0.0072 A           Wavelength=0.37380

Cell:              a=6.779(14)     b=5.3433(8)          c=6.901(3)
                   alpha=90        beta=109.04(13)      gamma=90
Temperature:       293 K
```

	Calculated	Reported
Volume	236.3(5)	236.3(5)
Space group	P 21/c	P 1 21/c 1
Hall group	-P 2ybc	-P 2ybc
Moiety formula	C10 H8	C10 H8
Sum formula	C10 H8	C10 H8
Mr	128.16	128.18
Dx,g cm-3	1.801	1.802
Z	2	2
Mu (mm-1)	0.045	0.044
F000	136.0	136.0
F000'	135.97	
h,k,lmax	13,10,13	5,10,12
Nref	1806	324
Tmin,Tmax	0.999,0.999	0.393,1.000
Tmin'	0.999	

Correction method= # Reported T Limits: Tmin=0.393 Tmax=1.000
AbsCorr = MULTI-SCAN

Data completeness= 0.179 Theta(max)= 21.240

R(reflections)= 0.0889(209) wR2(reflections)= 0.2233(324)

S = 0.927 Npar= 46

The following ALERTS were generated. Each ALERT has the format
test-name_ALERT_alert-type_alert-level.
Click on the hyperlinks for more details of the test.

Alert level A

PLAT029_ALERT_3_A _diffrn_measured_fraction_theta_full value Low . 0.301 Why?

Author Response: This measurement was performed at high pressure which, due to the high

---

Alert level B

PLAT088_ALERT_3_B Poor Data / Parameter Ratio .... 7.04 Note

Author Response: This measurement was performed at high pressure which, due to the high

```
PLAT149_ALERT_3_B s.u. on the   beta    Angle is Too Large .......       0.13 Degree
```

Author Response: Large errors due to low completeness.

```
PLAT411_ALERT_2_B Short Inter H...H Contact  H3        ..H4        .        1.94 Ang.
                                             -x,1/2+y,1/2-z  =      2_555 Check
```

Author Response: H atoms are close to each other at high pressure.

```
PLAT911_ALERT_3_B Missing FCF Refl Between Thmin & STh/L=    0.600        242 Report
                 2   0   0,    3   0   0,    4   0   0,    5   0   0,    2   1   0,    3   1   0,
                 4   1   0,    5   1   0,    2   2   0,    3   2   0,    4   2   0,    5   2   0,
                 2   3   0,    3   3   0,    4   3   0,    5   3   0,    4   4   0,    5   4   0,
                 4   5   0,   -5   1   1,   -4   1   1,   -3   1   1,   -2   1   1,   -1   1   1,
                 3   1   1,    4   1   1,    5   1   1,   -5   2   1,   -4   2   1,   -3   2   1,
                    (  212 More Missing: see the .ckf listing file)
```

Author Response: This measurement was performed at high pressure which, due to the high

---

Alert level C

```
PLAT148_ALERT_3_C s.u. on the      a    - Axis is (Too) Large ....      0.014 Ang.
PLAT340_ALERT_3_C Low Bond Precision on  C-C Bonds ...............    0.00717 Ang.
PLAT350_ALERT_3_C Short  C-H (X0.96,N1.08A)  C1         - H1       .      0.93 Ang.
PLAT350_ALERT_3_C Short  C-H (X0.96,N1.08A)  C2         - H2       .      0.93 Ang.
PLAT350_ALERT_3_C Short  C-H (X0.96,N1.08A)  C3         - H3       .      0.93 Ang.
PLAT350_ALERT_3_C Short  C-H (X0.96,N1.08A)  C4         - H4       .      0.93 Ang.
PLAT411_ALERT_2_C Short Inter H...H Contact  H3        ..H3        .        2.11 Ang.
                                                  -x,1-y,-z  =      3_565 Check
```

Author Response: H atoms are close to each other at high pressure.

```
PLAT913_ALERT_3_C Missing # of Very Strong Reflections in FCF ....            8 Note
                 1   0   0,    3   0   0,   -1   1   1,   -2   0   2,   -1   0   2,   -2   1   2,
                -1   1   2,    1   0   6,
PLAT939_ALERT_3_C Large Value of Not (SHELXL) Weight Optimized S .      14.50 Check
```

Alert level G

```
ABSMU01_ALERT_1_G  Calculation of _exptl_absorpt_correction_mu
                 not performed for this radiation type.
PLAT072_ALERT_2_G SHELXL First  Parameter in WGHT  Unusually Large       0.18 Report
PLAT073_ALERT_1_G H-atoms ref., but hydrogen treatment Reported as     constr Check
PLAT199_ALERT_1_G Reported _cell_measurement_temperature ..... (K)        293 Check
PLAT200_ALERT_1_G Reported   _diffrn_ambient_temperature ..... (K)        293 Check
PLAT432_ALERT_2_G Short Inter X...Y Contact  C1        ..C5        .        2.85 Ang.
                                            1-x,1/2+y,1/2-z  =      2_655 Check
PLAT432_ALERT_2_G Short Inter X...Y Contact  C1        ..C5        .        2.99 Ang.
                                             x,3/2-y,-1/2+z  =      4_575 Check
PLAT432_ALERT_2_G Short Inter X...Y Contact  C1        ..C4        .        3.04 Ang.
```

```
                                         1-x,1/2+y,1/2-z  =      2_655 Check
PLAT432_ALERT_2_G Short Inter X...Y Contact  C1       ..C4       .       3.12 Ang.
                                                 x,1+y,z  =      1_565 Check
PLAT432_ALERT_2_G Short Inter X...Y Contact  C1       ..C1       .       3.15 Ang.
                                             1-x,2-y,1-z  =      3_676 Check
PLAT432_ALERT_2_G Short Inter X...Y Contact  C2       ..C3       .       3.07 Ang.
                                          -x,1/2+y,1/2-z  =      2_555 Check
PLAT432_ALERT_2_G Short Inter X...Y Contact  C2       ..C5       .       3.12 Ang.
                                         1-x,1/2+y,1/2-z  =      2_655 Check
PLAT432_ALERT_2_G Short Inter X...Y Contact  C3       ..C4       .       3.17 Ang.
                                          x,1/2-y,-1/2+z  =      4_565 Check
PLAT802_ALERT_4_G CIF Input Record(s) with more than 80 Characters          4 Info
PLAT910_ALERT_3_G Missing FCF Reflection(s) Below Theta(Min)[Deg]=       2.61 Note
                1  0  0,   0  1  1,
PLAT912_ALERT_4_G Missing # of FCF Reflections Above STh/L=  0.600        492 Note
PLAT933_ALERT_2_G Number of HKL-OMIT Records in Embedded .res File          7 Note
                0  7  1,   0  7  5,   0  8  4,   1  5  4,   1  9  3,   1  9  4,
                4  3  4,
PLAT956_ALERT_1_G Calculated (ThMax) and Actual (FCF) Hmax Differ           8 Units
PLAT969_ALERT_5_G The 'Henn et al.' R-Factor-gap value ..........      16.159 Note
              Predicted wR2: Based on SigI**2  1.38 or SHELX Weight 24.04
PLAT978_ALERT_2_G Number C-C Bonds with Positive Residual Density.          3 Info
PLAT979_ALERT_1_G NoSpherA2 Scattering Factors Used .............      Please Note
```

```
   1 ALERT level A = Most likely a serious problem - resolve or explain
   4 ALERT level B = A potentially serious problem, consider carefully
   9 ALERT level C = Check. Ensure it is not caused by an omission or oversight
  21 ALERT level G = General information/check it is not something unexpected

   6 ALERT type 1 CIF construction/syntax error, inconsistent or missing data
  13 ALERT type 2 Indicator that the structure model may be wrong or deficient
  13 ALERT type 3 Indicator that the structure quality may be low
   2 ALERT type 4 Improvement, methodology, query or suggestion
   1 ALERT type 5 Informative message, check
```

Datablock: Naphthalene_14.8GPa_IAM

```
Bond precision:   C-C = 0.0098 A           Wavelength=0.37380

Cell:              a=6.622(11)      b=5.2755(7)          c=6.771(2)
                   alpha=90         beta=107.96(9)       gamma=90
Temperature:       293 K
```

	Calculated	Reported
Volume	225.0(4)	225.0(4)
Space group	P 21/c	P 1 21/c 1
Hall group	-P 2ybc	-P 2ybc
Moiety formula	C10 H8	C10 H8
Sum formula	C10 H8	C10 H8
Mr	128.16	128.18
Dx,g cm-3	1.892	1.892
Z	2	2
Mu (mm-1)	0.047	0.046
F000	136.0	136.0
F000'	135.97	
h,k,lmax	10,8,10	4,8,10
Nref	944	281
Tmin,Tmax	0.999,0.999	0.140,1.000
Tmin'	0.999	

Correction method= # Reported T Limits: Tmin=0.140 Tmax=1.000
AbsCorr = MULTI-SCAN

Data completeness= 0.298 Theta(max)= 17.250

R(reflections)= 0.0978(167) wR2(reflections)= 0.2562(281)

S = 0.946 Npar= 46

The following ALERTS were generated. Each ALERT has the format
test-name_ALERT_alert-type_alert-level.
Click on the hyperlinks for more details of the test.

Alert level A

PLAT029_ALERT_3_A _diffrn_measured_fraction_theta_full value Low . 0.327 Why?

Author Response: This measurement was performed at high pressure which, due to the high

---

Alert level B

PLAT088_ALERT_3_B Poor Data / Parameter Ratio .... 6.11 Note

Author Response: This measurement was performed at high pressure which, due to the high

```
PLAT149_ALERT_3_B s.u. on the   beta    Angle is Too Large .......       0.09 Degree
```

Author Response: Large errors due to low completeness.

```
PLAT411_ALERT_2_B Short Inter H...H Contact  H3        ..H4        .       1.88 Ang.
                                             -x,1/2+y,1/2-z  =      2_555 Check
```

Author Response: H atoms are close to each other at high pressure.

```
PLAT911_ALERT_3_B Missing FCF Refl Between Thmin & STh/L=    0.600        170 Report
                2  0  0,   3  0  0,   4  0  0,   2  1  0,   3  1  0,   4  1  0,
                0  2  0,   1  2  0,   2  2  0,   3  2  0,   4  2  0,   2  3  0,
                3  3  0,   4  3  0,   4  4  0,   4  5  0,   0  6  0,  -4  1  1,
               -3  1  1,  -2  1  1,   2  1  1,   3  1  1,   4  1  1,  -4  2  1,
               -3  2  1,  -2  2  1,  -1  2  1,   0  2  1,   3  2  1,   4  2  1,
                   (  140 More Missing: see the .ckf listing file)
```

Author Response: This measurement was performed at high pressure which, due to the high

---

Alert level C

```
PLAT084_ALERT_3_C High wR2 Value (i.e. > 0.25) ...................       0.26 Report
PLAT148_ALERT_3_C s.u. on the      a    - Axis is (Too) Large ....      0.011 Ang.
PLAT340_ALERT_3_C Low Bond Precision on  C-C Bonds ...............    0.00983 Ang.
PLAT350_ALERT_3_C Short  C-H (X0.96,N1.08A)  C1        - H1        .       0.93 Ang.
PLAT350_ALERT_3_C Short  C-H (X0.96,N1.08A)  C2        - H2        .       0.93 Ang.
PLAT350_ALERT_3_C Short  C-H (X0.96,N1.08A)  C3        - H3        .       0.93 Ang.
PLAT350_ALERT_3_C Short  C-H (X0.96,N1.08A)  C4        - H4        .       0.93 Ang.
PLAT411_ALERT_2_C Short Inter H...H Contact  H2        ..H3        .       2.15 Ang.
                                                  -x,1-y,-z  =      3_565 Check
```

Author Response: H atoms are close to each other at high pressure.

```
PLAT411_ALERT_2_C Short Inter H...H Contact  H3        ..H3        .       2.09 Ang.
                                                  -x,1-y,-z  =      3_565 Check
```

Author Response: H atoms are close to each other at high pressure.

```
PLAT910_ALERT_3_C Missing FCF Reflection(s) Below Theta(Min)[Deg]=       3.40 Note
                1  0  0,   1  1  0,  -1  1  1,   0  1  1,  -1  0  2,   0  0  2,
PLAT913_ALERT_3_C Missing # of Very Strong Reflections in FCF ....         12 Note
                1  0  0,   3  0  0,   0  2  0,  -1  1  1,   2  1  1,  -2  0  2,
               -1  0  2,   0  0  2,  -2  1  2,  -1  1  2,  -2  1  3,  -3  1  4,
PLAT939_ALERT_3_C Large Value of Not (SHELXL) Weight Optimized S .      10.87 Check
```

Alert level G

```
ABSMU01_ALERT_1_G  Calculation of _exptl_absorpt_correction_mu
                not performed for this radiation type.
```

```
PLAT072_ALERT_2_G SHELXL First  Parameter in WGHT  Unusually Large       0.20 Report
PLAT073_ALERT_1_G H-atoms ref., but hydrogen treatment Reported as     constr Check
PLAT199_ALERT_1_G Reported _cell_measurement_temperature ..... (K)        293 Check
PLAT200_ALERT_1_G Reported   _diffrn_ambient_temperature ..... (K)        293 Check
PLAT432_ALERT_2_G Short Inter X...Y Contact  C1       ..C5       .       2.81 Ang.
                                          1-x,1/2+y,1/2-z  =     2_655 Check
PLAT432_ALERT_2_G Short Inter X...Y Contact  C1       ..C5       .       2.92 Ang.
                                           x,3/2-y,-1/2+z  =     4_575 Check
PLAT432_ALERT_2_G Short Inter X...Y Contact  C1       ..C4       .       2.97 Ang.
                                          1-x,1/2+y,1/2-z  =     2_655 Check
PLAT432_ALERT_2_G Short Inter X...Y Contact  C1       ..C4       .       3.02 Ang.
                                                  x,1+y,z  =     1_565 Check
PLAT432_ALERT_2_G Short Inter X...Y Contact  C1       ..C1       .       3.07 Ang.
                                              1-x,2-y,1-z  =     3_676 Check
PLAT432_ALERT_2_G Short Inter X...Y Contact  C1       ..C2       .       3.15 Ang.
                                            x,3/2-y,1/2+z  =     4_576 Check
PLAT432_ALERT_2_G Short Inter X...Y Contact  C2       ..C3       .       2.93 Ang.
                                           -x,1/2+y,1/2-z  =     2_555 Check
PLAT432_ALERT_2_G Short Inter X...Y Contact  C2       ..C5       .       3.08 Ang.
                                          1-x,1/2+y,1/2-z  =     2_655 Check
PLAT432_ALERT_2_G Short Inter X...Y Contact  C2       ..C4       .       3.12 Ang.
                                           -x,1/2+y,1/2-z  =     2_555 Check
PLAT432_ALERT_2_G Short Inter X...Y Contact  C2       ..C5       .       3.19 Ang.
                                           x,3/2-y,-1/2+z  =     4_575 Check
PLAT432_ALERT_2_G Short Inter X...Y Contact  C3       ..C4       .       3.10 Ang.
                                           x,1/2-y,-1/2+z  =     4_565 Check
PLAT432_ALERT_2_G Short Inter X...Y Contact  C3       ..C4       .       3.14 Ang.
                                           -x,1/2+y,1/2-z  =     2_555 Check
PLAT432_ALERT_2_G Short Inter X...Y Contact  C3       ..C3       .       3.17 Ang.
                                                -x,1-y,-z  =     3_565 Check
PLAT802_ALERT_4_G CIF Input Record(s) with more than 80 Characters          4 Info
PLAT912_ALERT_4_G Missing # of FCF Reflections Above STh/L=  0.600        111 Note
PLAT933_ALERT_2_G Number of HKL-OMIT Records in Embedded .res File         14 Note
                0  0  2,   0  1  1,   0  1  2,   0  1  3,   0  2  0,   0  2  1,
                0  2  3,   0  3  8,   2  2  6,   2  2  7,   2  2  8,   3  6  3,
                4  3  7,   4  6  1,
PLAT956_ALERT_1_G Calculated (ThMax) and Actual (FCF) Hmax Differ           6 Units
PLAT969_ALERT_5_G The 'Henn et al.' R-Factor-gap value ..........      11.499 Note
              Predicted wR2: Based on SigI**2  2.23 or SHELX Weight 27.03
PLAT978_ALERT_2_G Number C-C Bonds with Positive Residual Density.          1 Info
PLAT979_ALERT_1_G NoSpherA2 Scattering Factors Used .............      Please Note
```

```
   1 ALERT level A = Most likely a serious problem - resolve or explain
   4 ALERT level B = A potentially serious problem, consider carefully
  12 ALERT level C = Check. Ensure it is not caused by an omission or oversight
  25 ALERT level G = General information/check it is not something unexpected

   6 ALERT type 1 CIF construction/syntax error, inconsistent or missing data
  19 ALERT type 2 Indicator that the structure model may be wrong or deficient
  14 ALERT type 3 Indicator that the structure quality may be low
   2 ALERT type 4 Improvement, methodology, query or suggestion
   1 ALERT type 5 Informative message, check
```

Datablock: Naphthalene_20.3GPa_IAM

Bond precision: C-C = 0.0063 A Wavelength=0.37380

Cell:	a=6.447(11)	b=5.1951(7)	c=6.6135(18)
	alpha=90	beta=106.64(8)	gamma=90
Temperature:	293 K		

	Calculated	Reported
Volume	212.2(4)	212.2(4)
Space group	P 21/c	P 1 21/c 1
Hall group	-P 2ybc	-P 2ybc
Moiety formula	C10 H8	C10 H8
Sum formula	C10 H8	C10 H8
Mr	128.16	128.18
Dx,g cm-3	2.006	2.006
Z	2	2
Mu (mm-1)	0.050	0.049
F000	136.0	136.0
F000'	135.97	
h,k,lmax	12,9,12	4,9,12
Nref	1530	321
Tmin,Tmax	0.999,0.999	0.034,1.000
Tmin'	0.999	

Correction method= # Reported T Limits: Tmin=0.034 Tmax=1.000
AbsCorr = MULTI-SCAN

Data completeness= 0.210 Theta(max)= 20.840

R(reflections)= 0.0755(221) wR2(reflections)= 0.1865(321)

S = 0.901 Npar= 46

The following ALERTS were generated. Each ALERT has the format
test-name_ALERT_alert-type_alert-level.
Click on the hyperlinks for more details of the test.

Alert level A

PLAT029_ALERT_3_A _diffrn_measured_fraction_theta_full value Low . 0.337 Why?

Author Response: This measurement was performed at high pressure which, due to the high

```
PLAT411_ALERT_2_A Short Inter H...H Contact  H3         ..H4         .       1.78 Ang.
                                             -x,1/2+y,1/2-z  =      2_555 Check
```

Author Response: H atoms are close to each other at high pressure.

Alert level B

```
PLAT088_ALERT_3_B Poor Data / Parameter Ratio ....................       6.98 Note
```

Author Response: This measurement was performed at high pressure which, due to the high

```
PLAT149_ALERT_3_B s.u. on the   beta    Angle is Too Large .......       0.08 Degree
```

Author Response: Large errors due to low completeness.

```
PLAT911_ALERT_3_B Missing FCF Refl Between Thmin & STh/L=    0.600        163 Report
                2   0   0,    3   0   0,    4   0   0,    2   1   0,    3   1   0,    4   1   0,
                0   2   0,    1   2   0,    2   2   0,    3   2   0,    4   2   0,    3   3   0,
                4   3   0,    1   4   0,    3   4   0,    4   4   0,    4   5   0,    0   6   0,
               -4   1   1,   -3   1   1,   -2   1   1,    2   1   1,    3   1   1,    4   1   1,
               -4   2   1,   -3   2   1,   -2   2   1,   -1   2   1,    0   2   1,    3   2   1,
                    (  133 More Missing: see the .ckf listing file)
```

Author Response: This measurement was performed at high pressure which, due to the high

---

Alert level C

```
PLAT148_ALERT_3_C s.u. on the      a    - Axis is (Too) Large ....      0.011 Ang.
PLAT334_ALERT_2_C Small <C-C> Benzene Dist.   C1        -C5_a     .       1.37 Ang.
PLAT334_ALERT_2_C Small <C-C> Benzene Dist.   C5        -C5_a     .       1.37 Ang.
PLAT340_ALERT_3_C Low Bond Precision on  C-C Bonds ...............    0.00633 Ang.
PLAT350_ALERT_3_C Short  C-H (X0.96,N1.08A)  C1         - H1       .       0.93 Ang.
PLAT350_ALERT_3_C Short  C-H (X0.96,N1.08A)  C2         - H2       .       0.93 Ang.
PLAT350_ALERT_3_C Short  C-H (X0.96,N1.08A)  C3         - H3       .       0.93 Ang.
PLAT350_ALERT_3_C Short  C-H (X0.96,N1.08A)  C4         - H4       .       0.93 Ang.
PLAT411_ALERT_2_C Short Inter H...H Contact  H1         ..H4       .       2.10 Ang.
                                                    x,1+y,z  =      1_565 Check
```

Author Response: H atoms are close to each other at high pressure.

```
PLAT411_ALERT_2_C Short Inter H...H Contact  H1         ..H1       .       2.14 Ang.
                                               1-x,2-y,1-z  =      3_676 Check
```

Author Response: H atoms are close to each other at high pressure.

```
PLAT411_ALERT_2_C Short Inter H...H Contact  H2       ..H3       .       2.08 Ang.
                                               -x,1-y,-z  =      3_565 Check
```

Author Response: H atoms are close to each other at high pressure.

```
PLAT411_ALERT_2_C Short Inter H...H Contact  H3       ..H3       .       2.02 Ang.
                                               -x,1-y,-z  =      3_565 Check
```

Author Response: H atoms are close to each other at high pressure.

```
PLAT910_ALERT_3_C Missing FCF Reflection(s) Below Theta(Min)[Deg]=       3.44 Note
                1  0  0,   1  1  0,  -1  1  1,   0  1  1,  -1  0  2,   0  0  2,
PLAT913_ALERT_3_C Missing # of Very Strong Reflections in FCF ....          7 Note
                1  0  0,   3  0  0,  -1  1  1,  -2  0  2,  -1  0  2,  -2  1  2,
               -1  1  2,
```

Alert level G

```
ABSMU01_ALERT_1_G  Calculation of _exptl_absorpt_correction_mu
                not performed for this radiation type.
PLAT072_ALERT_2_G SHELXL First  Parameter in WGHT  Unusually Large       0.15 Report
PLAT073_ALERT_1_G H-atoms ref., but hydrogen treatment Reported as     constr Check
PLAT199_ALERT_1_G Reported _cell_measurement_temperature ..... (K)        293 Check
PLAT200_ALERT_1_G Reported   _diffrn_ambient_temperature ..... (K)        293 Check
PLAT432_ALERT_2_G Short Inter X...Y Contact  C1       ..C5       .       2.72 Ang.
                                         1-x,1/2+y,1/2-z  =      2_655 Check
PLAT432_ALERT_2_G Short Inter X...Y Contact  C1       ..C5       .       2.86 Ang.
                                          x,3/2-y,-1/2+z  =      4_575 Check
PLAT432_ALERT_2_G Short Inter X...Y Contact  C1       ..C4       .       2.89 Ang.
                                         1-x,1/2+y,1/2-z  =      2_655 Check
PLAT432_ALERT_2_G Short Inter X...Y Contact  C1       ..C4       .       2.93 Ang.
                                                x,1+y,z  =      1_565 Check
PLAT432_ALERT_2_G Short Inter X...Y Contact  C1       ..C2       .       3.01 Ang.
                                           x,3/2-y,1/2+z  =      4_576 Check
PLAT432_ALERT_2_G Short Inter X...Y Contact  C1       ..C1       .       3.01 Ang.
                                             1-x,2-y,1-z  =      3_676 Check
PLAT432_ALERT_2_G Short Inter X...Y Contact  C1       ..C3       .       3.15 Ang.
                                         1-x,1/2+y,1/2-z  =      2_655 Check
PLAT432_ALERT_2_G Short Inter X...Y Contact  C1       ..C1       .       3.18 Ang.
                                        1-x,-1/2+y,1/2-z  =      2_645 Check
PLAT432_ALERT_2_G Short Inter X...Y Contact  C1       ..C1       .       3.18 Ang.
                                         1-x,1/2+y,1/2-z  =      2_655 Check
PLAT432_ALERT_2_G Short Inter X...Y Contact  C2       ..C3       .       2.87 Ang.
                                          -x,1/2+y,1/2-z  =      2_555 Check
PLAT432_ALERT_2_G Short Inter X...Y Contact  C2       ..C5       .       2.94 Ang.
                                         1-x,1/2+y,1/2-z  =      2_655 Check
PLAT432_ALERT_2_G Short Inter X...Y Contact  C2       ..C4       .       3.07 Ang.
                                          -x,1/2+y,1/2-z  =      2_555 Check
PLAT432_ALERT_2_G Short Inter X...Y Contact  C2       ..C5       .       3.08 Ang.
                                          x,3/2-y,-1/2+z  =      4_575 Check
PLAT432_ALERT_2_G Short Inter X...Y Contact  C3       ..C4       .       3.00 Ang.
                                          x,1/2-y,-1/2+z  =      4_565 Check
PLAT432_ALERT_2_G Short Inter X...Y Contact  C3       ..C4       .       3.06 Ang.
                                          -x,1/2+y,1/2-z  =      2_555 Check
```

```
PLAT432_ALERT_2_G Short Inter X...Y Contact  C3       ..C3       .       3.09 Ang.
                                                  -x,1-y,-z  =      3_565 Check
PLAT802_ALERT_4_G CIF Input Record(s) with more than 80 Characters          4 Info
PLAT912_ALERT_4_G Missing # of FCF Reflections Above STh/L=  0.600        316 Note
PLAT933_ALERT_2_G Number of HKL-OMIT Records in Embedded .res File         20 Note
               -3  6  2,  -3  7  1,  -3  8  1,   0  0  2,   0  0  4,   0  1  1,
                0  1  2,   0  1  3,   0  2  0,   0  2  1,   0  2  3,   1  0  2,
                2  2  3,   3  5  3,   3  6  5,   3  7  0,   4  5  4,   4  6  2,
                4  8  0,   4  8  1,
PLAT956_ALERT_1_G Calculated (ThMax) and Actual (FCF) Hmax Differ           8 Units
PLAT969_ALERT_5_G The 'Henn et al.' R-Factor-gap value ..........      9.388 Note
              Predicted wR2: Based on SigI**2  1.99 or SHELX Weight 20.66
PLAT978_ALERT_2_G Number C-C Bonds with Positive Residual Density.          1 Info
PLAT979_ALERT_1_G NoSpherA2 Scattering Factors Used .............     Please Note
```

```
   2 ALERT level A = Most likely a serious problem - resolve or explain
   3 ALERT level B = A potentially serious problem, consider carefully
  14 ALERT level C = Check. Ensure it is not caused by an omission or oversight
  28 ALERT level G = General information/check it is not something unexpected

   6 ALERT type 1 CIF construction/syntax error, inconsistent or missing data
  26 ALERT type 2 Indicator that the structure model may be wrong or deficient
  12 ALERT type 3 Indicator that the structure quality may be low
   2 ALERT type 4 Improvement, methodology, query or suggestion
   1 ALERT type 5 Informative message, check
```

Datablock: Naphthalene_24.5GPa_IAM

```
Bond precision:    C-C = 0.0092 A           Wavelength=0.37380

Cell:              a=6.311(14)     b=5.1374(7)          c=6.5216(18)
                   alpha=90        beta=106.01(10)      gamma=90
Temperature:       293 K
```

	Calculated	Reported
Volume	203.2(5)	203.2(5)
Space group	P 21/c	P 1 21/c 1
Hall group	-P 2ybc	-P 2ybc
Moiety formula	C10 H8	C10 H8
Sum formula	C10 H8	C10 H8
Mr	128.16	128.18
Dx,g cm-3	2.095	2.095
Z	2	2
Mu (mm-1)	0.052	0.051
F000	136.0	136.0
F000'	135.97	
h,k,lmax	12,9,12	4,9,12
Nref	1512	277
Tmin,Tmax	0.999,0.999	0.207,1.000
Tmin'	0.999	

Correction method= # Reported T Limits: Tmin=0.207 Tmax=1.000
AbsCorr = MULTI-SCAN

Data completeness= 0.183 Theta(max)= 21.080

R(reflections)= 0.0958(169) wR2(reflections)= 0.2571(277)

S = 0.978 Npar= 46

The following ALERTS were generated. Each ALERT has the format
test-name_ALERT_alert-type_alert-level.
Click on the hyperlinks for more details of the test.

Alert level A

PLAT029_ALERT_3_A _diffrn_measured_fraction_theta_full value Low . 0.303 Why?

Author Response: This measurement was performed at high pressure which, due to the high

PLAT411_ALERT_2_A Short Inter H...H Contact H3 ..H4 . 1.73 Ang.
-x,1/2+y,1/2-z = 2_555 Check

Author Response: H atoms are close to each other at high pressure.

Alert level B

PLAT088_ALERT_3_B Poor Data / Parameter Ratio .... 6.02 Note

Author Response: This measurement was performed at high pressure which, due to the high

```
PLAT149_ALERT_3_B s.u. on the   beta    Angle is Too Large .......       0.10 Degree
```

Author Response: Large errors due to low completeness.

```
PLAT411_ALERT_2_B Short Inter H...H Contact  H3       ..H3       .       1.95 Ang.
                                               -x,1-y,-z  =      3_565 Check
```

Author Response: H atoms are close to each other at high pressure.

```
PLAT911_ALERT_3_B Missing FCF Refl Between Thmin & STh/L=    0.600        176 Report
                2  0  0,   3  0  0,   4  0  0,   1  1  0,   2  1  0,   3  1  0,
                4  1  0,   1  2  0,   2  2  0,   3  2  0,   4  2  0,   1  3  0,
                3  3  0,   4  3  0,   1  4  0,   2  4  0,   3  4  0,   4  4  0,
                4  5  0,   0  6  0,  -4  1  1,  -3  1  1,  -2  1  1,  -1  1  1,
                1  1  1,   2  1  1,   3  1  1,   4  1  1,  -4  2  1,  -3  2  1,
                   (  146 More Missing: see the .ckf listing file)
```

Author Response: This measurement was performed at high pressure which, due to the high

---

Alert level C

```
PLAT084_ALERT_3_C High wR2 Value (i.e. > 0.25) ..................       0.26 Report
PLAT148_ALERT_3_C s.u. on the      a    - Axis is (Too) Large ....      0.014 Ang.
PLAT234_ALERT_4_C Large Hirshfeld Difference C4       --C5       .       0.20 Ang.
PLAT241_ALERT_2_C High   'MainMol' Ueq as Compared to Neighbors of         C4 Check
PLAT334_ALERT_2_C Small <C-C> Benzene Dist.   C1       -C5_a     .       1.36 Ang.
PLAT334_ALERT_2_C Small <C-C> Benzene Dist.   C5       -C5_a     .       1.36 Ang.
PLAT340_ALERT_3_C Low Bond Precision on  C-C Bonds ...............    0.00917 Ang.
PLAT350_ALERT_3_C Short  C-H (X0.96,N1.08A)  C1       - H1       .       0.93 Ang.
PLAT350_ALERT_3_C Short  C-H (X0.96,N1.08A)  C2       - H2       .       0.93 Ang.
PLAT350_ALERT_3_C Short  C-H (X0.96,N1.08A)  C3       - H3       .       0.93 Ang.
PLAT350_ALERT_3_C Short  C-H (X0.96,N1.08A)  C4       - H4       .       0.93 Ang.
PLAT411_ALERT_2_C Short Inter H...H Contact  H1       ..H4       .       2.07 Ang.
                                                 x,1+y,z  =      1_565 Check
```

Author Response: H atoms are close to each other at high pressure.

```
PLAT411_ALERT_2_C Short Inter H...H Contact  H1       ..H1       .       2.08 Ang.
                                             1-x,2-y,1-z  =      3_676 Check
```

Author Response: H atoms are close to each other at high pressure.

```
PLAT411_ALERT_2_C Short Inter H...H Contact  H2       ..H3       .       2.02 Ang.
                                               -x,1-y,-z  =      3_565 Check
```

Author Response: H atoms are close to each other at high pressure.

```
PLAT906_ALERT_3_C Large K Value in the Analysis of Variance ......      4.510 Check
PLAT913_ALERT_3_C Missing # of Very Strong Reflections in FCF ....          5 Note
              -1  1  1,  -2  0  2,  -1  0  2,  -2  1  2,  -1  1  2,
PLAT939_ALERT_3_C Large Value of Not (SHELXL) Weight Optimized S .      14.64 Check
```

Alert level G

```
ABSMU01_ALERT_1_G  Calculation of _exptl_absorpt_correction_mu
                not performed for this radiation type.
PLAT072_ALERT_2_G SHELXL First  Parameter in WGHT  Unusually Large       0.19 Report
PLAT073_ALERT_1_G H-atoms ref., but hydrogen treatment Reported as     constr Check
PLAT199_ALERT_1_G Reported _cell_measurement_temperature ..... (K)        293 Check
PLAT200_ALERT_1_G Reported   _diffrn_ambient_temperature ..... (K)        293 Check
PLAT432_ALERT_2_G Short Inter X...Y Contact  C1       ..C5      .       2.68 Ang.
                                            1-x,1/2+y,1/2-z  =     2_655 Check
PLAT432_ALERT_2_G Short Inter X...Y Contact  C1       ..C5      .       2.82 Ang.
                                             x,3/2-y,-1/2+z  =     4_575 Check
PLAT432_ALERT_2_G Short Inter X...Y Contact  C1       ..C4      .       2.84 Ang.
                                            1-x,1/2+y,1/2-z  =     2_655 Check
PLAT432_ALERT_2_G Short Inter X...Y Contact  C1       ..C4      .       2.88 Ang.
                                                    x,1+y,z  =     1_565 Check
PLAT432_ALERT_2_G Short Inter X...Y Contact  C1       ..C2      .       2.95 Ang.
                                              x,3/2-y,1/2+z  =     4_576 Check
PLAT432_ALERT_2_G Short Inter X...Y Contact  C1       ..C1      .       2.95 Ang.
                                                 1-x,2-y,1-z  =     3_676 Check
PLAT432_ALERT_2_G Short Inter X...Y Contact  C1       ..C3      .       3.09 Ang.
                                            1-x,1/2+y,1/2-z  =     2_655 Check
PLAT432_ALERT_2_G Short Inter X...Y Contact  C1       ..C1      .       3.15 Ang.
                                            1-x,1/2+y,1/2-z  =     2_655 Check
PLAT432_ALERT_2_G Short Inter X...Y Contact  C1       ..C1      .       3.15 Ang.
                                           1-x,-1/2+y,1/2-z  =     2_645 Check
PLAT432_ALERT_2_G Short Inter X...Y Contact  C1       ..C5      .       3.19 Ang.
                                                    x,1+y,z  =     1_565 Check
PLAT432_ALERT_2_G Short Inter X...Y Contact  C2       ..C3      .       2.80 Ang.
                                             -x,1/2+y,1/2-z  =     2_555 Check
PLAT432_ALERT_2_G Short Inter X...Y Contact  C2       ..C5      .       2.88 Ang.
                                            1-x,1/2+y,1/2-z  =     2_655 Check
PLAT432_ALERT_2_G Short Inter X...Y Contact  C2       ..C4      .       2.97 Ang.
                                             -x,1/2+y,1/2-z  =     2_555 Check
PLAT432_ALERT_2_G Short Inter X...Y Contact  C2       ..C5      .       3.04 Ang.
                                             x,3/2-y,-1/2+z  =     4_575 Check
PLAT432_ALERT_2_G Short Inter X...Y Contact  C3       ..C4      .       2.94 Ang.
                                             x,1/2-y,-1/2+z  =     4_565 Check
PLAT432_ALERT_2_G Short Inter X...Y Contact  C3       ..C4      .       2.99 Ang.
                                             -x,1/2+y,1/2-z  =     2_555 Check
PLAT432_ALERT_2_G Short Inter X...Y Contact  C3       ..C3      .       3.04 Ang.
                                                  -x,1-y,-z  =     3_565 Check
PLAT432_ALERT_2_G Short Inter X...Y Contact  C3       ..C3      .       3.20 Ang.
                                            -x,-1/2+y,1/2-z  =     2_545 Check
PLAT432_ALERT_2_G Short Inter X...Y Contact  C3       ..C3      .       3.20 Ang.
                                             -x,1/2+y,1/2-z  =     2_555 Check
PLAT432_ALERT_2_G Short Inter X...Y Contact  C4       ..C5      .       3.17 Ang.
                                             x,1/2-y,-1/2+z  =     4_565 Check
PLAT802_ALERT_4_G CIF Input Record(s) with more than 80 Characters          4 Info
```

```
PLAT910_ALERT_3_G Missing FCF Reflection(s) Below Theta(Min)[Deg]=       2.70 Note
                1  0  0,
PLAT912_ALERT_4_G Missing # of FCF Reflections Above STh/L=  0.600        351 Note
PLAT933_ALERT_2_G Number of HKL-OMIT Records in Embedded .res File         12 Note
               -1  3  3,  -1  3  4,  -1  4  2,  -1  4  3,  -1  4  4,  -1  4  6,
                0  3 10,   0  7  5,   1  0  2,   1  2  1,   2  2  3,   3  5  5,
PLAT956_ALERT_1_G Calculated (ThMax) and Actual (FCF) Hmax Differ           8 Units
PLAT969_ALERT_5_G The 'Henn et al.' R-Factor-gap value ..........     19.363 Note
              Predicted wR2: Based on SigI**2  1.33 or SHELX Weight 26.22
PLAT978_ALERT_2_G Number C-C Bonds with Positive Residual Density.          3 Info
PLAT979_ALERT_1_G NoSpherA2 Scattering Factors Used .............     Please Note
```

```
   2 ALERT level A = Most likely a serious problem - resolve or explain
   4 ALERT level B = A potentially serious problem, consider carefully
  17 ALERT level C = Check. Ensure it is not caused by an omission or oversight
  33 ALERT level G = General information/check it is not something unexpected

   6 ALERT type 1 CIF construction/syntax error, inconsistent or missing data
  31 ALERT type 2 Indicator that the structure model may be wrong or deficient
  15 ALERT type 3 Indicator that the structure quality may be low
   3 ALERT type 4 Improvement, methodology, query or suggestion
   1 ALERT type 5 Informative message, check
```

Datablock: Naphthalene_28.5GPa_IAM

Bond precision: C-C = 0.0083 A Wavelength=0.37380

Cell:	a=6.231(10)	b=5.1163(7)	c=6.427(2)
	alpha=90	beta=105.08(10)	gamma=90
Temperature:	293 K		

	Calculated	Reported
Volume	197.8(3)	197.8(4)
Space group	P 21/c	P 1 21/c 1
Hall group	-P 2ybc	-P 2ybc
Moiety formula	C10 H8	C10 H8
Sum formula	C10 H8	C10 H8
Mr	128.16	128.18
Dx,g cm-3	2.152	2.152
Z	2	2
Mu (mm-1)	0.053	0.053
F000	136.0	136.0
F000'	135.97	
h,k,lmax	9,8,10	5,8,9
Nref	821	240
Tmin,Tmax	0.999,0.999	0.066,1.000
Tmin'	0.999	

Correction method= # Reported T Limits: Tmin=0.066 Tmax=1.000
AbsCorr = MULTI-SCAN

Data completeness= 0.292 Theta(max)= 17.230

R(reflections)= 0.0778(150) wR2(reflections)= 0.2060(240)

S = 0.986 Npar= 46

The following ALERTS were generated. Each ALERT has the format
test-name_ALERT_alert-type_alert-level.
Click on the hyperlinks for more details of the test.

Alert level A

```
PLAT029_ALERT_3_A _diffrn_measured_fraction_theta_full value Low .      0.332 Why?
```

Author Response: This measurement was performed at high pressure which, due to the high

```
PLAT088_ALERT_3_A Poor Data / Parameter Ratio ..................        5.22 Note
```

Author Response: This measurement was performed at high pressure which, due to the high

```
PLAT411_ALERT_2_A Short Inter H...H Contact  H3       ..H4       .       1.69 Ang.
                                          -x,1/2+y,1/2-z  =      2_555 Check
```

Author Response: H atoms are close to each other at high pressure.

Alert level B

```
PLAT149_ALERT_3_B s.u. on the   beta    Angle is Too Large .......       0.10 Degree
```

Author Response: Large errors due to low completeness.

```
PLAT411_ALERT_2_B Short Inter H...H Contact  H3       ..H3       .       1.89 Ang.
                                              -x,1-y,-z  =      3_565 Check
```

Author Response: H atoms are close to each other at high pressure.

```
PLAT911_ALERT_3_B Missing FCF Refl Between Thmin & STh/L=    0.600        199 Report
                2  0  0,   3  0  0,   4  0  0,   5  0  0,   2  1  0,   3  1  0,
                4  1  0,   5  1  0,   1  2  0,   2  2  0,   3  2  0,   4  2  0,
                5  2  0,   3  3  0,   4  3  0,   5  3  0,   0  4  0,   3  4  0,
                4  4  0,   5  4  0,   4  5  0,   0  6  0,  -5  1  1,  -4  1  1,
               -3  1  1,  -2  1  1,   2  1  1,   3  1  1,   4  1  1,   5  1  1,
                   (  169 More Missing: see the .ckf listing file)
```

Author Response: This measurement was performed at high pressure which, due to the high

---

Alert level C

```
PLAT148_ALERT_3_C s.u. on the      a    - Axis is (Too) Large ....      0.010 Ang.
PLAT334_ALERT_2_C Small <C-C> Benzene Dist.   C1       -C5_a     .       1.37 Ang.
PLAT334_ALERT_2_C Small <C-C> Benzene Dist.   C5       -C5_a     .       1.37 Ang.
PLAT340_ALERT_3_C Low Bond Precision on  C-C Bonds ...............    0.00833 Ang.
PLAT350_ALERT_3_C Short  C-H (X0.96,N1.08A)  C1       - H1       .       0.93 Ang.
PLAT350_ALERT_3_C Short  C-H (X0.96,N1.08A)  C2       - H2       .       0.93 Ang.
PLAT350_ALERT_3_C Short  C-H (X0.96,N1.08A)  C3       - H3       .       0.93 Ang.
PLAT350_ALERT_3_C Short  C-H (X0.96,N1.08A)  C4       - H4       .       0.93 Ang.
PLAT411_ALERT_2_C Short Inter H...H Contact  H1       ..H4       .       2.13 Ang.
                                                x,1+y,z  =      1_565 Check
```

Author Response: H atoms are close to each other at high pressure.

```
PLAT411_ALERT_2_C Short Inter H...H Contact  H1       ..H1       .       2.11 Ang.
                                            1-x,2-y,1-z  =      3_676 Check
```

Author Response: H atoms are close to each other at high pressure.

```
PLAT411_ALERT_2_C Short Inter H...H Contact  H2       ..H3       .       2.00 Ang.
                                              -x,1-y,-z  =      3_565 Check
```

Author Response: H atoms are close to each other at high pressure.

```
PLAT411_ALERT_2_C Short Inter H...H Contact  H3       ..H4       .       2.11 Ang.
                                         x,1/2-y,-1/2+z  =      4_565 Check
```

Author Response: H atoms are close to each other at high pressure.

```
PLAT906_ALERT_3_C Large K Value in the Analysis of Variance ......      2.257 Check
PLAT910_ALERT_3_C Missing FCF Reflection(s) Below Theta(Min)[Deg]=       3.45 Note
                1  0  0,   1  1  0,  -1  1  1,   0  1  1,  -1  0  2,
PLAT913_ALERT_3_C Missing # of Very Strong Reflections in FCF ....          5 Note
               -1  1  1,  -2  0  2,  -1  0  2,  -2  1  2,  -1  1  2,
```

Alert level G

```
ABSMU01_ALERT_1_G  Calculation of _exptl_absorpt_correction_mu
                not performed for this radiation type.
PLAT072_ALERT_2_G SHELXL First  Parameter in WGHT  Unusually Large       0.14 Report
PLAT073_ALERT_1_G H-atoms ref., but hydrogen treatment Reported as     constr Check
PLAT199_ALERT_1_G Reported _cell_measurement_temperature ..... (K)        293 Check
PLAT200_ALERT_1_G Reported   _diffrn_ambient_temperature ..... (K)        293 Check
PLAT432_ALERT_2_G Short Inter X...Y Contact  C1       ..C5       .       2.60 Ang.
                                         1-x,1/2+y,1/2-z  =      2_655 Check
PLAT432_ALERT_2_G Short Inter X...Y Contact  C1       ..C5       .       2.76 Ang.
```

```
                                         x,3/2-y,-1/2+z  =      4_575 Check
PLAT432_ALERT_2_G Short Inter X...Y Contact  C1       ..C4       .       2.78 Ang.
                                       1-x,1/2+y,1/2-z  =      2_655 Check
PLAT432_ALERT_2_G Short Inter X...Y Contact  C1       ..C4       .       2.87 Ang.
                                                x,1+y,z  =      1_565 Check
PLAT432_ALERT_2_G Short Inter X...Y Contact  C1       ..C2       .       2.90 Ang.
                                          x,3/2-y,1/2+z  =      4_576 Check
PLAT432_ALERT_2_G Short Inter X...Y Contact  C1       ..C1       .       2.93 Ang.
                                            1-x,2-y,1-z  =      3_676 Check
PLAT432_ALERT_2_G Short Inter X...Y Contact  C1       ..C3       .       3.01 Ang.
                                        1-x,1/2+y,1/2-z  =      2_655 Check
PLAT432_ALERT_2_G Short Inter X...Y Contact  C1       ..C1       .       3.08 Ang.
                                        1-x,1/2+y,1/2-z  =      2_655 Check
PLAT432_ALERT_2_G Short Inter X...Y Contact  C1       ..C1       .       3.08 Ang.
                                       1-x,-1/2+y,1/2-z  =      2_645 Check
PLAT432_ALERT_2_G Short Inter X...Y Contact  C1       ..C2       .       3.16 Ang.
                                        1-x,1/2+y,1/2-z  =      2_655 Check
PLAT432_ALERT_2_G Short Inter X...Y Contact  C1       ..C5       .       3.17 Ang.
                                                x,1+y,z  =      1_565 Check
PLAT432_ALERT_2_G Short Inter X...Y Contact  C2       ..C3       .       2.77 Ang.
                                         -x,1/2+y,1/2-z  =      2_555 Check
PLAT432_ALERT_2_G Short Inter X...Y Contact  C2       ..C5       .       2.80 Ang.
                                        1-x,1/2+y,1/2-z  =      2_655 Check
PLAT432_ALERT_2_G Short Inter X...Y Contact  C2       ..C4       .       2.92 Ang.
                                         -x,1/2+y,1/2-z  =      2_555 Check
PLAT432_ALERT_2_G Short Inter X...Y Contact  C2       ..C5       .       2.97 Ang.
                                         x,3/2-y,-1/2+z  =      4_575 Check
PLAT432_ALERT_2_G Short Inter X...Y Contact  C3       ..C4       .       2.87 Ang.
                                         x,1/2-y,-1/2+z  =      4_565 Check
PLAT432_ALERT_2_G Short Inter X...Y Contact  C3       ..C4       .       2.95 Ang.
                                         -x,1/2+y,1/2-z  =      2_555 Check
PLAT432_ALERT_2_G Short Inter X...Y Contact  C3       ..C3       .       3.02 Ang.
                                              -x,1-y,-z  =      3_565 Check
PLAT432_ALERT_2_G Short Inter X...Y Contact  C3       ..C3       .       3.18 Ang.
                                         -x,1/2+y,1/2-z  =      2_555 Check
PLAT432_ALERT_2_G Short Inter X...Y Contact  C3       ..C3       .       3.18 Ang.
                                        -x,-1/2+y,1/2-z  =      2_545 Check
PLAT432_ALERT_2_G Short Inter X...Y Contact  C4       ..C5       .       3.13 Ang.
                                         x,1/2-y,-1/2+z  =      4_565 Check
PLAT802_ALERT_4_G CIF Input Record(s) with more than 80 Characters          4 Info
PLAT912_ALERT_4_G Missing # of FCF Reflections Above STh/L=  0.600        165 Note
PLAT933_ALERT_2_G Number of HKL-OMIT Records in Embedded .res File          8 Note
               -2  5  1,  -1  3  1,   0  1  1,   0  9  3,   1  2  0,   2  3  4,
                3  5  5,   4  5  5,
PLAT956_ALERT_1_G Calculated (ThMax) and Actual (FCF) Hmax Differ           4 Units
PLAT969_ALERT_5_G The 'Henn et al.' R-Factor-gap value ..........       3.750 Note
              Predicted wR2: Based on SigI**2  5.49 or SHELX Weight 20.85
PLAT978_ALERT_2_G Number C-C Bonds with Positive Residual Density.          7 Info
PLAT979_ALERT_1_G NoSpherA2 Scattering Factors Used .............     Please Note
```

---

3 **ALERT level A** = Most likely a serious problem - resolve or explain
3 **ALERT level B** = A potentially serious problem, consider carefully
15 **ALERT level C** = Check. Ensure it is not caused by an omission or oversight
33 **ALERT level G** = General information/check it is not something unexpected

6 ALERT type 1 CIF construction/syntax error, inconsistent or missing data

```
32 ALERT type 2 Indicator that the structure model may be wrong or deficient
13 ALERT type 3 Indicator that the structure quality may be low
 2 ALERT type 4 Improvement, methodology, query or suggestion
 1 ALERT type 5 Informative message, check
```

Datablock: Naphthalene_35.1GPa_IAM

Bond precision: C-C = 0.0085 A Wavelength=0.37380

Cell:	a=6.125(11)	b=5.0575(7)	c=6.322(3)
	alpha=90	beta=104.40(11)	gamma=90
Temperature:	293 K		

	Calculated	Reported
Volume	189.7(4)	189.7(4)
Space group	P 21/c	P 1 21/c 1
Hall group	-P 2ybc	-P 2ybc
Moiety formula	C10 H8	C10 H8
Sum formula	C10 H8	C10 H8
Mr	128.16	128.18
Dx,g cm-3	2.244	2.244
Z	2	2
Mu (mm-1)	0.055	0.055
F000	136.0	136.0
F000’	135.97	
h,k,lmax	9,8,10	5,8,9
Nref	829	227
Tmin,Tmax	0.999,0.999	0.312,1.000
Tmin’	0.999	

Correction method= # Reported T Limits: Tmin=0.312 Tmax=1.000
AbsCorr = MULTI-SCAN

Data completeness= 0.274 Theta(max)= 17.540

R(reflections)= 0.0926(154) wR2(reflections)= 0.2495(227)

S = 1.045 Npar= 46

The following ALERTS were generated. Each ALERT has the format
test-name_ALERT_alert-type_alert-level.
Click on the hyperlinks for more details of the test.

Alert level A

```
PLAT029_ALERT_3_A _diffrn_measured_fraction_theta_full value Low .      0.308 Why?
```

Author Response: This measurement was performed at high pressure which, due to the high

```
PLAT088_ALERT_3_A Poor Data / Parameter Ratio ....................       4.93 Note
```

Author Response: This measurement was performed at high pressure which, due to the high

```
PLAT411_ALERT_2_A Short Inter H...H Contact  H3        ..H4        .       1.67 Ang.
                                             -x,1/2+y,1/2-z  =      2_555 Check
```

Author Response: H atoms are close to each other at high pressure.

Alert level B

```
PLAT149_ALERT_3_B s.u. on the   beta    Angle is Too Large .......       0.11 Degree
```

Author Response: Large errors due to low completeness.

```
PLAT411_ALERT_2_B Short Inter H...H Contact  H2        ..H3        .       1.95 Ang.
                                                  -x,1-y,-z  =      3_565 Check
```

Author Response: H atoms are close to each other at high pressure.

```
PLAT411_ALERT_2_B Short Inter H...H Contact  H3        ..H3        .       1.87 Ang.
                                                  -x,1-y,-z  =      3_565 Check
```

Author Response: H atoms are close to each other at high pressure.

```
PLAT911_ALERT_3_B Missing FCF Refl Between Thmin & STh/L=    0.600        203 Report
                2  0  0,   3  0  0,   4  0  0,   5  0  0,   2  1  0,   3  1  0,
                4  1  0,   5  1  0,   0  2  0,   1  2  0,   2  2  0,   3  2  0,
                4  2  0,   5  2  0,   3  3  0,   4  3  0,   5  3  0,   3  4  0,
                4  4  0,   5  4  0,   2  5  0,   4  5  0,   0  6  0,  -5  1  1,
               -4  1  1,  -3  1  1,  -2  1  1,   2  1  1,   3  1  1,   4  1  1,
                   (  173 More Missing: see the .ckf listing file)
```

Author Response: This measurement was performed at high pressure which, due to the high

Alert level C

```
PLAT148_ALERT_3_C s.u. on the      a    - Axis is (Too) Large ....      0.011 Ang.
PLAT334_ALERT_2_C Small <C-C> Benzene Dist.   C1       -C5_a     .       1.37 Ang.
PLAT334_ALERT_2_C Small <C-C> Benzene Dist.   C5       -C5_a     .       1.37 Ang.
PLAT340_ALERT_3_C Low Bond Precision on  C-C Bonds ..............     0.0085 Ang.
PLAT350_ALERT_3_C Short  C-H (X0.96,N1.08A)  C1       - H1       .       0.93 Ang.
PLAT350_ALERT_3_C Short  C-H (X0.96,N1.08A)  C2       - H2       .       0.93 Ang.
PLAT350_ALERT_3_C Short  C-H (X0.96,N1.08A)  C3       - H3       .       0.93 Ang.
PLAT350_ALERT_3_C Short  C-H (X0.96,N1.08A)  C4       - H4       .       0.93 Ang.
PLAT411_ALERT_2_C Short Inter H...H Contact  H1       ..H4       .       2.04 Ang.
                                              x,1+y,z  =      1_565 Check
```

Author Response: H atoms are close to each other at high pressure.

```
PLAT411_ALERT_2_C Short Inter H...H Contact  H1       ..H1       .       2.12 Ang.
                                          1-x,2-y,1-z  =      3_676 Check
```

Author Response: H atoms are close to each other at high pressure.

```
PLAT411_ALERT_2_C Short Inter H...H Contact  H2       ..H3       .       2.12 Ang.
                                         -x,1/2+y,1/2-z  =      2_555 Check
```

Author Response: H atoms are close to each other at high pressure.

```
PLAT411_ALERT_2_C Short Inter H...H Contact  H3       ..H4       .       2.10 Ang.
                                         x,1/2-y,-1/2+z  =      4_565 Check
```

Author Response: H atoms are close to each other at high pressure.

```
PLAT767_ALERT_4_C INS Embedded LIST 6 Instruction Should be LIST 4     Please Check
PLAT910_ALERT_3_C Missing FCF Reflection(s) Below Theta(Min)[Deg]=       3.52 Note
                1  0  0,   1  1  0,  -1  1  1,   0  1  1,  -1  0  2,   0  0  2,
PLAT913_ALERT_3_C Missing # of Very Strong Reflections in FCF ....          5 Note
               -1  1  1,  -2  0  2,  -1  0  2,  -2  1  2,  -1  1  2,
```

Alert level G

```
ABSMU01_ALERT_1_G  Calculation of _exptl_absorpt_correction_mu
                not performed for this radiation type.
PLAT003_ALERT_2_G Number of Uiso or U(i,j)  Restrained non-H-Atoms          2 Report
PLAT072_ALERT_2_G SHELXL First  Parameter in WGHT  Unusually Large       0.19 Report
PLAT073_ALERT_1_G H-atoms ref., but hydrogen treatment Reported as     constr Check
PLAT186_ALERT_4_G The CIF-Embedded .res File Contains ISOR Records          2 Report
PLAT199_ALERT_1_G Reported _cell_measurement_temperature ..... (K)        293 Check
PLAT200_ALERT_1_G Reported   _diffrn_ambient_temperature ..... (K)        293 Check
PLAT432_ALERT_2_G Short Inter X...Y Contact  C1       ..C5       .       2.55 Ang.
                                        1-x,1/2+y,1/2-z  =      2_655 Check
PLAT432_ALERT_2_G Short Inter X...Y Contact  C1       ..C4       .       2.71 Ang.
                                        1-x,1/2+y,1/2-z  =      2_655 Check
```

```
PLAT432_ALERT_2_G Short Inter X...Y Contact  C1       ..C5       .       2.71 Ang.
                                       x,3/2-y,-1/2+z  =     4_575 Check
PLAT432_ALERT_2_G Short Inter X...Y Contact  C1       ..C4       .       2.80 Ang.
                                             x,1+y,z  =     1_565 Check
PLAT432_ALERT_2_G Short Inter X...Y Contact  C1       ..C2       .       2.87 Ang.
                                        x,3/2-y,1/2+z  =     4_576 Check
PLAT432_ALERT_2_G Short Inter X...Y Contact  C1       ..C1       .       2.89 Ang.
                                          1-x,2-y,1-z  =     3_676 Check
PLAT432_ALERT_2_G Short Inter X...Y Contact  C1       ..C3       .       2.98 Ang.
                                      1-x,1/2+y,1/2-z  =     2_655 Check
PLAT432_ALERT_2_G Short Inter X...Y Contact  C1       ..C1       .       3.04 Ang.
                                     1-x,-1/2+y,1/2-z  =     2_645 Check
PLAT432_ALERT_2_G Short Inter X...Y Contact  C1       ..C1       .       3.04 Ang.
                                      1-x,1/2+y,1/2-z  =     2_655 Check
PLAT432_ALERT_2_G Short Inter X...Y Contact  C1       ..C5       .       3.11 Ang.
                                             x,1+y,z  =     1_565 Check
PLAT432_ALERT_2_G Short Inter X...Y Contact  C1       ..C2       .       3.16 Ang.
                                      1-x,1/2+y,1/2-z  =     2_655 Check
PLAT432_ALERT_2_G Short Inter X...Y Contact  C1       ..C1       .       3.20 Ang.
                                       x,3/2-y,-1/2+z  =     4_575 Check
PLAT432_ALERT_2_G Short Inter X...Y Contact  C1       ..C1       .       3.20 Ang.
                                        x,3/2-y,1/2+z  =     4_576 Check
PLAT432_ALERT_2_G Short Inter X...Y Contact  C2       ..C3       .       2.64 Ang.
                                       -x,1/2+y,1/2-z  =     2_555 Check
PLAT432_ALERT_2_G Short Inter X...Y Contact  C2       ..C5       .       2.80 Ang.
                                      1-x,1/2+y,1/2-z  =     2_655 Check
PLAT432_ALERT_2_G Short Inter X...Y Contact  C2       ..C4       .       2.84 Ang.
                                       -x,1/2+y,1/2-z  =     2_555 Check
PLAT432_ALERT_2_G Short Inter X...Y Contact  C2       ..C5       .       2.96 Ang.
                                       x,3/2-y,-1/2+z  =     4_575 Check
PLAT432_ALERT_2_G Short Inter X...Y Contact  C2       ..C4       .       3.15 Ang.
                                             x,1+y,z  =     1_565 Check
PLAT432_ALERT_2_G Short Inter X...Y Contact  C2       ..C3       .       3.17 Ang.
                                           -x,1-y,-z  =     3_565 Check
PLAT432_ALERT_2_G Short Inter X...Y Contact  C2       ..C2       .       3.17 Ang.
                                        x,3/2-y,1/2+z  =     4_576 Check
PLAT432_ALERT_2_G Short Inter X...Y Contact  C2       ..C2       .       3.17 Ang.
                                       x,3/2-y,-1/2+z  =     4_575 Check
PLAT432_ALERT_2_G Short Inter X...Y Contact  C2       ..C4       .       3.20 Ang.
                                       x,3/2-y,-1/2+z  =     4_575 Check
PLAT432_ALERT_2_G Short Inter X...Y Contact  C3       ..C4       .       2.82 Ang.
                                       x,1/2-y,-1/2+z  =     4_565 Check
PLAT432_ALERT_2_G Short Inter X...Y Contact  C3       ..C4       .       2.89 Ang.
                                       -x,1/2+y,1/2-z  =     2_555 Check
PLAT432_ALERT_2_G Short Inter X...Y Contact  C3       ..C3       .       2.94 Ang.
                                           -x,1-y,-z  =     3_565 Check
PLAT432_ALERT_2_G Short Inter X...Y Contact  C3       ..C3       .       3.11 Ang.
                                      -x,-1/2+y,1/2-z  =     2_545 Check
PLAT432_ALERT_2_G Short Inter X...Y Contact  C3       ..C3       .       3.11 Ang.
                                       -x,1/2+y,1/2-z  =     2_555 Check
PLAT432_ALERT_2_G Short Inter X...Y Contact  C3       ..C5       .       3.15 Ang.
                                       x,1/2-y,-1/2+z  =     4_565 Check
PLAT432_ALERT_2_G Short Inter X...Y Contact  C4       ..C5       .       3.04 Ang.
                                       x,1/2-y,-1/2+z  =     4_565 Check
PLAT802_ALERT_4_G CIF Input Record(s) with more than 80 Characters          4 Info
PLAT860_ALERT_3_G Number of Least-Squares Restraints ............          12 Note
PLAT912_ALERT_4_G Missing # of FCF Reflections Above STh/L=  0.600        178 Note
```

```
PLAT933_ALERT_2_G Number of HKL-OMIT Records in Embedded .res File         20 Note
               -2  6  4,   0  0  2,   0  1  2,   0  1  3,   0  2  0,   0  2  1,
                0  2  3,   0  2  5,   0  3  3,   0  4  1,   1  2  0,   1  8  4,
                2  9  2,   3  1  8,   4  2  6,   4  3  6,   4  3  7,   4  5  5,
                4  6  1,   5  3  6,
PLAT956_ALERT_1_G Calculated (ThMax) and Actual (FCF) Hmax Differ           4 Units
PLAT969_ALERT_5_G The 'Henn et al.' R-Factor-gap value ..........      8.638 Note
              Predicted wR2: Based on SigI**2  2.89 or SHELX Weight 23.81
PLAT978_ALERT_2_G Number C-C Bonds with Positive Residual Density.          4 Info
PLAT979_ALERT_1_G NoSpherA2 Scattering Factors Used .............     Please Note
```

---

   3 **ALERT level A** = Most likely a serious problem - resolve or explain
   4 **ALERT level B** = A potentially serious problem, consider carefully
  15 **ALERT level C** = Check. Ensure it is not caused by an omission or oversight
  44 **ALERT level G** = General information/check it is not something unexpected

   6 ALERT type 1 CIF construction/syntax error, inconsistent or missing data
  42 ALERT type 2 Indicator that the structure model may be wrong or deficient
  13 ALERT type 3 Indicator that the structure quality may be low
   4 ALERT type 4 Improvement, methodology, query or suggestion
   1 ALERT type 5 Informative message, check

Datablock: Naphthalene_43.3GPa_IAM

Bond precision: C-C = 0.0157 A Wavelength=0.37380

Cell:	a=6.00(3)	b=5.009(2)	c=6.080(8)
	alpha=90	beta=102.6(3)	gamma=90
Temperature:	293 K		

	Calculated	Reported
Volume	178.3(9)	178.3(10)
Space group	P 21/c	P 1 21/c 1
Hall group	-P 2ybc	-P 2ybc
Moiety formula	C10 H8	C10 H8
Sum formula	C10 H8	C10 H8
Mr	128.16	128.18
Dx,g cm-3	2.387	2.388
Z	2	2
Mu (mm-1)	0.059	0.058
F000	136.0	136.0
F000'	135.97	
h,k,lmax	8,7,9	5,7,8
Nref	615	169
Tmin,Tmax	0.999,0.999	0.203,1.000
Tmin'	0.999	

Correction method= # Reported T Limits: Tmin=0.203 Tmax=1.000
AbsCorr = MULTI-SCAN

Data completeness= 0.275 Theta(max)= 16.130

R(reflections)= 0.0975(99) wR2(reflections)= 0.2843(169)

S = 1.166 Npar= 46

The following ALERTS were generated. Each ALERT has the format
test-name_ALERT_alert-type_alert-level.
Click on the hyperlinks for more details of the test.

Alert level A

PLAT029_ALERT_3_A _diffrn_measured_fraction_theta_full value Low . 0.300 Why?

Author Response: This measurement was performed at high pressure which, due to the high

PLAT088_ALERT_3_A Poor Data / Parameter Ratio .... 3.67 Note

Author Response: This measurement was performed at high pressure which, due to the high

PLAT411_ALERT_2_A Short Inter H...H Contact H3 ..H4 . 1.56 Ang.
-x,1/2+y,1/2-z = 2_555 Check

Author Response: H atoms are close to each other at high pressure.

PLAT411_ALERT_2_A Short Inter H...H Contact H3 ..H3 . 1.74 Ang.
-x,1-y,-z = 3_565 Check

Author Response: H atoms are close to each other at high pressure.

Alert level B

PLAT149_ALERT_3_B s.u. on the beta Angle is Too Large .... 0.30 Degree

Author Response: Large errors due to low completeness.

PLAT340_ALERT_3_B Low Bond Precision on C-C Bonds .... 0.01567 Ang.

Author Response: Large errors due to low completeness.

```
PLAT411_ALERT_2_B Short Inter H...H Contact  H2        ..H3        .        1.94 Ang.
                                           -x,1-y,-z  =      3_565 Check
```

Author Response: H atoms are close to each other at high pressure.

```
PLAT411_ALERT_2_B Short Inter H...H Contact  H3        ..H4        .        1.84 Ang.
                                      x,1/2-y,-1/2+z  =      4_565 Check
```

Author Response: H atoms are close to each other at high pressure.

```
PLAT911_ALERT_3_B Missing FCF Refl Between Thmin & STh/L=    0.600        196 Report
                2  0  0,   3  0  0,   4  0  0,   5  0  0,   2  1  0,   3  1  0,
                4  1  0,   5  1  0,   0  2  0,   1  2  0,   2  2  0,   3  2  0,
                4  2  0,   5  2  0,   3  3  0,   4  3  0,   5  3  0,   3  4  0,
                4  4  0,   5  4  0,   3  5  0,   0  6  0,  -5  1  1,  -4  1  1,
               -3  1  1,  -2  1  1,   2  1  1,   3  1  1,   4  1  1,   5  1  1,
                    (  166 More Missing: see the .ckf listing file)
```

Author Response: This measurement was performed at high pressure which, due to the high

---

Alert level C

```
PLAT084_ALERT_3_C High wR2 Value (i.e. > 0.25) ...................       0.28 Report
PLAT148_ALERT_3_C s.u. on the      a     - Axis is (Too) Large ....      0.030 Ang.
PLAT148_ALERT_3_C s.u. on the      c     - Axis is (Too) Large ....      0.008 Ang.
PLAT350_ALERT_3_C Short  C-H (X0.96,N1.08A)  C1        - H1        .       0.93 Ang.
PLAT350_ALERT_3_C Short  C-H (X0.96,N1.08A)  C2        - H2        .       0.93 Ang.
PLAT350_ALERT_3_C Short  C-H (X0.96,N1.08A)  C3        - H3        .       0.93 Ang.
PLAT350_ALERT_3_C Short  C-H (X0.96,N1.08A)  C4        - H4        .       0.93 Ang.
PLAT411_ALERT_2_C Short Inter H...H Contact  H1        ..H4        .       2.14 Ang.
                                              x,1+y,z  =      1_565 Check
```

Author Response: H atoms are close to each other at high pressure.

```
PLAT411_ALERT_2_C Short Inter H...H Contact  H1        ..H1        .       2.01 Ang.
                                         1-x,2-y,1-z  =      3_676 Check
```

Author Response: H atoms are close to each other at high pressure.

```
PLAT913_ALERT_3_C Missing # of Very Strong Reflections in FCF ....         13 Note
                1  0  0,   3  0  0,   0  2  0,   1  2  0,  -1  1  1,   2  1  1,
               -2  0  2,  -1  0  2,   0  0  2,  -2  1  2,  -1  1  2,   1  2  2,
                0  1  3,
PLAT939_ALERT_3_C Large Value of Not (SHELXL) Weight Optimized S .      19.43 Check
```

Alert level G

```
ABSMU01_ALERT_1_G  Calculation of _exptl_absorpt_correction_mu
                not performed for this radiation type.
```

```
PLAT072_ALERT_2_G SHELXL First  Parameter in WGHT  Unusually Large       0.19 Report
PLAT073_ALERT_1_G H-atoms ref., but hydrogen treatment Reported as     constr Check
PLAT199_ALERT_1_G Reported _cell_measurement_temperature ..... (K)        293 Check
PLAT200_ALERT_1_G Reported   _diffrn_ambient_temperature ..... (K)        293 Check
PLAT432_ALERT_2_G Short Inter X...Y Contact  C1       ..C5       .       2.46 Ang.
                                           1-x,1/2+y,1/2-z  =      2_655 Check
PLAT432_ALERT_2_G Short Inter X...Y Contact  C1       ..C5       .       2.60 Ang.
                                            x,3/2-y,-1/2+z  =      4_575 Check
PLAT432_ALERT_2_G Short Inter X...Y Contact  C1       ..C4       .       2.67 Ang.
                                           1-x,1/2+y,1/2-z  =      2_655 Check
PLAT432_ALERT_2_G Short Inter X...Y Contact  C1       ..C2       .       2.72 Ang.
                                             x,3/2-y,1/2+z  =      4_576 Check
PLAT432_ALERT_2_G Short Inter X...Y Contact  C1       ..C4       .       2.81 Ang.
                                                   x,1+y,z  =      1_565 Check
PLAT432_ALERT_2_G Short Inter X...Y Contact  C1       ..C1       .       2.84 Ang.
                                               1-x,2-y,1-z  =      3_676 Check
PLAT432_ALERT_2_G Short Inter X...Y Contact  C1       ..C3       .       2.87 Ang.
                                           1-x,1/2+y,1/2-z  =      2_655 Check
PLAT432_ALERT_2_G Short Inter X...Y Contact  C1       ..C1       .       2.96 Ang.
                                           1-x,1/2+y,1/2-z  =      2_655 Check
PLAT432_ALERT_2_G Short Inter X...Y Contact  C1       ..C1       .       2.96 Ang.
                                          1-x,-1/2+y,1/2-z  =      2_645 Check
PLAT432_ALERT_2_G Short Inter X...Y Contact  C1       ..C2       .       3.05 Ang.
                                           1-x,1/2+y,1/2-z  =      2_655 Check
PLAT432_ALERT_2_G Short Inter X...Y Contact  C1       ..C1       .       3.08 Ang.
                                            x,3/2-y,-1/2+z  =      4_575 Check
PLAT432_ALERT_2_G Short Inter X...Y Contact  C1       ..C1       .       3.08 Ang.
                                             x,3/2-y,1/2+z  =      4_576 Check
PLAT432_ALERT_2_G Short Inter X...Y Contact  C1       ..C5       .       3.08 Ang.
                                                   x,1+y,z  =      1_565 Check
PLAT432_ALERT_2_G Short Inter X...Y Contact  C2       ..C3       .       2.65 Ang.
                                            -x,1/2+y,1/2-z  =      2_555 Check
PLAT432_ALERT_2_G Short Inter X...Y Contact  C2       ..C5       .       2.69 Ang.
                                           1-x,1/2+y,1/2-z  =      2_655 Check
PLAT432_ALERT_2_G Short Inter X...Y Contact  C2       ..C4       .       2.77 Ang.
                                            -x,1/2+y,1/2-z  =      2_555 Check
PLAT432_ALERT_2_G Short Inter X...Y Contact  C2       ..C5       .       2.78 Ang.
                                            x,3/2-y,-1/2+z  =      4_575 Check
PLAT432_ALERT_2_G Short Inter X...Y Contact  C2       ..C2       .       3.05 Ang.
                                             x,3/2-y,1/2+z  =      4_576 Check
PLAT432_ALERT_2_G Short Inter X...Y Contact  C2       ..C2       .       3.05 Ang.
                                            x,3/2-y,-1/2+z  =      4_575 Check
PLAT432_ALERT_2_G Short Inter X...Y Contact  C2       ..C4       .       3.06 Ang.
                                            x,3/2-y,-1/2+z  =      4_575 Check
PLAT432_ALERT_2_G Short Inter X...Y Contact  C2       ..C4       .       3.11 Ang.
                                                   x,1+y,z  =      1_565 Check
PLAT432_ALERT_2_G Short Inter X...Y Contact  C2       ..C3       .       3.18 Ang.
                                                 -x,1-y,-z  =      3_565 Check
PLAT432_ALERT_2_G Short Inter X...Y Contact  C3       ..C4       .       2.67 Ang.
                                            x,1/2-y,-1/2+z  =      4_565 Check
PLAT432_ALERT_2_G Short Inter X...Y Contact  C3       ..C4       .       2.80 Ang.
                                            -x,1/2+y,1/2-z  =      2_555 Check
PLAT432_ALERT_2_G Short Inter X...Y Contact  C3       ..C3       .       2.92 Ang.
                                                 -x,1-y,-z  =      3_565 Check
PLAT432_ALERT_2_G Short Inter X...Y Contact  C3       ..C5       .       3.02 Ang.
                                            x,1/2-y,-1/2+z  =      4_565 Check
PLAT432_ALERT_2_G Short Inter X...Y Contact  C3       ..C3       .       3.08 Ang.
```

```
                                            -x,-1/2+y,1/2-z  =      2_545 Check
PLAT432_ALERT_2_G Short Inter X...Y Contact  C3       ..C3       .       3.08 Ang.
                                             -x,1/2+y,1/2-z  =      2_555 Check
PLAT432_ALERT_2_G Short Inter X...Y Contact  C4       ..C5       .       2.95 Ang.
                                             x,1/2-y,-1/2+z  =      4_565 Check
PLAT432_ALERT_2_G Short Inter X...Y Contact  C4       ..C4       .       3.09 Ang.
                                             x,1/2-y,-1/2+z  =      4_565 Check
PLAT432_ALERT_2_G Short Inter X...Y Contact  C4       ..C4       .       3.09 Ang.
                                              x,1/2-y,1/2+z  =      4_566 Check
PLAT802_ALERT_4_G CIF Input Record(s) with more than 80 Characters          4 Info
PLAT910_ALERT_3_G Missing FCF Reflection(s) Below Theta(Min)[Deg]=       3.55 Note
                1  0  0,   1  1  0,  -1  1  1,   0  1  1,
PLAT912_ALERT_4_G Missing # of FCF Reflections Above STh/L=  0.600        130 Note
PLAT933_ALERT_2_G Number of HKL-OMIT Records in Embedded .res File         11 Note
                0  1  3,   0  2  0,   0  2  1,   0  5  2,   0  6  0,   0  7  1,
                3  0  4,   3  1  3,   3  1  7,   3  1  8,   3  2  4,
PLAT956_ALERT_1_G Calculated (ThMax) and Actual (FCF) Hmax Differ           3 Units
PLAT969_ALERT_5_G The 'Henn et al.' R-Factor-gap value ..........     25.275 Note
              Predicted wR2: Based on SigI**2  1.12 or SHELX Weight 24.28
PLAT978_ALERT_2_G Number C-C Bonds with Positive Residual Density.          1 Info
PLAT979_ALERT_1_G NoSpherA2 Scattering Factors Used .............     Please Note
```

```
   4 ALERT level A = Most likely a serious problem - resolve or explain
   5 ALERT level B = A potentially serious problem, consider carefully
  11 ALERT level C = Check. Ensure it is not caused by an omission or oversight
  44 ALERT level G = General information/check it is not something unexpected

   6 ALERT type 1 CIF construction/syntax error, inconsistent or missing data
  40 ALERT type 2 Indicator that the structure model may be wrong or deficient
  15 ALERT type 3 Indicator that the structure quality may be low
   2 ALERT type 4 Improvement, methodology, query or suggestion
   1 ALERT type 5 Informative message, check
```

Datablock: Naphthalene_46.6GPa_IAM

Bond precision:	C-C = 0.0117 A		Wavelength=0.37380
Cell:	a=6.03(2)	b=4.9580(17)	c=6.103(8)
	alpha=90	beta=103.7(3)	gamma=90
Temperature:	293 K		

	Calculated	Reported
Volume	177.3(7)	177.2(7)
Space group	P 21/c	P 1 21/c 1
Hall group	-P 2ybc	-P 2ybc
Moiety formula	C10 H8	C10 H8
Sum formula	C10 H8	C10 H8
Mr	128.16	128.18
Dx,g cm-3	2.401	2.403
Z	2	2
Mu (mm-1)	0.059	0.059
F000	136.0	136.0
F000'	135.97	
h,k,lmax	9,7,9	5,7,8
Nref	666	188
Tmin,Tmax	0.999,0.999	0.128,1.000
Tmin'	0.999	

Correction method= # Reported T Limits: Tmin=0.128 Tmax=1.000
AbsCorr = MULTI-SCAN

Data completeness= 0.282   Theta(max)= 16.650

R(reflections)= 0.0967(107)   wR2(reflections)= 0.2738(188)

S = 1.082   Npar= 46

The following ALERTS were generated. Each ALERT has the format
test-name_ALERT_alert-type_alert-level.
Click on the hyperlinks for more details of the test.

Alert level A

PLAT029_ALERT_3_A _diffrn_measured_fraction_theta_full value Low .   0.324 Why?

Author Response: This measurement was performed at high pressure which, due to the high

PLAT088_ALERT_3_A Poor Data / Parameter Ratio ....   4.09 Note

Author Response: This measurement was performed at high pressure which, due to the high

PLAT411_ALERT_2_A Short Inter H...H Contact H3 ..H4 . 1.66 Ang.
-x,1/2+y,1/2-z = 2_555 Check

Author Response: H atoms are close to each other at high pressure.

```
PLAT411_ALERT_2_A Short Inter H...H Contact  H3       ..H3       .       1.65 Ang.
                                              -x,1-y,-z  =      3_565 Check
```

Author Response: H atoms are close to each other at high pressure.

Alert level B

```
PLAT149_ALERT_3_B s.u. on the   beta    Angle is Too Large .......       0.30 Degree
```

Author Response: Large errors due to low completeness.

```
PLAT340_ALERT_3_B Low Bond Precision on  C-C Bonds ...............    0.01167 Ang.
```

Author Response: Large errors due to low completeness.

```
PLAT411_ALERT_2_B Short Inter H...H Contact  H1       ..H4       .       1.89 Ang.
                                                x,1+y,z  =      1_565 Check
```

Author Response: H atoms are close to each other at high pressure.

```
PLAT411_ALERT_2_B Short Inter H...H Contact  H1       ..H1       .       1.82 Ang.
                                            1-x,2-y,1-z  =      3_676 Check
```

Author Response: H atoms are close to each other at high pressure.

```
PLAT411_ALERT_2_B Short Inter H...H Contact  H2       ..H3       .       1.85 Ang.
                                              -x,1-y,-z  =      3_565 Check
```

Author Response: H atoms are close to each other at high pressure.

```
PLAT411_ALERT_2_B Short Inter H...H Contact  H3       ..H4       .       1.86 Ang.
                                       x,1/2-y,-1/2+z  =      4_565 Check
```

Author Response: H atoms are close to each other at high pressure.

```
PLAT911_ALERT_3_B Missing FCF Refl Between Thmin & STh/L=    0.600        193 Report
                2  0  0,   3  0  0,   4  0  0,   5  0  0,   2  1  0,   3  1  0,
                4  1  0,   5  1  0,   0  2  0,   2  2  0,   3  2  0,   4  2  0,
                5  2  0,   2  3  0,   3  3  0,   4  3  0,   5  3  0,   0  4  0,
                3  4  0,   4  4  0,   5  4  0,   2  5  0,   3  5  0,  -5  1  1,
               -4  1  1,  -3  1  1,  -2  1  1,   3  1  1,   4  1  1,   5  1  1,
                   (  163 More Missing: see the .ckf listing file)
```

Author Response: This measurement was performed at high pressure which, due to the high

Alert level C

```
PLAT084_ALERT_3_C High wR2 Value (i.e. > 0.25) ..................       0.27 Report
PLAT148_ALERT_3_C s.u. on the      a    - Axis is (Too) Large ....      0.020 Ang.
PLAT148_ALERT_3_C s.u. on the      c    - Axis is (Too) Large ....      0.008 Ang.
PLAT213_ALERT_2_C Atom C2              has ADP max/min Ratio .....        3.8 prolat
PLAT350_ALERT_3_C Short  C-H (X0.96,N1.08A)  C1       - H1       .       0.93 Ang.
PLAT350_ALERT_3_C Short  C-H (X0.96,N1.08A)  C2       - H2       .       0.93 Ang.
PLAT350_ALERT_3_C Short  C-H (X0.96,N1.08A)  C3       - H3       .       0.93 Ang.
PLAT350_ALERT_3_C Short  C-H (X0.96,N1.08A)  C4       - H4       .       0.93 Ang.
PLAT906_ALERT_3_C Large K Value in the Analysis of Variance ......      8.590 Check
PLAT913_ALERT_3_C Missing # of Very Strong Reflections in FCF ....          7 Note
                1  0  0,   0  2  0,  -1  1  1,  -2  0  2,  -1  0  2,  -2  1  2,
               -1  1  2,
PLAT918_ALERT_3_C Reflection(s) with I(obs) much Smaller I(calc) .          2 Check
PLAT939_ALERT_3_C Large Value of Not (SHELXL) Weight Optimized S .      25.73 Check
```

Alert level G

```
ABSMU01_ALERT_1_G  Calculation of _exptl_absorpt_correction_mu
                not performed for this radiation type.
PLAT072_ALERT_2_G SHELXL First  Parameter in WGHT  Unusually Large       0.20 Report
PLAT073_ALERT_1_G H-atoms ref., but hydrogen treatment Reported as     constr Check
PLAT199_ALERT_1_G Reported _cell_measurement_temperature ..... (K)        293 Check
PLAT200_ALERT_1_G Reported   _diffrn_ambient_temperature ..... (K)        293 Check
PLAT432_ALERT_2_G Short Inter X...Y Contact  C1       ..C5       .       2.52 Ang.
                                            1-x,1/2+y,1/2-z  =      2_655 Check
PLAT432_ALERT_2_G Short Inter X...Y Contact  C1       ..C5       .       2.67 Ang.
                                             x,3/2-y,-1/2+z  =      4_575 Check
PLAT432_ALERT_2_G Short Inter X...Y Contact  C1       ..C2       .       2.68 Ang.
                                              x,3/2-y,1/2+z  =      4_576 Check
PLAT432_ALERT_2_G Short Inter X...Y Contact  C1       ..C4       .       2.71 Ang.
                                                   x,1+y,z  =      1_565 Check
PLAT432_ALERT_2_G Short Inter X...Y Contact  C1       ..C4       .       2.71 Ang.
                                            1-x,1/2+y,1/2-z  =      2_655 Check
PLAT432_ALERT_2_G Short Inter X...Y Contact  C1       ..C1       .       2.79 Ang.
                                                1-x,2-y,1-z  =      3_676 Check
PLAT432_ALERT_2_G Short Inter X...Y Contact  C1       ..C3       .       2.93 Ang.
                                            1-x,1/2+y,1/2-z  =      2_655 Check
PLAT432_ALERT_2_G Short Inter X...Y Contact  C1       ..C5       .       3.02 Ang.
                                                   x,1+y,z  =      1_565 Check
PLAT432_ALERT_2_G Short Inter X...Y Contact  C1       ..C1       .       3.05 Ang.
                                            1-x,1/2+y,1/2-z  =      2_655 Check
PLAT432_ALERT_2_G Short Inter X...Y Contact  C1       ..C1       .       3.05 Ang.
                                           1-x,-1/2+y,1/2-z  =      2_645 Check
PLAT432_ALERT_2_G Short Inter X...Y Contact  C1       ..C1       .       3.09 Ang.
                                             x,3/2-y,-1/2+z  =      4_575 Check
PLAT432_ALERT_2_G Short Inter X...Y Contact  C1       ..C1       .       3.09 Ang.
                                              x,3/2-y,1/2+z  =      4_576 Check
PLAT432_ALERT_2_G Short Inter X...Y Contact  C1       ..C2       .       3.12 Ang.
                                            1-x,1/2+y,1/2-z  =      2_655 Check
PLAT432_ALERT_2_G Short Inter X...Y Contact  C2       ..C3       .       2.68 Ang.
                                             -x,1/2+y,1/2-z  =      2_555 Check
PLAT432_ALERT_2_G Short Inter X...Y Contact  C2       ..C5       .       2.68 Ang.
                                            1-x,1/2+y,1/2-z  =      2_655 Check
PLAT432_ALERT_2_G Short Inter X...Y Contact  C2       ..C4       .       2.81 Ang.
```

```
                                              -x,1/2+y,1/2-z  =      2_555 Check
PLAT432_ALERT_2_G Short Inter X...Y Contact  C2       ..C5        .        2.86 Ang.
                                              x,3/2-y,-1/2+z  =      4_575 Check
PLAT432_ALERT_2_G Short Inter X...Y Contact  C2       ..C2        .        3.07 Ang.
                                              x,3/2-y,-1/2+z  =      4_575 Check
PLAT432_ALERT_2_G Short Inter X...Y Contact  C2       ..C2        .        3.07 Ang.
                                               x,3/2-y,1/2+z  =      4_576 Check
PLAT432_ALERT_2_G Short Inter X...Y Contact  C2       ..C4        .        3.09 Ang.
                                              x,3/2-y,-1/2+z  =      4_575 Check
PLAT432_ALERT_2_G Short Inter X...Y Contact  C2       ..C4        .        3.09 Ang.
                                                     x,1+y,z  =      1_565 Check
PLAT432_ALERT_2_G Short Inter X...Y Contact  C2       ..C3        .        3.11 Ang.
                                                   -x,1-y,-z  =      3_565 Check
PLAT432_ALERT_2_G Short Inter X...Y Contact  C3       ..C4        .        2.68 Ang.
                                              x,1/2-y,-1/2+z  =      4_565 Check
PLAT432_ALERT_2_G Short Inter X...Y Contact  C3       ..C3        .        2.85 Ang.
                                                   -x,1-y,-z  =      3_565 Check
PLAT432_ALERT_2_G Short Inter X...Y Contact  C3       ..C4        .        2.87 Ang.
                                              -x,1/2+y,1/2-z  =      2_555 Check
PLAT432_ALERT_2_G Short Inter X...Y Contact  C3       ..C5        .        3.03 Ang.
                                              x,1/2-y,-1/2+z  =      4_565 Check
PLAT432_ALERT_2_G Short Inter X...Y Contact  C3       ..C3        .        3.12 Ang.
                                             -x,-1/2+y,1/2-z  =      2_545 Check
PLAT432_ALERT_2_G Short Inter X...Y Contact  C3       ..C3        .        3.12 Ang.
                                              -x,1/2+y,1/2-z  =      2_555 Check
PLAT432_ALERT_2_G Short Inter X...Y Contact  C4       ..C5        .        2.95 Ang.
                                              x,1/2-y,-1/2+z  =      4_565 Check
PLAT432_ALERT_2_G Short Inter X...Y Contact  C4       ..C4        .        3.10 Ang.
                                               x,1/2-y,1/2+z  =      4_566 Check
PLAT432_ALERT_2_G Short Inter X...Y Contact  C4       ..C4        .        3.10 Ang.
                                              x,1/2-y,-1/2+z  =      4_565 Check
PLAT802_ALERT_4_G CIF Input Record(s) with more than 80 Characters          4 Info
PLAT910_ALERT_3_G Missing FCF Reflection(s) Below Theta(Min)[Deg]=       3.58 Note
               1  0  0,   1  1  0,  -1  1  1,   0  1  1,
PLAT912_ALERT_4_G Missing # of FCF Reflections Above STh/L=  0.600        136 Note
PLAT933_ALERT_2_G Number of HKL-OMIT Records in Embedded .res File          8 Note
              -3  7  1,   0  4  5,   1  1  3,   1  4  1,   3  0  4,   3  2  6,
               3  7  0,   4  4  5,
PLAT956_ALERT_1_G Calculated (ThMax) and Actual (FCF) Hmax Differ           4 Units
PLAT969_ALERT_5_G The 'Henn et al.' R-Factor-gap value ...........     39.619 Note
              Predicted wR2: Based on SigI**2  0.69 or SHELX Weight 25.23
PLAT978_ALERT_2_G Number C-C Bonds with Positive Residual Density.          1 Info
PLAT979_ALERT_1_G NoSpherA2 Scattering Factors Used .............     Please Note
```

```
   4 ALERT level A = Most likely a serious problem - resolve or explain
   7 ALERT level B = A potentially serious problem, consider carefully
  12 ALERT level C = Check. Ensure it is not caused by an omission or oversight
  44 ALERT level G = General information/check it is not something unexpected

   6 ALERT type 1 CIF construction/syntax error, inconsistent or missing data
  41 ALERT type 2 Indicator that the structure model may be wrong or deficient
  17 ALERT type 3 Indicator that the structure quality may be low
   2 ALERT type 4 Improvement, methodology, query or suggestion
   1 ALERT type 5 Informative message, check
```

Datablock: Naphthalene_50.7GPa_IAM

```
Bond precision:   C-C = 0.0225 A          Wavelength=0.37380

Cell:               a=5.93(2)      b=4.913(2)          c=6.035(11)
                    alpha=90       beta=103.3(3)       gamma=90
Temperature:        293 K

                 Calculated                  Reported
Volume           171.1(7)                    171.2(8)
Space group      P 21/c                      P 1 21/c 1
Hall group       -P 2ybc                     -P 2ybc
Moiety formula   C10 H8                      C10 H8
Sum formula      C10 H8                      C10 H8
Mr               128.16                      128.16
Dx,g cm-3        2.488                       2.486
Z                2                           2
Mu (mm-1)        0.061                       0.061
F000             136.0                       136.0
F000'            135.97
h,k,lmax         8,6,8                       5,6,7
Nref             461                         122
Tmin,Tmax        0.999,0.999                 0.336,1.000
Tmin'            0.999

Correction method= # Reported T Limits: Tmin=0.336 Tmax=1.000
AbsCorr = MULTI-SCAN

Data completeness= 0.265           Theta(max)= 14.834

R(reflections)= 0.0888( 67)                    wR2(reflections)=
                                               0.2704( 122)
S = 1.180                  Npar= 46
```

The following ALERTS were generated. Each ALERT has the format
test-name_ALERT_alert-type_alert-level.
Click on the hyperlinks for more details of the test.

Alert level A

PLAT029_ALERT_3_A _diffrn_measured_fraction_theta_full value Low .   0.277 Why?

Author Response: This measurement was performed at high pressure which, due to the high

```
PLAT411_ALERT_2_A Short  Inter H...H Contact  H3        ..H4        .        1.52 Ang.
                                              -x,1/2+y,1/2-z   =      2_555 Check
```

Author Response: H atoms are close to each other at high pressure.

Alert level B

```
PLAT149_ALERT_3_B s.u. on the   beta    Angle is Too Large .......       0.30 Degree
```

Author Response: Large errors due to low completeness.

```
PLAT340_ALERT_3_B Low Bond Precision on  C-C Bonds ...............     0.0225 Ang.
```

Author Response: Large errors due to low completeness.

```
PLAT411_ALERT_2_B Short  Inter H...H Contact  H1        ..H4        .        1.87 Ang.
                                                     x,1+y,z   =      1_565 Check
```

Author Response: H atoms are close to each other at high pressure.

```
PLAT411_ALERT_2_B Short  Inter H...H Contact  H1        ..H1        .        1.95 Ang.
                                                 1-x,2-y,1-z   =      3_676 Check
```

Author Response: H atoms are close to each other at high pressure.

```
PLAT411_ALERT_2_B Short  Inter H...H Contact  H2        ..H3        .        1.84 Ang.
                                                   -x,1-y,-z   =      3_565 Check
```

Author Response: H atoms are close to each other at high pressure.

```
PLAT411_ALERT_2_B Short  Inter H...H Contact  H3        ..H3        .        1.80 Ang.
                                                   -x,1-y,-z   =      3_565 Check
```

Author Response: H atoms are close to each other at high pressure.

Alert level C

```
PLAT084_ALERT_3_C High wR2 Value (i.e. > 0.25) ...................       0.27 Report
PLAT148_ALERT_3_C s.u. on the      a    - Axis is (Too) Large ....      0.020 Ang.
PLAT148_ALERT_3_C s.u. on the      c    - Axis is (Too) Large ....      0.011 Ang.
PLAT234_ALERT_4_C Large Hirshfeld Difference C1       --C2        .      0.17 Ang.
PLAT241_ALERT_2_C High   'MainMol' Ueq as Compared to Neighbors of         C2 Check
PLAT411_ALERT_2_C Short  Inter H...H Contact  H2        ..H3        .        2.05 Ang.
                                              -x,1/2+y,1/2-z   =      2_555 Check
```

Author Response: H atoms are close to each other at high pressure.

```
PLAT411_ALERT_2_C Short Inter H...H Contact  H3       ..H4       .       2.06 Ang.
                                             x,1/2-y,-1/2+z  =      4_565 Check
```

Author Response: H atoms are close to each other at high pressure.

Alert level G

```
ABSMU01_ALERT_1_G  Calculation of _exptl_absorpt_correction_mu
                not performed for this radiation type.
PLAT003_ALERT_2_G Number of Uiso or U(i,j)  Restrained non-H-Atoms         2 Report
PLAT012_ALERT_1_G N.O.K.   _shelx_res_checksum Found in CIF ......     Please Check
PLAT072_ALERT_2_G SHELXL First  Parameter in WGHT  Unusually Large      0.20 Report
PLAT186_ALERT_4_G The CIF-Embedded .res File Contains ISOR Records         2 Report
PLAT199_ALERT_1_G Reported _cell_measurement_temperature ..... (K)       293 Check
PLAT200_ALERT_1_G Reported   _diffrn_ambient_temperature ..... (K)       293 Check
PLAT432_ALERT_2_G Short Inter X...Y Contact  C1       ..C5       .       2.46 Ang.
                                            1-x,1/2+y,1/2-z  =      2_655 Check
PLAT432_ALERT_2_G Short Inter X...Y Contact  C1       ..C5       .       2.57 Ang.
                                             x,3/2-y,-1/2+z  =      4_575 Check
PLAT432_ALERT_2_G Short Inter X...Y Contact  C1       ..C4       .       2.61 Ang.
                                            1-x,1/2+y,1/2-z  =      2_655 Check
PLAT432_ALERT_2_G Short Inter X...Y Contact  C1       ..C4       .       2.68 Ang.
                                                     x,1+y,z  =      1_565 Check
PLAT432_ALERT_2_G Short Inter X...Y Contact  C1       ..C2       .       2.71 Ang.
                                              x,3/2-y,1/2+z  =      4_576 Check
PLAT432_ALERT_2_G Short Inter X...Y Contact  C1       ..C1       .       2.76 Ang.
                                                 1-x,2-y,1-z  =      3_676 Check
PLAT432_ALERT_2_G Short Inter X...Y Contact  C1       ..C3       .       2.90 Ang.
                                            1-x,1/2+y,1/2-z  =      2_655 Check
PLAT432_ALERT_2_G Short Inter X...Y Contact  C1       ..C1       .       2.95 Ang.
                                            1-x,1/2+y,1/2-z  =      2_655 Check
PLAT432_ALERT_2_G Short Inter X...Y Contact  C1       ..C1       .       2.95 Ang.
                                           1-x,-1/2+y,1/2-z  =      2_645 Check
PLAT432_ALERT_2_G Short Inter X...Y Contact  C1       ..C5       .       2.99 Ang.
                                                     x,1+y,z  =      1_565 Check
PLAT432_ALERT_2_G Short Inter X...Y Contact  C1       ..C2       .       3.03 Ang.
                                            1-x,1/2+y,1/2-z  =      2_655 Check
PLAT432_ALERT_2_G Short Inter X...Y Contact  C1       ..C1       .       3.06 Ang.
                                             x,3/2-y,-1/2+z  =      4_575 Check
PLAT432_ALERT_2_G Short Inter X...Y Contact  C1       ..C1       .       3.06 Ang.
                                              x,3/2-y,1/2+z  =      4_576 Check
PLAT432_ALERT_2_G Short Inter X...Y Contact  C2       ..C3       .       2.55 Ang.
                                             -x,1/2+y,1/2-z  =      2_555 Check
PLAT432_ALERT_2_G Short Inter X...Y Contact  C2       ..C5       .       2.71 Ang.
                                            1-x,1/2+y,1/2-z  =      2_655 Check
PLAT432_ALERT_2_G Short Inter X...Y Contact  C2       ..C4       .       2.75 Ang.
                                             -x,1/2+y,1/2-z  =      2_555 Check
PLAT432_ALERT_2_G Short Inter X...Y Contact  C2       ..C5       .       2.82 Ang.
                                             x,3/2-y,-1/2+z  =      4_575 Check
PLAT432_ALERT_2_G Short Inter X...Y Contact  C2       ..C2       .       3.04 Ang.
                                             x,3/2-y,-1/2+z  =      4_575 Check
```

```
PLAT432_ALERT_2_G Short Inter X...Y Contact  C2       ..C2       .       3.04 Ang.
                                              x,3/2-y,1/2+z  =     4_576 Check
PLAT432_ALERT_2_G Short Inter X...Y Contact  C2       ..C3       .       3.05 Ang.
                                                   -x,1-y,-z  =     3_565 Check
PLAT432_ALERT_2_G Short Inter X...Y Contact  C2       ..C4       .       3.07 Ang.
                                                     x,1+y,z  =     1_565 Check
PLAT432_ALERT_2_G Short Inter X...Y Contact  C2       ..C4       .       3.09 Ang.
                                             x,3/2-y,-1/2+z  =     4_575 Check
PLAT432_ALERT_2_G Short Inter X...Y Contact  C3       ..C4       .       2.70 Ang.
                                             x,1/2-y,-1/2+z  =     4_565 Check
PLAT432_ALERT_2_G Short Inter X...Y Contact  C3       ..C4       .       2.77 Ang.
                                             -x,1/2+y,1/2-z  =     2_555 Check
PLAT432_ALERT_2_G Short Inter X...Y Contact  C3       ..C3       .       2.82 Ang.
                                                   -x,1-y,-z  =     3_565 Check
PLAT432_ALERT_2_G Short Inter X...Y Contact  C3       ..C3       .       2.98 Ang.
                                            -x,-1/2+y,1/2-z  =     2_545 Check
PLAT432_ALERT_2_G Short Inter X...Y Contact  C3       ..C3       .       2.98 Ang.
                                             -x,1/2+y,1/2-z  =     2_555 Check
PLAT432_ALERT_2_G Short Inter X...Y Contact  C3       ..C5       .       3.02 Ang.
                                             x,1/2-y,-1/2+z  =     4_565 Check
PLAT432_ALERT_2_G Short Inter X...Y Contact  C4       ..C5       .       2.87 Ang.
                                             x,1/2-y,-1/2+z  =     4_565 Check
PLAT432_ALERT_2_G Short Inter X...Y Contact  C4       ..C4       .       3.06 Ang.
                                              x,1/2-y,1/2+z  =     4_566 Check
PLAT432_ALERT_2_G Short Inter X...Y Contact  C4       ..C4       .       3.06 Ang.
                                             x,1/2-y,-1/2+z  =     4_565 Check
PLAT802_ALERT_4_G CIF Input Record(s) with more than 80 Characters          3 Info
PLAT860_ALERT_3_G Number of Least-Squares Restraints ............          12 Note
PLAT883_ALERT_1_G Absent Datum for _atom_sites_solution_primary ..     Please Do !
PLAT933_ALERT_2_G Number of HKL-OMIT Records in Embedded .res File          5 Note
                0  6  2,   0  6  3,   1  3  0,   2  1  2,   3  0  4,
PLAT941_ALERT_3_G Average HKL Measurement Multiplicity ..........        1.3 Low
PLAT950_ALERT_5_G Calculated (ThMax) and CIF-Reported Hmax Differ           3 Units
```

```
  2 ALERT level A = Most likely a serious problem - resolve or explain
  6 ALERT level B = A potentially serious problem, consider carefully
  7 ALERT level C = Check. Ensure it is not caused by an omission or oversight
 44 ALERT level G = General information/check it is not something unexpected

  5 ALERT type 1 CIF construction/syntax error, inconsistent or missing data
 42 ALERT type 2 Indicator that the structure model may be wrong or deficient
  8 ALERT type 3 Indicator that the structure quality may be low
  3 ALERT type 4 Improvement, methodology, query or suggestion
  1 ALERT type 5 Informative message, check
```

It is advisable to attempt to resolve as many as possible of the alerts in all categories. Often the minor alerts point to easily fixed oversights, errors and omissions in your CIF or refinement strategy, so attention to these fine details can be worthwhile. It is up to the individual to critically assess their own results and, if necessary, seek expert advice.

PLATON version of 04/06/2025; check.def file version of 30/05/2025

Datablock Naphthalene_00.0GPa_IAM - ellipsoid plot

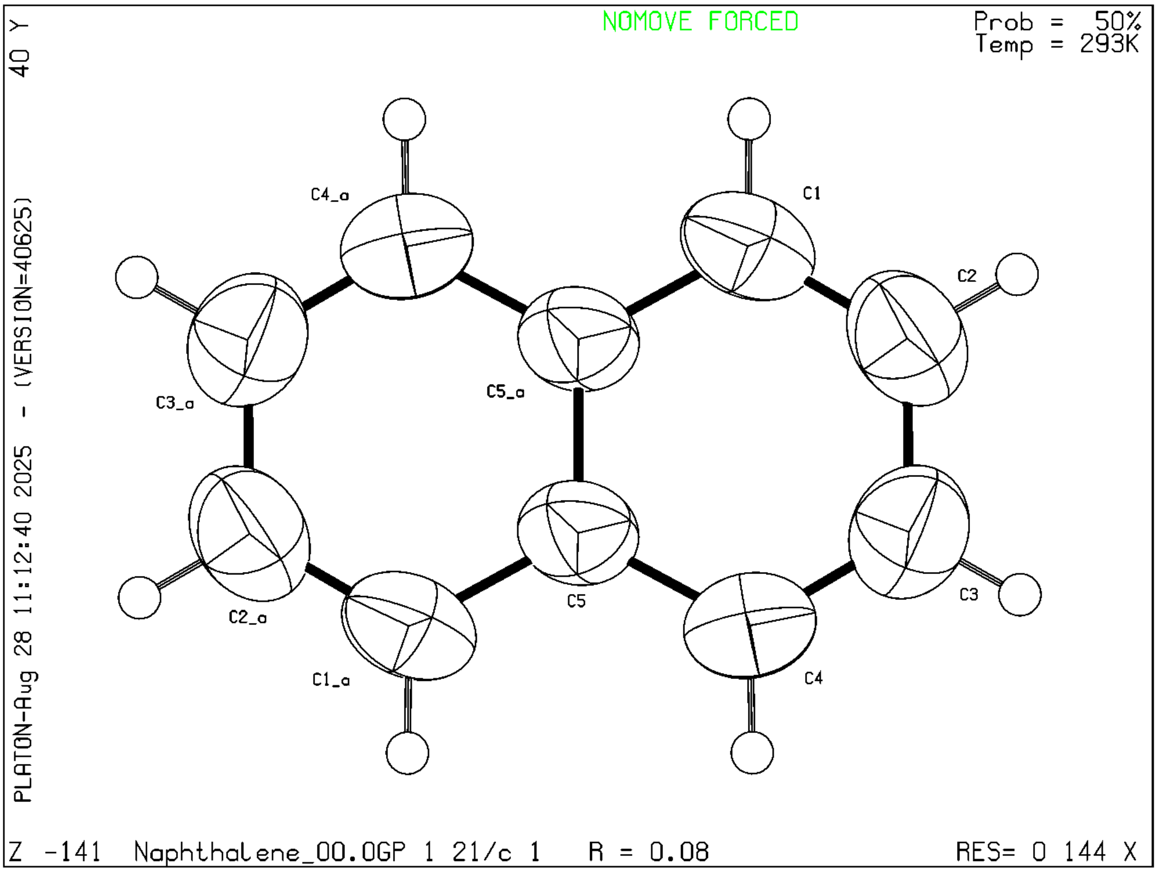

Datablock Naphthalene_06.2GPa_IAM - ellipsoid plot

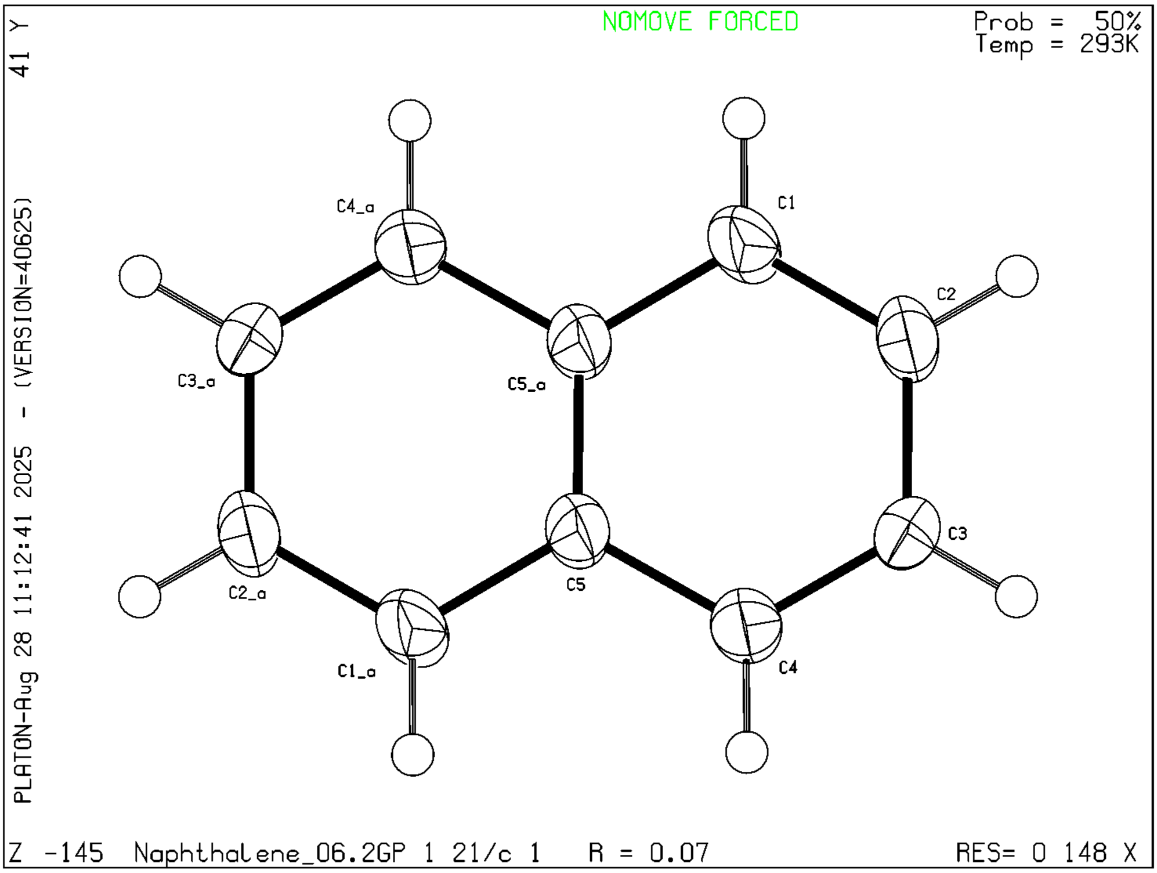

Datablock Naphthalene_09.9GPa_IAM - ellipsoid plot

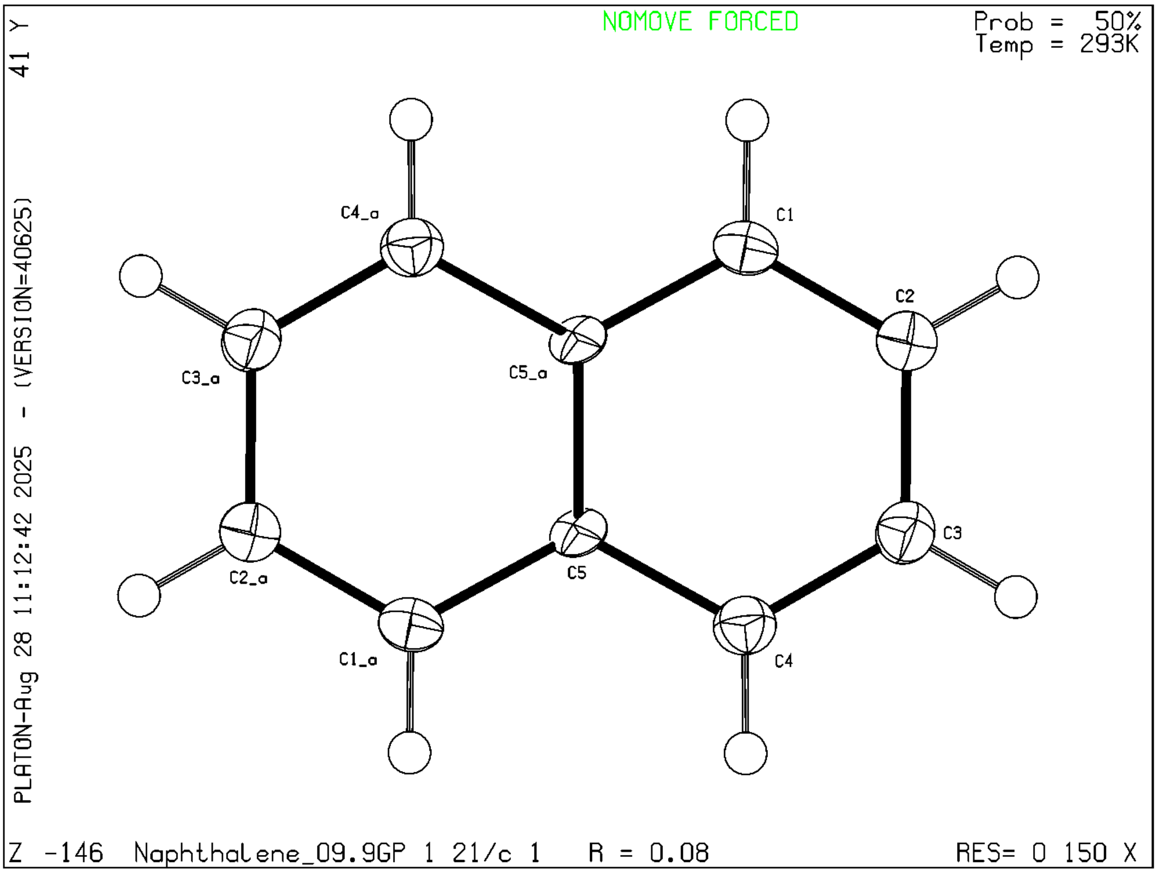

Datablock Naphthalene_10.8GPa_IAM - ellipsoid plot

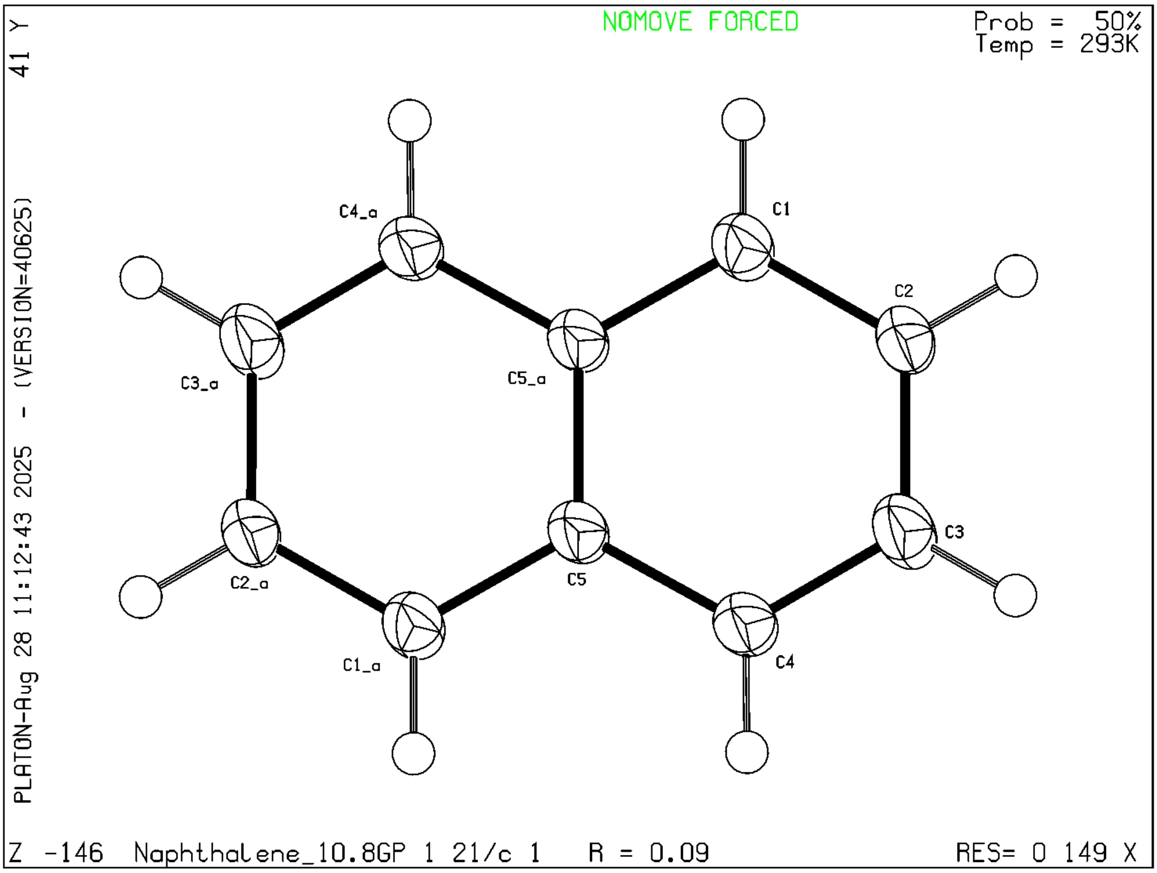

Datablock Naphthalene_14.8GPa_IAM - ellipsoid plot

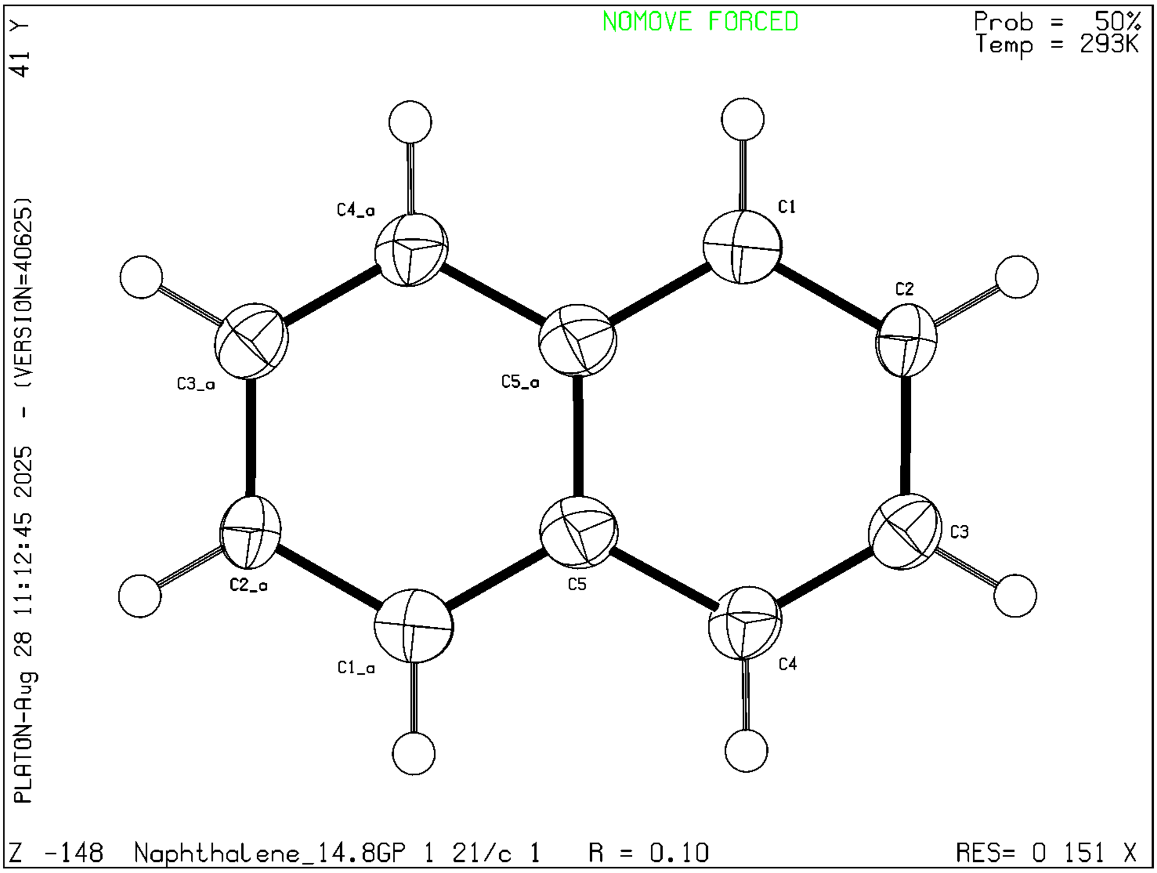

Datablock Naphthalene_20.3GPa_IAM - ellipsoid plot

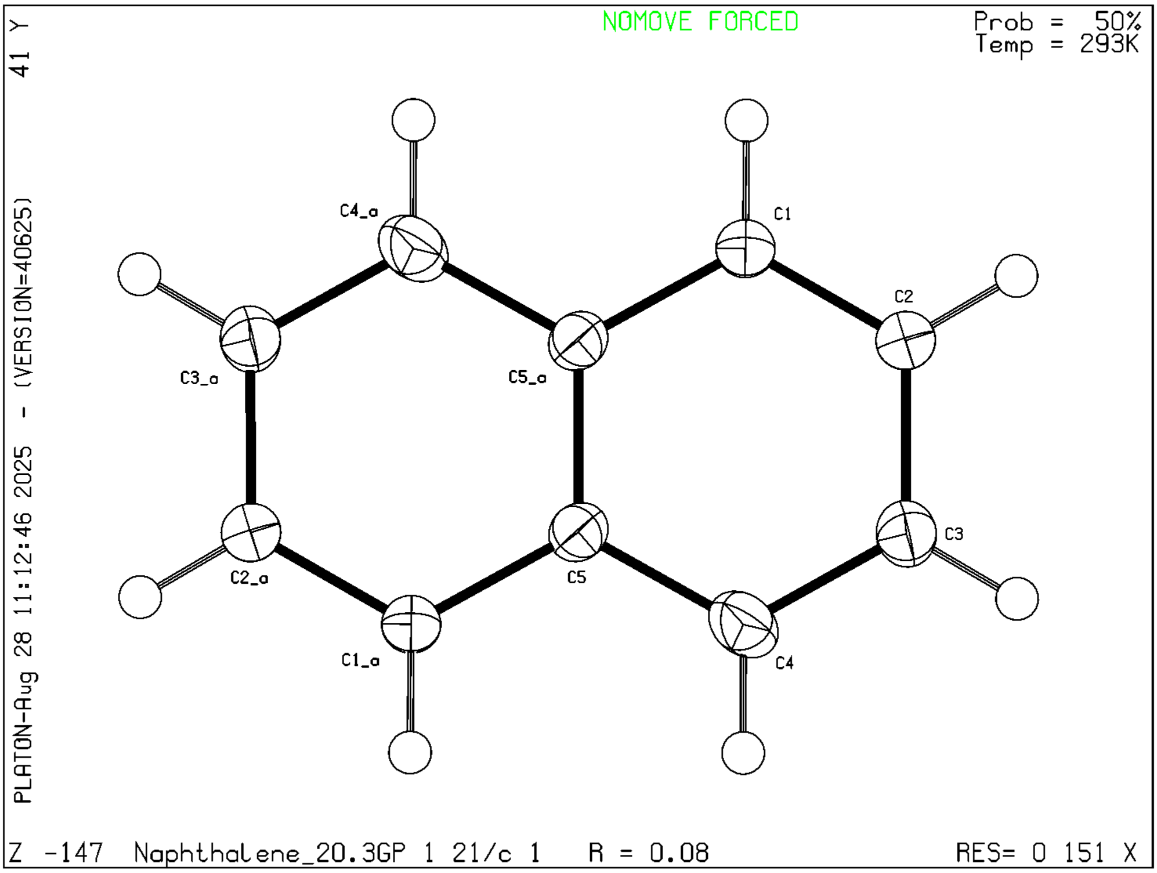

Datablock Naphthalene_24.5GPa_IAM - ellipsoid plot

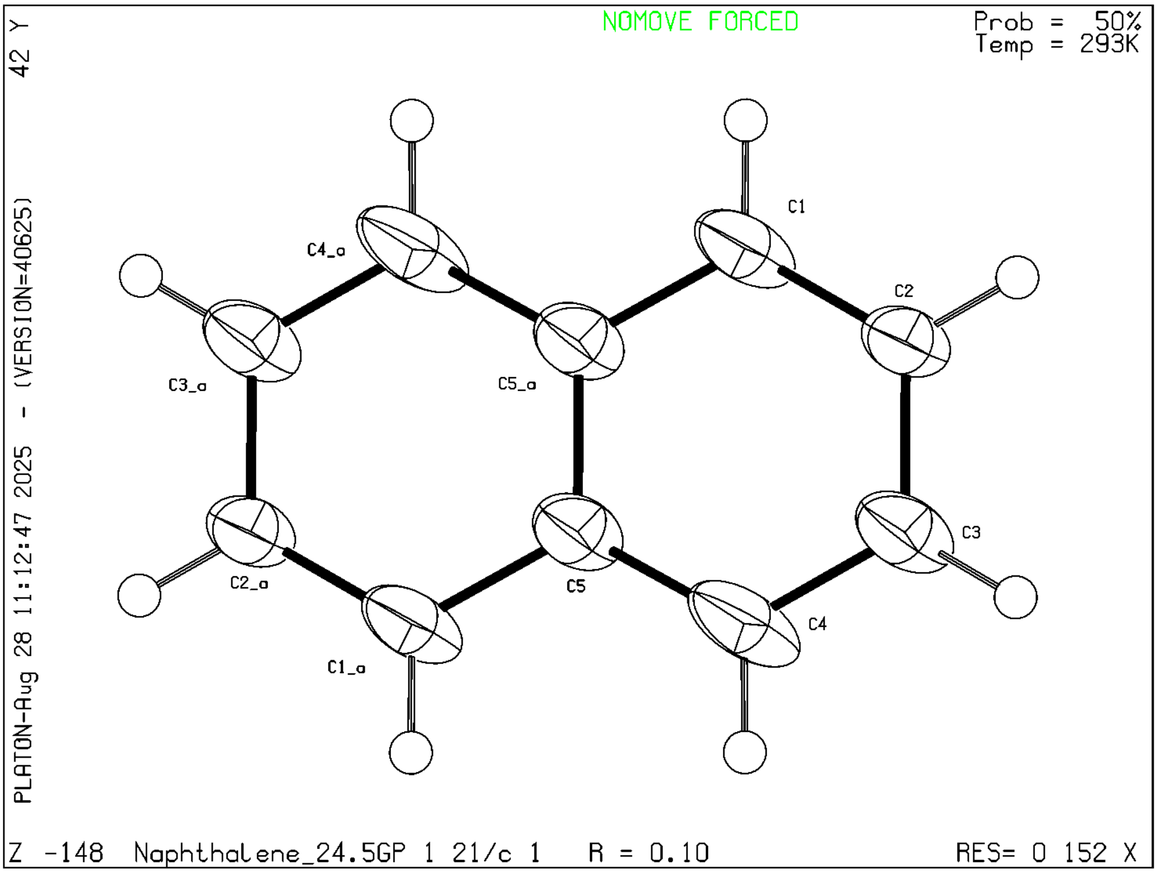

Datablock Naphthalene_28.5GPa_IAM - ellipsoid plot

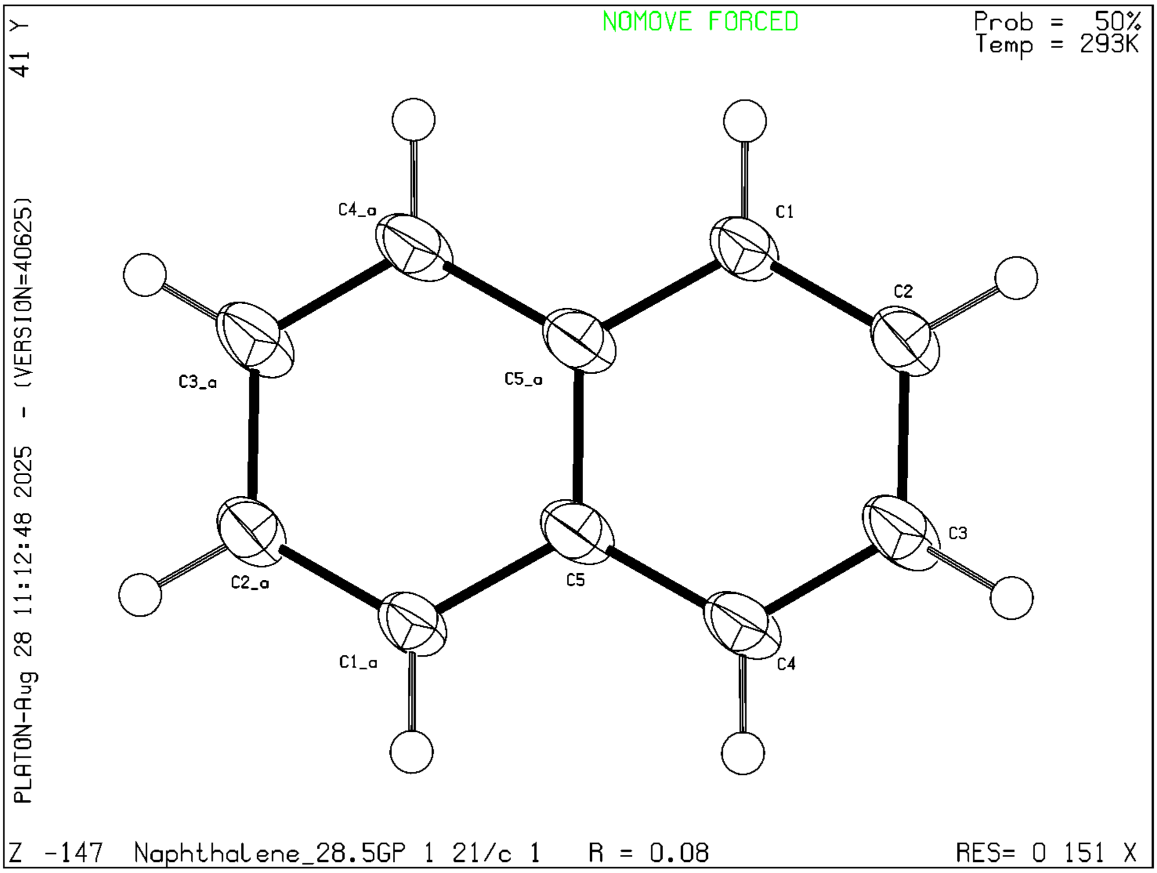

Datablock Naphthalene_35.1GPa_IAM - ellipsoid plot

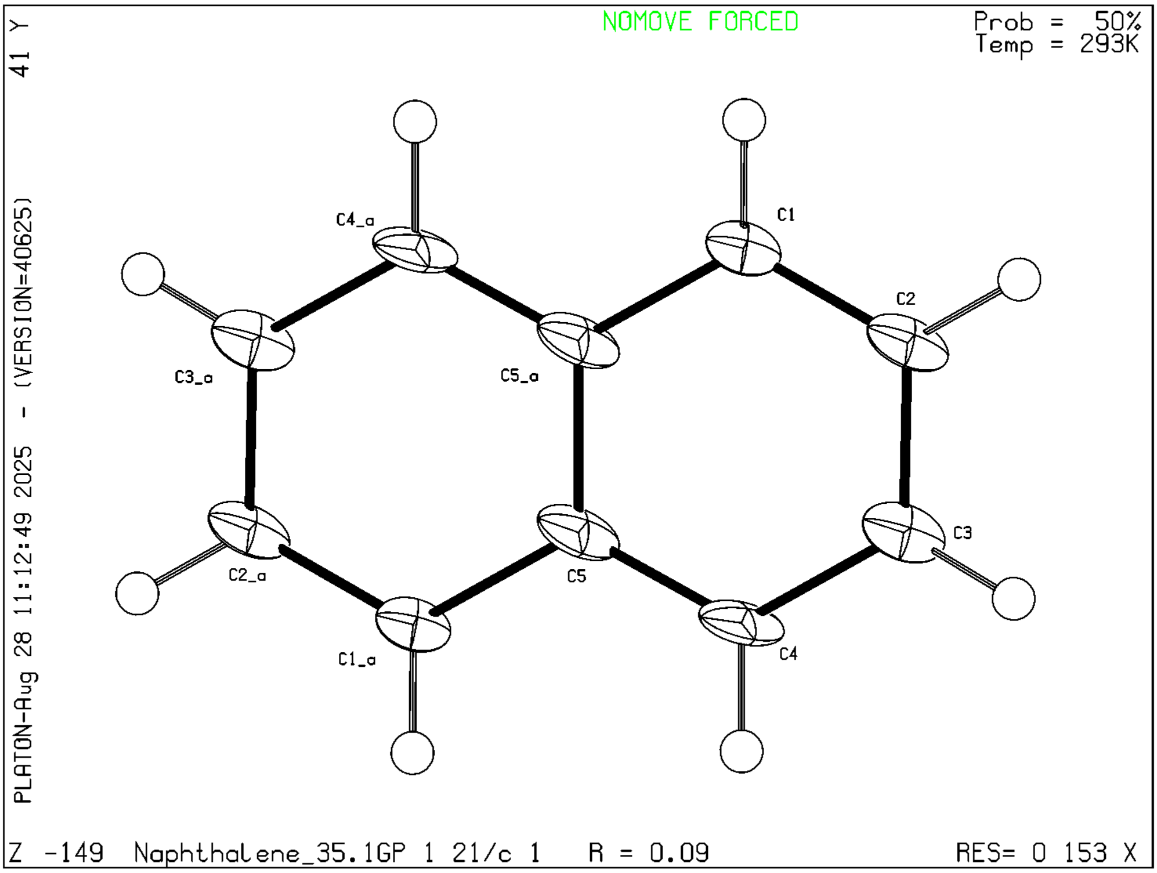

Datablock Naphthalene_43.3GPa_IAM - ellipsoid plot

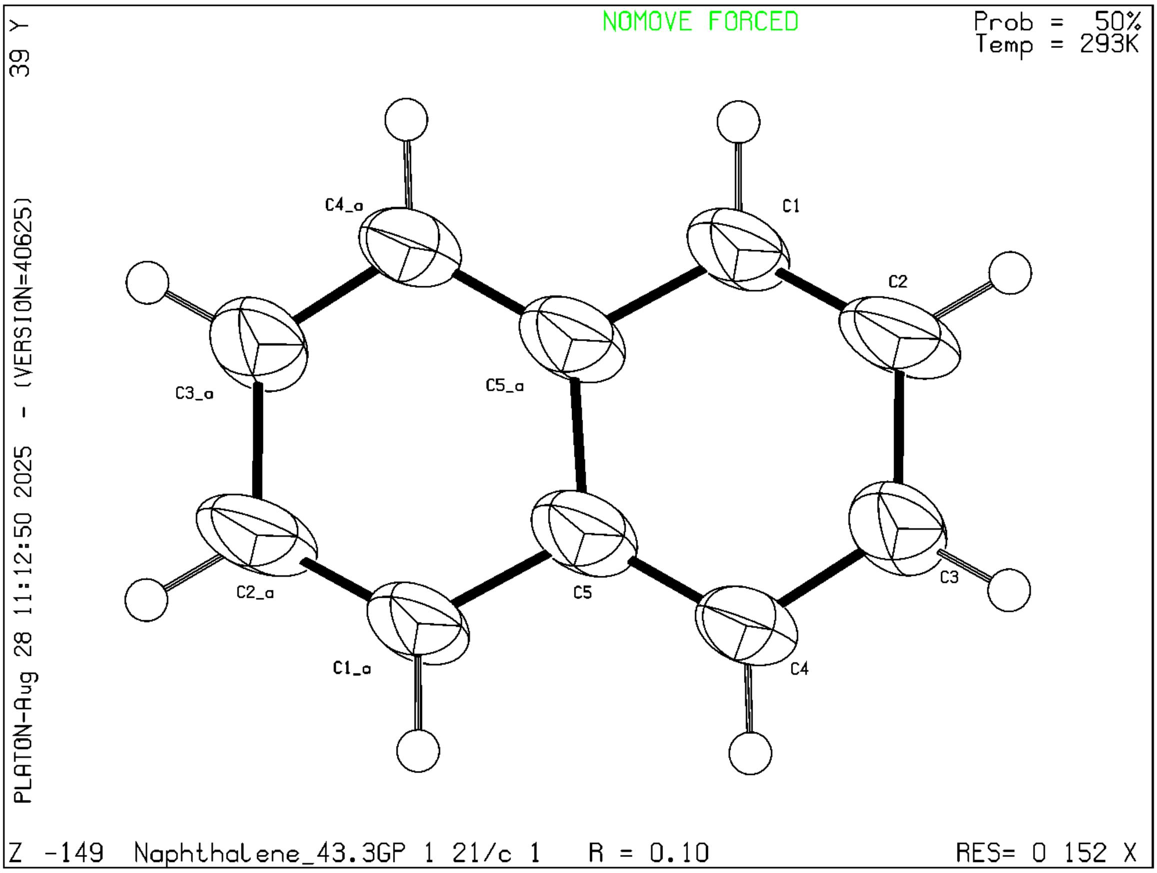

Datablock Naphthalene_46.6GPa_IAM - ellipsoid plot

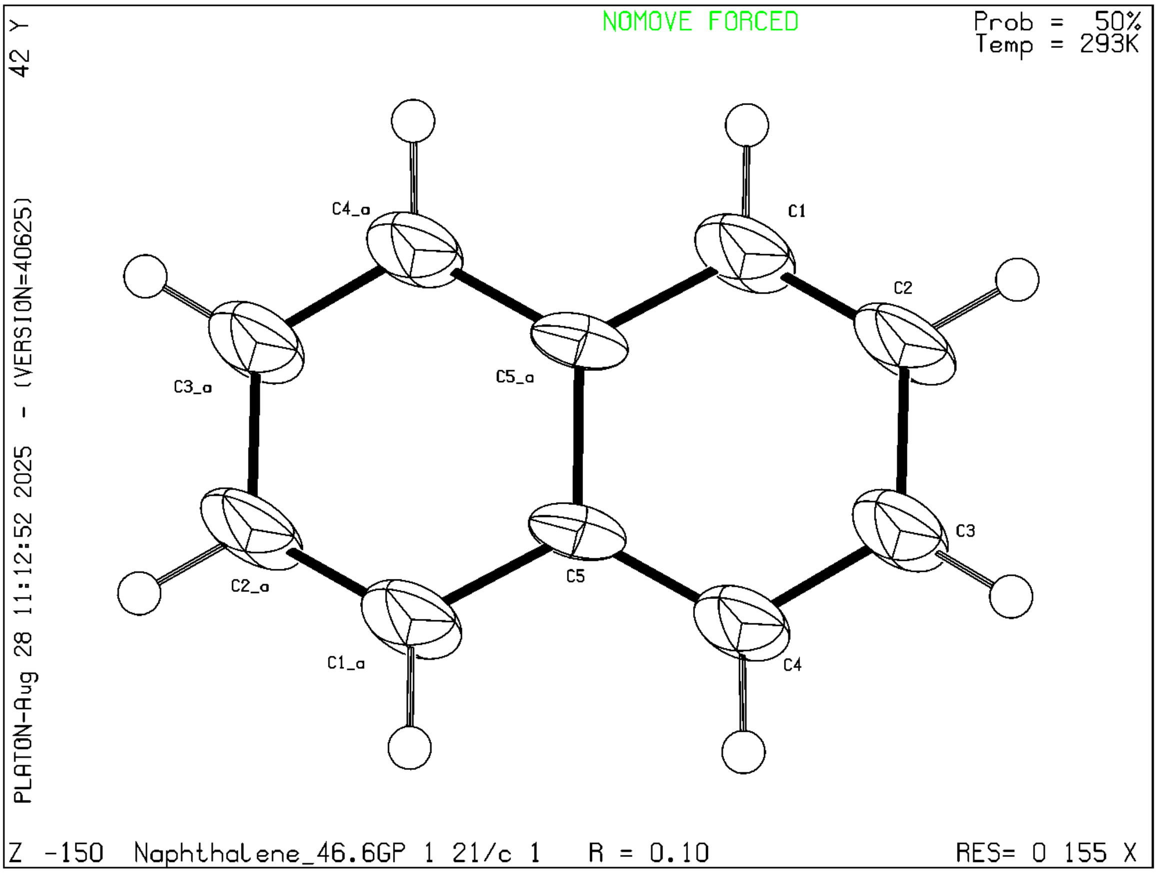

Datablock Naphthalene_50.7GPa_IAM - ellipsoid plot

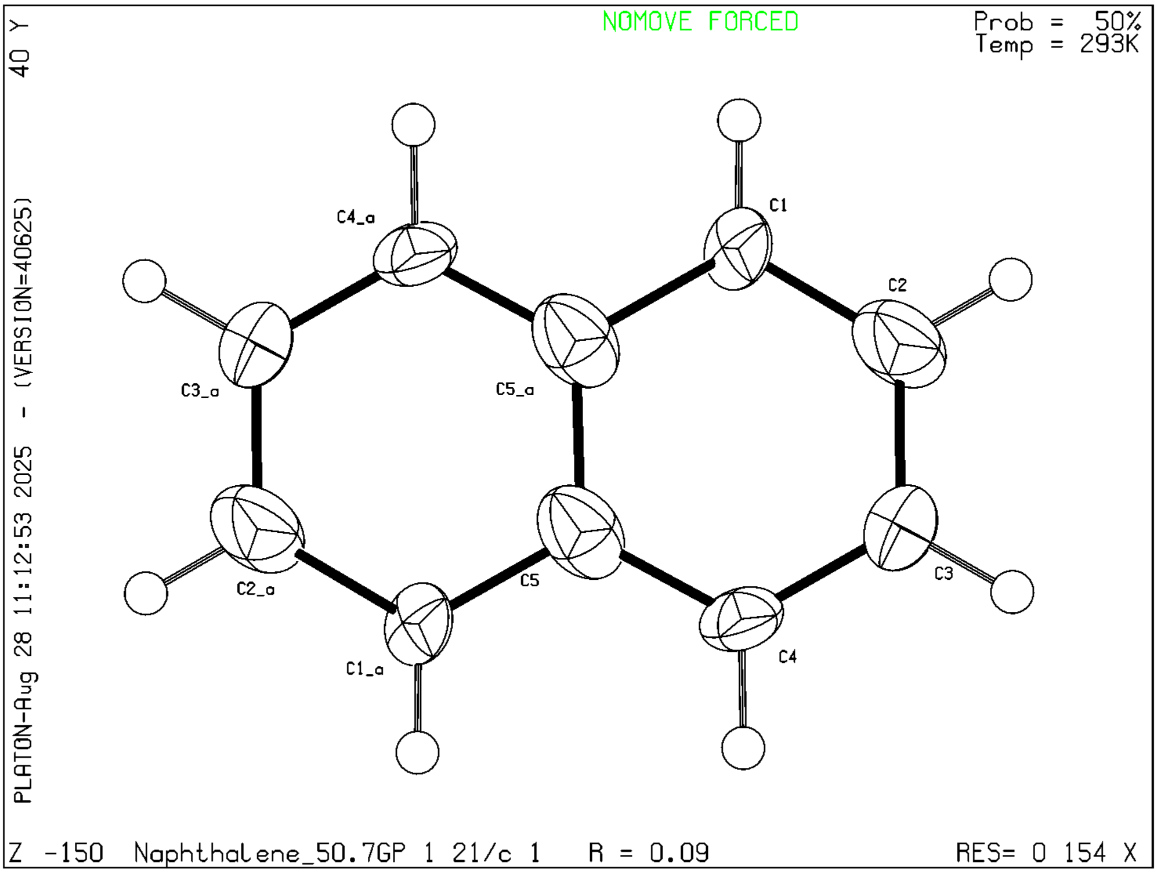

Supplement: Supplementary file 15 [file ao5c06935_si_015.pdf]
