# Supplementary material for: Compressional Behavior of Naphthalene (C10H8) and Anthracene (C14H10) up to 50 GPa
Source: ACS Omega. 2025 Oct 19;10(42):50230–42. doi: 10.1021/acsomega.5c06935 (PMC12573047; doi:10.1021/acsomega.5c06935)

checkCIF/PLATON report

Structure factors have been supplied for datablock(s) Anthracene_00.0GPa_IAM, Anthracene_01.5GPa_IAM, Anthracene_04.0GPa_IAM, Anthracene_08.3GPa_IAM, Anthracene_10.8GPa_IAM, Anthracene_13.5GPa_IAM, Anthracene_15.8GPa_IAM, Anthracene_18.4GPa_IAM, Anthracene_21.5GPa_IAM, Anthracene_25.3GPa_IAM, Anthracene_29.0GPa_IAM, Anthracene_34.0GPa_IAM, Anthracene_35.5GPa_IAM, Anthracene_38.6GPa_IAM, Anthracene_42.3GPa_IAM

THIS REPORT IS FOR GUIDANCE ONLY. IF USED AS PART OF A REVIEW PROCEDURE FOR PUBLICATION, IT SHOULD NOT REPLACE THE EXPERTISE OF AN EXPERIENCED CRYSTALLOGRAPHIC REFEREE.

No syntax errors found. CIF dictionary Interpreting this report

Datablock: Anthracene_00.0GPa_IAM

```
Bond precision:   C-C = 0.0071 A          Wavelength=0.37380

Cell:              a=9.488(5)      b=6.0253(3)         c=8.5642(14)
                   alpha=90        beta=103.52(3)      gamma=90
Temperature:       293 K

                 Calculated                    Reported
Volume           476.0(3)                      476.0(3)
Space group      P 21/c                        P 1 21/c 1
Hall group       -P 2ybc                       -P 2ybc
Moiety formula   C14 H10                       C14 H10
Sum formula      C14 H10                       C14 H10
Mr               178.22                        178.22
Dx,g cm-3        1.243                         1.243
Z                2                             2
Mu (mm-1)        0.031                         0.032
F000             188.0                         188.0
F000'            187.97
h,k,lmax         13,8,12                       9,8,12
Nref             1442                          514
Tmin,Tmax        1.000,1.000                   0.458,1.000
Tmin'            1.000
```

Correction method= # Reported T Limits: Tmin=0.458 Tmax=1.000
AbsCorr = MULTI-SCAN

Data completeness= 0.356 Theta(max)= 15.455

R(reflections)= 0.0403(354) wR2(reflections)= 0.1327(514)

S = 1.020 Npar= 64

The following ALERTS were generated. Each ALERT has the format
test-name_ALERT_alert-type_alert-level.
Click on the hyperlinks for more details of the test.

Alert level A

PLAT029_ALERT_3_A _diffrn_measured_fraction_theta_full value Low . 0.367 Why?

Author Response: This measurement was performed at high pressure which, due to the high

PLAT901_ALERT_1_A Cell Parameters in CIF and FCF do not Match .... ! Error

---

Alert level C

PLAT234_ALERT_4_C Large Hirshfeld Difference C002 --C005 . 0.18 Ang.
PLAT242_ALERT_2_C Low 'MainMol' Ueq as Compared to Neighbors of C005 Check
PLAT340_ALERT_3_C Low Bond Precision on C-C Bonds .... 0.00713 Ang.

Alert level G

ABSMU01_ALERT_1_G Calculation of _exptl_absorpt_correction_mu
not performed for this radiation type.
PLAT012_ALERT_1_G N.O.K. _shelx_res_checksum Found in CIF ...... Please Check
PLAT199_ALERT_1_G Reported _cell_measurement_temperature ..... (K) 293 Check
PLAT200_ALERT_1_G Reported _diffrn_ambient_temperature ..... (K) 293 Check
PLAT333_ALERT_2_G Large Aver C6-Ring C-C Dist C001 -C004_a . 1.42 Ang.
PLAT333_ALERT_2_G Large Aver C6-Ring C-C Dist C002 -C005 . 1.42 Ang.
PLAT720_ALERT_4_G Number of Unusual/Non-Standard Labels .... 12 Note
C001 H001 C002 H002 C003 H003 C004 C005
C006 H006 C007 H007
PLAT802_ALERT_4_G CIF Input Record(s) with more than 80 Characters 2 Info
PLAT883_ALERT_1_G Absent Datum for _atom_sites_solution_primary .. Please Do !
PLAT933_ALERT_2_G Number of HKL-OMIT Records in Embedded .res File 1 Note
-1 0 6,
PLAT941_ALERT_3_G Average HKL Measurement Multiplicity .... 1.8 Low
PLAT950_ALERT_5_G Calculated (ThMax) and CIF-Reported Hmax Differ 4 Units

---

2 **ALERT level A** = Most likely a serious problem - resolve or explain
0 **ALERT level B** = A potentially serious problem, consider carefully
3 **ALERT level C** = Check. Ensure it is not caused by an omission or oversight
12 **ALERT level G** = General information/check it is not something unexpected

```
   6 ALERT type 1 CIF construction/syntax error, inconsistent or missing data
   4 ALERT type 2 Indicator that the structure model may be wrong or deficient
   3 ALERT type 3 Indicator that the structure quality may be low
   3 ALERT type 4 Improvement, methodology, query or suggestion
   1 ALERT type 5 Informative message, check
```

Datablock: Anthracene_01.5GPa_IAM

```
Bond precision:    C-C = 0.0141 A           Wavelength=0.41000

Cell:              a=8.0741(10)     b=5.8283(7)        c=9.032(11)
                   alpha=90         beta=101.44(4)     gamma=90
Temperature:       293 K
```

	Calculated	Reported
Volume	416.6(5)	416.6(5)
Space group	P 21/c	P 1 21/c 1
Hall group	-P 2ybc	-P 2ybc
Moiety formula	C14 H10	C14 H10
Sum formula	C14 H10	C14 H10
Mr	178.22	178.22
Dx,g cm-3	1.421	1.421
Z	2	2
Mu (mm-1)	0.038	0.037
F000	188.0	188.0
F000’	187.97	
h,k,lmax	10,7,11	5,7,10
Nref	1000	336
Tmin,Tmax	1.000,1.000	0.661,1.000
Tmin’	1.000	

```
Correction method= # Reported T Limits: Tmin=0.661 Tmax=1.000
AbsCorr = MULTI-SCAN

Data completeness= 0.336           Theta(max)= 15.711

R(reflections)= 0.0591( 163)                      wR2(reflections)=
                                                  0.2379( 336)
S = 0.704                    Npar= 64
```

```
The following ALERTS were generated. Each ALERT has the format
       test-name_ALERT_alert-type_alert-level.
Click on the hyperlinks for more details of the test.
```

Alert level A

```
PLAT029_ALERT_3_A _diffrn_measured_fraction_theta_full value Low .      0.395 Why?
```

Author Response: This measurement was performed at high pressure which, due to the high

```
PLAT088_ALERT_3_A Poor Data / Parameter Ratio ....................       5.25 Note
```

Author Response: This measurement was performed at high pressure which, due to the high

---

Alert level B

```
PLAT340_ALERT_3_B Low Bond Precision on  C-C Bonds ...............    0.01413 Ang.
```

Author Response: Large errors due to low completeness.

```
PLAT911_ALERT_3_B Missing FCF Refl Between Thmin & STh/L=    0.600        270 Report
                2  0  0,   3  0  0,   4  0  0,   5  0  0,   1  1  0,   2  1  0,
                3  1  0,   4  1  0,   5  1  0,   1  2  0,   2  2  0,   3  2  0,
                4  2  0,   5  2  0,   2  3  0,   3  3  0,   4  3  0,   5  3  0,
                3  4  0,   4  4  0,   5  4  0,   3  5  0,   4  5  0,   5  5  0,
                0  6  0,   4  6  0,  -5  1  1,  -4  1  1,  -3  1  1,  -2  1  1,
                   (  240 More Missing: see the .ckf listing file)
```

Author Response: This measurement was performed at high pressure which, due to the high

---

Alert level C

```
CRYSC01_ALERT_1_C No recognised colour has been given for crystal colour.
GOODF01_ALERT_2_C  The least squares goodness of fit parameter lies
            outside the range 0.80 <> 2.00
            Goodness of fit given =      0.704
PLAT026_ALERT_3_C Ratio Observed / Unique Reflections (too) Low ..        49% Check
PLAT148_ALERT_3_C s.u. on the      c    - Axis is (Too) Large ....      0.011 Ang.
PLAT906_ALERT_3_C Large K Value in the Analysis of Variance ......     19.551 Check
PLAT906_ALERT_3_C Large K Value in the Analysis of Variance ......      2.278 Check
```

Alert level G

```
ABSMU01_ALERT_1_G  Calculation of _exptl_absorpt_correction_mu
                not performed for this radiation type.
PLAT003_ALERT_2_G Number of Uiso or U(i,j)  Restrained non-H-Atoms          6 Report
PLAT012_ALERT_1_G N.O.K.   _shelx_res_checksum Found in CIF ......     Please Check
PLAT013_ALERT_1_G N.O.K.   _shelx_hkl_checksum Found in CIF ......     Please Check
PLAT186_ALERT_4_G The CIF-Embedded .res File Contains ISOR Records          1 Report
```

```
PLAT187_ALERT_4_G The CIF-Embedded .res File Contains RIGU Records          1 Report
PLAT199_ALERT_1_G Reported _cell_measurement_temperature ..... (K)        293 Check
PLAT200_ALERT_1_G Reported   _diffrn_ambient_temperature ..... (K)        293 Check
PLAT335_ALERT_2_G Check Large C6 Ring C-C Range C002     -C005          0.21 Ang.
PLAT720_ALERT_4_G Number of Unusual/Non-Standard Labels ..........         12 Note
              C001    H001    C002    H002    C003    H003    C004    C005
              C006    H006    C007    H007
PLAT802_ALERT_4_G CIF Input Record(s) with more than 80 Characters          3 Info
PLAT860_ALERT_3_G Number of Least-Squares Restraints ............          81 Note
PLAT883_ALERT_1_G Absent Datum for _atom_sites_solution_primary ..     Please Do !
PLAT910_ALERT_3_G Missing FCF Reflection(s) Below Theta(Min)[Deg]=       2.41 Note
                1  0  0,
PLAT912_ALERT_4_G Missing # of FCF Reflections Above STh/L=  0.600         83 Note
PLAT913_ALERT_3_G Missing # of Very Strong Reflections in FCF ....          1 Note
               -2  0  2,
PLAT933_ALERT_2_G Number of HKL-OMIT Records in Embedded .res File          7 Note
               -4  2  6,   0  4  4,   0  5  2,   0  6  1,   0  6  6,   1  4  3,
                1  5  7,
PLAT941_ALERT_3_G Average HKL Measurement Multiplicity ..........         2.2 Low
PLAT950_ALERT_5_G Calculated (ThMax) and CIF-Reported Hmax Differ           5 Units
PLAT956_ALERT_1_G Calculated (ThMax) and Actual (FCF) Hmax Differ           5 Units
PLAT963_ALERT_2_G Both SHELXL WEIGHT Parameter Values Zero .......     Please Check
PLAT969_ALERT_5_G The 'Henn et al.' R-Factor-gap value ..........      0.704 Note
              Predicted wR2: Based on SigI**2 33.79 or SHELX Weight 33.79
PLAT978_ALERT_2_G Number C-C Bonds with Positive Residual Density.          0 Info
```

---

2 **ALERT level A** = Most likely a serious problem - resolve or explain
2 **ALERT level B** = A potentially serious problem, consider carefully
6 **ALERT level C** = Check. Ensure it is not caused by an omission or oversight
23 **ALERT level G** = General information/check it is not something unexpected

8 ALERT type 1 CIF construction/syntax error, inconsistent or missing data
6 ALERT type 2 Indicator that the structure model may be wrong or deficient
12 ALERT type 3 Indicator that the structure quality may be low
5 ALERT type 4 Improvement, methodology, query or suggestion
2 ALERT type 5 Informative message, check

Datablock: Anthracene_04.0GPa_IAM

Bond precision:	C-C = 0.0214 A		Wavelength=0.41000
Cell:	a=8.747(11)	b=5.6633(7)	c=7.718(2)
	alpha=90	beta=99.35(7)	gamma=90
Temperature:	293 K		

	Calculated	Reported
Volume	377.3(5)	377.3(5)
Space group	P 21/c	P 1 21/c 1
Hall group	-P 2ybc	-P 2ybc
Moiety formula	C14 H10	C14 H10
Sum formula	C14 H10	C14 H10
Mr	178.22	178.22
Dx,g cm-3	1.569	1.569
Z	2	2
Mu (mm-1)	0.041	0.041
F000	188.0	188.0
F000'	187.97	
h,k,lmax	11,7,10	7,7,10
Nref	897	285
Tmin,Tmax	0.999,1.000	0.089,1.000
Tmin'	0.999	

Correction method= # Reported T Limits: Tmin=0.089 Tmax=1.000
AbsCorr = MULTI-SCAN

Data completeness= 0.318 Theta(max)= 15.633

R(reflections)= 0.0540(130) wR2(reflections)= 0.1318(285)

S = 0.953 Npar= 64

The following ALERTS were generated. Each ALERT has the format
test-name_ALERT_alert-type_alert-level.
Click on the hyperlinks for more details of the test.

Alert level A

PLAT029_ALERT_3_A _diffrn_measured_fraction_theta_full value Low . 0.328 Why?

Author Response: This measurement was performed at high pressure which, due to the high

PLAT901_ALERT_1_A Cell Parameters in CIF and FCF do not Match .... ! Error

---

Alert level B

PLAT149_ALERT_3_B s.u. on the beta Angle is Too Large .... 0.07 Degree

Author Response: Large errors due to low completeness.

```
PLAT340_ALERT_3_B Low Bond Precision on  C-C Bonds ..............    0.02138 Ang.
```

Author Response: Large errors due to low completeness.

```
PLAT369_ALERT_2_B Long   C(sp2)-C(sp2) Bond  C002     - C005     .       1.57 Ang.
```

Author Response: Large errors due to low completeness.

Alert level C

```
PLAT026_ALERT_3_C Ratio Observed / Unique Reflections (too) Low ..        46% Check
PLAT148_ALERT_3_C s.u. on the      a    - Axis is (Too) Large ....      0.011 Ang.
PLAT250_ALERT_2_C Large U3/U1 Ratio for <U(i,j)> Tensor(Resd    1)        2.9 Note
```

Alert level G

```
ABSMU01_ALERT_1_G  Calculation of _exptl_absorpt_correction_mu
                not performed for this radiation type.
PLAT003_ALERT_2_G Number of Uiso or U(i,j)  Restrained non-H-Atoms          6 Report
PLAT012_ALERT_1_G N.O.K.   _shelx_res_checksum Found in CIF ......     Please Check
PLAT013_ALERT_1_G N.O.K.   _shelx_hkl_checksum Found in CIF ......     Please Check
PLAT186_ALERT_4_G The CIF-Embedded .res File Contains ISOR Records          1 Report
PLAT199_ALERT_1_G Reported _cell_measurement_temperature ..... (K)        293 Check
PLAT200_ALERT_1_G Reported   _diffrn_ambient_temperature ..... (K)        293 Check
PLAT335_ALERT_2_G Check Large C6 Ring C-C Range C001     -C004_a         0.19 Ang.
PLAT335_ALERT_2_G Check Large C6 Ring C-C Range C002     -C005           0.33 Ang.
PLAT432_ALERT_2_G Short Inter X...Y Contact  C004     ..C007     .       3.16 Ang.
                                              x,3/2-y,1/2+z  =      4_576 Check
PLAT720_ALERT_4_G Number of Unusual/Non-Standard Labels ..........         12 Note
              C001    H001    C002    H002    C003    H003    C004    C005
              C006    H006    C007    H007
PLAT802_ALERT_4_G CIF Input Record(s) with more than 80 Characters          3 Info
PLAT860_ALERT_3_G Number of Least-Squares Restraints ............          36 Note
PLAT933_ALERT_2_G Number of HKL-OMIT Records in Embedded .res File         11 Note
               -5  6  4,  -3  1  9,  -5  5  4,  -3  0  8,  -3  3  7,  -2  2  2,
               -2  3  2,   1  4  3,  -1  2  1,  -3  2  4,   2  5  0,
PLAT941_ALERT_3_G Average HKL Measurement Multiplicity ...........        1.8 Low
PLAT950_ALERT_5_G Calculated (ThMax) and CIF-Reported Hmax Differ           4 Units
```

---

2 **ALERT level A** = Most likely a serious problem - resolve or explain
3 **ALERT level B** = A potentially serious problem, consider carefully
3 **ALERT level C** = Check. Ensure it is not caused by an omission or oversight
16 **ALERT level G** = General information/check it is not something unexpected

6 ALERT type 1 CIF construction/syntax error, inconsistent or missing data
7 ALERT type 2 Indicator that the structure model may be wrong or deficient
7 ALERT type 3 Indicator that the structure quality may be low
3 ALERT type 4 Improvement, methodology, query or suggestion
1 ALERT type 5 Informative message, check

Datablock: Anthracene_08.3GPa_IAM

```
Bond precision:   C-C = 0.0111 A          Wavelength=0.41000

Cell:              a=8.522(6)      b=5.5444(4)         c=7.4715(11)
                   alpha=90        beta=97.85(3)       gamma=90
Temperature:       293 K
```

	Calculated	Reported
Volume	349.7(3)	349.7(3)
Space group	P 21/c	P 1 21/c 1
Hall group	-P 2ybc	-P 2ybc
Moiety formula	C14 H10	C14 H10
Sum formula	C14 H10	C14 H10
Mr	178.22	178.22
Dx,g cm-3	1.693	1.692
Z	2	2
Mu (mm-1)	0.045	0.044
F000	188.0	188.0
F000'	187.97	
h,k,lmax	11,7,10	7,7,10
Nref	942	354
Tmin,Tmax	0.999,0.999	0.009,1.000
Tmin'	0.999	

Correction method= # Reported T Limits: Tmin=0.009 Tmax=1.000
AbsCorr = MULTI-SCAN

Data completeness= 0.376   Theta(max)= 16.306

R(reflections)= 0.0707(237)   wR2(reflections)= 0.2104(354)

S = 1.072   Npar= 64

The following ALERTS were generated. Each ALERT has the format
test-name_ALERT_alert-type_alert-level.
Click on the hyperlinks for more details of the test.

Alert level A

PLAT029_ALERT_3_A _diffrn_measured_fraction_theta_full value Low .   0.391 Why?

Author Response: This measurement was performed at high pressure which, due to the high

```
PLAT088_ALERT_3_A Poor Data / Parameter Ratio ...................       5.53 Note
```

Author Response: This measurement was performed at high pressure which, due to the high

---

Alert level B

```
PLAT340_ALERT_3_B Low Bond Precision on  C-C Bonds ..............    0.01113 Ang.
```

Author Response: Large errors due to low completeness.

```
PLAT911_ALERT_3_B Missing FCF Refl Between Thmin & STh/L=    0.600        322 Report
                2   0   0,   3   0   0,   4   0   0,   5   0   0,   6   0   0,   7   0   0,
                2   1   0,   3   1   0,   4   1   0,   5   1   0,   6   1   0,   7   1   0,
                3   2   0,   4   2   0,   5   2   0,   6   2   0,   7   2   0,   3   3   0,
                4   3   0,   5   3   0,   6   3   0,   7   3   0,   4   4   0,   5   4   0,
                6   4   0,   7   4   0,   5   5   0,   6   5   0,  -7   1   1,  -6   1   1,
                   (  292 More Missing: see the .ckf listing file)
```

Author Response: This measurement was performed at high pressure which, due to the high

---

Alert level C

```
PLAT230_ALERT_2_C Hirshfeld Test Diff for    C003     --C007     .         6.2 s.u.
PLAT241_ALERT_2_C High   'MainMol' Ueq as Compared to Neighbors of       C007 Check
PLAT242_ALERT_2_C Low    'MainMol' Ueq as Compared to Neighbors of       C003 Check
PLAT250_ALERT_2_C Large U3/U1 Ratio for <U(i,j)> Tensor(Resd    1)         2.2 Note
```

Alert level G

```
ABSMU01_ALERT_1_G  Calculation of _exptl_absorpt_correction_mu
                not performed for this radiation type.
PLAT003_ALERT_2_G Number of Uiso or U(i,j)  Restrained non-H-Atoms          1 Report
PLAT012_ALERT_1_G N.O.K.   _shelx_res_checksum Found in CIF ......     Please Check
PLAT072_ALERT_2_G SHELXL First  Parameter in WGHT  Unusually Large       0.15 Report
PLAT186_ALERT_4_G The CIF-Embedded .res File Contains ISOR Records          1 Report
PLAT199_ALERT_1_G Reported _cell_measurement_temperature ..... (K)         293 Check
PLAT200_ALERT_1_G Reported   _diffrn_ambient_temperature ..... (K)         293 Check
PLAT432_ALERT_2_G Short Inter X...Y Contact  C001     ..C001     .       3.15 Ang.
                                                 1-x,-y,1-z  =      3_656 Check
PLAT432_ALERT_2_G Short Inter X...Y Contact  C001     ..C003     .       3.16 Ang.
                                           1-x,-1/2+y,1/2-z  =      2_645 Check
PLAT432_ALERT_2_G Short Inter X...Y Contact  C003     ..C005     .       3.03 Ang.
                                             x,3/2-y,-1/2+z  =      4_575 Check
PLAT432_ALERT_2_G Short Inter X...Y Contact  C003     ..C004     .       3.07 Ang.
                                             x,3/2-y,-1/2+z  =      4_575 Check
PLAT432_ALERT_2_G Short Inter X...Y Contact  C006     ..C007     .       3.13 Ang.
                                            -x,-1/2+y,1/2-z  =      2_545 Check
PLAT720_ALERT_4_G Number of Unusual/Non-Standard Labels .........          12 Note
              C001     H001     C002     H002     C003     H003     C004     C005
              C006     H006     C007     H007
```

```
PLAT802_ALERT_4_G CIF Input Record(s) with more than 80 Characters          3 Info
PLAT860_ALERT_3_G Number of Least-Squares Restraints .............          6 Note
PLAT883_ALERT_1_G Absent Datum for _atom_sites_solution_primary ..     Please Do !
PLAT910_ALERT_3_G Missing FCF Reflection(s) Below Theta(Min)[Deg]=       2.65 Note
                1  0  0,   1  1  0,
PLAT912_ALERT_4_G Missing # of FCF Reflections Above STh/L=  0.600        105 Note
PLAT913_ALERT_3_G Missing # of Very Strong Reflections in FCF ....          3 Note
               -1  1  1,  -2  0  2,  -2  1  2,
PLAT933_ALERT_2_G Number of HKL-OMIT Records in Embedded .res File          1 Note
               -6  6  4,
PLAT941_ALERT_3_G Average HKL Measurement Multiplicity ..........        1.5 Low
PLAT950_ALERT_5_G Calculated (ThMax) and CIF-Reported Hmax Differ           4 Units
PLAT956_ALERT_1_G Calculated (ThMax) and Actual (FCF) Hmax Differ           4 Units
PLAT969_ALERT_5_G The 'Henn et al.' R-Factor-gap value ..........      6.969 Note
              Predicted wR2: Based on SigI**2  3.02 or SHELX Weight 19.62
PLAT978_ALERT_2_G Number C-C Bonds with Positive Residual Density.          0 Info
```

```
   2 ALERT level A = Most likely a serious problem - resolve or explain
   2 ALERT level B = A potentially serious problem, consider carefully
   4 ALERT level C = Check. Ensure it is not caused by an omission or oversight
  25 ALERT level G = General information/check it is not something unexpected

   6 ALERT type 1 CIF construction/syntax error, inconsistent or missing data
  13 ALERT type 2 Indicator that the structure model may be wrong or deficient
   8 ALERT type 3 Indicator that the structure quality may be low
   4 ALERT type 4 Improvement, methodology, query or suggestion
   2 ALERT type 5 Informative message, check
```

Datablock: Anthracene_10.8GPa_IAM

Bond precision: C-C = 0.0064 A Wavelength=0.41000

Cell:	a=8.422(5)	b=5.4845(4)	c=7.3579(8)
	alpha=90	beta=97.26(2)	gamma=90
Temperature:	293 K		

	Calculated	Reported
Volume	337.1(2)	337.12(19)
Space group	P 21/c	P 1 21/c 1
Hall group	-P 2ybc	-P 2ybc
Moiety formula	C14 H10	C14 H10
Sum formula	C14 H10	C14 H10
Mr	178.22	178.22
Dx,g cm-3	1.756	1.756
Z	2	2
Mu (mm-1)	0.046	0.046
F000	188.0	188.0
F000'	187.97	
h,k,lmax	13,8,11	7,8,11
Nref	1399	402
Tmin,Tmax	0.999,0.999	0.327,1.000
Tmin'	0.999	

Correction method= # Reported T Limits: Tmin=0.327 Tmax=1.000
AbsCorr = MULTI-SCAN

Data completeness= 0.287 Theta(max)= 18.942

R(reflections)= 0.0454(246) wR2(reflections)= 0.1123(402)

S = 0.930 Npar= 64

The following ALERTS were generated. Each ALERT has the format
test-name_ALERT_alert-type_alert-level.
Click on the hyperlinks for more details of the test.

Alert level A

```
PLAT029_ALERT_3_A _diffrn_measured_fraction_theta_full value Low .      0.376 Why?
```

Author Response: This measurement was performed at high pressure which, due to the high

```
PLAT901_ALERT_1_A Cell Parameters in CIF and FCF do not Match ....          ! Error
```

Alert level C

```
PLAT250_ALERT_2_C Large U3/U1 Ratio for <U(i,j)> Tensor(Resd    1)         2.9 Note
PLAT340_ALERT_3_C Low Bond Precision on  C-C Bonds ...............     0.00638 Ang.
PLAT411_ALERT_2_C Short Inter H...H Contact  H003     ..H003      .       2.09 Ang.
                                                    1-x,-y,1-z  =      3_656 Check
PLAT411_ALERT_2_C Short Inter H...H Contact  H004     ..H007      .       2.09 Ang.
                                               -x,-1/2+y,1/2-z  =      2_545 Check
```

Alert level G

```
ABSMU01_ALERT_1_G  Calculation of _exptl_absorpt_correction_mu
                not performed for this radiation type.
PLAT012_ALERT_1_G N.O.K.   _shelx_res_checksum Found in CIF ......     Please Check
PLAT199_ALERT_1_G Reported _cell_measurement_temperature ..... (K)        293 Check
PLAT200_ALERT_1_G Reported   _diffrn_ambient_temperature ..... (K)        293 Check
PLAT432_ALERT_2_G Short Inter X...Y Contact  C001     ..C005     .       3.02 Ang.
                                              x,3/2-y,1/2+z  =      4_576 Check
PLAT432_ALERT_2_G Short Inter X...Y Contact  C001     ..C003     .       3.14 Ang.
                                             x,1/2-y,-1/2+z  =      4_565 Check
PLAT432_ALERT_2_G Short Inter X...Y Contact  C002     ..C005     .       2.99 Ang.
                                              x,3/2-y,1/2+z  =      4_576 Check
PLAT432_ALERT_2_G Short Inter X...Y Contact  C002     ..C006     .       3.17 Ang.
                                              x,3/2-y,1/2+z  =      4_576 Check
PLAT432_ALERT_2_G Short Inter X...Y Contact  C003     ..C003     .       3.09 Ang.
                                                 1-x,-y,1-z  =      3_656 Check
PLAT432_ALERT_2_G Short Inter X...Y Contact  C003     ..C005     .       3.12 Ang.
                                          1-x,-1/2+y,1/2-z  =      2_645 Check
PLAT432_ALERT_2_G Short Inter X...Y Contact  C003     ..C005     .       3.20 Ang.
                                              x,3/2-y,1/2+z  =      4_576 Check
PLAT432_ALERT_2_G Short Inter X...Y Contact  C004     ..C005     .       3.16 Ang.
                                                   x,-1+y,z  =      1_545 Check
PLAT432_ALERT_2_G Short Inter X...Y Contact  C006     ..C007     .       3.08 Ang.
                                             -x,1/2+y,1/2-z  =      2_555 Check
PLAT720_ALERT_4_G Number of Unusual/Non-Standard Labels ..........         12 Note
              C001    C002    C003    H003    C004    H004    C005    H005
              C006    H006    C007    H007
PLAT802_ALERT_4_G CIF Input Record(s) with more than 80 Characters          3 Info
PLAT883_ALERT_1_G Absent Datum for _atom_sites_solution_primary ..     Please Do !
PLAT933_ALERT_2_G Number of HKL-OMIT Records in Embedded .res File         30 Note
               -7  5  5,  -6  3  8,  -6  6  4,  -5  2  9,  -5  2 10,  -5  3  7,
               -5  4  7,  -4  2  8,  -4  2  9,  -4  4  6,  -4  5  2,  -4  5  8,
               -4  6  2,  -3  2  8,  -3  7  2,  -3  7  5,  -2  3  1,  -2  3  4,
               -2  5  5,  -2  6  5,  -2  7  4,  -2  7  5,  -1  3  3,  -1  6  5,
               -1  7  3,   0  2  7,   0  6  4,   1  1  0,   2  2  1,   3  7  3,
PLAT941_ALERT_3_G Average HKL Measurement Multiplicity ..........        1.4 Low
PLAT950_ALERT_5_G Calculated (ThMax) and CIF-Reported Hmax Differ           6 Units
```

---

2 **ALERT level A** = Most likely a serious problem - resolve or explain
0 **ALERT level B** = A potentially serious problem, consider carefully
4 **ALERT level C** = Check. Ensure it is not caused by an omission or oversight
19 **ALERT level G** = General information/check it is not something unexpected

6 ALERT type 1 CIF construction/syntax error, inconsistent or missing data
13 ALERT type 2 Indicator that the structure model may be wrong or deficient
3 ALERT type 3 Indicator that the structure quality may be low
2 ALERT type 4 Improvement, methodology, query or suggestion
1 ALERT type 5 Informative message, check

Datablock: Anthracene_13.5GPa_IAM

Bond precision: C-C = 0.0086 A   Wavelength=0.41000

Cell:	a=8.291(3)	b=5.4007(3)	c=7.2067(6)
	alpha=90	beta=96.21(2)	gamma=90
Temperature:	293 K		

	Calculated	Reported
Volume	320.80(12)	320.81(14)
Space group	P 21/c	P 1 21/c 1
Hall group	-P 2ybc	-P 2ybc
Moiety formula	C14 H10	C14 H10
Sum formula	C14 H10	C14 H10
Mr	178.22	178.22
Dx,g cm-3	1.845	1.845
Z	2	2
Mu (mm-1)	0.049	0.048
F000	188.0	188.0
F000’	187.97	
h,k,lmax	14,9,12	7,9,11
Nref	1809	459
Tmin,Tmax	0.999,0.999	0.358,1.000
Tmin’	0.999	

Correction method= # Reported T Limits: Tmin=0.358 Tmax=1.000
AbsCorr = MULTI-SCAN

Data completeness= 0.254 Theta(max)= 21.019

R(reflections)= 0.0588(248) wR2(reflections)= 0.1955(459)

S = 1.094 Npar= 64

The following ALERTS were generated. Each ALERT has the format
test-name_ALERT_alert-type_alert-level.
Click on the hyperlinks for more details of the test.

Alert level A

PLAT029_ALERT_3_A _diffrn_measured_fraction_theta_full value Low . 0.412 Why?

Author Response: This measurement was performed at high pressure which, due to the high

---

Alert level B

PLAT088_ALERT_3_B Poor Data / Parameter Ratio .... 7.17 Note

Author Response: This measurement was performed at high pressure which, due to the high

```
PLAT911_ALERT_3_B Missing FCF Refl Between Thmin & STh/L=    0.600        294 Report
                2  0  0,   3  0  0,   4  0  0,   5  0  0,   6  0  0,   7  0  0,
                2  1  0,   3  1  0,   4  1  0,   5  1  0,   6  1  0,   7  1  0,
                3  2  0,   4  2  0,   5  2  0,   6  2  0,   7  2  0,   2  3  0,
                3  3  0,   4  3  0,   5  3  0,   6  3  0,   7  3  0,   5  4  0,
                6  4  0,   7  4  0,   5  5  0,   6  5  0,  -7  1  1,  -6  1  1,
                   (  264 More Missing: see the .ckf listing file)
```

Author Response: This measurement was performed at high pressure which, due to the high

---

Alert level C

```
PLAT230_ALERT_2_C Hirshfeld Test Diff for    C001     --C004     .         5.1 s.u.
PLAT250_ALERT_2_C Large U3/U1 Ratio for <U(i,j)> Tensor(Resd    1)         3.0 Note
PLAT340_ALERT_3_C Low Bond Precision on  C-C Bonds ...............     0.00862 Ang.
PLAT411_ALERT_2_C Short Inter H...H Contact  H003     ..H003     .        2.05 Ang.
                                             1-x,-y,1-z  =      3_656 Check
PLAT411_ALERT_2_C Short Inter H...H Contact  H004     ..H007     .        2.04 Ang.
                                        -x,-1/2+y,1/2-z  =      2_545 Check
PLAT411_ALERT_2_C Short Inter H...H Contact  H006     ..H007     .        2.11 Ang.
                                              -x,1-y,-z  =      3_565 Check
PLAT906_ALERT_3_C Large K Value in the Analysis of Variance ......       4.515 Check
```

Alert level G

```
ABSMU01_ALERT_1_G  Calculation of _exptl_absorpt_correction_mu
                not performed for this radiation type.
PLAT012_ALERT_1_G N.O.K.   _shelx_res_checksum Found in CIF ......     Please Check
PLAT072_ALERT_2_G SHELXL First  Parameter in WGHT  Unusually Large        0.10 Report
PLAT152_ALERT_1_G The Supplied and Calc. Volume s.u. Differ by ...          -2 Units
PLAT199_ALERT_1_G Reported _cell_measurement_temperature ..... (K)         293 Check
PLAT200_ALERT_1_G Reported   _diffrn_ambient_temperature ..... (K)         293 Check
PLAT432_ALERT_2_G Short Inter X...Y Contact  C001     ..C005     .        2.94 Ang.
                                          x,3/2-y,1/2+z  =      4_576 Check
PLAT432_ALERT_2_G Short Inter X...Y Contact  C001     ..C003     .        3.06 Ang.
                                         x,1/2-y,-1/2+z  =      4_565 Check
PLAT432_ALERT_2_G Short Inter X...Y Contact  C001     ..C006     .        3.17 Ang.
                                          x,3/2-y,1/2+z  =      4_576 Check
PLAT432_ALERT_2_G Short Inter X...Y Contact  C002     ..C005     .        2.90 Ang.
                                          x,3/2-y,1/2+z  =      4_576 Check
PLAT432_ALERT_2_G Short Inter X...Y Contact  C002     ..C006     .        3.05 Ang.
                                          x,3/2-y,1/2+z  =      4_576 Check
PLAT432_ALERT_2_G Short Inter X...Y Contact  C003     ..C003     .        3.01 Ang.
                                             1-x,-y,1-z  =      3_656 Check
PLAT432_ALERT_2_G Short Inter X...Y Contact  C003     ..C005     .        3.05 Ang.
                                       1-x,-1/2+y,1/2-z  =      2_645 Check
PLAT432_ALERT_2_G Short Inter X...Y Contact  C003     ..C005     .        3.12 Ang.
                                          x,3/2-y,1/2+z  =      4_576 Check
PLAT432_ALERT_2_G Short Inter X...Y Contact  C003     ..C004     .        3.13 Ang.
                                          x,1/2-y,1/2+z  =      4_566 Check
PLAT432_ALERT_2_G Short Inter X...Y Contact  C004     ..C005     .        3.08 Ang.
```

```
                                             x,-1+y,z  =      1_545 Check
PLAT432_ALERT_2_G Short Inter X...Y Contact  C004      ..C006     .       3.17 Ang.
                                      -x,-1/2+y,1/2-z  =      2_545 Check
PLAT432_ALERT_2_G Short Inter X...Y Contact  C006      ..C007     .       3.04 Ang.
                                       -x,1/2+y,1/2-z  =      2_555 Check
PLAT720_ALERT_4_G Number of Unusual/Non-Standard Labels ..........          12 Note
              C001    C002    C003    H003    C004    H004    C005    H005
              C006    H006    C007    H007
PLAT802_ALERT_4_G CIF Input Record(s) with more than 80 Characters           3 Info
PLAT883_ALERT_1_G Absent Datum for _atom_sites_solution_primary ..     Please Do !
PLAT910_ALERT_3_G Missing FCF Reflection(s) Below Theta(Min)[Deg]=       2.72 Note
                1  0  0,   1  1  0,
PLAT912_ALERT_4_G Missing # of FCF Reflections Above STh/L=  0.600         499 Note
PLAT913_ALERT_3_G Missing # of Very Strong Reflections in FCF ....           3 Note
               -1  1  1,  -2  0  2,  -2  1  2,
PLAT933_ALERT_2_G Number of HKL-OMIT Records in Embedded .res File           6 Note
               -6  6  4,  -5  7  3,  -4  1 10,  -4  3  6,  -3  3  4,  -3  4  5,
PLAT941_ALERT_3_G Average HKL Measurement Multiplicity ..........          1.5 Low
PLAT950_ALERT_5_G Calculated (ThMax) and CIF-Reported Hmax Differ            7 Units
PLAT956_ALERT_1_G Calculated (ThMax) and Actual (FCF) Hmax Differ            7 Units
PLAT969_ALERT_5_G The 'Henn et al.' R-Factor-gap value ..........     16.771 Note
              Predicted wR2: Based on SigI**2  1.17 or SHELX Weight 17.87
PLAT978_ALERT_2_G Number C-C Bonds with Positive Residual Density.           0 Info
```

---

   1 **ALERT level A** = Most likely a serious problem - resolve or explain
   2 **ALERT level B** = A potentially serious problem, consider carefully
   7 **ALERT level C** = Check. Ensure it is not caused by an omission or oversight
  30 **ALERT level G** = General information/check it is not something unexpected

   7 ALERT type 1 CIF construction/syntax error, inconsistent or missing data
  20 ALERT type 2 Indicator that the structure model may be wrong or deficient
   8 ALERT type 3 Indicator that the structure quality may be low
   3 ALERT type 4 Improvement, methodology, query or suggestion
   2 ALERT type 5 Informative message, check

Datablock: Anthracene_15.8GPa_IAM

Bond precision: C-C = 0.0080 A   Wavelength=0.41000

Cell:	a=8.209(5)	b=5.3592(4)	c=7.1344(9)
	alpha=90	beta=95.72(3)	gamma=90
Temperature:	293 K		

	Calculated	Reported
Volume	312.3(2)	312.31(18)
Space group	P 21/c	P 1 21/c 1
Hall group	-P 2ybc	-P 2ybc
Moiety formula	C14 H10	C14 H10
Sum formula	C14 H10	C14 H10
Mr	178.22	178.22
Dx,g cm-3	1.895	1.895
Z	2	2
Mu (mm-1)	0.050	0.049
F000	188.0	188.0
F000'	187.97	
h,k,lmax	11,7,10	7,7,10
Nref	952	348
Tmin,Tmax	0.999,0.999	0.603,1.000
Tmin'	0.999	

Correction method= # Reported T Limits: Tmin=0.603 Tmax=1.000
AbsCorr = MULTI-SCAN

Data completeness= 0.366 Theta(max)= 17.012

R(reflections)= 0.0445(225) wR2(reflections)= 0.1227(348)

S = 1.066 Npar= 64

The following ALERTS were generated. Each ALERT has the format
test-name_ALERT_alert-type_alert-level.
Click on the hyperlinks for more details of the test.

Alert level A

PLAT029_ALERT_3_A _diffrn_measured_fraction_theta_full value Low . 0.393 Why?

Author Response: This measurement was performed at high pressure which, due to the high

PLAT088_ALERT_3_A Poor Data / Parameter Ratio .... 5.44 Note

Author Response: This measurement was performed at high pressure which, due to the high

---

Alert level B

PLAT411_ALERT_2_B Short Inter H...H Contact H004 ..H007 . 1.99 Ang.
-x,-1/2+y,1/2-z = 2_545 Check

Author Response: H atoms are close to each other at high pressure.

```
PLAT911_ALERT_3_B Missing FCF Refl Between Thmin & STh/L=    0.600        299 Report
                2  0  0,   3  0  0,   4  0  0,   5  0  0,   6  0  0,   7  0  0,
                2  1  0,   3  1  0,   4  1  0,   5  1  0,   6  1  0,   7  1  0,
                2  2  0,   3  2  0,   4  2  0,   5  2  0,   6  2  0,   7  2  0,
                2  3  0,   3  3  0,   4  3  0,   5  3  0,   6  3  0,   7  3  0,
                5  4  0,   6  4  0,   7  4  0,   5  5  0,   6  5  0,  -7  1  1,
                   (  269 More Missing: see the .ckf listing file)
```

Author Response: This measurement was performed at high pressure which, due to the high

---

Alert level C

```
CRYSC01_ALERT_1_C The word below has not been recognised as a standard
            identifier.
            yellowish
PLAT234_ALERT_4_C Large Hirshfeld Difference C001     --C004     .       0.16 Ang.
PLAT250_ALERT_2_C Large U3/U1 Ratio for <U(i,j)> Tensor(Resd    1)        3.4 Note
PLAT340_ALERT_3_C Low Bond Precision on  C-C Bonds ..............      0.008 Ang.
PLAT411_ALERT_2_C Short Inter H...H Contact  H003     ..H003     .       2.03 Ang.
                                                 1-x,-y,1-z  =      3_656 Check
```

Author Response: H atoms are close to each other at high pressure.

```
PLAT411_ALERT_2_C Short Inter H...H Contact  H006     ..H007     .       2.06 Ang.
                                                  -x,1-y,-z  =      3_565 Check
```

Author Response: H atoms are close to each other at high pressure.

```
PLAT906_ALERT_3_C Large K Value in the Analysis of Variance ......      6.412 Check
```

Alert level G

```
ABSMU01_ALERT_1_G  Calculation of _exptl_absorpt_correction_mu
                not performed for this radiation type.
PLAT012_ALERT_1_G N.O.K.   _shelx_res_checksum Found in CIF ......     Please Check
PLAT152_ALERT_1_G The Supplied and Calc. Volume s.u. Differ by ...          2 Units
PLAT199_ALERT_1_G Reported _cell_measurement_temperature ..... (K)        293 Check
PLAT200_ALERT_1_G Reported   _diffrn_ambient_temperature ..... (K)        293 Check
PLAT432_ALERT_2_G Short Inter X...Y Contact  C001     ..C005     .       2.90 Ang.
                                              x,3/2-y,1/2+z  =      4_576 Check
PLAT432_ALERT_2_G Short Inter X...Y Contact  C001     ..C003     .       3.02 Ang.
                                             x,1/2-y,-1/2+z  =      4_565 Check
PLAT432_ALERT_2_G Short Inter X...Y Contact  C001     ..C006     .       3.13 Ang.
                                              x,3/2-y,1/2+z  =      4_576 Check
PLAT432_ALERT_2_G Short Inter X...Y Contact  C002     ..C005     .       2.87 Ang.
                                              x,3/2-y,1/2+z  =      4_576 Check
PLAT432_ALERT_2_G Short Inter X...Y Contact  C002     ..C006     .       3.01 Ang.
                                              x,3/2-y,1/2+z  =      4_576 Check
```

```
PLAT432_ALERT_2_G Short Inter X...Y Contact  C003     ..C003     .       2.98 Ang.
                                           1-x,-y,1-z  =      3_656 Check
PLAT432_ALERT_2_G Short Inter X...Y Contact  C003     ..C005     .       3.02 Ang.
                                     1-x,-1/2+y,1/2-z  =      2_645 Check
PLAT432_ALERT_2_G Short Inter X...Y Contact  C003     ..C005     .       3.09 Ang.
                                        x,3/2-y,1/2+z  =      4_576 Check
PLAT432_ALERT_2_G Short Inter X...Y Contact  C003     ..C004     .       3.09 Ang.
                                        x,1/2-y,1/2+z  =      4_566 Check
PLAT432_ALERT_2_G Short Inter X...Y Contact  C004     ..C005     .       3.04 Ang.
                                             x,-1+y,z  =      1_545 Check
PLAT432_ALERT_2_G Short Inter X...Y Contact  C004     ..C006     .       3.09 Ang.
                                      -x,-1/2+y,1/2-z  =      2_545 Check
PLAT432_ALERT_2_G Short Inter X...Y Contact  C004     ..C007     .       3.17 Ang.
                                      -x,-1/2+y,1/2-z  =      2_545 Check
PLAT432_ALERT_2_G Short Inter X...Y Contact  C006     ..C007     .       2.96 Ang.
                                       -x,1/2+y,1/2-z  =      2_555 Check
PLAT720_ALERT_4_G Number of Unusual/Non-Standard Labels .........         12 Note
              C001    C002    C003    H003    C004    H004    C005    H005
              C006    H006    C007    H007
PLAT802_ALERT_4_G CIF Input Record(s) with more than 80 Characters          3 Info
PLAT883_ALERT_1_G Absent Datum for _atom_sites_solution_primary ..     Please Do !
PLAT910_ALERT_3_G Missing FCF Reflection(s) Below Theta(Min)[Deg]=       2.75 Note
                1  0  0,   1  1  0,
PLAT912_ALERT_4_G Missing # of FCF Reflections Above STh/L=  0.600        143 Note
PLAT913_ALERT_3_G Missing # of Very Strong Reflections in FCF ....          3 Note
               -1  1  1,  -2  0  2,  -2  1  2,
PLAT933_ALERT_2_G Number of HKL-OMIT Records in Embedded .res File          3 Note
               -6  6  4,  -4  7  2,   0  6  1,
PLAT941_ALERT_3_G Average HKL Measurement Multiplicity ..........         1.5 Low
PLAT950_ALERT_5_G Calculated (ThMax) and CIF-Reported Hmax Differ           4 Units
PLAT956_ALERT_1_G Calculated (ThMax) and Actual (FCF) Hmax Differ           4 Units
PLAT969_ALERT_5_G The 'Henn et al.' R-Factor-gap value ..........       7.823 Note
              Predicted wR2: Based on SigI**2  1.57 or SHELX Weight 11.52
PLAT978_ALERT_2_G Number C-C Bonds with Positive Residual Density.          1 Info
```

```
   2 ALERT level A = Most likely a serious problem - resolve or explain
   2 ALERT level B = A potentially serious problem, consider carefully
   7 ALERT level C = Check. Ensure it is not caused by an omission or oversight
  30 ALERT level G = General information/check it is not something unexpected

   8 ALERT type 1 CIF construction/syntax error, inconsistent or missing data
  19 ALERT type 2 Indicator that the structure model may be wrong or deficient
   8 ALERT type 3 Indicator that the structure quality may be low
   4 ALERT type 4 Improvement, methodology, query or suggestion
   2 ALERT type 5 Informative message, check
```

Datablock: Anthracene_18.4GPa_IAM

Bond precision: C-C = 0.0084 A   Wavelength=0.41000

Cell:	a=8.156(6)	b=5.3045(3)	c=7.0586(3)
	alpha=90	beta=95.183(13)	gamma=90
Temperature:	293 K		

	Calculated	Reported
Volume	304.1(2)	304.1(2)
Space group	P 21/c	P 1 21/c 1
Hall group	-P 2ybc	-P 2ybc
Moiety formula	C14 H10	C14 H10
Sum formula	C14 H10	C14 H10
Mr	178.22	178.22
Dx,g cm-3	1.946	1.946
Z	2	2
Mu (mm-1)	0.051	0.051
F000	188.0	188.0
F000'	187.97	
h,k,lmax	11,7,10	3,7,10
Nref	921	291
Tmin,Tmax	0.999,0.999	0.370,1.000
Tmin'	0.999	

Correction method= # Reported T Limits: Tmin=0.370 Tmax=1.000
AbsCorr = MULTI-SCAN

Data completeness= 0.316 Theta(max)= 16.985

R(reflections)= 0.0620(234) wR2(reflections)= 0.1887(291)

S = 1.096 Npar= 64

The following ALERTS were generated. Each ALERT has the format
test-name_ALERT_alert-type_alert-level.
Click on the hyperlinks for more details of the test.

Alert level A

PLAT029_ALERT_3_A _diffrn_measured_fraction_theta_full value Low . 0.330 Why?

Author Response: This measurement was performed at high pressure which, due to the high

PLAT088_ALERT_3_A Poor Data / Parameter Ratio .... 4.55 Note

Author Response: This measurement was performed at high pressure which, due to the high

Alert level B

```
PLAT250_ALERT_2_B Large U3/U1 Ratio for <U(i,j)> Tensor(Resd    1)        5.3 Note
```

Author Response: Large errors due to low completeness.

```
PLAT411_ALERT_2_B Short Inter H...H Contact  H003     ..H003     .       1.97 Ang.
                                                1-x,-y,1-z  =      3_656 Check
```

Author Response: H atoms are close to each other at high pressure.

```
PLAT411_ALERT_2_B Short Inter H...H Contact  H004     ..H007     .       1.92 Ang.
                                           -x,-1/2+y,1/2-z  =      2_545 Check
```

Author Response: H atoms are close to each other at high pressure.

```
PLAT911_ALERT_3_B Missing FCF Refl Between Thmin & STh/L=    0.600        102 Report
                2  0  0,   3  0  0,   2  1  0,   3  1  0,   1  2  0,   2  2  0,
                3  2  0,   2  3  0,   3  3  0,   3  4  0,   3  5  0,   0  6  0,
                1  6  0,   3  6  0,  -3  1  1,  -2  1  1,  -1  1  1,   1  1  1,
                2  1  1,   3  1  1,  -3  2  1,  -2  2  1,   1  2  1,   2  2  1,
                3  2  1,  -3  3  1,  -2  3  1,   2  3  1,   3  3  1,  -3  4  1,
                   (   72 More Missing: see the .ckf listing file)
```

Author Response: This measurement was performed at high pressure which, due to the high

Alert level C

```
PLAT234_ALERT_4_C Large Hirshfeld Difference C001     --C004     .       0.16 Ang.
PLAT242_ALERT_2_C Low    'MainMol' Ueq as Compared to Neighbors of       C004 Check
PLAT340_ALERT_3_C Low Bond Precision on  C-C Bonds ...............    0.00838 Ang.
PLAT411_ALERT_2_C Short Inter H...H Contact  H004     ..H005     .       2.12 Ang.
                                                  x,-1+y,z  =      1_545 Check
```

Author Response: H atoms are close to each other at high pressure.

```
PLAT411_ALERT_2_C Short Inter H...H Contact  H006     ..H007     .       2.01 Ang.
                                                 -x,1-y,-z  =      3_565 Check
```

Author Response: H atoms are close to each other at high pressure.

```
PLAT411_ALERT_2_C Short Inter H...H Contact  H007     ..H007     .       2.12 Ang.
                                                 -x,1-y,-z  =      3_565 Check
```

Author Response: H atoms are close to each other at high pressure.

```
PLAT906_ALERT_3_C Large K Value in the Analysis of Variance ......      2.958 Check
```

Alert level G

```
ABSMU01_ALERT_1_G  Calculation of _exptl_absorpt_correction_mu
                not performed for this radiation type.
PLAT012_ALERT_1_G N.O.K.   _shelx_res_checksum Found in CIF ......     Please Check
PLAT072_ALERT_2_G SHELXL First  Parameter in WGHT  Unusually Large       0.15 Report
PLAT199_ALERT_1_G Reported _cell_measurement_temperature ..... (K)        293 Check
PLAT200_ALERT_1_G Reported   _diffrn_ambient_temperature ..... (K)        293 Check
PLAT432_ALERT_2_G Short Inter X...Y Contact  C001     ..C005     .       2.86 Ang.
                                              x,3/2-y,1/2+z  =      4_576 Check
PLAT432_ALERT_2_G Short Inter X...Y Contact  C001     ..C003     .       2.98 Ang.
                                             x,1/2-y,-1/2+z  =      4_565 Check
PLAT432_ALERT_2_G Short Inter X...Y Contact  C001     ..C006     .       3.07 Ang.
                                              x,3/2-y,1/2+z  =      4_576 Check
PLAT432_ALERT_2_G Short Inter X...Y Contact  C001     ..C005     .       3.20 Ang.
                                           1-x,-1/2+y,1/2-z  =      2_645 Check
PLAT432_ALERT_2_G Short Inter X...Y Contact  C002     ..C005     .       2.84 Ang.
                                              x,3/2-y,1/2+z  =      4_576 Check
PLAT432_ALERT_2_G Short Inter X...Y Contact  C002     ..C006     .       2.96 Ang.
                                              x,3/2-y,1/2+z  =      4_576 Check
PLAT432_ALERT_2_G Short Inter X...Y Contact  C002     ..C005     .       3.18 Ang.
                                           1-x,-1/2+y,1/2-z  =      2_645 Check
PLAT432_ALERT_2_G Short Inter X...Y Contact  C003     ..C003     .       2.92 Ang.
                                                 1-x,-y,1-z  =      3_656 Check
PLAT432_ALERT_2_G Short Inter X...Y Contact  C003     ..C005     .       2.99 Ang.
                                           1-x,-1/2+y,1/2-z  =      2_645 Check
PLAT432_ALERT_2_G Short Inter X...Y Contact  C003     ..C005     .       3.05 Ang.
                                              x,3/2-y,1/2+z  =      4_576 Check
PLAT432_ALERT_2_G Short Inter X...Y Contact  C003     ..C004     .       3.07 Ang.
                                              x,1/2-y,1/2+z  =      4_566 Check
PLAT432_ALERT_2_G Short Inter X...Y Contact  C004     ..C005     .       3.00 Ang.
                                                   x,-1+y,z  =      1_545 Check
PLAT432_ALERT_2_G Short Inter X...Y Contact  C004     ..C006     .       3.06 Ang.
                                            -x,-1/2+y,1/2-z  =      2_545 Check
PLAT432_ALERT_2_G Short Inter X...Y Contact  C004     ..C007     .       3.11 Ang.
                                            -x,-1/2+y,1/2-z  =      2_545 Check
PLAT432_ALERT_2_G Short Inter X...Y Contact  C004     ..C007     .       3.18 Ang.
                                              x,1/2-y,1/2+z  =      4_566 Check
PLAT432_ALERT_2_G Short Inter X...Y Contact  C005     ..C006     .       3.16 Ang.
                                              x,3/2-y,1/2+z  =      4_576 Check
PLAT432_ALERT_2_G Short Inter X...Y Contact  C006     ..C007     .       2.92 Ang.
                                              -x,1/2+y,1/2-z  =      2_555 Check
PLAT432_ALERT_2_G Short Inter X...Y Contact  C007     ..C007     .       3.16 Ang.
                                                 -x,1-y,-z  =      3_565 Check
PLAT720_ALERT_4_G Number of Unusual/Non-Standard Labels ..........         12 Note
              C001    C002    C003    H003    C004    H004    C005    H005
              C006    H006    C007    H007
PLAT802_ALERT_4_G CIF Input Record(s) with more than 80 Characters          3 Info
PLAT883_ALERT_1_G Absent Datum for _atom_sites_solution_primary ..     Please Do !
PLAT910_ALERT_3_G Missing FCF Reflection(s) Below Theta(Min)[Deg]=       2.78 Note
                1  0  0,   1  1  0,
PLAT912_ALERT_4_G Missing # of FCF Reflections Above STh/L=  0.600         10 Note
PLAT913_ALERT_3_G Missing # of Very Strong Reflections in FCF ....          3 Note
               -1  1  1,  -2  0  2,  -2  1  2,
PLAT933_ALERT_2_G Number of HKL-OMIT Records in Embedded .res File          4 Note
```

```
              -2  3  3,  -1  0  2,  -1  1  1,   3  3  7,
PLAT941_ALERT_3_G Average HKL Measurement Multiplicity ..........        1.7 Low
PLAT950_ALERT_5_G Calculated (ThMax) and CIF-Reported Hmax Differ          8 Units
PLAT956_ALERT_1_G Calculated (ThMax) and Actual (FCF) Hmax Differ          8 Units
PLAT969_ALERT_5_G The 'Henn et al.' R-Factor-gap value ..........     11.953 Note
             Predicted wR2: Based on SigI**2  1.58 or SHELX Weight 17.22
PLAT978_ALERT_2_G Number C-C Bonds with Positive Residual Density.         1 Info
```

---

2 **ALERT level A** = Most likely a serious problem - resolve or explain
4 **ALERT level B** = A potentially serious problem, consider carefully
7 **ALERT level C** = Check. Ensure it is not caused by an omission or oversight
35 **ALERT level G** = General information/check it is not something unexpected

6 ALERT type 1 CIF construction/syntax error, inconsistent or missing data
28 ALERT type 2 Indicator that the structure model may be wrong or deficient
8 ALERT type 3 Indicator that the structure quality may be low
4 ALERT type 4 Improvement, methodology, query or suggestion
2 ALERT type 5 Informative message, check

Datablock: Anthracene_21.5GPa_IAM

Bond precision: C-C = 0.0088 A   Wavelength=0.41000

Cell:	a=8.075(5)	b=5.2590(4)	c=6.9637(9)
	alpha=90	beta=94.70(3)	gamma=90
Temperature:	293 K		

	Calculated	Reported
Volume	294.73(19)	294.7(2)
Space group	P 21/c	P 1 21/c 1
Hall group	-P 2ybc	-P 2ybc
Moiety formula	C14 H10	C14 H10
Sum formula	C14 H10	C14 H10
Mr	178.22	178.22
Dx,g cm-3	2.008	2.008
Z	2	2
Mu (mm-1)	0.053	0.052
F000	188.0	188.0
F000'	187.97	
h,k,lmax	14,9,12	7,9,11
Nref	1647	412
Tmin,Tmax	0.999,0.999	0.040,1.000
Tmin'	0.999	

Correction method= # Reported T Limits: Tmin=0.040 Tmax=1.000
AbsCorr = MULTI-SCAN

```
Data completeness= 0.250            Theta(max)= 21.022

R(reflections)= 0.0714( 251)                        wR2(reflections)=
                                                    0.2184( 412)
S = 1.029                    Npar= 64
```

The following ALERTS were generated. Each ALERT has the format
test-name_ALERT_alert-type_alert-level.
Click on the hyperlinks for more details of the test.

Alert level A

```
PLAT029_ALERT_3_A _diffrn_measured_fraction_theta_full value Low .      0.400 Why?
```

Author Response: This measurement was performed at high pressure which, due to the high

---

Alert level B

```
PLAT088_ALERT_3_B Poor Data / Parameter Ratio ....................       6.44 Note
```

Author Response: This measurement was performed at high pressure which, due to the high

```
PLAT250_ALERT_2_B Large U3/U1 Ratio for <U(i,j)> Tensor(Resd    1)        4.9 Note
```

Author Response: Large errors due to low completeness.

```
PLAT411_ALERT_2_B Short Inter H...H Contact  H003     ..H003     .       1.92 Ang.
                                                1-x,-y,1-z  =      3_656 Check
```

Author Response: H atoms are close to each other at high pressure.

```
PLAT411_ALERT_2_B Short Inter H...H Contact  H004     ..H007     .       1.88 Ang.
                                           -x,-1/2+y,1/2-z  =      2_545 Check
```

Author Response: H atoms are close to each other at high pressure.

```
PLAT411_ALERT_2_B Short Inter H...H Contact  H006     ..H007     .       1.97 Ang.
                                                 -x,1-y,-z  =      3_565 Check
```

Author Response: H atoms are close to each other at high pressure.

```
PLAT911_ALERT_3_B Missing FCF Refl Between Thmin & STh/L=    0.600        282 Report
                2  0  0,   3  0  0,   4  0  0,   5  0  0,   6  0  0,   7  0  0,
                2  1  0,   3  1  0,   4  1  0,   5  1  0,   6  1  0,   7  1  0,
                3  2  0,   4  2  0,   5  2  0,   6  2  0,   7  2  0,   4  3  0,
                5  3  0,   6  3  0,   7  3  0,   4  4  0,   5  4  0,   6  4  0,
                7  4  0,   5  5  0,  -7  1  1,  -6  1  1,  -5  1  1,  -4  1  1,
                   (  252 More Missing: see the .ckf listing file)
```

Author Response: This measurement was performed at high pressure which, due to the high

---

Alert level C

```
PLAT234_ALERT_4_C Large Hirshfeld Difference C005     --C006     .       0.18 Ang.
PLAT340_ALERT_3_C Low Bond Precision on  C-C Bonds ..............    0.00875 Ang.
PLAT411_ALERT_2_C Short Inter H...H Contact  H004     ..H005     .       2.10 Ang.
                                                   x,-1+y,z  =      1_545 Check
```

Author Response: H atoms are close to each other at high pressure.

```
PLAT411_ALERT_2_C Short Inter H...H Contact  H007     ..H007     .       2.08 Ang.
                                                  -x,1-y,-z  =      3_565 Check
```

Author Response: H atoms are close to each other at high pressure.

```
PLAT906_ALERT_3_C Large K Value in the Analysis of Variance ......      5.661 Check
```

Alert level G

```
ABSMU01_ALERT_1_G  Calculation of _exptl_absorpt_correction_mu
                not performed for this radiation type.
PLAT012_ALERT_1_G N.O.K.   _shelx_res_checksum Found in CIF ......     Please Check
PLAT072_ALERT_2_G SHELXL First  Parameter in WGHT  Unusually Large       0.15 Report
PLAT199_ALERT_1_G Reported _cell_measurement_temperature ..... (K)        293 Check
PLAT200_ALERT_1_G Reported   _diffrn_ambient_temperature ..... (K)        293 Check
PLAT432_ALERT_2_G Short Inter X...Y Contact  C001     ..C005     .       2.83 Ang.
                                             x,3/2-y,1/2+z  =      4_576 Check
PLAT432_ALERT_2_G Short Inter X...Y Contact  C001     ..C003     .       2.92 Ang.
                                            x,1/2-y,-1/2+z  =      4_565 Check
PLAT432_ALERT_2_G Short Inter X...Y Contact  C001     ..C006     .       3.05 Ang.
                                             x,3/2-y,1/2+z  =      4_576 Check
PLAT432_ALERT_2_G Short Inter X...Y Contact  C001     ..C005     .       3.13 Ang.
                                          1-x,-1/2+y,1/2-z  =      2_645 Check
PLAT432_ALERT_2_G Short Inter X...Y Contact  C002     ..C005     .       2.80 Ang.
                                             x,3/2-y,1/2+z  =      4_576 Check
PLAT432_ALERT_2_G Short Inter X...Y Contact  C002     ..C006     .       2.92 Ang.
                                             x,3/2-y,1/2+z  =      4_576 Check
PLAT432_ALERT_2_G Short Inter X...Y Contact  C002     ..C005     .       3.14 Ang.
                                          1-x,-1/2+y,1/2-z  =      2_645 Check
PLAT432_ALERT_2_G Short Inter X...Y Contact  C003     ..C003     .       2.87 Ang.
                                                1-x,-y,1-z  =      3_656 Check
PLAT432_ALERT_2_G Short Inter X...Y Contact  C003     ..C005     .       2.94 Ang.
```

```
                                          1-x,-1/2+y,1/2-z  =      2_645 Check
PLAT432_ALERT_2_G Short Inter X...Y Contact  C003     ..C005     .       3.02 Ang.
                                             x,3/2-y,1/2+z  =      4_576 Check
PLAT432_ALERT_2_G Short Inter X...Y Contact  C003     ..C004     .       3.04 Ang.
                                             x,1/2-y,1/2+z  =      4_566 Check
PLAT432_ALERT_2_G Short Inter X...Y Contact  C004     ..C005     .       2.95 Ang.
                                                  x,-1+y,z  =      1_545 Check
PLAT432_ALERT_2_G Short Inter X...Y Contact  C004     ..C006     .       2.99 Ang.
                                           -x,-1/2+y,1/2-z  =      2_545 Check
PLAT432_ALERT_2_G Short Inter X...Y Contact  C004     ..C007     .       3.06 Ang.
                                           -x,-1/2+y,1/2-z  =      2_545 Check
PLAT432_ALERT_2_G Short Inter X...Y Contact  C004     ..C007     .       3.12 Ang.
                                             x,1/2-y,1/2+z  =      4_566 Check
PLAT432_ALERT_2_G Short Inter X...Y Contact  C005     ..C006     .       3.11 Ang.
                                             x,3/2-y,1/2+z  =      4_576 Check
PLAT432_ALERT_2_G Short Inter X...Y Contact  C006     ..C007     .       2.87 Ang.
                                            -x,1/2+y,1/2-z  =      2_555 Check
PLAT432_ALERT_2_G Short Inter X...Y Contact  C007     ..C007     .       3.13 Ang.
                                                 -x,1-y,-z  =      3_565 Check
PLAT720_ALERT_4_G Number of Unusual/Non-Standard Labels ..........         12 Note
              C001    C002    C003    H003    C004    H004    C005    H005
              C006    H006    C007    H007
PLAT802_ALERT_4_G CIF Input Record(s) with more than 80 Characters          3 Info
PLAT883_ALERT_1_G Absent Datum for _atom_sites_solution_primary ..     Please Do !
PLAT910_ALERT_3_G Missing FCF Reflection(s) Below Theta(Min)[Deg]=       2.80 Note
                1  0  0,   1  1  0,
PLAT912_ALERT_4_G Missing # of FCF Reflections Above STh/L=  0.600        484 Note
PLAT913_ALERT_3_G Missing # of Very Strong Reflections in FCF ....          2 Note
               -2  0  2,  -2  1  2,
PLAT933_ALERT_2_G Number of HKL-OMIT Records in Embedded .res File          9 Note
               -6  6  4,  -5  6  3,  -5  7  3,  -4  6  2,  -4  6  3,  -4  7  2,
               -1  2 11,   0  0  2,   0  3 10,
PLAT941_ALERT_3_G Average HKL Measurement Multiplicity ..........         1.5 Low
PLAT950_ALERT_5_G Calculated (ThMax) and CIF-Reported Hmax Differ           7 Units
PLAT956_ALERT_1_G Calculated (ThMax) and Actual (FCF) Hmax Differ           7 Units
PLAT969_ALERT_5_G The 'Henn et al.' R-Factor-gap value ..........     23.459 Note
              Predicted wR2: Based on SigI**2  0.93 or SHELX Weight 21.23
PLAT978_ALERT_2_G Number C-C Bonds with Positive Residual Density.          2 Info
```

```
   1 ALERT level A = Most likely a serious problem - resolve or explain
   6 ALERT level B = A potentially serious problem, consider carefully
   5 ALERT level C = Check. Ensure it is not caused by an omission or oversight
  35 ALERT level G = General information/check it is not something unexpected

   6 ALERT type 1 CIF construction/syntax error, inconsistent or missing data
  27 ALERT type 2 Indicator that the structure model may be wrong or deficient
   8 ALERT type 3 Indicator that the structure quality may be low
   4 ALERT type 4 Improvement, methodology, query or suggestion
   2 ALERT type 5 Informative message, check
```

Datablock: Anthracene_25.3GPa_IAM

Bond precision: C-C = 0.0095 A   Wavelength=0.41000

Cell:	a=7.989(10)	b=5.2110(4)	c=6.9048(5)
	alpha=90	beta=94.05(2)	gamma=90
Temperature:	293 K		

	Calculated	Reported
Volume	286.7(4)	286.7(4)
Space group	P 21/c	P 1 21/c 1
Hall group	-P 2ybc	-P 2ybc
Moiety formula	C14 H10	C14 H10
Sum formula	C14 H10	C14 H10
Mr	178.22	178.22
Dx,g cm-3	2.065	2.064
Z	2	2
Mu (mm-1)	0.055	0.054
F000	188.0	188.0
F000'	187.97	
h,k,lmax	13,9,11	3,8,11
Nref	1549	343
Tmin,Tmax	0.999,0.999	0.267,1.000
Tmin'	0.999	

Correction method= # Reported T Limits: Tmin=0.267 Tmax=1.000
AbsCorr = MULTI-SCAN

Data completeness= 0.221 Theta(max)= 20.804

R(reflections)= 0.0815(239) wR2(reflections)= 0.2608(343)

S = 1.094 Npar= 64

The following ALERTS were generated. Each ALERT has the format
test-name_ALERT_alert-type_alert-level.
Click on the hyperlinks for more details of the test.

Alert level A

PLAT029_ALERT_3_A _diffrn_measured_fraction_theta_full value Low . 0.301 Why?

Author Response: This measurement was performed at high pressure which, due to the high

PLAT088_ALERT_3_A Poor Data / Parameter Ratio .... 5.36 Note

Author Response: This measurement was performed at high pressure which, due to the high

```
Alert level B
PLAT411_ALERT_2_B Short Inter H...H Contact  H003     ..H003     .       1.91 Ang.
                                              1-x,-y,1-z  =      3_656 Check
```

Author Response: H atoms are close to each other at high pressure.

```
PLAT411_ALERT_2_B Short Inter H...H Contact  H004     ..H007     .       1.86 Ang.
                                         -x,-1/2+y,1/2-z  =      2_545 Check
```

Author Response: H atoms are close to each other at high pressure.

```
PLAT411_ALERT_2_B Short Inter H...H Contact  H006     ..H007     .       1.93 Ang.
                                               -x,1-y,-z  =      3_565 Check
```

Author Response: H atoms are close to each other at high pressure.

```
PLAT911_ALERT_3_B Missing FCF Refl Between Thmin & STh/L=    0.600        113 Report
                2  0  0,   3  0  0,   2  1  0,   3  1  0,   1  2  0,   2  2  0,
                3  2  0,   1  3  0,   2  3  0,   3  3  0,   0  4  0,   3  4  0,
                1  5  0,   3  5  0,   0  6  0,   1  6  0,  -3  1  1,  -2  1  1,
               -1  1  1,   1  1  1,   2  1  1,   3  1  1,  -3  2  1,  -2  2  1,
                2  2  1,   3  2  1,  -3  3  1,  -2  3  1,   2  3  1,   3  3  1,
                   (   83 More Missing: see the .ckf listing file)
```

Author Response: This measurement was performed at high pressure which, due to the high

```
Alert level C
PLAT084_ALERT_3_C High wR2 Value (i.e. > 0.25) ...................       0.26 Report
PLAT148_ALERT_3_C s.u. on the      a    - Axis is (Too) Large ....      0.010 Ang.
PLAT340_ALERT_3_C Low Bond Precision on  C-C Bonds ..............     0.0095 Ang.
PLAT411_ALERT_2_C Short Inter H...H Contact  H004     ..H005     .       2.01 Ang.
                                                x,-1+y,z  =      1_545 Check
```

Author Response: H atoms are close to each other at high pressure.

```
PLAT411_ALERT_2_C Short Inter H...H Contact  H007     ..H007     .       2.05 Ang.
                                               -x,1-y,-z  =      3_565 Check
```

Author Response: H atoms are close to each other at high pressure.

```
PLAT906_ALERT_3_C Large K Value in the Analysis of Variance ......      2.687 Check
```

```
Alert level G
```

```
ABSMU01_ALERT_1_G  Calculation of _exptl_absorpt_correction_mu
                not performed for this radiation type.
PLAT003_ALERT_2_G Number of Uiso or U(i,j)  Restrained non-H-Atoms          1 Report
PLAT012_ALERT_1_G N.O.K.   _shelx_res_checksum Found in CIF ......     Please Check
PLAT072_ALERT_2_G SHELXL First  Parameter in WGHT  Unusually Large       0.20 Report
PLAT186_ALERT_4_G The CIF-Embedded .res File Contains ISOR Records          1 Report
PLAT199_ALERT_1_G Reported _cell_measurement_temperature ..... (K)        293 Check
PLAT200_ALERT_1_G Reported   _diffrn_ambient_temperature ..... (K)        293 Check
PLAT432_ALERT_2_G Short Inter X...Y Contact  C001     ..C005     .       2.80 Ang.
                                              x,3/2-y,1/2+z  =      4_576 Check
PLAT432_ALERT_2_G Short Inter X...Y Contact  C001     ..C003     .       2.88 Ang.
                                             x,1/2-y,-1/2+z  =      4_565 Check
PLAT432_ALERT_2_G Short Inter X...Y Contact  C001     ..C006     .       3.00 Ang.
                                              x,3/2-y,1/2+z  =      4_576 Check
PLAT432_ALERT_2_G Short Inter X...Y Contact  C001     ..C005     .       3.14 Ang.
                                          1-x,-1/2+y,1/2-z  =      2_645 Check
PLAT432_ALERT_2_G Short Inter X...Y Contact  C001     ..C002     .       3.18 Ang.
                                          1-x,-1/2+y,1/2-z  =      2_645 Check
PLAT432_ALERT_2_G Short Inter X...Y Contact  C002     ..C005     .       2.78 Ang.
                                              x,3/2-y,1/2+z  =      4_576 Check
PLAT432_ALERT_2_G Short Inter X...Y Contact  C002     ..C006     .       2.88 Ang.
                                              x,3/2-y,1/2+z  =      4_576 Check
PLAT432_ALERT_2_G Short Inter X...Y Contact  C002     ..C005     .       3.12 Ang.
                                          1-x,-1/2+y,1/2-z  =      2_645 Check
PLAT432_ALERT_2_G Short Inter X...Y Contact  C002     ..C003     .       3.18 Ang.
                                             x,1/2-y,-1/2+z  =      4_565 Check
PLAT432_ALERT_2_G Short Inter X...Y Contact  C003     ..C003     .       2.82 Ang.
                                                1-x,-y,1-z  =      3_656 Check
PLAT432_ALERT_2_G Short Inter X...Y Contact  C003     ..C005     .       2.94 Ang.
                                          1-x,-1/2+y,1/2-z  =      2_645 Check
PLAT432_ALERT_2_G Short Inter X...Y Contact  C003     ..C004     .       2.97 Ang.
                                              x,1/2-y,1/2+z  =      4_566 Check
PLAT432_ALERT_2_G Short Inter X...Y Contact  C003     ..C005     .       3.00 Ang.
                                              x,3/2-y,1/2+z  =      4_576 Check
PLAT432_ALERT_2_G Short Inter X...Y Contact  C004     ..C005     .       2.89 Ang.
                                                  x,-1+y,z  =      1_545 Check
PLAT432_ALERT_2_G Short Inter X...Y Contact  C004     ..C006     .       2.95 Ang.
                                           -x,-1/2+y,1/2-z  =      2_545 Check
PLAT432_ALERT_2_G Short Inter X...Y Contact  C004     ..C007     .       3.03 Ang.
                                           -x,-1/2+y,1/2-z  =      2_545 Check
PLAT432_ALERT_2_G Short Inter X...Y Contact  C004     ..C007     .       3.07 Ang.
                                              x,1/2-y,1/2+z  =      4_566 Check
PLAT432_ALERT_2_G Short Inter X...Y Contact  C005     ..C006     .       3.06 Ang.
                                              x,3/2-y,1/2+z  =      4_576 Check
PLAT432_ALERT_2_G Short Inter X...Y Contact  C006     ..C007     .       2.82 Ang.
                                             -x,1/2+y,1/2-z  =      2_555 Check
PLAT432_ALERT_2_G Short Inter X...Y Contact  C007     ..C007     .       3.10 Ang.
                                                 -x,1-y,-z  =      3_565 Check
PLAT720_ALERT_4_G Number of Unusual/Non-Standard Labels ..........         12 Note
              C001    C002    C003    H003    C004    H004    C005    H005
              C006    H006    C007    H007
PLAT802_ALERT_4_G CIF Input Record(s) with more than 80 Characters          3 Info
PLAT860_ALERT_3_G Number of Least-Squares Restraints ............          6 Note
PLAT883_ALERT_1_G Absent Datum for _atom_sites_solution_primary ..     Please Do !
PLAT910_ALERT_3_G Missing FCF Reflection(s) Below Theta(Min)[Deg]=       2.83 Note
                1  0  0,   1  1  0,
PLAT912_ALERT_4_G Missing # of FCF Reflections Above STh/L=  0.600        109 Note
```

```
PLAT913_ALERT_3_G Missing # of Very Strong Reflections in FCF ....          2 Note
               -2  0  2,  -2  1  2,
PLAT933_ALERT_2_G Number of HKL-OMIT Records in Embedded .res File          5 Note
               -1  0  2,   0  3  4,   1  5  5,   2  4  1,   3  7  3,
PLAT941_ALERT_3_G Average HKL Measurement Multiplicity ..........         1.7 Low
PLAT950_ALERT_5_G Calculated (ThMax) and CIF-Reported Hmax Differ          10 Units
PLAT956_ALERT_1_G Calculated (ThMax) and Actual (FCF) Hmax Differ          10 Units
PLAT969_ALERT_5_G The 'Henn et al.' R-Factor-gap value ..........       9.490 Note
              Predicted wR2: Based on SigI**2  2.75 or SHELX Weight 23.85
PLAT978_ALERT_2_G Number C-C Bonds with Positive Residual Density.          0 Info
```

```
   2 ALERT level A = Most likely a serious problem - resolve or explain
   4 ALERT level B = A potentially serious problem, consider carefully
   6 ALERT level C = Check. Ensure it is not caused by an omission or oversight
  40 ALERT level G = General information/check it is not something unexpected

   6 ALERT type 1 CIF construction/syntax error, inconsistent or missing data
  29 ALERT type 2 Indicator that the structure model may be wrong or deficient
  11 ALERT type 3 Indicator that the structure quality may be low
   4 ALERT type 4 Improvement, methodology, query or suggestion
   2 ALERT type 5 Informative message, check
```

Datablock: Anthracene_29.0GPa_IAM

Bond precision: C-C = 0.0079 A Wavelength=0.41000

Cell:	a=7.935(11)	b=5.1607(5)	c=6.8359(6)
	alpha=90	beta=93.54(2)	gamma=90
Temperature:	293 K		

	Calculated	Reported
Volume	279.4(4)	279.4(4)
Space group	P 21/c	P 1 21/c 1
Hall group	-P 2ybc	-P 2ybc
Moiety formula	C14 H10	C14 H10
Sum formula	C14 H10	C14 H10
Mr	178.22	178.22
Dx,g cm-3	2.118	2.118
Z	2	2
Mu (mm-1)	0.056	0.055
F000	188.0	188.0
F000'	187.97	
h,k,lmax	13,9,11	3,8,11
Nref	1548	334
Tmin,Tmax	0.999,0.999	0.206,1.000
Tmin'	0.999	

```
Correction method= # Reported T Limits: Tmin=0.206 Tmax=1.000
AbsCorr = MULTI-SCAN

Data completeness= 0.216          Theta(max)= 20.983

R(reflections)= 0.0636( 238)                      wR2(reflections)=
                                                  0.2025( 334)
S = 1.101                   Npar= 64
```

The following ALERTS were generated. Each ALERT has the format
test-name_ALERT_alert-type_alert-level.
Click on the hyperlinks for more details of the test.

Alert level A

PLAT029_ALERT_3_A _diffrn_measured_fraction_theta_full value Low .   0.321 Why?

Author Response: This measurement was performed at high pressure which, due to the high

PLAT088_ALERT_3_A Poor Data / Parameter Ratio ....   5.22 Note

Author Response: This measurement was performed at high pressure which, due to the high

---

Alert level B

PLAT411_ALERT_2_B Short Inter H...H Contact H003   ..H003   .   1.83 Ang.
1-x,-y,1-z =   3_656 Check

Author Response: H atoms are close to each other at high pressure.

PLAT411_ALERT_2_B Short Inter H...H Contact H004   ..H005   .   1.95 Ang.
x,-1+y,z =   1_545 Check

Author Response: H atoms are close to each other at high pressure.

PLAT411_ALERT_2_B Short Inter H...H Contact H004   ..H007   .   1.82 Ang.
-x,-1/2+y,1/2-z =   2_545 Check

Author Response: H atoms are close to each other at high pressure.

PLAT411_ALERT_2_B Short Inter H...H Contact H006   ..H007   .   1.89 Ang.
-x,1-y,-z =   3_565 Check

Author Response: H atoms are close to each other at high pressure.

```
PLAT911_ALERT_3_B Missing FCF Refl Between Thmin & STh/L=    0.600        101 Report
                2  0  0,   3  0  0,   2  1  0,   3  1  0,   1  2  0,   2  2  0,
                3  2  0,   1  3  0,   2  3  0,   3  3  0,   3  4  0,   3  5  0,
                0  6  0,   1  6  0,  -3  1  1,  -2  1  1,  -1  1  1,   1  1  1,
                2  1  1,   3  1  1,  -3  2  1,  -2  2  1,   2  2  1,   3  2  1,
               -3  3  1,  -2  3  1,   2  3  1,   3  3  1,  -3  4  1,   2  4  1,
                    (   71 More Missing: see the .ckf listing file)
```

Author Response: This measurement was performed at high pressure which, due to the high

---

Alert level C

```
PLAT148_ALERT_3_C s.u. on the      a    - Axis is (Too) Large ....       0.011 Ang.
PLAT234_ALERT_4_C Large Hirshfeld Difference C001     --C004     .        0.16 Ang.
PLAT242_ALERT_2_C Low    'MainMol' Ueq as Compared to Neighbors of       C002 Check
PLAT250_ALERT_2_C Large U3/U1 Ratio for <U(i,j)> Tensor(Resd    1)        2.3 Note
PLAT340_ALERT_3_C Low Bond Precision on  C-C Bonds ..............    0.00788 Ang.
PLAT411_ALERT_2_C Short Inter H...H Contact  H007      ..H007     .        2.04 Ang.
                                                -x,1-y,-z  =      3_565 Check
```

Author Response: H atoms are close to each other at high pressure.

```
PLAT918_ALERT_3_C Reflection(s) with I(obs) much Smaller I(calc) .          1 Check
PLAT939_ALERT_3_C Large Value of Not (SHELXL) Weight Optimized S .      17.08 Check
```

Alert level G

```
ABSMU01_ALERT_1_G  Calculation of _exptl_absorpt_correction_mu
                not performed for this radiation type.
PLAT012_ALERT_1_G N.O.K.   _shelx_res_checksum Found in CIF ......     Please Check
PLAT072_ALERT_2_G SHELXL First  Parameter in WGHT  Unusually Large       0.15 Report
PLAT199_ALERT_1_G Reported _cell_measurement_temperature ..... (K)        293 Check
PLAT200_ALERT_1_G Reported   _diffrn_ambient_temperature ..... (K)        293 Check
PLAT432_ALERT_2_G Short Inter X...Y Contact  C001      ..C005     .        2.77 Ang.
                                            x,3/2-y,1/2+z  =      4_576 Check
PLAT432_ALERT_2_G Short Inter X...Y Contact  C001      ..C003     .        2.85 Ang.
                                           x,1/2-y,-1/2+z  =      4_565 Check
PLAT432_ALERT_2_G Short Inter X...Y Contact  C001      ..C006     .        2.97 Ang.
                                            x,3/2-y,1/2+z  =      4_576 Check
PLAT432_ALERT_2_G Short Inter X...Y Contact  C001      ..C005     .        3.12 Ang.
                                        1-x,-1/2+y,1/2-z  =      2_645 Check
PLAT432_ALERT_2_G Short Inter X...Y Contact  C001      ..C002     .        3.12 Ang.
                                        1-x,-1/2+y,1/2-z  =      2_645 Check
PLAT432_ALERT_2_G Short Inter X...Y Contact  C002      ..C005     .        2.75 Ang.
                                            x,3/2-y,1/2+z  =      4_576 Check
PLAT432_ALERT_2_G Short Inter X...Y Contact  C002      ..C006     .        2.85 Ang.
                                            x,3/2-y,1/2+z  =      4_576 Check
PLAT432_ALERT_2_G Short Inter X...Y Contact  C002      ..C005     .        3.09 Ang.
                                        1-x,-1/2+y,1/2-z  =      2_645 Check
PLAT432_ALERT_2_G Short Inter X...Y Contact  C002      ..C003     .        3.14 Ang.
                                           x,1/2-y,-1/2+z  =      4_565 Check
PLAT432_ALERT_2_G Short Inter X...Y Contact  C002      ..C003     .        3.15 Ang.
                                         1-x,1/2+y,1/2-z  =      2_655 Check
```

```
PLAT432_ALERT_2_G Short Inter X...Y Contact  C003     ..C003     .       2.76 Ang.
                                              1-x,-y,1-z  =      3_656 Check
PLAT432_ALERT_2_G Short Inter X...Y Contact  C003     ..C005     .       2.91 Ang.
                                        1-x,-1/2+y,1/2-z  =      2_645 Check
PLAT432_ALERT_2_G Short Inter X...Y Contact  C003     ..C004     .       2.94 Ang.
                                           x,1/2-y,1/2+z  =      4_566 Check
PLAT432_ALERT_2_G Short Inter X...Y Contact  C003     ..C005     .       2.97 Ang.
                                           x,3/2-y,1/2+z  =      4_576 Check
PLAT432_ALERT_2_G Short Inter X...Y Contact  C003     ..C007     .       3.19 Ang.
                                           x,1/2-y,1/2+z  =      4_566 Check
PLAT432_ALERT_2_G Short Inter X...Y Contact  C003     ..C005     .       3.19 Ang.
                                                x,-1+y,z  =      1_545 Check
PLAT432_ALERT_2_G Short Inter X...Y Contact  C004     ..C005     .       2.85 Ang.
                                                x,-1+y,z  =      1_545 Check
PLAT432_ALERT_2_G Short Inter X...Y Contact  C004     ..C006     .       2.91 Ang.
                                         -x,-1/2+y,1/2-z  =      2_545 Check
PLAT432_ALERT_2_G Short Inter X...Y Contact  C004     ..C007     .       2.99 Ang.
                                         -x,-1/2+y,1/2-z  =      2_545 Check
PLAT432_ALERT_2_G Short Inter X...Y Contact  C004     ..C007     .       3.03 Ang.
                                           x,1/2-y,1/2+z  =      4_566 Check
PLAT432_ALERT_2_G Short Inter X...Y Contact  C005     ..C006     .       3.01 Ang.
                                           x,3/2-y,1/2+z  =      4_576 Check
PLAT432_ALERT_2_G Short Inter X...Y Contact  C006     ..C007     .       2.77 Ang.
                                          -x,1/2+y,1/2-z  =      2_555 Check
PLAT432_ALERT_2_G Short Inter X...Y Contact  C007     ..C007     .       3.06 Ang.
                                               -x,1-y,-z  =      3_565 Check
PLAT432_ALERT_2_G Short Inter X...Y Contact  C007     ..C007     .       3.18 Ang.
                                          -x,1/2+y,1/2-z  =      2_555 Check
PLAT432_ALERT_2_G Short Inter X...Y Contact  C007     ..C007     .       3.18 Ang.
                                         -x,-1/2+y,1/2-z  =      2_545 Check
PLAT720_ALERT_4_G Number of Unusual/Non-Standard Labels ..........         12 Note
              C001    C002    C003    H003    C004    H004    C005    H005
              C006    H006    C007    H007
PLAT802_ALERT_4_G CIF Input Record(s) with more than 80 Characters          3 Info
PLAT883_ALERT_1_G Absent Datum for _atom_sites_solution_primary ..     Please Do !
PLAT910_ALERT_3_G Missing FCF Reflection(s) Below Theta(Min)[Deg]=       2.85 Note
               1  0  0,   1  1  0,
PLAT912_ALERT_4_G Missing # of FCF Reflections Above STh/L=  0.600        130 Note
PLAT913_ALERT_3_G Missing # of Very Strong Reflections in FCF ....          2 Note
              -2  0  2,  -2  1  2,
PLAT933_ALERT_2_G Number of HKL-OMIT Records in Embedded .res File          1 Note
              -1  4  9,
PLAT941_ALERT_3_G Average HKL Measurement Multiplicity ...........        1.6 Low
PLAT950_ALERT_5_G Calculated (ThMax) and CIF-Reported Hmax Differ          10 Units
PLAT956_ALERT_1_G Calculated (ThMax) and Actual (FCF) Hmax Differ          10 Units
PLAT969_ALERT_5_G The 'Henn et al.' R-Factor-gap value ...........     35.171 Note
              Predicted wR2: Based on SigI**2  0.58 or SHELX Weight 18.39
PLAT978_ALERT_2_G Number C-C Bonds with Positive Residual Density.          1 Info
```

---

2 **ALERT level A** = Most likely a serious problem - resolve or explain
5 **ALERT level B** = A potentially serious problem, consider carefully
8 **ALERT level C** = Check. Ensure it is not caused by an omission or oversight
42 **ALERT level G** = General information/check it is not something unexpected

6 ALERT type 1 CIF construction/syntax error, inconsistent or missing data

```
35 ALERT type 2 Indicator that the structure model may be wrong or deficient
10 ALERT type 3 Indicator that the structure quality may be low
 4 ALERT type 4 Improvement, methodology, query or suggestion
 2 ALERT type 5 Informative message, check
```

Datablock: Anthracene_34.0GPa_IAM

Bond precision: C-C = 0.0085 A Wavelength=0.41000

Cell:	a=7.855(16)	b=5.1011(9)	c=6.7534(8)
	alpha=90	beta=93.02(3)	gamma=90
Temperature:	293 K		

	Calculated	Reported
Volume	270.2(6)	270.2(6)
Space group	P 21/c	P 1 21/c 1
Hall group	-P 2ybc	-P 2ybc
Moiety formula	C14 H10	C14 H10
Sum formula	C14 H10	C14 H10
Mr	178.22	178.22
Dx,g cm-3	2.191	2.190
Z	2	2
Mu (mm-1)	0.058	0.057
F000	188.0	188.0
F000'	187.97	
h,k,lmax	13,9,11	3,8,11
Nref	1543	285
Tmin,Tmax	0.999,0.999	0.472,1.000
Tmin'	0.999	

Correction method= # Reported T Limits: Tmin=0.472 Tmax=1.000
AbsCorr = MULTI-SCAN

Data completeness= 0.185 Theta(max)= 21.212

R(reflections)= 0.0563(196) wR2(reflections)= 0.1739(285)

S = 0.997 Npar= 64

The following ALERTS were generated. Each ALERT has the format
test-name_ALERT_alert-type_alert-level.
Click on the hyperlinks for more details of the test.

Alert level A

```
PLAT029_ALERT_3_A _diffrn_measured_fraction_theta_full value Low .      0.284 Why?
```

Author Response: This measurement was performed at high pressure which, due to the high

```
PLAT088_ALERT_3_A Poor Data / Parameter Ratio ....................       4.45 Note
```

Author Response: This measurement was performed at high pressure which, due to the high

```
PLAT411_ALERT_2_A Short Inter H...H Contact  H003     ..H003     .       1.79 Ang.
                                              1-x,-y,1-z  =      3_656 Check
```

Author Response: H atoms are close to each other at high pressure.

```
PLAT411_ALERT_2_A Short Inter H...H Contact  H004     ..H007     .       1.78 Ang.
                                         -x,-1/2+y,1/2-z  =      2_545 Check
```

Author Response: H atoms are close to each other at high pressure.

Alert level B

```
PLAT411_ALERT_2_B Short Inter H...H Contact  H004     ..H005     .       1.95 Ang.
                                                x,-1+y,z  =      1_545 Check
```

Author Response: H atoms are close to each other at high pressure.

```
PLAT411_ALERT_2_B Short Inter H...H Contact  H006     ..H007     .       1.86 Ang.
                                               -x,1-y,-z  =      3_565 Check
```

Author Response: H atoms are close to each other at high pressure.

```
PLAT911_ALERT_3_B Missing FCF Refl Between Thmin & STh/L=    0.600        116 Report
                2  0  0,   3  0  0,   2  1  0,   3  1  0,   0  2  0,   1  2  0,
                2  2  0,   3  2  0,   1  3  0,   2  3  0,   3  3  0,   0  4  0,
                3  4  0,   3  5  0,   0  6  0,   1  6  0,  -3  1  1,  -2  1  1,
               -1  1  1,   1  1  1,   2  1  1,   3  1  1,  -3  2  1,  -2  2  1,
                2  2  1,   3  2  1,  -3  3  1,  -2  3  1,  -1  3  1,   1  3  1,
                   (   86 More Missing: see the .ckf listing file)
```

Author Response: This measurement was performed at high pressure which, due to the high

Alert level C

```
PLAT148_ALERT_3_C s.u. on the      a    - Axis is (Too) Large ....      0.016 Ang.
PLAT234_ALERT_4_C Large Hirshfeld Difference C001     --C004     .       0.17 Ang.
PLAT340_ALERT_3_C Low Bond Precision on  C-C Bonds ..............     0.0085 Ang.
PLAT411_ALERT_2_C Short Inter H...H Contact  H007     ..H007     .       2.00 Ang.
                                                -x,1-y,-z  =      3_565 Check
```

Author Response: H atoms are close to each other at high pressure.

```
PLAT906_ALERT_3_C Large K Value in the Analysis of Variance ......      8.669 Check
PLAT939_ALERT_3_C Large Value of Not (SHELXL) Weight Optimized S .      14.97 Check
```

Alert level G

```
ABSMU01_ALERT_1_G  Calculation of _exptl_absorpt_correction_mu
                not performed for this radiation type.
PLAT003_ALERT_2_G Number of Uiso or U(i,j)  Restrained non-H-Atoms          1 Report
PLAT012_ALERT_1_G N.O.K.   _shelx_res_checksum Found in CIF ......     Please Check
PLAT072_ALERT_2_G SHELXL First  Parameter in WGHT  Unusually Large       0.14 Report
PLAT186_ALERT_4_G The CIF-Embedded .res File Contains ISOR Records          1 Report
PLAT199_ALERT_1_G Reported _cell_measurement_temperature ..... (K)        293 Check
PLAT200_ALERT_1_G Reported   _diffrn_ambient_temperature ..... (K)        293 Check
PLAT432_ALERT_2_G Short Inter X...Y Contact  C001     ..C005     .       2.72 Ang.
                                            x,3/2-y,1/2+z  =      4_576 Check
PLAT432_ALERT_2_G Short Inter X...Y Contact  C001     ..C003     .       2.81 Ang.
                                           x,1/2-y,-1/2+z  =      4_565 Check
PLAT432_ALERT_2_G Short Inter X...Y Contact  C001     ..C006     .       2.90 Ang.
                                            x,3/2-y,1/2+z  =      4_576 Check
PLAT432_ALERT_2_G Short Inter X...Y Contact  C001     ..C005     .       3.09 Ang.
                                        1-x,-1/2+y,1/2-z  =      2_645 Check
PLAT432_ALERT_2_G Short Inter X...Y Contact  C001     ..C002     .       3.09 Ang.
                                        1-x,-1/2+y,1/2-z  =      2_645 Check
PLAT432_ALERT_2_G Short Inter X...Y Contact  C002     ..C005     .       2.70 Ang.
                                            x,3/2-y,1/2+z  =      4_576 Check
PLAT432_ALERT_2_G Short Inter X...Y Contact  C002     ..C006     .       2.78 Ang.
                                            x,3/2-y,1/2+z  =      4_576 Check
PLAT432_ALERT_2_G Short Inter X...Y Contact  C002     ..C005     .       3.04 Ang.
                                        1-x,-1/2+y,1/2-z  =      2_645 Check
PLAT432_ALERT_2_G Short Inter X...Y Contact  C002     ..C003     .       3.10 Ang.
                                           x,1/2-y,-1/2+z  =      4_565 Check
PLAT432_ALERT_2_G Short Inter X...Y Contact  C002     ..C003     .       3.11 Ang.
                                         1-x,1/2+y,1/2-z  =      2_655 Check
PLAT432_ALERT_2_G Short Inter X...Y Contact  C003     ..C003     .       2.71 Ang.
                                               1-x,-y,1-z  =      3_656 Check
PLAT432_ALERT_2_G Short Inter X...Y Contact  C003     ..C005     .       2.86 Ang.
                                        1-x,-1/2+y,1/2-z  =      2_645 Check
PLAT432_ALERT_2_G Short Inter X...Y Contact  C003     ..C004     .       2.91 Ang.
                                            x,1/2-y,1/2+z  =      4_566 Check
PLAT432_ALERT_2_G Short Inter X...Y Contact  C003     ..C005     .       2.92 Ang.
                                            x,3/2-y,1/2+z  =      4_576 Check
PLAT432_ALERT_2_G Short Inter X...Y Contact  C003     ..C007     .       3.14 Ang.
                                            x,1/2-y,1/2+z  =      4_566 Check
PLAT432_ALERT_2_G Short Inter X...Y Contact  C003     ..C005     .       3.14 Ang.
```

```
                                                 x,-1+y,z  =      1_545 Check
PLAT432_ALERT_2_G Short Inter X...Y Contact  C004     ..C005     .       2.81 Ang.
                                                 x,-1+y,z  =      1_545 Check
PLAT432_ALERT_2_G Short Inter X...Y Contact  C004     ..C006     .       2.88 Ang.
                                          -x,-1/2+y,1/2-z  =      2_545 Check
PLAT432_ALERT_2_G Short Inter X...Y Contact  C004     ..C007     .       2.94 Ang.
                                          -x,-1/2+y,1/2-z  =      2_545 Check
PLAT432_ALERT_2_G Short Inter X...Y Contact  C004     ..C007     .       2.97 Ang.
                                             x,1/2-y,1/2+z  =      4_566 Check
PLAT432_ALERT_2_G Short Inter X...Y Contact  C004     ..C006     .       3.19 Ang.
                                             x,3/2-y,1/2+z  =      4_576 Check
PLAT432_ALERT_2_G Short Inter X...Y Contact  C005     ..C006     .       2.96 Ang.
                                             x,3/2-y,1/2+z  =      4_576 Check
PLAT432_ALERT_2_G Short Inter X...Y Contact  C006     ..C007     .       2.74 Ang.
                                            -x,1/2+y,1/2-z  =      2_555 Check
PLAT432_ALERT_2_G Short Inter X...Y Contact  C007     ..C007     .       3.02 Ang.
                                                -x,1-y,-z  =      3_565 Check
PLAT432_ALERT_2_G Short Inter X...Y Contact  C007     ..C007     .       3.14 Ang.
                                            -x,1/2+y,1/2-z  =      2_555 Check
PLAT432_ALERT_2_G Short Inter X...Y Contact  C007     ..C007     .       3.14 Ang.
                                          -x,-1/2+y,1/2-z  =      2_545 Check
PLAT720_ALERT_4_G Number of Unusual/Non-Standard Labels ..........         12 Note
              C001    C002    C003    H003    C004    H004    C005    H005
              C006    H006    C007    H007
PLAT802_ALERT_4_G CIF Input Record(s) with more than 80 Characters          3 Info
PLAT860_ALERT_3_G Number of Least-Squares Restraints .............          6 Note
PLAT883_ALERT_1_G Absent Datum for _atom_sites_solution_primary ..     Please Do !
PLAT910_ALERT_3_G Missing FCF Reflection(s) Below Theta(Min)[Deg]=       2.89 Note
                1  0  0,   1  1  0,
PLAT912_ALERT_4_G Missing # of FCF Reflections Above STh/L=  0.600        163 Note
PLAT913_ALERT_3_G Missing # of Very Strong Reflections in FCF ....          2 Note
               -2  0  2,  -2  1  2,
PLAT933_ALERT_2_G Number of HKL-OMIT Records in Embedded .res File          2 Note
               -2  7  5,   3  6  3,
PLAT941_ALERT_3_G Average HKL Measurement Multiplicity ...........        1.5 Low
PLAT950_ALERT_5_G Calculated (ThMax) and CIF-Reported Hmax Differ          10 Units
PLAT956_ALERT_1_G Calculated (ThMax) and Actual (FCF) Hmax Differ          10 Units
PLAT969_ALERT_5_G The 'Henn et al.' R-Factor-gap value ...........     20.963 Note
              Predicted wR2: Based on SigI**2  0.83 or SHELX Weight 17.45
PLAT978_ALERT_2_G Number C-C Bonds with Positive Residual Density.          0 Info
```

```
   4 ALERT level A = Most likely a serious problem - resolve or explain
   3 ALERT level B = A potentially serious problem, consider carefully
   6 ALERT level C = Check. Ensure it is not caused by an omission or oversight
  46 ALERT level G = General information/check it is not something unexpected

   6 ALERT type 1 CIF construction/syntax error, inconsistent or missing data
  35 ALERT type 2 Indicator that the structure model may be wrong or deficient
  11 ALERT type 3 Indicator that the structure quality may be low
   5 ALERT type 4 Improvement, methodology, query or suggestion
   2 ALERT type 5 Informative message, check
```

Datablock: Anthracene_35.5GPa_IAM

Bond precision: C-C = 0.0084 A Wavelength=0.41000

Cell:	a=7.843(16)	b=5.0901(9)	c=6.7422(8)
	alpha=90	beta=92.91(3)	gamma=90
Temperature:	293 K		

	Calculated	Reported
Volume	268.8(6)	268.8(6)
Space group	P 21/c	P 1 21/c 1
Hall group	-P 2ybc	-P 2ybc
Moiety formula	C14 H10	C14 H10
Sum formula	C14 H10	C14 H10
Mr	178.22	178.22
Dx,g cm-3	2.202	2.202
Z	2	2
Mu (mm-1)	0.058	0.057
F000	188.0	188.0
F000’	187.97	
h,k,lmax	13,8,11	3,8,11
Nref	1542	309
Tmin,Tmax	0.999,0.999	0.116,1.000
Tmin’	0.999	

Correction method= # Reported T Limits: Tmin=0.116 Tmax=1.000
AbsCorr = MULTI-SCAN

Data completeness= 0.200 Theta(max)= 21.242

R(reflections)= 0.0558(213) wR2(reflections)= 0.1694(309)

S = 1.021 Npar= 64

The following ALERTS were generated. Each ALERT has the format
test-name_ALERT_alert-type_alert-level.
Click on the hyperlinks for more details of the test.

Alert level A

PLAT029_ALERT_3_A _diffrn_measured_fraction_theta_full value Low . 0.300 Why?

Author Response: This measurement was performed at high pressure which, due to the high

PLAT088_ALERT_3_A Poor Data / Parameter Ratio .... 4.83 Note

Author Response: This measurement was performed at high pressure which, due to the high

Alert level B

```
PLAT411_ALERT_2_B Short Inter H...H Contact  H003     ..H003     .       1.85 Ang.
                                              1-x,-y,1-z  =      3_656 Check
```

Author Response: H atoms are close to each other at high pressure.

```
PLAT411_ALERT_2_B Short Inter H...H Contact  H004     ..H005     .       1.97 Ang.
                                                x,-1+y,z  =      1_545 Check
```

Author Response: H atoms are close to each other at high pressure.

```
PLAT411_ALERT_2_B Short Inter H...H Contact  H004     ..H007     .       1.80 Ang.
                                         -x,-1/2+y,1/2-z  =      2_545 Check
```

Author Response: H atoms are close to each other at high pressure.

```
PLAT411_ALERT_2_B Short Inter H...H Contact  H006     ..H007     .       1.86 Ang.
                                               -x,1-y,-z  =      3_565 Check
```

Author Response: H atoms are close to each other at high pressure.

```
PLAT411_ALERT_2_B Short Inter H...H Contact  H007     ..H007     .       1.99 Ang.
                                               -x,1-y,-z  =      3_565 Check
```

Author Response: H atoms are close to each other at high pressure.

```
PLAT911_ALERT_3_B Missing FCF Refl Between Thmin & STh/L=    0.600        108 Report
                2  0  0,   3  0  0,   2  1  0,   3  1  0,   1  2  0,   2  2  0,
                3  2  0,   1  3  0,   2  3  0,   3  3  0,   2  4  0,   3  4  0,
                3  5  0,   0  6  0,  -3  1  1,  -2  1  1,  -1  1  1,   1  1  1,
                2  1  1,   3  1  1,  -3  2  1,  -2  2  1,  -1  2  1,   2  2  1,
                3  2  1,  -3  3  1,  -2  3  1,   2  3  1,   3  3  1,  -3  4  1,
                   (   78 More Missing: see the .ckf listing file)
```

Author Response: This measurement was performed at high pressure which, due to the high

Alert level C

```
PLAT148_ALERT_3_C s.u. on the      a    - Axis is (Too) Large ....      0.016 Ang.
PLAT234_ALERT_4_C Large Hirshfeld Difference C005     --C006     .       0.19 Ang.
PLAT250_ALERT_2_C Large U3/U1 Ratio for <U(i,j)> Tensor(Resd    1)        3.7 Note
PLAT340_ALERT_3_C Low Bond Precision on  C-C Bonds ..............    0.00837 Ang.
PLAT411_ALERT_2_C Short Inter H...H Contact  H004     ..H007     .       2.12 Ang.
                                            x,1/2-y,1/2+z  =      4_566 Check
```

Author Response: H atoms are close to each other at high pressure.

```
PLAT906_ALERT_3_C Large K Value in the Analysis of Variance ......      2.822 Check
PLAT913_ALERT_3_C Missing # of Very Strong Reflections in FCF ....          5 Note
               -1  1  1,  -2  0  2,  -1  0  2,  -2  1  2,  -1  1  2,
PLAT939_ALERT_3_C Large Value of Not (SHELXL) Weight Optimized S .      16.80 Check
```

Alert level G

```
ABSMU01_ALERT_1_G  Calculation of _exptl_absorpt_correction_mu
                not performed for this radiation type.
PLAT012_ALERT_1_G N.O.K.   _shelx_res_checksum Found in CIF ......     Please Check
PLAT072_ALERT_2_G SHELXL First  Parameter in WGHT  Unusually Large       0.13 Report
PLAT199_ALERT_1_G Reported _cell_measurement_temperature ..... (K)        293 Check
PLAT200_ALERT_1_G Reported   _diffrn_ambient_temperature ..... (K)        293 Check
PLAT432_ALERT_2_G Short Inter X...Y Contact  C001     ..C005     .       2.71 Ang.
                                              x,3/2-y,1/2+z  =     4_576 Check
PLAT432_ALERT_2_G Short Inter X...Y Contact  C001     ..C003     .       2.79 Ang.
                                             x,1/2-y,-1/2+z  =     4_565 Check
PLAT432_ALERT_2_G Short Inter X...Y Contact  C001     ..C006     .       2.89 Ang.
                                              x,3/2-y,1/2+z  =     4_576 Check
PLAT432_ALERT_2_G Short Inter X...Y Contact  C001     ..C005     .       3.06 Ang.
                                          1-x,-1/2+y,1/2-z  =     2_645 Check
PLAT432_ALERT_2_G Short Inter X...Y Contact  C001     ..C002     .       3.10 Ang.
                                          1-x,-1/2+y,1/2-z  =     2_645 Check
PLAT432_ALERT_2_G Short Inter X...Y Contact  C002     ..C005     .       2.67 Ang.
                                              x,3/2-y,1/2+z  =     4_576 Check
PLAT432_ALERT_2_G Short Inter X...Y Contact  C002     ..C006     .       2.75 Ang.
                                              x,3/2-y,1/2+z  =     4_576 Check
PLAT432_ALERT_2_G Short Inter X...Y Contact  C002     ..C005     .       3.05 Ang.
                                          1-x,-1/2+y,1/2-z  =     2_645 Check
PLAT432_ALERT_2_G Short Inter X...Y Contact  C002     ..C003     .       3.09 Ang.
                                             x,1/2-y,-1/2+z  =     4_565 Check
PLAT432_ALERT_2_G Short Inter X...Y Contact  C002     ..C003     .       3.12 Ang.
                                           1-x,1/2+y,1/2-z  =     2_655 Check
PLAT432_ALERT_2_G Short Inter X...Y Contact  C002     ..C003     .       3.19 Ang.
                                                   x,1+y,z  =     1_565 Check
PLAT432_ALERT_2_G Short Inter X...Y Contact  C003     ..C003     .       2.70 Ang.
                                                1-x,-y,1-z  =     3_656 Check
PLAT432_ALERT_2_G Short Inter X...Y Contact  C003     ..C005     .       2.86 Ang.
                                          1-x,-1/2+y,1/2-z  =     2_645 Check
PLAT432_ALERT_2_G Short Inter X...Y Contact  C003     ..C004     .       2.88 Ang.
                                              x,1/2-y,1/2+z  =     4_566 Check
PLAT432_ALERT_2_G Short Inter X...Y Contact  C003     ..C005     .       2.91 Ang.
                                              x,3/2-y,1/2+z  =     4_576 Check
PLAT432_ALERT_2_G Short Inter X...Y Contact  C003     ..C007     .       3.10 Ang.
                                              x,1/2-y,1/2+z  =     4_566 Check
PLAT432_ALERT_2_G Short Inter X...Y Contact  C003     ..C005     .       3.13 Ang.
                                                  x,-1+y,z  =     1_545 Check
PLAT432_ALERT_2_G Short Inter X...Y Contact  C004     ..C005     .       2.80 Ang.
                                                  x,-1+y,z  =     1_545 Check
PLAT432_ALERT_2_G Short Inter X...Y Contact  C004     ..C006     .       2.88 Ang.
                                           -x,-1/2+y,1/2-z  =     2_545 Check
PLAT432_ALERT_2_G Short Inter X...Y Contact  C004     ..C007     .       2.95 Ang.
                                              x,1/2-y,1/2+z  =     4_566 Check
PLAT432_ALERT_2_G Short Inter X...Y Contact  C004     ..C007     .       2.95 Ang.
```

```
                                           -x,-1/2+y,1/2-z  =      2_545 Check
PLAT432_ALERT_2_G Short Inter X...Y Contact  C004     ..C006     .       3.18 Ang.
                                            x,3/2-y,1/2+z  =      4_576 Check
PLAT432_ALERT_2_G Short Inter X...Y Contact  C005     ..C006     .       2.95 Ang.
                                            x,3/2-y,1/2+z  =      4_576 Check
PLAT432_ALERT_2_G Short Inter X...Y Contact  C006     ..C007     .       2.75 Ang.
                                           -x,1/2+y,1/2-z  =      2_555 Check
PLAT432_ALERT_2_G Short Inter X...Y Contact  C007     ..C007     .       3.03 Ang.
                                                -x,1-y,-z  =      3_565 Check
PLAT432_ALERT_2_G Short Inter X...Y Contact  C007     ..C007     .       3.16 Ang.
                                           -x,1/2+y,1/2-z  =      2_555 Check
PLAT432_ALERT_2_G Short Inter X...Y Contact  C007     ..C007     .       3.16 Ang.
                                          -x,-1/2+y,1/2-z  =      2_545 Check
PLAT720_ALERT_4_G Number of Unusual/Non-Standard Labels ..........         12 Note
              C001    C002    C003    H003    C004    H004    C005    H005
              C006    H006    C007    H007
PLAT802_ALERT_4_G CIF Input Record(s) with more than 80 Characters          3 Info
PLAT883_ALERT_1_G Absent Datum for _atom_sites_solution_primary ..     Please Do !
PLAT910_ALERT_3_G Missing FCF Reflection(s) Below Theta(Min)[Deg]=       2.89 Note
                1  0  0,   1  1  0,
PLAT912_ALERT_4_G Missing # of FCF Reflections Above STh/L=  0.600        147 Note
PLAT933_ALERT_2_G Number of HKL-OMIT Records in Embedded .res File          3 Note
               -1  8  3,   0  5  1,   2  2  3,
PLAT941_ALERT_3_G Average HKL Measurement Multiplicity ..........         1.6 Low
PLAT950_ALERT_5_G Calculated (ThMax) and CIF-Reported Hmax Differ          10 Units
PLAT956_ALERT_1_G Calculated (ThMax) and Actual (FCF) Hmax Differ          10 Units
PLAT969_ALERT_5_G The 'Henn et al.' R-Factor-gap value ..........      25.815 Note
              Predicted wR2: Based on SigI**2  0.66 or SHELX Weight 16.58
PLAT978_ALERT_2_G Number C-C Bonds with Positive Residual Density.          2 Info
```

---

   2 **ALERT level A** = Most likely a serious problem - resolve or explain
   6 **ALERT level B** = A potentially serious problem, consider carefully
   8 **ALERT level C** = Check. Ensure it is not caused by an omission or oversight
  43 **ALERT level G** = General information/check it is not something unexpected

   6 ALERT type 1 CIF construction/syntax error, inconsistent or missing data
  37 ALERT type 2 Indicator that the structure model may be wrong or deficient
  10 ALERT type 3 Indicator that the structure quality may be low
   4 ALERT type 4 Improvement, methodology, query or suggestion
   2 ALERT type 5 Informative message, check

Datablock: Anthracene_38.6GPa_IAM

```
Bond precision:    C-C = 0.0096 A            Wavelength=0.41000

Cell:               a=7.816(16)       b=5.0564(7)         c=6.6915(7)
                    alpha=90          beta=92.66(3)       gamma=90
Temperature:        293 K
```

	Calculated	Reported
Volume	264.2(5)	264.2(5)
Space group	P 21/c	P 1 21/c 1
Hall group	-P 2ybc	-P 2ybc
Moiety formula	C14 H10	C14 H10
Sum formula	C14 H10	C14 H10
Mr	178.22	178.22
Dx,g cm-3	2.240	2.241
Z	2	2
Mu (mm-1)	0.059	0.058
F000	188.0	188.0
F000’	187.97	
h,k,lmax	13,8,11	3,8,11
Nref	1485	284
Tmin,Tmax	0.999,0.999	0.344,1.000
Tmin’	0.999	

Correction method= # Reported T Limits: Tmin=0.344 Tmax=1.000
AbsCorr = MULTI-SCAN

Data completeness= 0.191 Theta(max)= 21.051

R(reflections)= 0.0555(196) wR2(reflections)= 0.1724(284)

S = 1.074 Npar= 64

The following ALERTS were generated. Each ALERT has the format
test-name_ALERT_alert-type_alert-level.
Click on the hyperlinks for more details of the test.

Alert level A

PLAT029_ALERT_3_A _diffrn_measured_fraction_theta_full value Low . 0.287 Why?

Author Response: This measurement was performed at high pressure which, due to the high

PLAT088_ALERT_3_A Poor Data / Parameter Ratio .... 4.44 Note

Author Response: This measurement was performed at high pressure which, due to the high

PLAT411_ALERT_2_A Short Inter H...H Contact H004 ..H007 . 1.76 Ang.
-x,-1/2+y,1/2-z = 2_545 Check

Author Response: H atoms are close to each other at high pressure.

Alert level B

```
PLAT411_ALERT_2_B Short Inter H...H Contact  H003     ..H003     .       1.87 Ang.
                                                 1-x,-y,1-z  =      3_656 Check
```

Author Response: H atoms are close to each other at high pressure.

```
PLAT411_ALERT_2_B Short Inter H...H Contact  H004     ..H005     .       1.93 Ang.
                                                   x,-1+y,z  =      1_545 Check
```

Author Response: H atoms are close to each other at high pressure.

```
PLAT411_ALERT_2_B Short Inter H...H Contact  H006     ..H007     .       1.83 Ang.
                                                  -x,1-y,-z  =      3_565 Check
```

Author Response: H atoms are close to each other at high pressure.

```
PLAT411_ALERT_2_B Short Inter H...H Contact  H007     ..H007     .       1.98 Ang.
                                                  -x,1-y,-z  =      3_565 Check
```

Author Response: H atoms are close to each other at high pressure.

```
PLAT911_ALERT_3_B Missing FCF Refl Between Thmin & STh/L=    0.600        112 Report
                2  0  0,   3  0  0,   2  1  0,   3  1  0,   1  2  0,   2  2  0,
                3  2  0,   2  3  0,   3  3  0,   0  4  0,   2  4  0,   3  4  0,
                3  5  0,   0  6  0,  -3  1  1,  -2  1  1,  -1  1  1,   2  1  1,
                3  1  1,  -3  2  1,  -2  2  1,  -1  2  1,   2  2  1,   3  2  1,
               -3  3  1,  -2  3  1,   2  3  1,   3  3  1,  -3  4  1,   1  4  1,
                    (   82 More Missing: see the .ckf listing file)
```

Author Response: This measurement was performed at high pressure which, due to the high

---

Alert level C

```
PLAT148_ALERT_3_C s.u. on the      a    - Axis is (Too) Large ....      0.016 Ang.
PLAT250_ALERT_2_C Large U3/U1 Ratio for <U(i,j)> Tensor(Resd    1)        2.3 Note
PLAT340_ALERT_3_C Low Bond Precision on  C-C Bonds ..............    0.00962 Ang.
PLAT411_ALERT_2_C Short Inter H...H Contact  H004     ..H007     .       2.10 Ang.
                                               x,1/2-y,1/2+z  =      4_566 Check
```

Author Response: H atoms are close to each other at high pressure.

```
PLAT906_ALERT_3_C Large K Value in the Analysis of Variance ......      2.856 Check
PLAT913_ALERT_3_C Missing # of Very Strong Reflections in FCF ....          5 Note
               -1  1  1,  -2  0  2,  -1  0  2,  -2  1  2,  -1  1  2,
PLAT939_ALERT_3_C Large Value of Not (SHELXL) Weight Optimized S .      12.09 Check
```

Alert level G

```
ABSMU01_ALERT_1_G  Calculation of _exptl_absorpt_correction_mu
                not performed for this radiation type.
PLAT012_ALERT_1_G N.O.K.   _shelx_res_checksum Found in CIF ......     Please Check
PLAT072_ALERT_2_G SHELXL First  Parameter in WGHT  Unusually Large       0.13 Report
PLAT199_ALERT_1_G Reported _cell_measurement_temperature ..... (K)        293 Check
PLAT200_ALERT_1_G Reported   _diffrn_ambient_temperature ..... (K)        293 Check
PLAT432_ALERT_2_G Short Inter X...Y Contact  C001     ..C005     .       2.70 Ang.
                                              x,3/2-y,1/2+z  =     4_576 Check
PLAT432_ALERT_2_G Short Inter X...Y Contact  C001     ..C003     .       2.75 Ang.
                                             x,1/2-y,-1/2+z  =     4_565 Check
PLAT432_ALERT_2_G Short Inter X...Y Contact  C001     ..C006     .       2.89 Ang.
                                              x,3/2-y,1/2+z  =     4_576 Check
PLAT432_ALERT_2_G Short Inter X...Y Contact  C001     ..C005     .       3.05 Ang.
                                          1-x,-1/2+y,1/2-z  =     2_645 Check
PLAT432_ALERT_2_G Short Inter X...Y Contact  C001     ..C002     .       3.05 Ang.
                                          1-x,-1/2+y,1/2-z  =     2_645 Check
PLAT432_ALERT_2_G Short Inter X...Y Contact  C001     ..C005     .       3.19 Ang.
                                                   x,-1+y,z  =     1_545 Check
PLAT432_ALERT_2_G Short Inter X...Y Contact  C002     ..C005     .       2.67 Ang.
                                              x,3/2-y,1/2+z  =     4_576 Check
PLAT432_ALERT_2_G Short Inter X...Y Contact  C002     ..C006     .       2.75 Ang.
                                              x,3/2-y,1/2+z  =     4_576 Check
PLAT432_ALERT_2_G Short Inter X...Y Contact  C002     ..C005     .       3.04 Ang.
                                          1-x,-1/2+y,1/2-z  =     2_645 Check
PLAT432_ALERT_2_G Short Inter X...Y Contact  C002     ..C003     .       3.06 Ang.
                                             x,1/2-y,-1/2+z  =     4_565 Check
PLAT432_ALERT_2_G Short Inter X...Y Contact  C002     ..C003     .       3.10 Ang.
                                           1-x,1/2+y,1/2-z  =     2_655 Check
PLAT432_ALERT_2_G Short Inter X...Y Contact  C002     ..C003     .       3.16 Ang.
                                                    x,1+y,z  =     1_565 Check
PLAT432_ALERT_2_G Short Inter X...Y Contact  C003     ..C003     .       2.68 Ang.
                                                 1-x,-y,1-z  =     3_656 Check
PLAT432_ALERT_2_G Short Inter X...Y Contact  C003     ..C004     .       2.86 Ang.
                                              x,1/2-y,1/2+z  =     4_566 Check
PLAT432_ALERT_2_G Short Inter X...Y Contact  C003     ..C005     .       2.87 Ang.
                                          1-x,-1/2+y,1/2-z  =     2_645 Check
PLAT432_ALERT_2_G Short Inter X...Y Contact  C003     ..C005     .       2.90 Ang.
                                              x,3/2-y,1/2+z  =     4_576 Check
PLAT432_ALERT_2_G Short Inter X...Y Contact  C003     ..C007     .       3.09 Ang.
                                              x,1/2-y,1/2+z  =     4_566 Check
PLAT432_ALERT_2_G Short Inter X...Y Contact  C003     ..C005     .       3.11 Ang.
                                                   x,-1+y,z  =     1_545 Check
PLAT432_ALERT_2_G Short Inter X...Y Contact  C004     ..C005     .       2.77 Ang.
                                                   x,-1+y,z  =     1_545 Check
PLAT432_ALERT_2_G Short Inter X...Y Contact  C004     ..C006     .       2.84 Ang.
                                           -x,-1/2+y,1/2-z  =     2_545 Check
PLAT432_ALERT_2_G Short Inter X...Y Contact  C004     ..C007     .       2.91 Ang.
                                           -x,-1/2+y,1/2-z  =     2_545 Check
PLAT432_ALERT_2_G Short Inter X...Y Contact  C004     ..C007     .       2.93 Ang.
                                              x,1/2-y,1/2+z  =     4_566 Check
PLAT432_ALERT_2_G Short Inter X...Y Contact  C004     ..C006     .       3.15 Ang.
                                              x,3/2-y,1/2+z  =     4_576 Check
PLAT432_ALERT_2_G Short Inter X...Y Contact  C005     ..C006     .       2.92 Ang.
                                              x,3/2-y,1/2+z  =     4_576 Check
PLAT432_ALERT_2_G Short Inter X...Y Contact  C006     ..C007     .       2.70 Ang.
                                             -x,1/2+y,1/2-z  =     2_555 Check
```

```
PLAT432_ALERT_2_G Short Inter X...Y Contact  C006     ..C007     .       3.16 Ang.
                                            -x,1-y,-z  =      3_565 Check
PLAT432_ALERT_2_G Short Inter X...Y Contact  C007     ..C007     .       2.99 Ang.
                                            -x,1-y,-z  =      3_565 Check
PLAT432_ALERT_2_G Short Inter X...Y Contact  C007     ..C007     .       3.11 Ang.
                                       -x,1/2+y,1/2-z  =      2_555 Check
PLAT432_ALERT_2_G Short Inter X...Y Contact  C007     ..C007     .       3.11 Ang.
                                      -x,-1/2+y,1/2-z  =      2_545 Check
PLAT720_ALERT_4_G Number of Unusual/Non-Standard Labels .........         12 Note
              C001    C002    C003    H003    C004    H004    C005    H005
              C006    H006    C007    H007
PLAT802_ALERT_4_G CIF Input Record(s) with more than 80 Characters          3 Info
PLAT883_ALERT_1_G Absent Datum for _atom_sites_solution_primary ..     Please Do !
PLAT910_ALERT_3_G Missing FCF Reflection(s) Below Theta(Min)[Deg]=       2.91 Note
                1  0  0,   1  1  0,
PLAT912_ALERT_4_G Missing # of FCF Reflections Above STh/L=  0.600        158 Note
PLAT933_ALERT_2_G Number of HKL-OMIT Records in Embedded .res File          3 Note
                2  3 11,   2  4  8,   2  5  6,
PLAT941_ALERT_3_G Average HKL Measurement Multiplicity ..........         1.6 Low
PLAT950_ALERT_5_G Calculated (ThMax) and CIF-Reported Hmax Differ          10 Units
PLAT956_ALERT_1_G Calculated (ThMax) and Actual (FCF) Hmax Differ          10 Units
PLAT969_ALERT_5_G The 'Henn et al.' R-Factor-gap value ..........     28.408 Note
              Predicted wR2: Based on SigI**2  0.61 or SHELX Weight 16.05
PLAT978_ALERT_2_G Number C-C Bonds with Positive Residual Density.          0 Info
```

---

3 **ALERT level A** = Most likely a serious problem - resolve or explain
5 **ALERT level B** = A potentially serious problem, consider carefully
7 **ALERT level C** = Check. Ensure it is not caused by an omission or oversight
45 **ALERT level G** = General information/check it is not something unexpected

6 ALERT type 1 CIF construction/syntax error, inconsistent or missing data
39 ALERT type 2 Indicator that the structure model may be wrong or deficient
10 ALERT type 3 Indicator that the structure quality may be low
3 ALERT type 4 Improvement, methodology, query or suggestion
2 ALERT type 5 Informative message, check

Datablock: Anthracene_42.3GPa_IAM

Bond precision: C-C = 0.0125 A   Wavelength=0.41000

Cell:	a=7.769(19)	b=5.0266(9)	c=6.6325(8)
	alpha=90	beta=92.14(4)	gamma=90
Temperature:	293 K		

	Calculated	Reported
Volume	258.8(6)	258.8(6)
Space group	P 21/c	P 1 21/c 1
Hall group	-P 2ybc	-P 2ybc
Moiety formula	C14 H10	C14 H10
Sum formula	C14 H10	C14 H10
Mr	178.22	178.22
Dx,g cm-3	2.287	2.287
Z	2	2
Mu (mm-1)	0.060	0.060
F000	188.0	188.0
F000'	187.97	
h,k,lmax	13,8,11	3,8,10
Nref	1394	284
Tmin,Tmax	0.999,0.999	0.535,1.000
Tmin'	0.999	

Correction method= # Reported T Limits: Tmin=0.535 Tmax=1.000
AbsCorr = MULTI-SCAN

Data completeness= 0.204 Theta(max)= 20.708

R(reflections)= 0.0590(175) wR2(reflections)= 0.1940(284)

S = 1.102 Npar= 64

The following ALERTS were generated. Each ALERT has the format
test-name_ALERT_alert-type_alert-level.
Click on the hyperlinks for more details of the test.

Alert level A

PLAT029_ALERT_3_A _diffrn_measured_fraction_theta_full value Low . 0.303 Why?

Author Response: This measurement was performed at high pressure which, due to the high

PLAT088_ALERT_3_A Poor Data / Parameter Ratio .... 4.44 Note

Author Response: This measurement was performed at high pressure which, due to the high

PLAT411_ALERT_2_A Short Inter H...H Contact H004 ..H007 . 1.75 Ang.
-x,-1/2+y,1/2-z = 2_545 Check

Author Response: H atoms are close to each other at high pressure.

Alert level B

```
PLAT340_ALERT_3_B Low Bond Precision on  C-C Bonds ..............     0.0125 Ang.
```

Author Response: Large errors due to low completeness.

```
PLAT411_ALERT_2_B Short Inter H...H Contact  H003     ..H003     .       1.82 Ang.
                                                1-x,-y,1-z  =      3_656 Check
```

Author Response: H atoms are close to each other at high pressure.

```
PLAT411_ALERT_2_B Short Inter H...H Contact  H004     ..H005     .       1.88 Ang.
                                                  x,-1+y,z  =      1_545 Check
```

Author Response: H atoms are close to each other at high pressure.

```
PLAT411_ALERT_2_B Short Inter H...H Contact  H006     ..H007     .       1.80 Ang.
                                                 -x,1-y,-z  =      3_565 Check
```

Author Response: H atoms are close to each other at high pressure.

```
PLAT411_ALERT_2_B Short Inter H...H Contact  H007     ..H007     .       1.98 Ang.
                                                 -x,1-y,-z  =      3_565 Check
```

Author Response: H atoms are close to each other at high pressure.

```
PLAT911_ALERT_3_B Missing FCF Refl Between Thmin & STh/L=    0.600         97 Report
                3  0  0,   2  1  0,   3  1  0,   1  2  0,   2  2  0,   3  2  0,
                1  3  0,   2  3  0,   3  3  0,   2  4  0,   3  4  0,   3  5  0,
                0  6  0,  -3  1  1,  -2  1  1,   2  1  1,   3  1  1,  -3  2  1,
               -2  2  1,  -1  2  1,   2  2  1,   3  2  1,  -3  3  1,  -2  3  1,
                2  3  1,   3  3  1,  -3  4  1,   3  4  1,  -3  5  1,  -1  5  1,
                   (   67 More Missing: see the .ckf listing file)
```

Author Response: This measurement was performed at high pressure which, due to the high

Alert level C

```
PLAT148_ALERT_3_C s.u. on the      a    - Axis is (Too) Large ....      0.019 Ang.
PLAT234_ALERT_4_C Large Hirshfeld Difference C005     --C006     .       0.17 Ang.
PLAT411_ALERT_2_C Short Inter H...H Contact  H004     ..H007     .       2.08 Ang.
                                            x,1/2-y,1/2+z  =      4_566 Check
```

Author Response: H atoms are close to each other at high pressure.

```
PLAT906_ALERT_3_C Large K Value in the Analysis of Variance ......      4.234 Check
PLAT910_ALERT_3_C Missing FCF Reflection(s) Below Theta(Min)[Deg]=       3.55 Note
                1  0  0,   2  0  0,   1  1  0,  -1  1  1,   0  1  1,   1  1  1,
PLAT913_ALERT_3_C Missing # of Very Strong Reflections in FCF ....          5 Note
               -1  1  1,  -2  0  2,  -1  0  2,  -2  1  2,  -1  1  2,
```

Alert level G

```
ABSMU01_ALERT_1_G  Calculation of _exptl_absorpt_correction_mu
                not performed for this radiation type.
PLAT012_ALERT_1_G N.O.K.   _shelx_res_checksum Found in CIF ......     Please Check
PLAT072_ALERT_2_G SHELXL First  Parameter in WGHT  Unusually Large       0.10 Report
PLAT199_ALERT_1_G Reported _cell_measurement_temperature ..... (K)        293 Check
PLAT200_ALERT_1_G Reported   _diffrn_ambient_temperature ..... (K)        293 Check
PLAT432_ALERT_2_G Short Inter X...Y Contact  C001     ..C005     .       2.67 Ang.
                                              x,3/2-y,1/2+z  =     4_576 Check
PLAT432_ALERT_2_G Short Inter X...Y Contact  C001     ..C003     .       2.74 Ang.
                                             x,1/2-y,-1/2+z  =     4_565 Check
PLAT432_ALERT_2_G Short Inter X...Y Contact  C001     ..C006     .       2.85 Ang.
                                              x,3/2-y,1/2+z  =     4_576 Check
PLAT432_ALERT_2_G Short Inter X...Y Contact  C001     ..C005     .       3.04 Ang.
                                          1-x,-1/2+y,1/2-z  =     2_645 Check
PLAT432_ALERT_2_G Short Inter X...Y Contact  C001     ..C002     .       3.04 Ang.
                                          1-x,-1/2+y,1/2-z  =     2_645 Check
PLAT432_ALERT_2_G Short Inter X...Y Contact  C001     ..C005     .       3.16 Ang.
                                                   x,-1+y,z  =     1_545 Check
PLAT432_ALERT_2_G Short Inter X...Y Contact  C002     ..C005     .       2.65 Ang.
                                              x,3/2-y,1/2+z  =     4_576 Check
PLAT432_ALERT_2_G Short Inter X...Y Contact  C002     ..C006     .       2.71 Ang.
                                              x,3/2-y,1/2+z  =     4_576 Check
PLAT432_ALERT_2_G Short Inter X...Y Contact  C002     ..C005     .       3.02 Ang.
                                          1-x,-1/2+y,1/2-z  =     2_645 Check
PLAT432_ALERT_2_G Short Inter X...Y Contact  C002     ..C003     .       3.03 Ang.
                                             x,1/2-y,-1/2+z  =     4_565 Check
PLAT432_ALERT_2_G Short Inter X...Y Contact  C002     ..C003     .       3.06 Ang.
                                           1-x,1/2+y,1/2-z  =     2_655 Check
PLAT432_ALERT_2_G Short Inter X...Y Contact  C002     ..C003     .       3.14 Ang.
                                                   x,1+y,z  =     1_565 Check
PLAT432_ALERT_2_G Short Inter X...Y Contact  C003     ..C003     .       2.66 Ang.
                                                1-x,-y,1-z  =     3_656 Check
PLAT432_ALERT_2_G Short Inter X...Y Contact  C003     ..C004     .       2.83 Ang.
                                              x,1/2-y,1/2+z  =     4_566 Check
PLAT432_ALERT_2_G Short Inter X...Y Contact  C003     ..C005     .       2.83 Ang.
                                          1-x,-1/2+y,1/2-z  =     2_645 Check
PLAT432_ALERT_2_G Short Inter X...Y Contact  C003     ..C005     .       2.87 Ang.
                                              x,3/2-y,1/2+z  =     4_576 Check
PLAT432_ALERT_2_G Short Inter X...Y Contact  C003     ..C007     .       3.06 Ang.
                                              x,1/2-y,1/2+z  =     4_566 Check
PLAT432_ALERT_2_G Short Inter X...Y Contact  C003     ..C005     .       3.08 Ang.
                                                   x,-1+y,z  =     1_545 Check
PLAT432_ALERT_2_G Short Inter X...Y Contact  C004     ..C005     .       2.74 Ang.
                                                   x,-1+y,z  =     1_545 Check
PLAT432_ALERT_2_G Short Inter X...Y Contact  C004     ..C006     .       2.83 Ang.
                                           -x,-1/2+y,1/2-z  =     2_545 Check
PLAT432_ALERT_2_G Short Inter X...Y Contact  C004     ..C007     .       2.89 Ang.
                                              x,1/2-y,1/2+z  =     4_566 Check
PLAT432_ALERT_2_G Short Inter X...Y Contact  C004     ..C007     .       2.90 Ang.
```

```
                                        -x,-1/2+y,1/2-z  =      2_545 Check
PLAT432_ALERT_2_G Short Inter X...Y Contact  C004     ..C006     .       3.13 Ang.
                                         x,3/2-y,1/2+z  =      4_576 Check
PLAT432_ALERT_2_G Short Inter X...Y Contact  C005     ..C006     .       2.88 Ang.
                                         x,3/2-y,1/2+z  =      4_576 Check
PLAT432_ALERT_2_G Short Inter X...Y Contact  C006     ..C007     .       2.67 Ang.
                                        -x,1/2+y,1/2-z  =      2_555 Check
PLAT432_ALERT_2_G Short Inter X...Y Contact  C006     ..C007     .       3.15 Ang.
                                             -x,1-y,-z  =      3_565 Check
PLAT432_ALERT_2_G Short Inter X...Y Contact  C007     ..C007     .       2.98 Ang.
                                             -x,1-y,-z  =      3_565 Check
PLAT432_ALERT_2_G Short Inter X...Y Contact  C007     ..C007     .       3.09 Ang.
                                        -x,-1/2+y,1/2-z  =      2_545 Check
PLAT432_ALERT_2_G Short Inter X...Y Contact  C007     ..C007     .       3.09 Ang.
                                        -x,1/2+y,1/2-z  =      2_555 Check
PLAT720_ALERT_4_G Number of Unusual/Non-Standard Labels ..........         12 Note
              C001    C002    C003    H003    C004    H004    C005    H005
              C006    H006    C007    H007
PLAT802_ALERT_4_G CIF Input Record(s) with more than 80 Characters          3 Info
PLAT883_ALERT_1_G Absent Datum for _atom_sites_solution_primary ..     Please Do !
PLAT912_ALERT_4_G Missing # of FCF Reflections Above STh/L=  0.600        140 Note
PLAT933_ALERT_2_G Number of HKL-OMIT Records in Embedded .res File          2 Note
                0  2  9,   1  4  7,
PLAT941_ALERT_3_G Average HKL Measurement Multiplicity ..........         1.6 Low
PLAT950_ALERT_5_G Calculated (ThMax) and CIF-Reported Hmax Differ          10 Units
PLAT956_ALERT_1_G Calculated (ThMax) and Actual (FCF) Hmax Differ          10 Units
PLAT969_ALERT_5_G The 'Henn et al.' R-Factor-gap value ..........       6.839 Note
              Predicted wR2: Based on SigI**2  2.84 or SHELX Weight 17.60
PLAT978_ALERT_2_G Number C-C Bonds with Positive Residual Density.          0 Info
```

```
  3 ALERT level A = Most likely a serious problem - resolve or explain
  6 ALERT level B = A potentially serious problem, consider carefully
  6 ALERT level C = Check. Ensure it is not caused by an omission or oversight
 44 ALERT level G = General information/check it is not something unexpected

  6 ALERT type 1 CIF construction/syntax error, inconsistent or missing data
 38 ALERT type 2 Indicator that the structure model may be wrong or deficient
  9 ALERT type 3 Indicator that the structure quality may be low
  4 ALERT type 4 Improvement, methodology, query or suggestion
  2 ALERT type 5 Informative message, check
```

It is advisable to attempt to resolve as many as possible of the alerts in all categories. Often the minor alerts point to easily fixed oversights, errors and omissions in your CIF or refinement strategy, so attention to these fine details can be worthwhile. It is up to the individual to critically assess their own results and, if necessary, seek expert advice.

Validation response form

Please find below a validation response form (VRF) that can be filled in and pasted into your CIF.

```
# start Validation Reply Form
_vrf_PLAT901_Anthracene_00.0GPa_IAM
;
PROBLEM: Cell Parameters in CIF and FCF do not Match ....          ! Error
RESPONSE: ...
;
_vrf_PLAT901_Anthracene_04.0GPa_IAM
;
PROBLEM: Cell Parameters in CIF and FCF do not Match ....          ! Error
RESPONSE: ...
;
_vrf_PLAT901_Anthracene_10.8GPa_IAM
;
PROBLEM: Cell Parameters in CIF and FCF do not Match ....          ! Error
RESPONSE: ...
;
# end Validation Reply Form
```

PLATON version of 04/06/2025; check.def file version of 30/05/2025

Datablock Anthracene_00.0GPa_IAM - ellipsoid plot

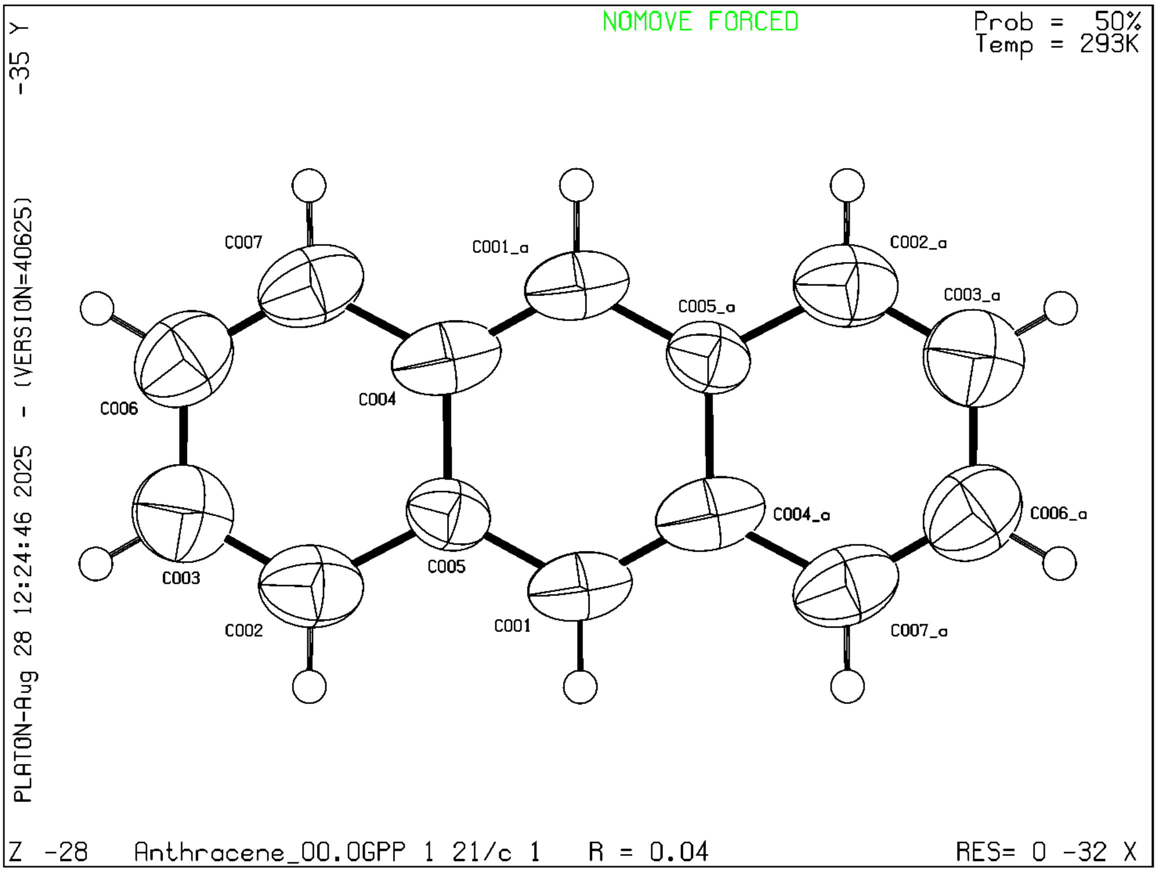

Datablock Anthracene_01.5GPa_IAM - ellipsoid plot

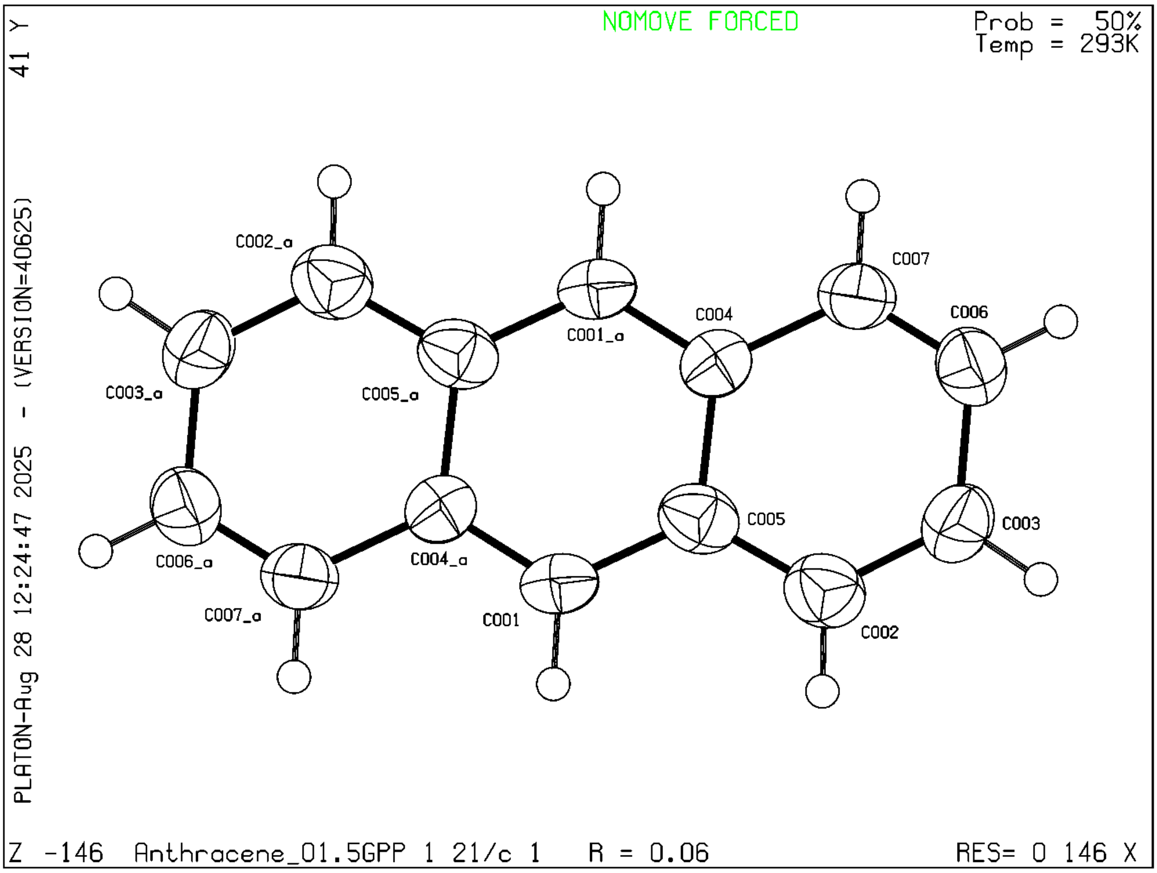

Datablock Anthracene_04.0GPa_IAM - ellipsoid plot

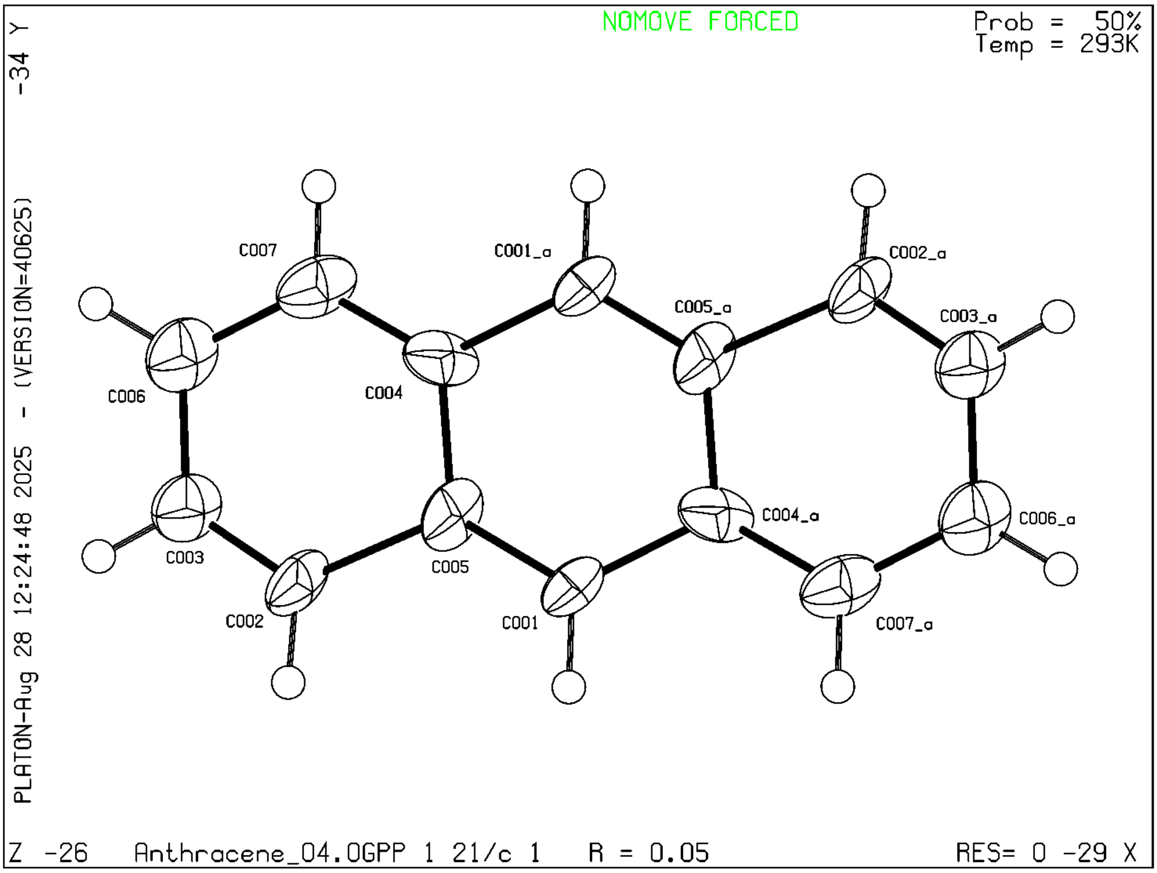

Datablock Anthracene_08.3GPa_IAM - ellipsoid plot

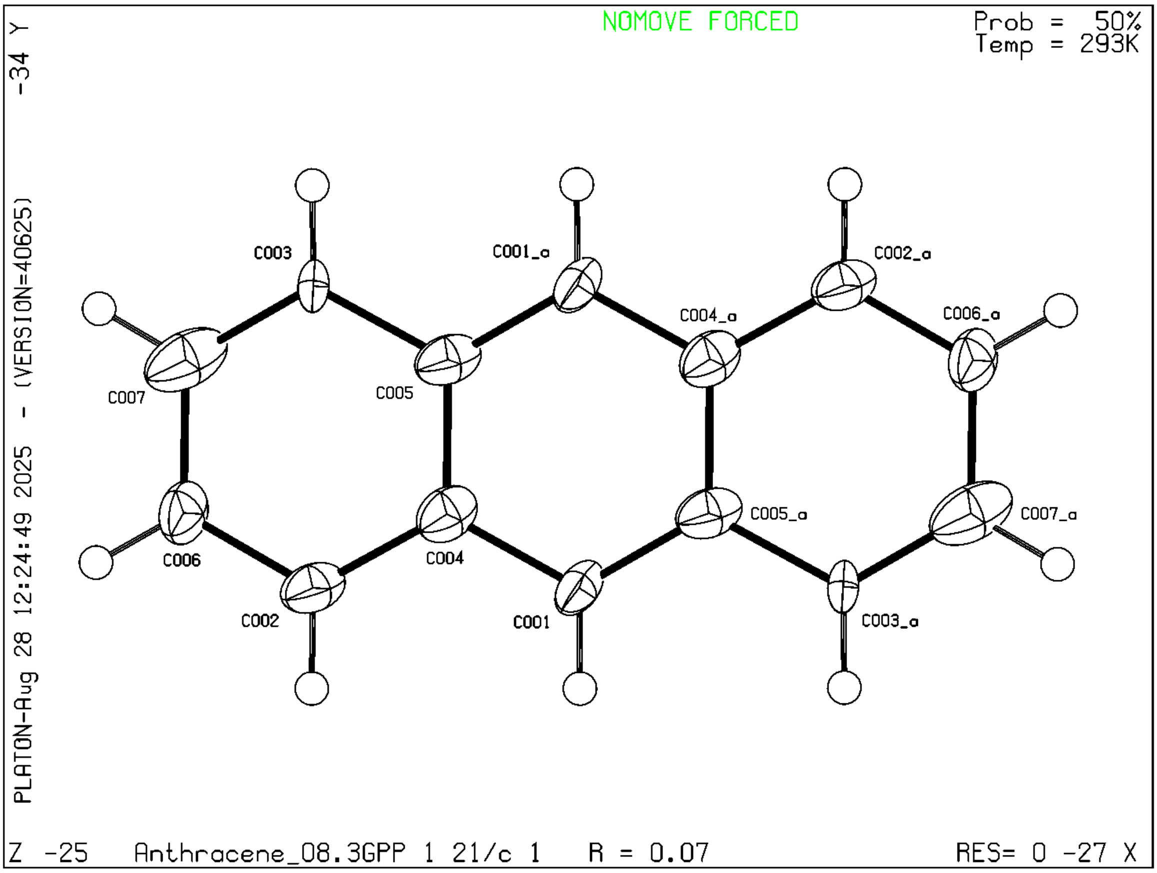

Datablock Anthracene_10.8GPa_IAM - ellipsoid plot

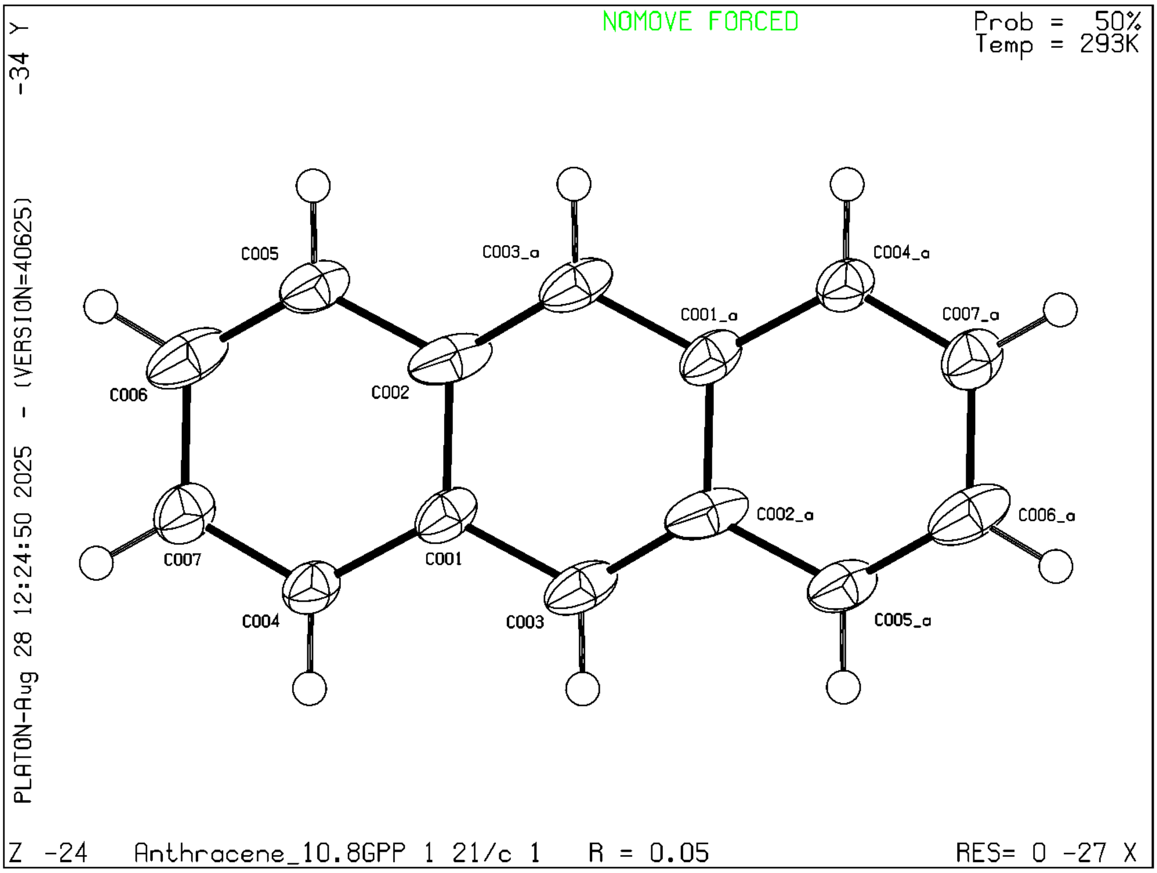

Datablock Anthracene_13.5GPa_IAM - ellipsoid plot

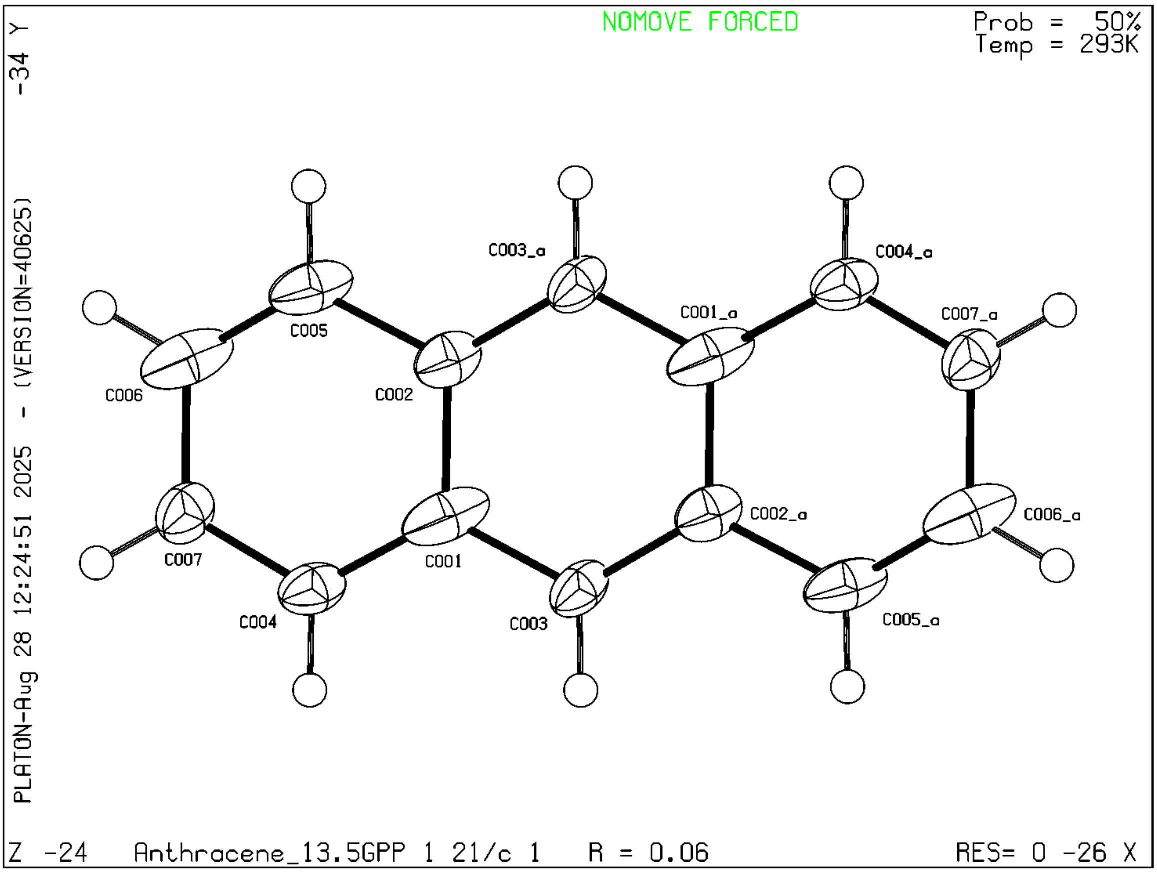

Datablock Anthracene_15.8GPa_IAM - ellipsoid plot

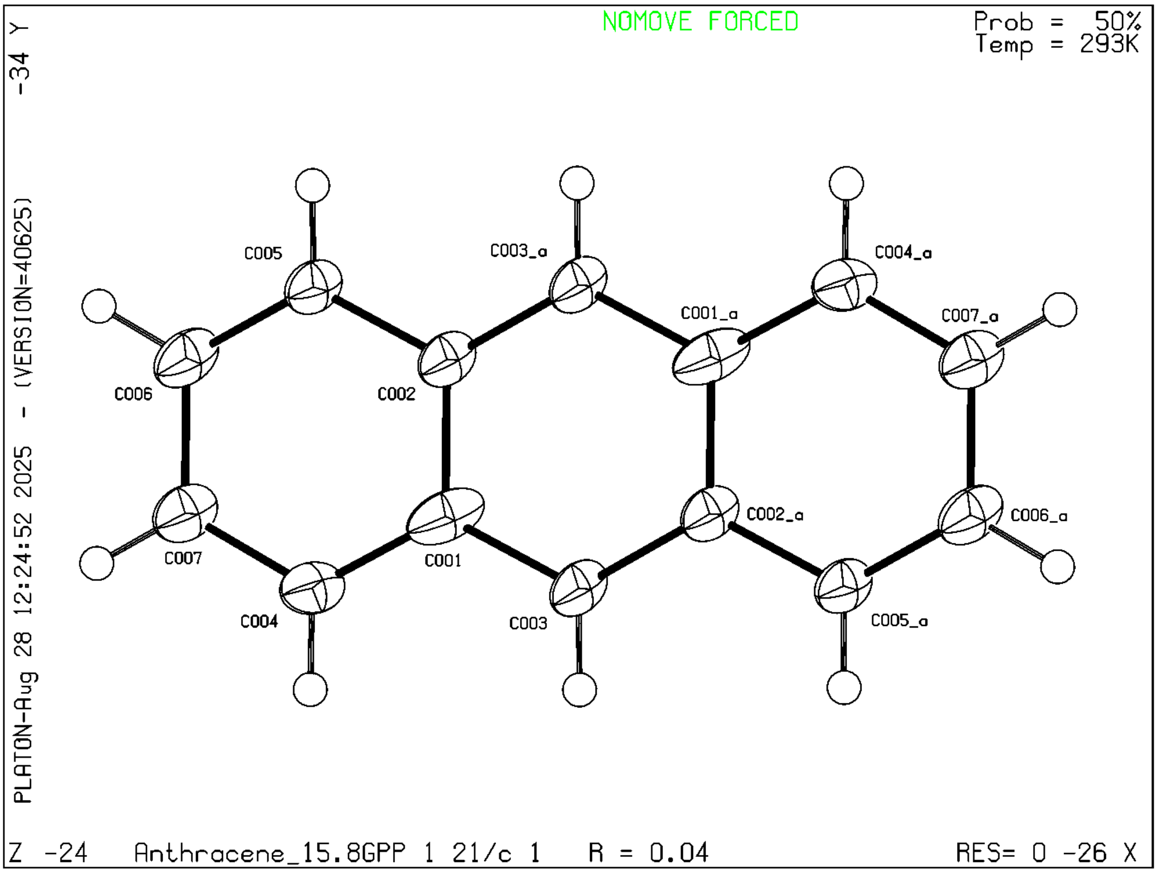

Datablock Anthracene_18.4GPa_IAM - ellipsoid plot

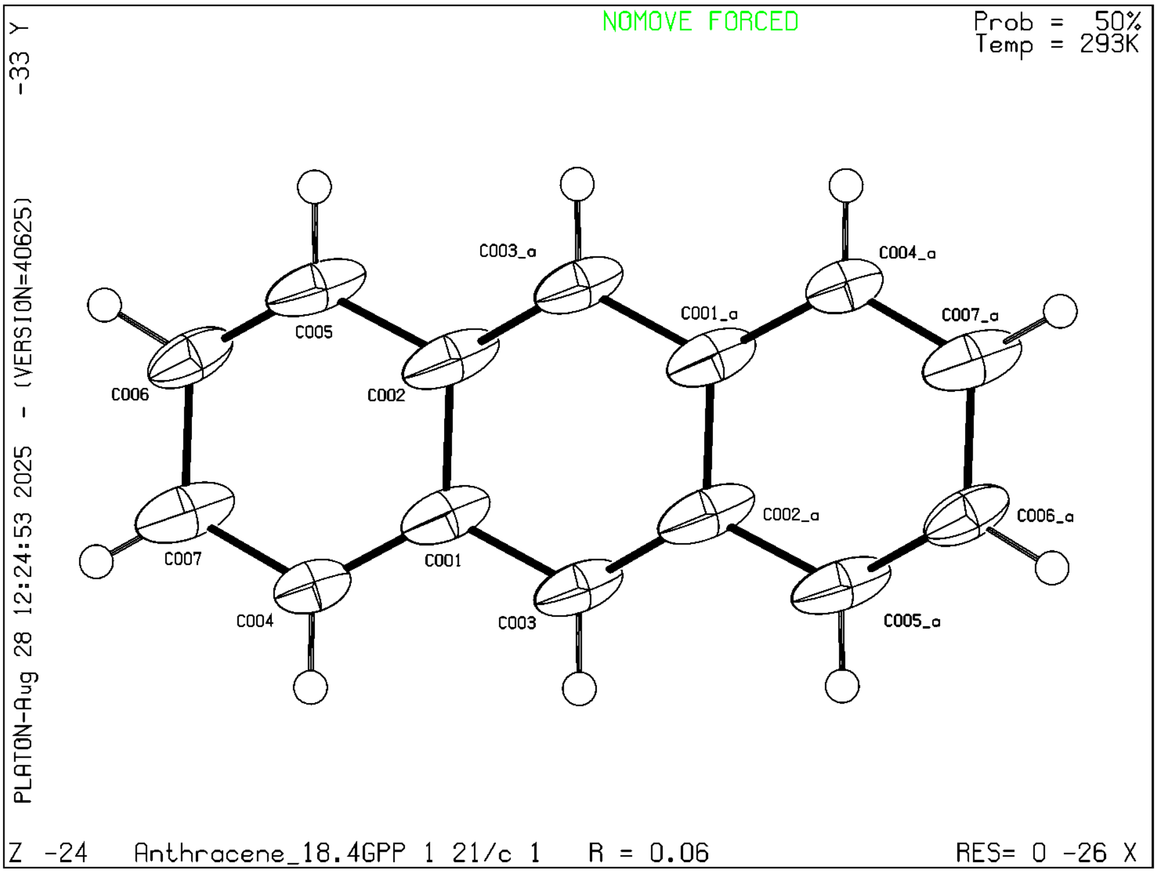

Datablock Anthracene_21.5GPa_IAM - ellipsoid plot

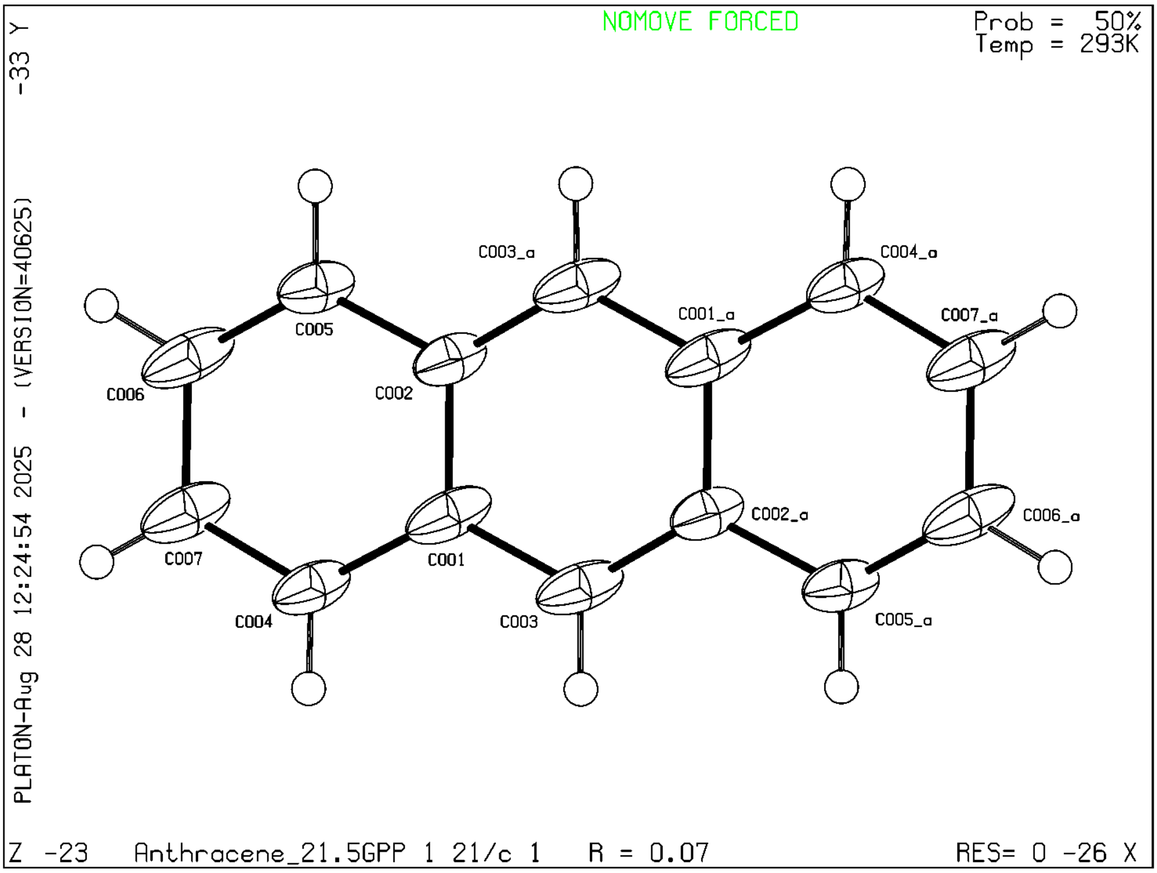

Datablock Anthracene_25.3GPa_IAM - ellipsoid plot

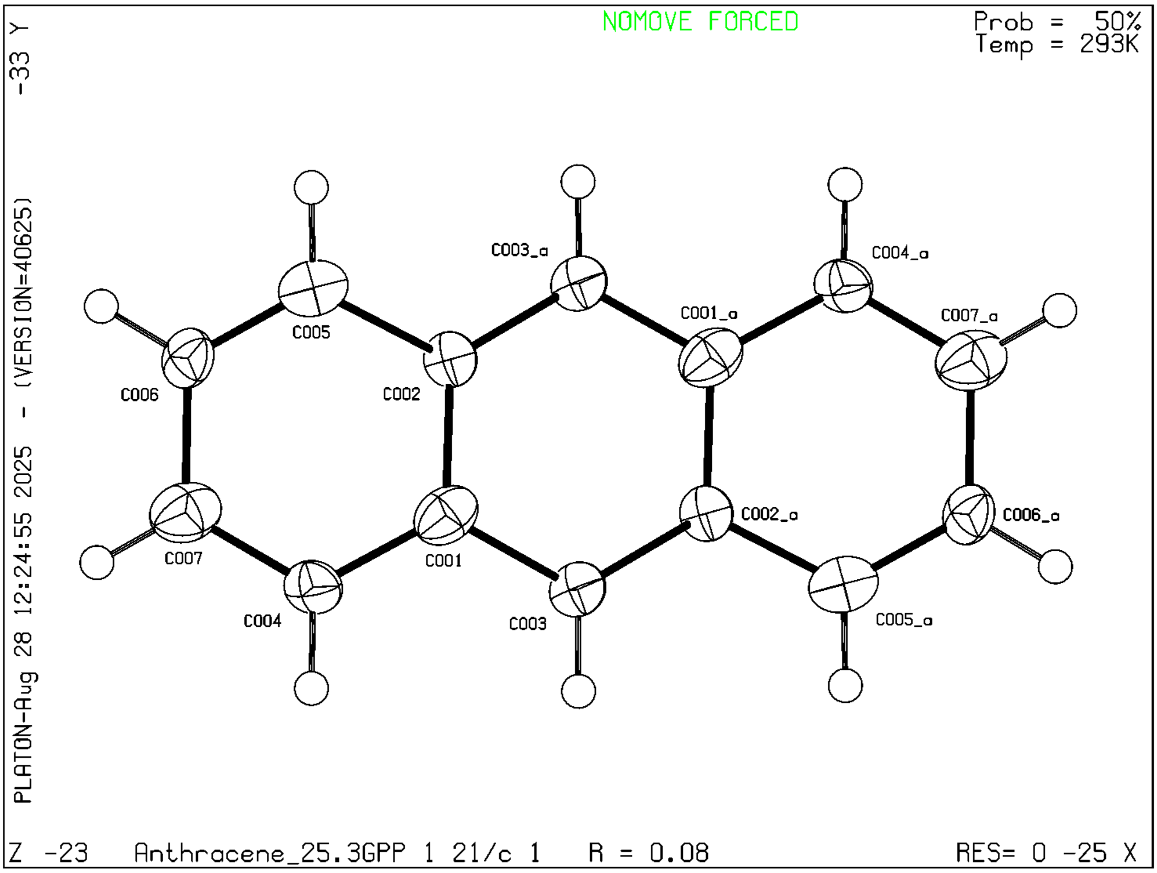

Datablock Anthracene_29.0GPa_IAM - ellipsoid plot

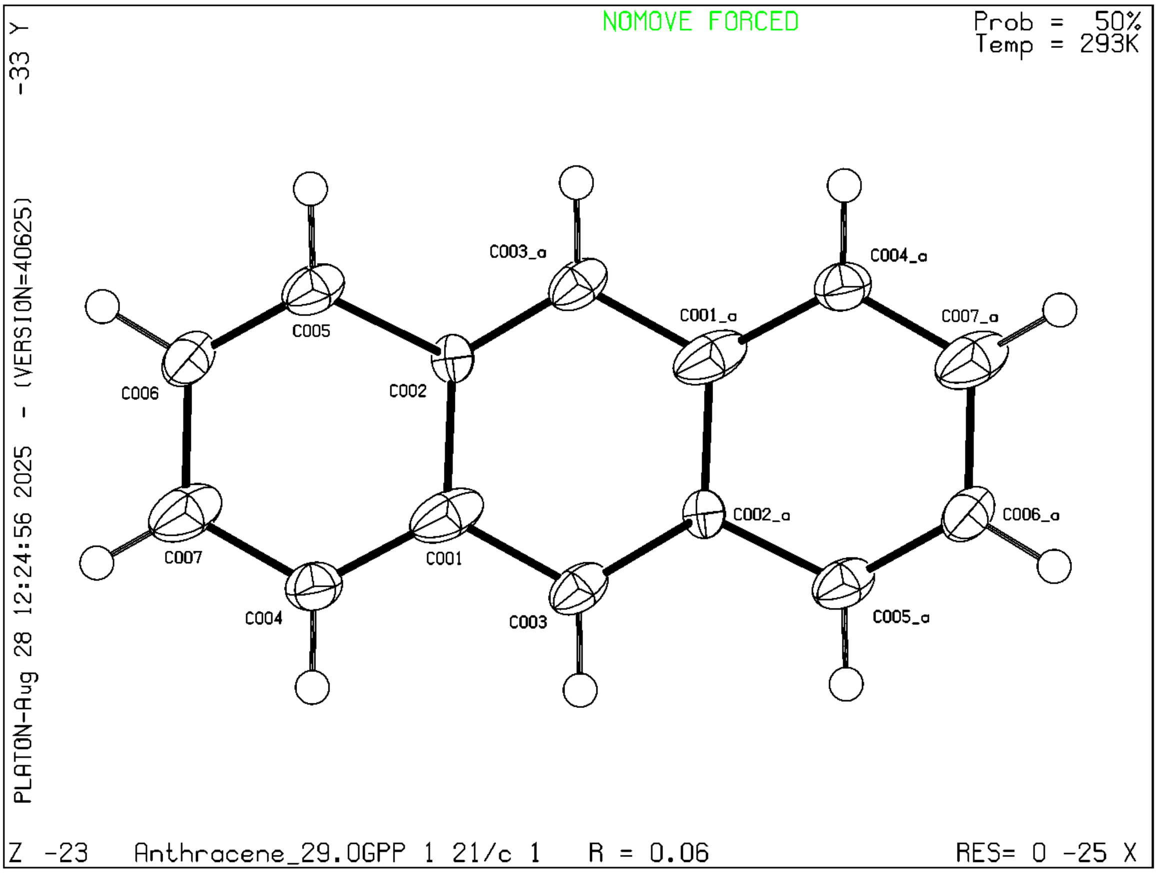

Datablock Anthracene_34.0GPa_IAM - ellipsoid plot

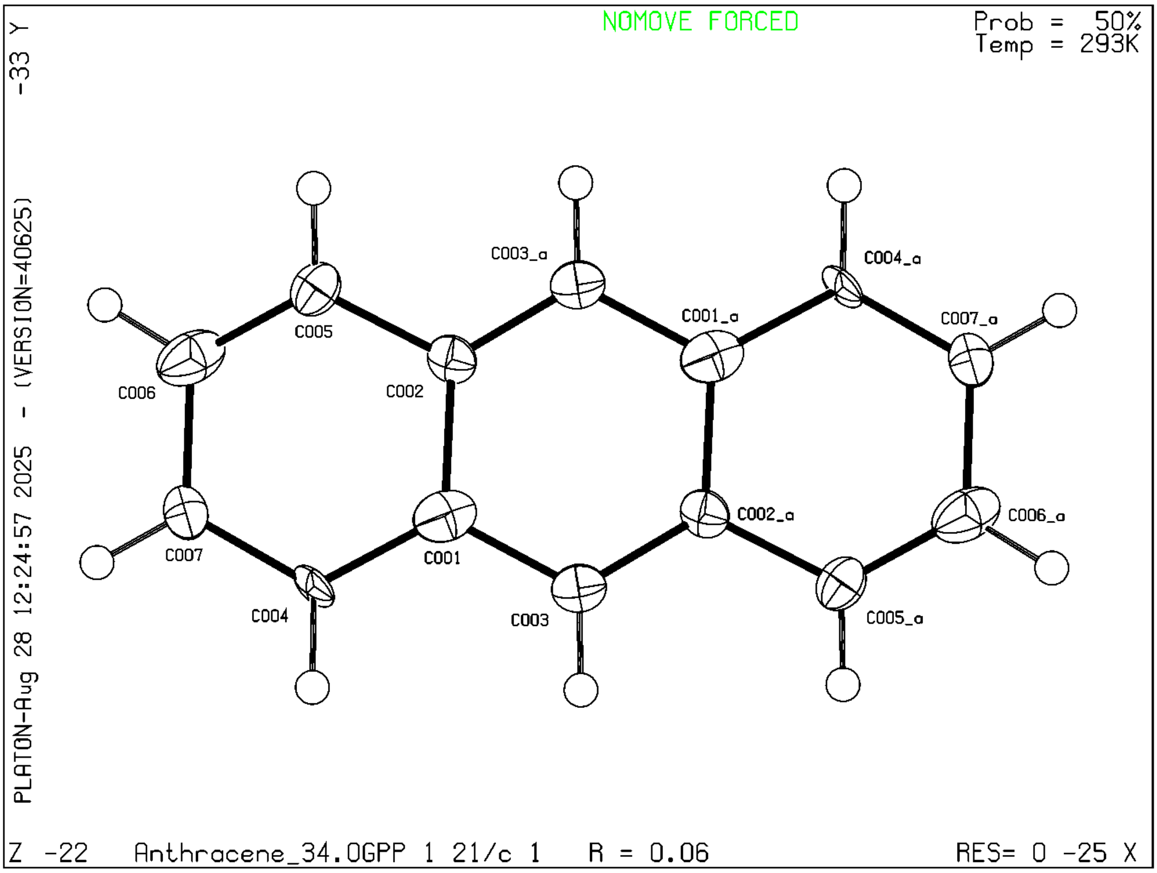

Datablock Anthracene_35.5GPa_IAM - ellipsoid plot

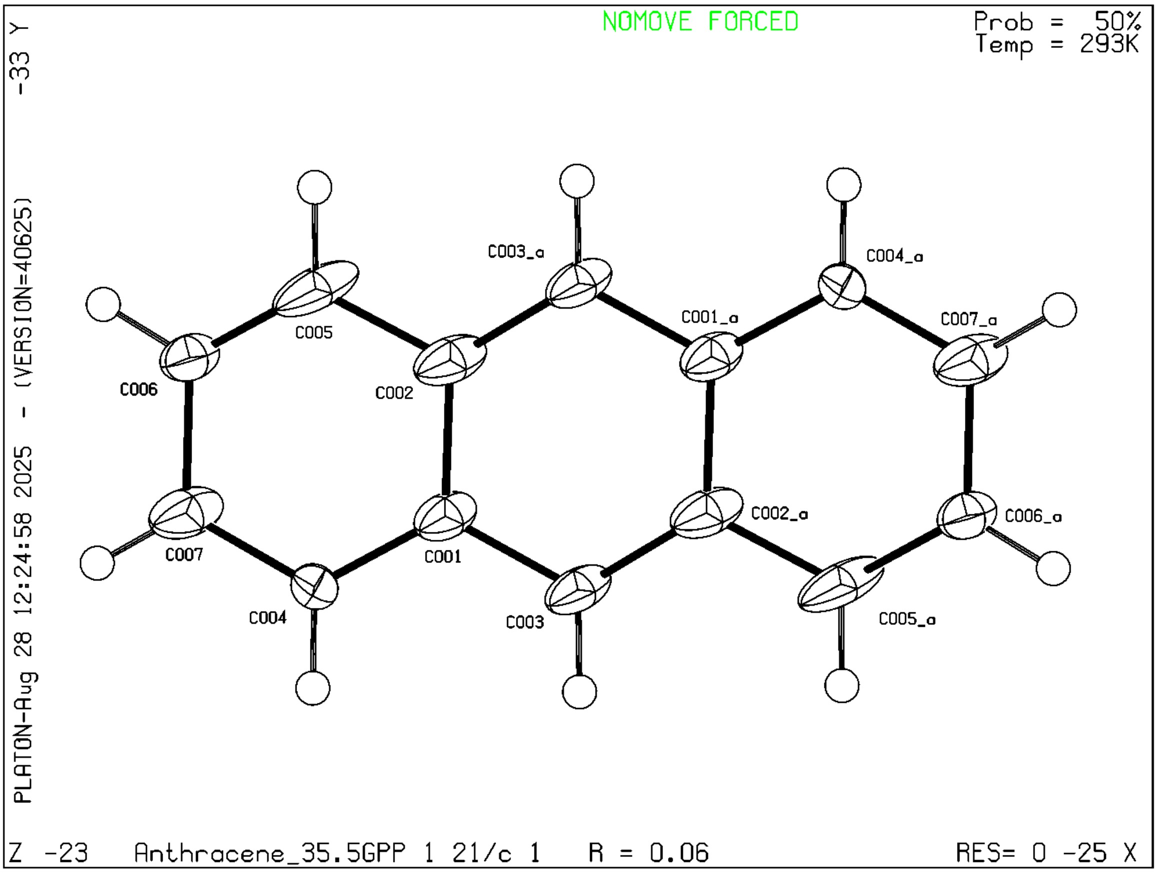

Datablock Anthracene_38.6GPa_IAM - ellipsoid plot

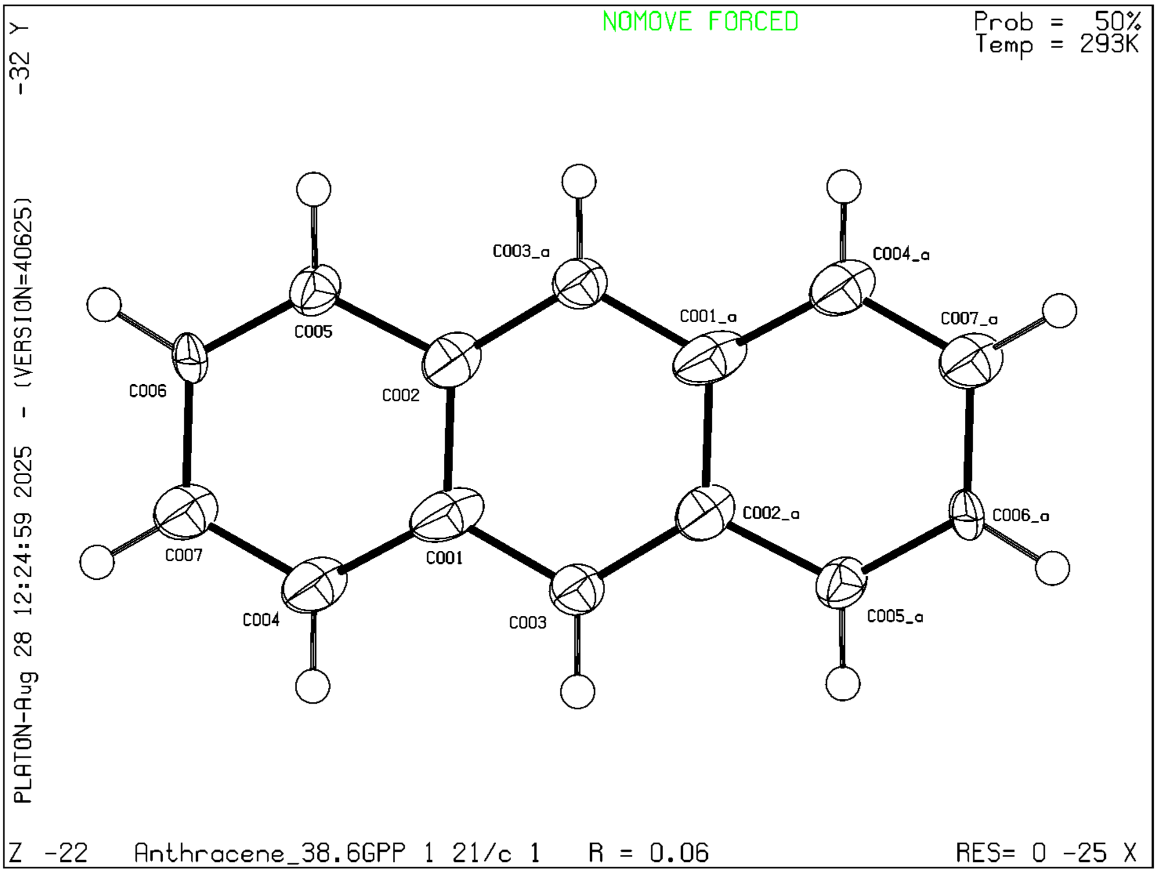

Datablock Anthracene_42.3GPa_IAM - ellipsoid plot

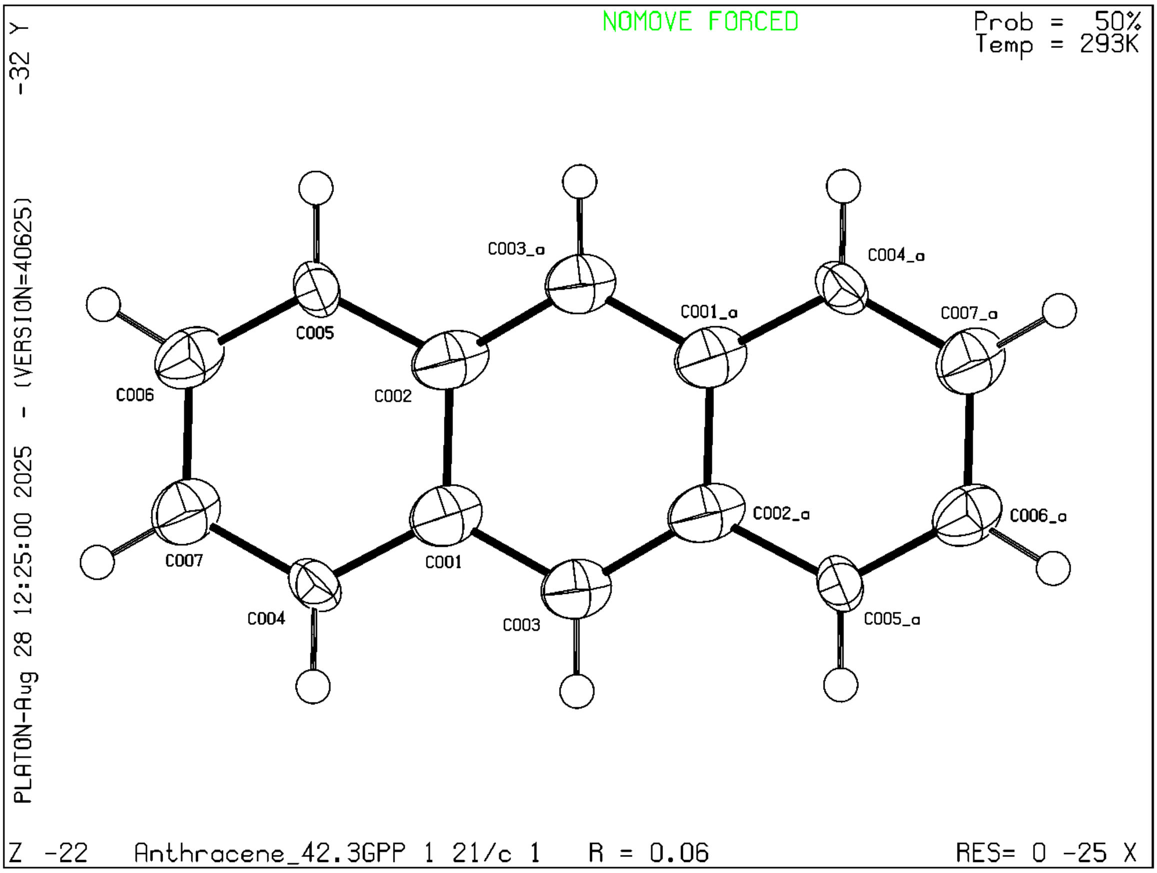

Supplement: Supplementary file 32 [file ao5c06935_si_032.pdf]
